# Stroke: Epidemiology, Risk Factors, Signaling Pathways, and Clinical Management

**DOI:** 10.1002/mco2.70558

**Published:** 2025-12-17

**Authors:** He Ren, Yuchun Liu, Mingyue Zhao, Hangyu Shen, Sheng Nie, Xiang Gao, Yi Huang

**Affiliations:** ^1^ School of Medicine Zhejiang University Hangzhou Zhejiang China; ^2^ Department of Neurosurgery Ningbo Key Laboratory of Nervous System and Brain Function The First Affiliated Hospital of Ningbo University Ningbo Zhejiang China

**Keywords:** cell transplantation, clinical management, clinical treatment, neuroprotection, risk factors, signaling pathways, stem cells, stroke

## Abstract

Stroke can be classified into ischemic stroke (IS), hemorrhagic stroke (HS), and subarachnoid hemorrhage. The high incidence, disability, and mortality rates, especially from IS, place a huge burden on global health. The pathophysiological processes following IS mainly involve energy deficiency, ion homeostasis imbalance, oxidative stress, neuroinflammation, programmed cell death, blood–brain barrier disruption, and cerebral edema. Transient ischemic attack is an early warning sign of IS, characterized by temporary neurological deficits. HS mainly involves primary damage caused by the mass effect of hematoma and secondary damage caused by the toxic components of hematoma. Although advances in acute‐phase reperfusion technology have reduced mortality, the fundamental challenge of a narrow therapeutic window limits patient eligibility and long‐term recovery outcomes. This review aims to provide a comprehensive overview of stroke, detailing its epidemiology, risk factors, pathophysiological mechanisms, signaling pathways, and clinical management methods. Here, we focus on the latest research progress in IS and emphasize the hope that regenerative therapies, especially stem cell therapies, offer for stroke patients. This review aims to provide a detailed overview of current research and clinical practice in stroke, propose emerging strategies for treating stroke patients, and provide an outlook on future research directions in this field.

## Graphical abstract

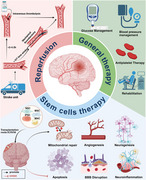

## 1 Introduction

Stroke remains a leading cause of death and disability worldwide. It can be divided into ischemic stroke (IS), hemorrhagic stroke (HS), and subarachnoid hemorrhage (SAH). IS, caused by the obstruction of blood flow to the brain, accounts for approximately 87% of all stroke cases [1, 2]. HS, which results from the rupture of blood vessels and bleeding into the brain tissue [3]. SAH accounts for about 3% of strokes and is more common in young people, characterized by sudden and severe headaches [4]. Stroke has become the primary cause of death and disability in countries with a medium and low‐medium sociodemographic index (SDI) [5]. China, in particular, faces a significant burden, with millions of individuals affected annually [6]. Major risk factors include diabetes, hypertension, hyperlipidemia, and smoking [7].

The pathophysiology of stroke is multifaceted, involving intricate mechanisms that depend on the type of stroke. The brain tissue affected by IS can be divided into the ischemic core and the ischemic penumbra. In the acute phase (within 2 weeks), the ischemic core is characterized by oxidative stress and neurotoxicity resulting from a lack of oxygen, glucose, and ATP. This leads to a cascade of events, including disruption of cellular ionic homeostasis, acidosis, intracellular calcium overload, free radical production, release of arachidonic acid (AA) metabolites, cytokine toxicity, complement activation, BBB disruption, glial activation, and leukocyte infiltration, Collectively, these processes ultimately cause cell damage and apoptosis [8]. The damage in this area is irreversible and cannot be salvaged. In contrast, the blood flow in the ischemic penumbra is insufficient to support normal neurological function but is adequate needed to prevent immediate cell death. Brain cells in this region can be salvaged if blood flow is restored, in a timely manner [8, 9]. Following reperfusion, secondary tissue pulsation and the loss of vascular structural integrity, can lead to vascular barrier disruption and vasogenic edema [10]. During the recovery period, the rebuilding of neurovascular units and the protective effects of neuroinflammation contribute to partial functional recovery in patients. HS leads to mechanical damage due to blood accumulation, increased intracranial pressure (ICP), and subsequent brain injury [11]. Transient ischemic attack (TIA), characterized by transient neurological deficits, serves as a crucial early warning sign or precursor that often precedes a full‐blown stroke, signaling the immediate need for early intervention and prevention [12].

IS is a condition with a wide therapeutic time window, and its clinical manifestations vary at different stages, necessitating targeted treatment approaches [13]. Currently, treatment for IS is limited to the rapid and effective removal of thrombi via intravenous (IV) administration of tissue plasminogen activator (tPA) within 4.5 h of symptom onset or endovascular mechanical thrombectomy within 6 h [14]. The primary goal of these treatments is to restore blood flow to the occluded vessels and salvage the penumbra. However, only a small number of patients are eligible for these interventions. In most medical centers, fewer than 10% of IS patients receive IV thrombolysis (IVT), and it is estimated that only about 7–15% have the opportunity to undergo acute endovascular intervention [15]. Furthermore, vascular reperfusion therapy can lead to various complications, including myocardial ischemic–reperfusion injury, BBB damage, and immune imbalance, among others [16]. For HS, immediate control of blood pressure and surgical interventions may be necessary, but the clinical outcomes often remain poor [11]. In TIA, the primary approach involves risk factor management and the prevention of further cerebrovascular events [17]

At present, the clinical management of IS still faces significant limitations, leading to poor treatment outcomes. In recent years, the rapid development of stem cell biology has offered new hope for the treatment of IS. Stem cells possess strong proliferative capacity, low differentiation level, multipotent differentiation potential, anti‐inflammatory properties, low immunogenicity, immune modulation abilities, and a high secretory capacity. These characteristics have also shown promise in treating other central nervous system (CNS) diseases, such as HS and spinal cord injury [18, 19]. The current strategy for stem cell transplantation in the treatment of IS focuses on salvaging the ischemic penumbra by restoring a new neuronal network [20]. Numerous preclinical and clinical studies on stem cell therapy for stroke have yielded promising results, indicating that stem cells hold great potential for improving outcomes in IS [21]. In animal models, stem cell therapy has demonstrated a range of benefits, including extending the penumbra's salvageable period in the acute phase, inhibiting excessive inflammation in the subacute phase, and promoting neurogenesis and angiogenesis in the chronic phase [22].

Given the significant burden of stroke, the limitations of current clinical management, this review will explore the epidemiology, risk factors, pathophysiological processes, and clinical management of stroke. In addition, this review also introduces regenerative medicine, especially the application of stem cell therapy in IS. Our aim is to summarize the progress and limitations of current stroke research, propose optimization strategies, and look forward to its future development prospects, so as to provide theoretical support for stroke prevention and treatment practices.

## 2 Epidemiology of Stroke

Currently, stroke remains an international public health threat. A clear understanding of the global epidemiology of stroke and the differences in stroke rates worldwide is crucial for guiding public health policies and limiting the harm caused by stroke. In this section, we explore key changes in global stroke epidemiology over the past 30 years, analyze the differences in stroke burden across different countries and regions, races, sexes, and ages, and propose various strategies to mitigate global stroke burden disparities.

### 2.1 The Global Burden of Stroke

Stroke is a focal neurological deficit caused by acute focal damage to the CNS (brain, spinal cord, or retina). Most strokes are IS, which are caused by the occlusion of cerebral arteriovenous or venous sinuses. HS is caused by the rupture of blood vessels in the brain parenchyma or ventricular system due to nontraumatic causes. SAH refers to the occurrence of bleeding in the subarachnoid space between the arachnoid and pia mater due to nontraumatic causes [3, 4, 23]. Stroke is the second leading cause of death worldwide, accounting for 10.7% of all deaths (after ischemic heart disease and COVID‐19). It is also the fourth leading cause of disability‐adjusted life years (DALYs) globally, responsible for 5.6% of all DALYs (after COVID‐19, ischemic heart disease, and neonatal diseases) [2]. Each year, there are approximately 93.8 million people living with the consequences of stroke worldwide, with 11.9 million new cases and 7.3 million deaths, resulting in 160 million DALYs [2, 24]. Over the past 30 years, the global number of deaths and disabilities from stroke has increased dramatically. The absolute number of new strokes has risen by 70%, and the prevalence has increased by 85%, placing a serious burden on global health systems [25, 26]. Of all stroke cases, 87% are IS, 10% are HS, and 3% are SAH. Taking 2021 as an example, the global prevalence rate stood at 69.93 million cases for IS, 16.61 million cases for HS, and 7.85 million cases for SAH. The age‐standardized prevalence rate witnessed a decline of 4.08% [25]. Among newly diagnosed strokes, IS accounts for 62.4%, HS for 27.9%, and SAH for 9.7% [25]. Approximately 9.5 million cases of IS occur globally each year, leading to around 2.7 million deaths [24, 27, 28]. Therefore, stroke, and particularly IS, represents a major threat to human health and quality of life in modern society.

### 2.2 Regional and Urban–Rural Differences in Stroke

The burden of stroke (incidence, morbidity, mortality, and DALYs) varies significantly across different regions. Historically, stroke was considered a disease of affluent countries. However, with global population aging, improved living standards, urbanization, and a reduction in infectious disease mortality in middle and low‐middle SDI regions, the incidence of cardiovascular disease and stroke has risen rapidly. Stroke has now become the main cause of death and disability in these countries [5, 29, 30]. In 2020, low and middle‐SDI (LMSDI) regions accounted for 86% of global stroke deaths and 89% of global DALYs lost [26].

Currently, the highest burden of stroke is found in East Asia, Central Asia, and sub‐Saharan Africa, while the lowest is in North America, Australasia, and Latin America [2, 31, 32]. Specifically, the age‐standardized prevalence rate of IS is highest in Sub‐Saharan Africa, followed by West Africa within Sub‐Saharan region, East Asia, and Central Asia. The prevalence rate of HS is highest in West Africa within Sub‐Saharan region, Southeast Asia, Oceania, and high‐income Asia‐Pacific regions. The age‐standardized prevalence rate of SAH is highest in high‐income Asia‐Pacific regions and Andean Latin America [25]. Although the age‐standardized incidence and mortality rates of stroke have declined globally over the past 30 years (with the age‐standardized incidence of all strokes decreasing by 17.0% and IS by 10%), the overall decline in LMSDI regions is slower than in high‐SDI regions [24, 26]. Since 1990, the absolute numbers of stroke incidence, mortality, and stroke‐related DALYs have remained essentially unchanged in high‐SDI countries. In contrast, there has been a significant increase in these absolute numbers in middle and LMSDI regions, which are the main contributors to the overall global increase in stroke over the past three decades [26, 33].

Significant disparities in stroke burden are also a result of varying levels of access to, and quality of, stroke management and public health interventions across different regions and countries. Many LMSDI regions lack basic healthcare infrastructure, including trained personnel, diagnostic tools, and treatment facilities. In some areas, stroke units are rare or nonexistent, and thrombolytic drugs are often unavailable [34, 35, 36]. The Middle East and North Africa (MENA) region, for example, has a very high incidence of stroke. The quality of stroke units varies significantly, with great differences in medical services, personnel skills, and training. The number of stroke units in the MENA region is relatively small (only 150), and they are mostly located in high‐income countries or the private sector. Consequently, only a small portion of the population has access, and low‐income individuals in most low‐resource areas have limited access to advanced treatments like mechanical thrombectomy, leading to one of the highest stroke burdens globally [37]. Similarly, the incidence of stroke in sub‐Saharan Africa is two to three times higher than in Western Europe and the United States. With the lowest accessibility to stroke services (only 10 countries have stroke units, and only 30% of hospitals can provide acute stroke care), the burden is high, and the 3‐year mortality rate exceeds 80% [38, 39].

Differences in stroke burden are also pronounced within the same country, particularly between urban and rural areas. Multiple studies have shown that stroke mortality rates are significantly higher in rural areas than in cities. In the United States, stroke mortality is 19% higher in rural areas of the South, 13% higher in the Northeast, 4% higher in the Midwest, and 1% higher in the West, a difference potentially linked to a higher prevalence of stroke risk factors like hypertension and diabetes in rural populations [40, 41].

In China, there are significant geographical differences in stroke burden that have also changed over time. From 1985 to 2013, stroke incidence showed a clear north‐south gradient, with rates of 1097.1, 917.7, and 619.4 per 100,000 people in the northern, central, and southern regions, respectively. The growth rates also varied, increasing by 2.0 times, 1.5 times, and 1.2 times in these regions. Furthermore, the stroke incidence rate in rural China is higher (945.7 vs. 797.5 per 100,000 people) than in urban areas, which may be related to poorer hypertension control and limited access to medical resources in rural regions [42, 43, 44]. In India, the stroke burden also varies significantly between urban and rural areas, with urban incidence rates ranging from 33 to 123 per 100,000 and rural rates at 123.57 per 100,000 [45].

In regions with higher health expenditures and human development index, stroke incidence is lower; however, in regions with higher gross national income (GNI) per capita, stroke risk is increased due to increased risk factors such as diabetes and hypertension [46]. Furthermore, there are significant gender, ethnic, and regional disparities in the quality of stroke care across different regions of India, with rural and underdeveloped areas at a disadvantage in terms of health education and access to telemedicine [47, 48, 49].

### 2.3 Age and Gender Distribution of the Stroke Population

Stroke is generally considered a disease of the elderly, with its incidence and prevalence strongly correlated with age. The average age of onset is between 60 and 70 years. The average age of a first stroke is 61.5 ± 13.4 years for men and 63.4 ± 15.4 years for women, indicating that women typically experience their first stroke 2–3 years later than men [50]. Due to the current trend of global population aging, the overall incidence of stroke is expected to rise significantly in the coming decades [51, 52].

In contrast to the elderly, the incidence of stroke among young adults (aged 15–45 years) is relatively low. Each year, about 2 million young people have a stroke, accounting for 10–15% of the total [53, 54, 55]. The mechanisms of stroke in this population may differ from those in the elderly, including genetic factors and vascular malformations [56, 57]. Many studies have shown that the incidence of stroke among young women is higher than among young men. In the 25–34 and 35–44 age groups, women have more strokes than men (incidence rate ratio: male:female = 0.70 and 0.87) [58, 59]. This may be linked to specific risk factors in young women, who are more prone to nonatherosclerotic strokes, carotid artery dissection, patent foramen ovale, and hormonal factors (e.g., pregnancy, contraceptive use) that increase their risk [58, 59].

Contrary to the decline in stroke incidence among the elderly, the incidence and proportion of stroke among young adults are increasing annually. Globally, the rise in stroke incidence among young adults is up to 40%, particularly among young men, highlighting the need for enhanced stroke management for this group [53, 54, 58].

Thanks to progress in managing stroke risk factors and optimizing prevention strategies, the incidence of stroke among the elderly has shown a downward trend over the past 30 years (IS has an average annual decrease of 1.5% while HS has an average annual decrease of 1.2%) [60, 61, 62, 63, 64]. However, the elderly still bear the main global stroke burden, with people over 50 years old accounting for over 90% of the total [2]. Among this group, men are the dominant demographic between ages 45 and 64 years, with an incidence rate significantly higher than that of women. For example, in the 45–54 and 55–64 years age groups, the male‐to‐female incidence rate ratio is 1.25 and 1.41, respectively [58]. This is related to the fact that middle‐aged men in this age group have a higher prevalence of traditional risk factors such as atherosclerosis, hypertension, smoking, increased BMI, and diabetes [58, 65, 66, 67].

In the age group over 65 years, the incidence rate of postmenopausal women becomes higher than that of men, making them the dominant population (incidence rate ratio of men to women is about 1:1.2) [68, 69]. The neuroprotective effect of estrogen [70] decreases after menopause, leading to reduced vascular adaptability and an increased risk of stroke in older women [51, 71]. Additionally, because women generally live longer, the total number of stroke events in women over 65 exceeds that of men, which is an indirect reason for the higher incidence in women at this stage [72, 73].

Gender‐specific differences are significant in IS, but no such trend is observed in HS or SAH [74]. Overall, the age and gender distribution of stroke presents a “U‐shaped curve,” with young women > young men, middle‐aged men > middle‐aged women, and elderly women > elderly men. These differences are related to various biological, social, and behavioral factors, highlighting the importance of considering age‐ and gender‐specific measures in prevention and intervention strategies [75].

### 2.4 Racial Differences in Stroke

In general, the incidence of stroke is higher in Black individuals than in White individuals. Research indicates that the age‐ and sex‐adjusted incidence rate ratio (IRR) for Black vs. White individuals is 1.51. This disparity is more pronounced in younger populations: the Black/White IRR for individuals aged 45–54 years is 4.02, while the IRR in the group over 85 years is 0.86 [76].

Over the past few decades, while the stroke incidence rate for White individuals has declined, the rate for Black individuals has remained largely unchanged. Although stroke mortality has decreased by about 50% across all races, the mortality rate for Black individuals remains high, and the average age of death from stroke is 2 years younger for Black individuals than for White individuals [77, 78].

The difference in stroke risk between Black and White individuals may be partly attributed to socioeconomic factors. For example, a retrospective study of 34,596 patients (26,640 White [77.0%] and 7956 Black [23.0%]) from 43 hospitals evaluated racial differences in mechanical thrombectomy outcomes. The study found that Black stroke patients received mechanical thrombectomy less frequently than White patients, partly due to longer hospital visit times and fewer opportunities for treatment after a stroke [79]. A follow‐up study involving 126,018 women (11,389 Black and 114,629 White) showed that Black women had a higher overall stroke risk than White women (age‐adjusted HR, 1.47). Although this association was weakened after adjusting for socioeconomic and stroke risk factors, the risk for Black women aged 50–60 years was still significantly higher than for White women [80]. Regarding HS, the incidence of HS in the White population rises with age, whereas no such age‐related difference is observed in the Black population [81]. The increased incidence of HS among Black and Hispanic populations can be partly attributed to the higher prevalence of hypertension in these groups [82].

The stroke risk for Asian and Hispanic populations may fall between that of Black and White populations [83, 84]. However, the underutilization and severity of stroke treatment among Asian individuals may be related to cultural or healthcare system differences [83].

### 2.5 Epidemiology of TIA

TIA refers to an acute onset of temporary neurological dysfunction, typically lasting for less than 1 h. It is usually caused by focal ischemia in the brain, spinal cord, or retina, and is not related to acute tissue infarction [85]. ASA supports the definition of TIA based on tissue (i.e., focal ischemic attack rather than acute infarction) rather than time‐based definition [23]. Although TIA is not a true “stroke” in the strict sense, it acts as an early warning or precursor to stroke, presenting significant short‐term risks for stroke and cardiovascular events. A meta‐analysis reveals that the cumulative stroke risks at 2, 7, 30, and 90‐days post‐TIA are 1.2, 3.4, 5.0, and 7.4%, respectively [86]. In a retrospective study encompassing 14,059 participants and spanning a 66‐year follow‐up period (totaling 366,209 person‐years), 435 individuals suffered from TIA. The estimated incidence rate of TIA stood at 1.19 per 1000 person‐years. Moreover, compared with age‐ and sex‐matched controls, participants who had experienced TIA exhibited a significantly higher risk of stroke (HR = 4.37%) [87].

At present, owing to the transient characteristic of TIA, patients’ limited awareness of its symptoms and severity, and the lack of standardized national monitoring systems, numerous TIAs remain undetected, resulting in reported cases being fewer than the actual occurrences. This issue is further compounded by the inconsistency in epidemiological research criteria and the public and healthcare systems' inability to identify transient focal neurological symptoms linked to TIA. Consequently, accurately estimating the true prevalence of TIA is unfeasible, and population‐based epidemiological studies on TIA are still scarce [17, 88, 89]. In the United States, the number of TIA diagnosed each year is between 200,000 and 500,000 [90]. The 2017 research report from Eastern Finland indicated that the crude incidence rate of TIA was 122 per 100,000 inhabitants, while the crude incidence rate for first‐time TIA was 86 per 100,000 inhabitants. The average age of patients experiencing their first TIA was 70 years, with women averaging 72 years and men averaging 68 years [91].

TIA exhibits a higher incidence and risk among the elderly, males, and Black Americans. A report encompassing 1.3 million residents revealed that the overall incidence rate of TIA was significantly higher in Black individuals compared with White individuals (98.0 vs. 81.3); similarly, males had a markedly higher overall incidence rate of TIA than females (101.4 vs. 69.8) [92]. Gender differences exist in the risk factors for TIA. Males are more prone to the influence of unhealthy lifestyle habits. Compared with females, hypertension and smoking constitute the primary risk factors for TIA in males. Owing to potential biological factors, such as hormonal mechanisms or genetics, females are more susceptible to diabetes or atrial fibrillation (AF), thereby elevating the risk of TIA [93]. Additionally, a strong correlation is observed between advancing age and an increased risk of mortality from TIA. Specifically, for every 10‐year increment in age, the risk of death rises by 79% [94].

## 3 Risk Factors for Stroke

**FIGURE 1:**
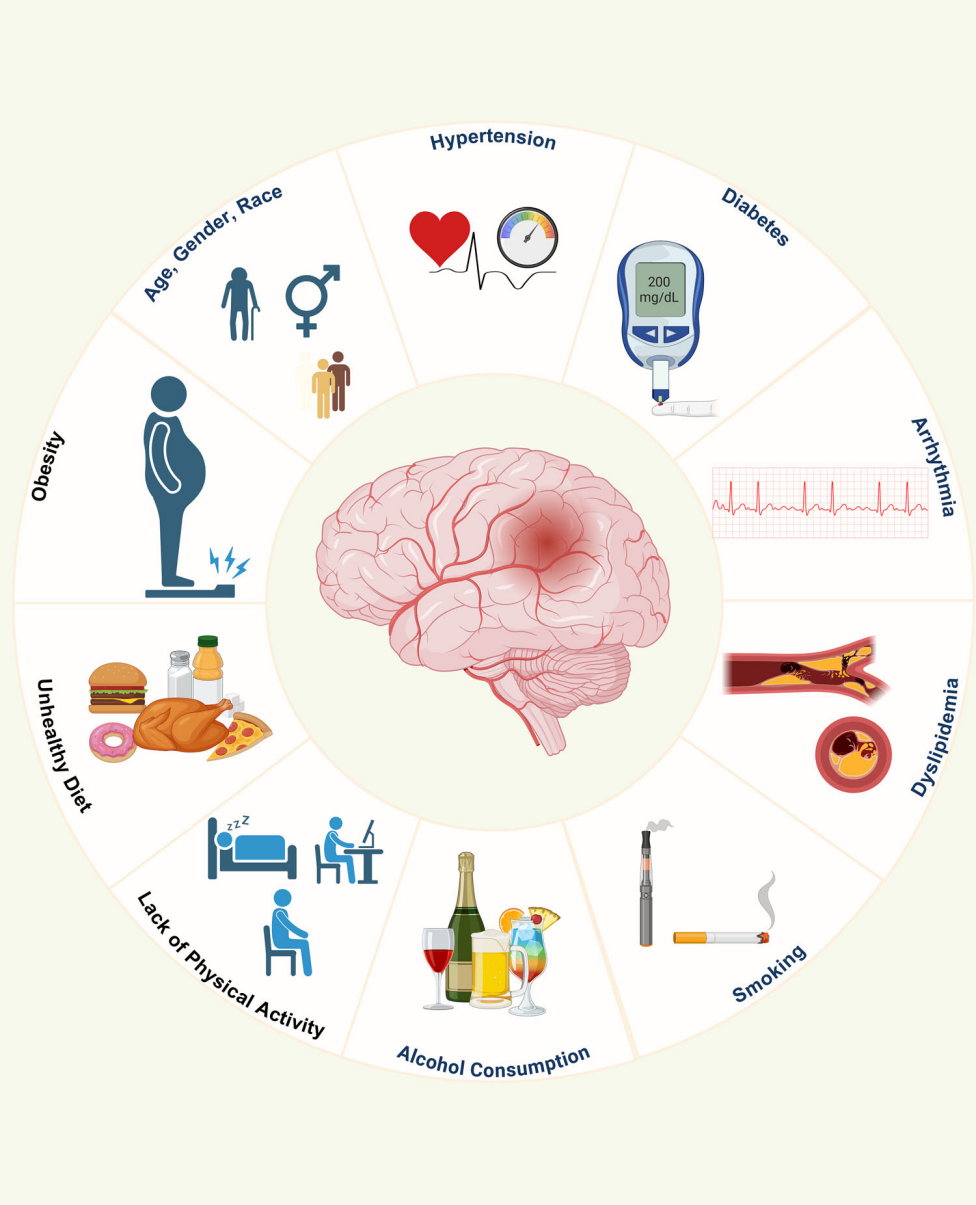
Risk factors contributing to stroke. The risk of stroke is significantly influenced by a combination of nonmodifiable and modifiable factors. Nonmodifiable factors include demographic aspects such as age, gender, and race. Modifiable and lifestyle‐related factors encompass chronic conditions like hypertension, diabetes, dyslipidemia, and arrhythmia, as well as behaviors such as smoking, alcohol consumption, unhealthy diet, lack of physical activity, and obesity.

For effective risk management and the development of prevention strategies, stroke risk factors are categorized as either nonmodifiable or modifiable (Figure 1). Nonmodifiable factors include age, gender, and race. As mentioned earlier, age is a critical independent risk factor, and the risk of stroke increases significantly with age. The risk of stroke also varies among different races, with Black people having a higher risk of stroke and recurrence than White people. Modifiable factors mainly include hypertension, dyslipidemia, smoking, diabetes, heart disease, obesity, alcohol consumption, unhealthy diet, and lack of physical activity. These factors are highly correlated in both middle‐aged and young populations, accounting for over 90% of stroke risk. Managing these factors can effectively reduce both the occurrence and recurrence of stroke [95].

### 3.1 Hypertension

Hypertension is the most common modifiable risk factor for stroke and plays a significant role in both ischemic and HS [96]. The American Heart Association (AHA) defines hypertension as a systolic blood pressure (SBP) of ≥130 mmHg, a diastolic blood pressure (DBP) of ≥80 mmHg, self‐reported use of antihypertensive drugs, or at least two previous reports of hypertension by a physician or other health professional [25].

Hypertension induces widespread changes in intracranial arterial endothelial cells (ECs) and smooth muscle function, increasing the risk of vascular injury and thrombosis through multiple mechanisms [95, 97]. Increased stress on the vascular endothelium can raise BBB permeability, leading to local or multifocal cerebral edema [98, 99]. Endothelial injury and altered blood cell–endothelial interactions can cause local thrombosis and ischemic lesions, leading to lacunar infarction via focal stenosis and occlusion [100]. Degenerative changes in smooth muscle and ECs promote the formation of intracranial microaneurysms, which can lead to hypertensive HS [97, 101]. Furthermore, hypertension accelerates atherosclerosis and increases the likelihood of carotid stenosis, plaque formation, and atherosclerotic heart disease, all of which are closely linked to stroke [102]. In a Mendelian randomization study conducted in China on adults aged 40–79 years, a 10 mmHg rise in genetically predicted SBP was linked to 1.37‐fold increase in the hazard ratio (HR) for IS and a 1.71‐fold increase for HS [103].

Effective management of hypertension through medication and lifestyle adjustments can significantly reduce the burden of stroke across all age groups [104]. One study of 33,357 adults in the ALLHAT trial showed that during a median follow‐up of 4.4 years, 936 patients experienced a stroke. Analysis of their blood pressure revealed that the risk of stroke was lowest in the range of SBP/DBP < 110/<60 mmHg and highest in the range of 170–190/85–100 mmHg [105]. Properly controlling blood pressure can reduce the incidence of stroke by 42% and the risk of HS by 63% [106]. Hypertension is also the most prevalent risk factor for TIA. In the TIA population, the prevalence of hypertension is 58.8% among males and 55.5% among females; in the non‐TIA population, it is 19.7% among males and 22.5% among females [93]. Hypertension also affects stroke prognosis. A study of 430,977 adults in China, aged 30–79 years, with a median follow‐up of 10 years, found 5168 stroke deaths. Compared with those with normal blood pressure, the stroke mortality rates in the prehypertension, isolated systolic hypertension, and compound hypertension groups increased sequentially, with HRs of 1.20, 2.52, and 5.60, respectively [107]. The study revealed that all hypertension subtypes are linked to an elevated risk of cardiovascular and cerebrovascular events. Moreover, blood pressure exerts a more significant influence on HS compared with IS. Among hypertensive patients, the mortality rate from HS is notably higher than that from IS (with HRs of 2.90, 2.86, and 6.91 for HS, vs. 2.04, 2.13, and 3.93 for IS) [107]. Therefore, in clinical practice, identifying and treating hypertension is a critical factor in preventing stroke and improving patient prognosis.

### 3.2 Diabetes

Diabetes is defined by a fasting plasma glucose (FPG) of ≥126 mg/dL, an oral glucose tolerance test (OGTT) 2‐h glucose of ≥200 mg/dL, a random glucose of ≥200 mg/dL with hyperglycemia symptoms, or an HbA1c of ≥6.5%. Prediabetes, also known as impaired glucose tolerance (IGT) or impaired fasting glucose, increases the risk of developing diabetes and is defined by an FPG of 100–125 mg/dL, a 2‐h OGTT glucose of 140–199 mg/dL, or an HbA1c of 5.7–6.4% [108].

Both diabetes and IGT are significant risk factors for stroke. IGT accounts for 20% of the population attributable risk (PAR) of stroke, while diabetes contributes to 22.6% of the PAR [95, 109, 110]. A cohort study encompassing 47,720 individuals with type 1 diabetes and 686,158 individuals with type 2 diabetes demonstrated that, compared with control subjects, patients with type 1 diabetes had a 2.54‐fold higher risk of IS and a 1.88‐fold higher risk of HS. Among patients with type 2 diabetes, the risk of IS was 1.37 times higher, whereas no significant increase was observed in the risk of HS, except for a slight elevation when HbA1c levels exceeded 72 mmol/mol [111]. Diabetes also increases the risk of TIA. Studies show that the prevalence of diabetes in the TIA group is 11.15% for males and 13.2% for females, while in the non‐TIA group, it is only 4.0% for males and 5.3% for females [93].

The risk of stroke in diabetes patients has doubled, and about 20% of the death causes in diabetes patients are caused by stroke [112]. Diabetes increases stroke risk by mediating vascular endothelial dysfunction, promoting atherosclerosis, inducing systemic inflammation, and causing blood hypercoagulability. In patients with diabetes, due to the increased inactivation of nitric oxide (NO) or the decreased responsiveness of smooth muscle to NO, the NO‐mediated vasodilation function is damaged, causing endothelial damage and dysfunction and triggering the cascade reaction of atherosclerosis, making arteries stiff and elastic [113, 114]. The inflammatory response is enhanced in diabetic patients, especially serum markers related to cardiovascular risk such as CRP, IL‐1, IL‐6, TNF‐α, and adiponectin. This inflammation further promotes the development of atherosclerotic plaque, increasing stroke risk [115]. Diabetes causes blood acquired hypercoagulability and promotes thrombosis, which is an important reason for incidence rate and mortality of IS [116, 117]. A meta‐analysis of 27 studies and 274,631 patients with a history of IS showed that diabetes was associated with a significant risk of stroke recurrence. Controlling blood glucose can help reduce the stroke burden. A meta‐analysis of 19 randomized controlled trials (RCTs) (155,027 patients with type 2 diabetes) found that compared with a placebo, glucagon‐like peptide‐1 (GLP‐1) agonists reduced nonfatal stroke by 15% and stroke incidence by 16% [118].

### 3.3 Arrhythmia

Cardioembolic stroke accounts for 20–25% of all ISs, with AF being the most common underlying cause. Stroke and TIA attacks are often the first clinical manifestation of AF [106]. AF affects nearly 3 million people in the United States and 4.5 million in Europe [119]. It is the most common arrhythmia, increasing the risk of IS by three to five times and accounting for an estimated 15% of all strokes worldwide [119, 120]. A meta‐analysis of 50 studies found that approximately 24% of IS patients had AF [121]. In patients with TIA, the prevalence of AF is 14.7% for males and 16.4% for females, whereas in patients without TIA, the prevalence is 2.5% for males and 3.6% for females [93]. Because AF is often asymptomatic and goes undetected, the true risk of stroke attributable to AF may be severely underestimated [25].

AF leads to atrial blood stasis and the formation of thrombi in the left atrial appendage, causing cardioembolic stroke [122, 123]. AF also causes atrioventricular asynchrony, resulting in reduced cardiac output, decreased blood pressure, and diminished cerebral blood flow (CBF) [124, 125, 126]. Furthermore, AF is a proinflammatory state where inflammation enhances blood hypercoagulability, promotes thrombosis, and impairs cerebral vascular regulation, all of which increase stroke risk [127, 128, 129].

In addition to promoting stroke occurrence, AF patients experience more severe neurological deficits, higher recurrence rates, and higher rates of disability and mortality from stroke [130]. A meta‐analysis of 23,054 patients with AF and a history of stroke found that persistent/permanent AF had a higher risk of stroke recurrence compared with paroxysmal AF. A stroke database containing 4079 stroke patients showed that 260 (6.4%) had AF, of which 106 (2.6%) were newly diagnosed. Compared with non‐AF stroke patients, those with AF had significantly higher NIHSS scores (7.9 vs. 5.9), a higher modified Rankin score (mRS) (*p* < 0.001), and a significantly higher mortality rate at 90‐day follow‐up (*p* = 0.002) [131]. In a clinical study of 1121 patients with IS, 17.8% had AF. Compared with non‐AF stroke patients, those with AF‐related stroke had a higher mortality rate (median survival 531 vs. 1808 days) and were more prone to severe disability [132]. Oral anticoagulants (e.g., rivaroxaban) can reduce stroke risk by about 65% in AF patients, but the risk of bleeding must be carefully balanced [133, 134, 135].

### 3.4 Dyslipidemia

Cholesterol is a primary pathogenic risk factor for atherosclerosis and cardiovascular disease. The relationship between dyslipidemia (high low‐density lipoprotein cholesterol [LDL‐C], low high‐density lipoprotein cholesterol [HDL‐C], high triglycerides [TGs]) and stroke risk is complex. On the one hand, dyslipidemia increases the risk of IS by promoting atherosclerosis and vascular damage, accounting for 48.2% of the attributable risk (PAR) among stroke patients [110, 117, 136]. On the other hand, a meta‐analysis of 23 prospective cohort and case–control studies showed that a 1‐mmol/L increase in TC concentration was associated with a 15% reduction in the risk of HS [137].

LDL‐C is the factor most closely associated with IS risk and is the preferred target for lifestyle and drug therapy. Multiple studies from RCTs, Mendelian randomization analyses, and population‐based cohorts have proven a direct causal relationship between serum LDL‐C and the risk of atherosclerotic IS. The Mendelian randomization study showed that for every 1 mmol/L decrease in LDL‐C predicted by genes, the risk of IS decreased by 25%, but the risk of HS increased by 13% [138]. Elevated LDL‐C levels increase the risk of both stroke occurrence and poor prognosis [139, 140]. A meta‐analysis of 688,376 participants from nine cohort studies found that a decrease of 1 mmol/L in LDL‐C levels resulted in a 15% reduction in IS risk [141]. In an RCT that included patients with a history of IS/TIA and significant atherosclerosis, those with low LDL‐C (<70 mg/dL) had a lower risk of subsequent cardiovascular events (HR, 0.78) [142]. A meta‐analysis of LDL‐C‐lowering drug trials showed that for every 1 mmol/L decrease in LDL‐C, the risk of IS decreased by 20%, but the risk of cerebral hemorrhage increased by 17% [138].

HDL‐C has a protective effect against atherosclerosis and IS, with HDL‐C levels being negatively correlated with stroke risk [143]. A meta‐analysis involving 62 prospective cohort studies (900,501 participants and 25,678 stroke patients) showed that for every 1 mmol/L increase in HDL‐C levels, the overall risk of stroke decreased by 18% (relative risk [RR], 0.82), with an RR of 0.75 for IS and an RR of 1.21 for cerebral hemorrhage [138].

The relationship between abnormal TGs and stroke risk is complex and depends on the stroke subtype [140]. Elevated TG levels are a risk factor for IS. A cohort study of 5,688,055 young adults (20–39 years) with a median follow‐up of 7.1 years found that higher serum TG concentrations were associated with an increased risk of stroke (HR, 2.53) [144]. Conversely, low TG levels are associated with an increased risk of HS. A prospective cohort study of 27,937 patients found that those in the lowest quartile for TG levels had a higher risk of HS [145].

Overall, early diagnosis and the use of statins to treat dyslipidemia are crucial for reducing the risk of IS and other ischemic cardiovascular events, which is particularly important for long‐term prognosis [146]. Research has shown that in patients with minor IS or TIA, elevated untreated LDL‐C levels are linked to a heightened short‐term risk of IS. Lipid‐lowering therapy may prove beneficial for those with minor stroke or TIA and LDL‐C levels of 2.6 mmol/L or higher [147]. However, certain studies have revealed that statin therapy can elevate the risk of cerebral hemorrhage [148]. In some stroke patients with a history of bleeding, small vessel disease, or cerebral amyloid angiopathy, statins have been significantly linked to an increased risk of cerebral hemorrhage [149]. These studies have further complicated the relationship between dyslipidemia and cerebral hemorrhage.

### 3.5 Smoking

Behavioral factors, including smoking, alcohol consumption, low physical activity, and an unhealthy diet, collectively account for 74.2% of the stroke risk burden [95]. Among these, smoking is a core component and has a significant impact on stroke risk, contributing to approximately 15% of all stroke deaths annually [150, 151]. Currently, there are over 1 billion adult smokers and 700 million children who are passive smokers worldwide. Smoking rates are particularly high among young people, with the proportion and intensity of smoking increasing sharply after the age of 18 years [152].

A Mendelian randomization study, based on genetic data from 370,000 individuals, showed a causal association between smoking behavior (characterized by 372 SNPs) and IS, independent of other factors like obesity and education level [150]. A case–control study involving 13,462 acute stroke cases and 13,488 controls found that smoking is associated with an increased risk of all strokes, and the association with IS (OR 1.85) is stronger than that with cerebral hemorrhage (OR 1.19) [153]. The risk of IS is dose‐dependent on daily smoking volume. A meta‐analysis of 141 cohort studies found that low smoking volume (≈1 cigarette/day) increased stroke risk by 50% compared with high volume (≈20 cigarettes/day) [154]. Smoking is also a significant risk factor for TIA, particularly among male patients. Research has revealed that 41.3% of male TIA patients smoke, in contrast to only 4.2% of female patients [93]. Persistent smoking after experiencing a TIA doubles the risk of recurrent cerebrovascular events and elevates mortality rates. Studies indicate that patients who continue smoking post‐TIA face a stroke risk as high as 17.8% within 90 days, with nearly half of these strokes occurring within the first 2 days after the TIA [17]. Furthermore, the FINRISK study, encompassing 65,521 individuals, demonstrated a strong correlation between smoking and the risk of SAH (HR, 2.77). This risk exhibits a dose–response relationship and cumulative effect, peaking among heavy smokers [155].

Numerous studies show that smoking has detrimental effects on the cardiovascular system and influences the occurrence and prognosis of stroke by promoting the initiation and progression of atherosclerosis, inducing inflammation and oxidative stress, and increasing coagulation risk [146, 156]. Cigarette smoke reduces NO production by endothelial NO synthase (eNOS) through the release of free radicals and reactive oxygen species (ROS). This impairs blood flow‐mediated vasodilation, promotes vascular smooth muscle cell (VSMC) proliferation, and releases matrix metalloproteinases (MMPs), thereby initiating atherosclerosis [157]. Nicotine in smoke increases VSMC proliferation by stimulating the release of fibroblast growth factor (FGF) and promotes VSMCs to degrade the extracellular matrix (ECM) and reshape the cytoskeleton via nAChR [158]. VSMCs that migrate to the intima release platelet‐derived growth factor (PDGF), acquire a myofibroblast/osteoblast phenotype, and transform into foam cells [159]. Finally, nicotine activates α7 homomer nAChR in ECs, leading to cholinergic‐induced neovascularization and plaque formation [160].

Smoking exacerbates neuroinflammation and oxidative stress by activating NF‐κB [161, 162], increasing proinflammatory cytokines (TNF‐α, IL‐6) [163, 164], and upregulating adhesion molecules (intercellular adhesion molecule‐1 [ICAM‐1], E‐selectin, P‐selectin) [165]. It generates ROS through NAD(P)H oxidase and xanthine oxidase [162, 166] and affects stroke progression by downregulating eNOS and upregulating inducible NOS (iNOS) [167, 168]. In addition, cigarettes can promote endothelial dysfunction, mediate the imbalance between coagulation factors and anticoagulant factors, keep the blood in a procoagulant state, and promote thrombus [169, 170, 171].

As a crucial modifiable risk factor, smoking cessation is vital for both primary and secondary stroke prevention. Quitting can rapidly reduce stroke risk; after 2–4 years, the smoking‐related risk virtually disappears [172, 173]. Quitting after a stroke significantly improves a patient's prognosis, reducing the risk of recurrence and mortality. Cross‐sectional studies show that the 5‐year risk of recurrent stroke, myocardial infarction, or death is 15.7% for patients who quit smoking after a stroke, compared with 22.6% for those who continue to smoke [174].

### 3.6 Alcohol Consumption

Alcohol consumption is a significant modifiable risk factor for stroke in young adults, increasing stroke risk through mechanisms such as hypertension, blood pressure fluctuations, and metabolic disorders [175]. The relationship between alcohol consumption and stroke risk depends on the type of stroke. For IS, a J‐shaped relationship exists: light to moderate consumption (up to 2 drinks/day for men and 1 drink/day for women) has a protective effect, while heavy consumption is associated with an increased risk [146, 176].

In contrast, there is a more direct linear relationship between alcohol consumption and HS. A study of 1,536,668 patients (mean age 29.5 years, 71.5% male) with a median follow‐up of 6 years found that for those with a cumulative alcohol consumption exceeding 105 g/week for 2, 3, and 4 years, the risk of stroke (primarily HS) was significantly higher than for patients with a lower consumption score (HR 1.19, 1.22, 1.23) [177]. In high‐risk populations, such as Asian communities, managing alcohol intake is increasingly being emphasized as a preventive strategy for young people [177].

### 3.7 Lack of Physical Activity

A sedentary lifestyle and lack of physical activity can lead to obesity and hypertension, increasing the risk of stroke. For every 1‐h increase in sedentary time per day, the risk of stroke increases by 14% [178]. A prospective study involving 437,318 participants found that insufficient physical activity is associated with an increased risk of total stroke (HR 1.52), IS (HR 1.49), and HS (HR 1.83) [178]. In the TIA population, 27.2% of men and 28.0% of women were insufficiently physically active, compared with only 13.6% of men and 16.7% of women in the non‐TIA population [93].

A longitudinal cohort study of 3,472 stroke survivors found that a lack of physical activity before the stroke was associated with an increased risk of developing dependence on activities of daily living 3 months after the stroke [179]. Physical activity can reduce stroke risk and improve prognosis by controlling hypertension, reducing the incidence of diabetes, and managing weight [180]. Research has found that habitual exercise and enhanced cardiovascular fitness are linked to higher cognitive function, increased CBF, and a mitigation of the decline in gray matter volume and white matter integrity [181, 182]. A cross‐sectional study of 102,578 individuals (3851 stroke patients) showed that different types, frequencies, and intensities of physical activity can reduce stroke risk, including recent moderate‐intensity activity, high‐intensity activity (OR, 0.6), and muscle‐strengthening training [183].

### 3.8 Unhealthy Diet

An unhealthy diet increases stroke risk through its direct effects and its contribution to other risk factors like hypertension, diabetes, and dyslipidemia [184]. Insufficient vegetable intake can promote hypertension, worsen vascular damage, and increase stroke risk. A case–control study found that among stroke patients, low vegetable intake and hypertension were prevalent at 68.1 and 95.9%, respectively. The combined risk of low vegetable intake and hypertension was extremely high [185]. The dietary fiber and antioxidants (e.g., vitamin C, magnesium) in vegetables have neuroprotective effects and can reduce stroke risk [186]. Consuming vegetables can lower stroke risk by 8% [187]. For every 200 g/day increase in the combined intake of fruits and vegetables, the risk of IS is reduced by 13% [188]. A meta‐analysis encompassing six cohort studies revealed that a high daily consumption of green leafy vegetables could decrease the overall stroke risk by 7%, the IS risk by 8%, and the HS risk by 5% [189]. A Bayesian meta‐regression analysis of 12 cohort studies indicated that elevating vegetable intake to 306–372 g per day could reduce the IS risk by 23.2% and the HS risk by 15.9% [190].

Animal fat is another key factor in increasing stroke risk. A 12% increase in stroke risk is linked to increased red meat intake, rising to 17% for processed red meat [187]. A high‐sugar diet can increase inflammatory markers, oxidative stress, and endothelial dysfunction, elevating stroke risk [191]. Research shows that high fructose intake (e.g., from sugary drinks) promotes visceral fat accumulation and insulin resistance, contributing to hyperlipidemia and diabetes, thereby creating a hidden danger for stroke. Conversely, limiting caloric fructose intake can reduce liver fat and improve insulin dynamics [192].

Even artificial sweeteners in “zero‐sugar” beverages can promote stroke. A study of 2888 stroke patients over age 45 years found that those who consumed ≥1 artificially sweetened soft drink daily (e.g., zero‐sugar cola) had a 1.97‐fold and 2.34‐fold increased risk of total and IS, respectively, compared with those who did not [193]. A high‐salt diet and hypertension are also associated with an increased stroke risk. An increase of 100 mmol in sodium intake is linked to a 32% increase in stroke incidence and an 89% increase in stroke mortality [194].

### 3.9 Obesity

Obesity is a key metabolic risk factor associated with hypertension, diabetes, and dyslipidemia, all of which increase stroke risk [195, 196]. Mendelian randomization studies have shown a significant correlation between a high BMI and an increased risk of IS [150]. A meta‐analysis of 97 cohort studies and 1.8 million participants found that 76% of the impact of BMI on stroke risk was mediated by blood pressure, cholesterol, and glucose levels. Blood pressure alone accounts for 65% of the risk caused by excess weight. For every 5 kg/m^2^ increase in BMI, stroke risk rises by 18% [197]. The link between obesity and TIA/IS may stem from increased fat deposition around the carotid arteries, which impairs vascular health and works synergistically with hypertension and diabetes to contribute to metabolic syndrome, thereby elevating the risk of TIA/IS [93, 198].

Current research suggests that increased abdominal obesity, measured by waist‐to‐hip ratio, is a greater contributor to stroke risk than overall weight gain measured by BMI [146, 199]. A retrospective study found that compared with individuals with a normal waist circumference and BMI, those who were overweight but without abdominal obesity, those with only abdominal obesity, and those who were both overweight and had abdominal obesity had a higher stroke risk, with HRs of 1.09, 1.63, and 1.40, respectively. Additionally, compared with overweight individuals, those with abdominal obesity had a significantly higher risk of IS [200].

## 4 Pathophysiological Processes and Related Signaling Pathways After Stroke

The pathophysiological process following stroke involves complex mechanisms of cell damage and repair, which vary depending on the stroke subtype. This section elaborates on the pathophysiological processes and related signaling pathways after stroke in various subtypes, providing potential targets for clinical treatment. Following IS, reduced blood flow to the brain leads to a lack of cellular energy, triggering a series of damage cascades, including excitotoxicity, ion homeostasis imbalance, calcium overload, free radical toxicity, inflammatory response, neuronal necrosis, and blood–brain barrier (BBB) disruption. Brain injury following HS is mainly due to primary damage caused by the mass effect of hematoma and secondary damage caused by hematoma's toxic components. TIA is a temporary neurological deficit in which energy metabolism has not completely collapsed and almost no tissue damage is involved.

### 4.1 Pathophysiological Processes and Related Signaling Pathways After IS

Following an IS, the interruption of blood flow leads to a lack of oxygen and nutrients in the affected area. This triggers a cascade of damaging events, including ion homeostasis imbalance (excitotoxicity, calcium overload, acidosis), oxidative stress, inflammatory response activation, programmed cell death (PCD), BBB disruption, and cerebral edema. These pathological processes are interconnected and ultimately lead to ischemic necrosis [8]. During the recovery period, the body promotes the restoration of behavioral function through self‐repair mechanisms such as the reparative effects of neuroinflammation, neural plasticity, angiogenesis, and neural network regeneration [201, 202, 203, 204].

#### 4.1.1 Energy Deficiency, Excitotoxicity, and Ion Homeostasis Imbalance

ATP is essential for the function of energy‐dependent ion pumps like Na⁺/K⁺‐ATPase [205]. After an IS, insufficient blood supply in the ischemic region leads to decreased ATP production. This renders energy‐dependent Na⁺/K⁺‐ATPase ineffective, causing a large influx of Na^+^ and Ca^2+^ and an efflux of K^+^. This imbalance results in cellular edema and depolarization of the membrane potential [206, 207, 208].

Glutamate is the brain's primary excitatory neurotransmitter. After a stroke, reduced CBF impairs ion transporters, such as the glial glutamate transporters GLT‐1 and GLAST [209], leading to abnormal glutamate release and reuptake. This accumulation of glutamate in the extracellular space causes overactivation of N‐methyl‐d‐aspartate receptors (NMDARs), particularly the GluN2B subunits. By enhancing NMDAR‐mediated excitatory postsynaptic currents, this process promotes excitotoxicity, which triggers downstream cascade reactions that ultimately lead to neuronal dysfunction [210, 211, 212, 213].

**FIGURE 2:**
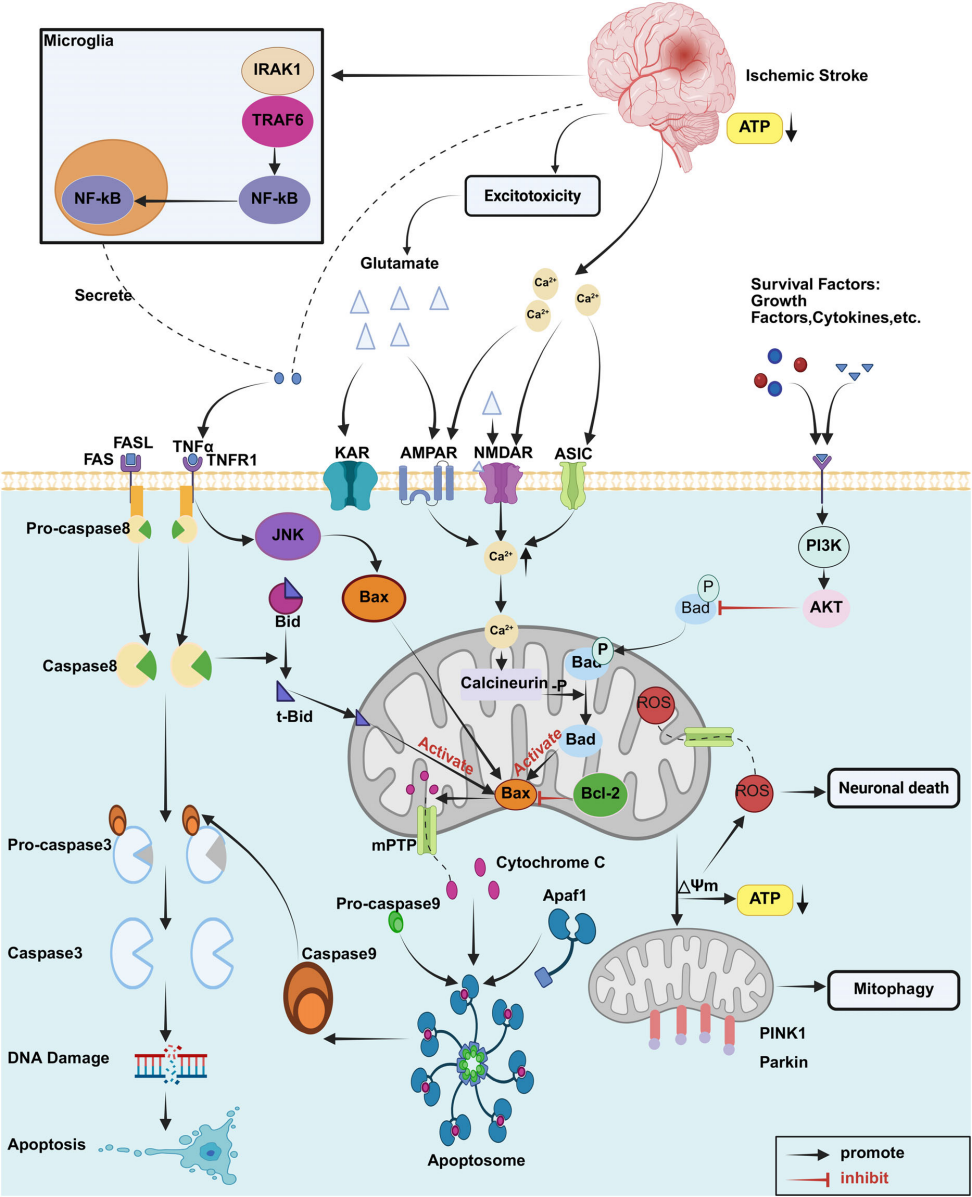
Schematic diagram of IS pathophysiology focusing on excitotoxicity, mitochondrial dysfunction, and neuronal apoptosis. Following IS, neuronal death is triggered by coordinated excitotoxic and inflammatory pathways. Excessive glutamate release activates ionotropic receptors (NMDAR, AMPAR, KAR, and ASIC), leading to a massive Ca^2+^ influx that results in mitochondrial Ca^2+^ overload and the generation of ROS. This accumulation of ROS and Ca^2+^ damages mitochondria and opens the MPTP, which facilitates the release of cytochrome *c*. In the endogenous pathway, released cytochrome *c* leads to DNA damage and apoptosis through a series of pathways. The exogenous pathway is mediated by inflammatory factors. Microglia contribute to the inflammatory environment. Ischemia induces mitochondrial membrane potential depolarization (ΔΨm). Depolarized mitochondria lead to PINK1 accumulation. PINK1 selectively recruits Parkin and triggers mitophagy. *Abbreviations*: AKT, protein kinase b (PKB); AMPAR, α‐amino‐3‐hydroxy‐5‐methyl‐4‐isoxazolepropionic acid receptor; Apaf1, apoptotic protease activating factor‐1; ASIC, acid‐sensing ion channel; ATP, adenosine triphosphate; Bad, Bcl‐2‐associated death promoter; BAX, Bcl‐2‐associated x protein; BCL, B‐cell lymphoma; BID, BH3‐interaction domain death agonist; FAS, Fas cell surface death receptor; FASL, Fas ligand; JNK, c‐Jun n‐terminal kinase; KAR, kainate receptor; MPTP, mitochondrial permeability transition pore; NMDAR, n‐methyl‐d‐aspartate receptor; PI3K, phosphoinositide 3‐kinase; PINK, PTEN‐induced putative kinase 1; TNF, tumor necrosis factor; TNFR, tumor necrosis factor receptor.

NMDARs serve as a major signaling hub, and many neuronal death signaling proteins directly bind to their cytoplasmic tails, inducing neuronal death (Figure 2) [214, 215]. As a ligand‐gated ion channel for Ca^2+^, NMDAR overactivation by glutamate stimulates intracellular calcium overload, which in turn leads to mitochondrial calcium overload and depolarization, triggering cell death [216].

After a stroke, glutamate activates NMDARs, allowing Ca^2+^ to flow into cells. This influx induces calcineurin‐mediated dephosphorylation and activation of DAPK1 at serine‐308. Activated DAPK1 then binds to the C‐terminal domain of the NMDAR GluN2B subunit, enhancing receptor activity to promote excitotoxicity. DAPK1 can also be phosphorylated by extracellular signal‐regulated kinase (ERK) at serine‐735, directly inducing neuronal death by enhancing DAPK1 activity and inhibiting ERK's prosurvival function [217].

Furthermore, glutamate‐induced NMDAR activation triggers calcium‐dependent calpain‐mediated hydrolysis of the p35 protein, which is cleaved into a smaller 25 kDa form (p25). This induces neuronal death by causing abnormal activation and targeted redirection of cyclin‐dependent kinase 5 [218]. PTEN also directly binds to the C‐terminal domain of the NMDAR GluN1 subunit to enhance excitotoxicity, and it translocates to the nucleus in a GluN2B‐dependent manner to induce neuronal death [215].

In addition to NMDARs, glutamate can activate CP‐AMPARs, leading to Na⁺ and Ca^2+^ influx. This causes cellular edema, membrane depolarization, and an imbalance of intracellular ion homeostasis, ultimately affecting neuronal survival [208].

Calcium overload is a core event of excitotoxicity. After a stroke, Ca^2+^ enters cells through voltage‐dependent and ligand‐gated ion channels, leading to an ion imbalance and the activation of downstream signaling pathways [8]. Insufficient ATP can inhibit ATP‐sensitive K⁺ channels, causing membrane depolarization and activating voltage‐gated l‐type Ca^2+^ channels [219, 220]. The high calcium permeability of overactivated NMDARs and CP‐AMPARs leads to a large influx of calcium, causing mitochondrial dysfunction, ROS generation, and endoplasmic reticulum (ER) stress [221, 222, 223]. The ER, as a calcium reservoir, responds to an increase in Ca^2+^ concentration by activating the unfolded protein response pathway [219]. During the ER stress response, ATF6 and other transcription factors activate the ER Ca^2+^ pump, which releases stored calcium and further increases intracellular Ca^2+^ concentration [224].

Calcium overload activates calmodulin (CaM)‐dependent kinase II (CaMKII), which promotes DRP1‐mediated mitochondrial fission. This leads to mitochondrial dynamics disorders, and the interaction between DRP1 and BAX promotes neuronal apoptosis [225]. The ER–mitochondria contact site transmits calcium signals through the IP3R–VDAC1–MCU pathway, resulting in mitochondrial calcium overload [226]. Mitochondrial calcium overload promotes ROS generation and mitochondrial damage, impairing mitochondrial ATP production and forming a vicious cycle of “energy depletion–ion imbalance–secondary damage,” further exacerbating brain damage [227, 228].

#### 4.1.2 Oxidative Stress

Oxidative stress is a key event in the cascade of cerebral ischemic–reperfusion injury [229]. Hypoxia and reperfusion lead to the production of a large amount of ROS and reactive nitrogen species (RNS), which cause destructive effects through various mechanisms [229]. ROS are highly reactive oxygen‐containing chemical substances, including singlet oxygen, superoxide anions (O^2−^), hydrogen peroxide (H_2_O_2_), and hydroxyl radicals (⋅OH) [230]. After an IS, the excessive production and insufficient clearance of ROS lead to their accumulation in cells, creating a state of “oxidative stress” [231, 232]. Mitochondria generate superoxide anion radicals during electron transfer and, after a stroke, mitochondrial dysfunction leads to a large amount of ROS production, making them the primary source of ROS [232]. Additionally, AA metabolism via cyclooxygenase and lipoxygenase (LOX) pathways can also produce some superoxide [233]. After ischemic–reperfusion, activated microglia and peripheral infiltrating immune cells further generate oxygen free radicals by activating the Nox2/gp91phox system containing NADPH oxidase, which exacerbates oxidative stress [234, 235].

ROS can react with DNA, proteins, and lipids, causing varying degrees of damage and functional impairment (Figure 2) [236]. Ischemia induces mitochondrial membrane potential depolarization (ΔΨm). Depolarized mitochondria lead to excessive ROS production, decreased ATP production, and PTEN‐induced putative kinase 1 (PINK1) accumulation. PINK1 selectively recruits Parkin and triggers mitophagy [227]. Furthermore, elevated ROS levels and calcium overload open the membrane permeability transition pore (MPTP), releasing ROS, cytochrome *c* (Cyt*c*), and other substances, ultimately leading to neuronal death [227].

After ROS is released by mitochondria, Cyt*c* binds with apoptotic protein activator protein‐1 (Apaf‐1) and deoxyadenosine triphosphate to form apoptotic bodies, activate caspase‐9, and activate downstream caspase‐3. Caspase‐3 can cleave nuclear DNA repair enzymes, leading to increased oxidative DNA damage in the brain [237]. After ROS activates caspases, caspase‐activated DNAse is activated, which can cleave DNA and lead to cell apoptosis [238]. ROS can also activate p38 and JNK in the MAPK family, thereby promoting the release of inflammatory mediators in the NF‐κ B pathway, promoting inflammation and apoptosis [239]. Bad is a proapoptotic protein that belongs to the Bcl‐2 family. When Bad binds to Bcl‐2, it inhibits the antiapoptotic effect of Bcl‐2 and promotes cell death. ROS activates JNK, which phosphorylates Bad and induces its translocation from the cytoplasm to the mitochondria, where it binds to other mitochondrial membrane proteins (e.g., Bcl‐2, Bcl‐xL), further transmitting apoptotic signals and promoting neuronal damage [240].

RNS are nitrogen‐containing compounds formed by NO and redox reactions. They exhibit strong reactivity [241]. NO is an important free radical involved in poststroke brain injury, produced from l‐arginine by NOS enzymes. Neuronal NOS (nNOS), which requires Ca^2+^/CaM for activation, and iNOS, expressed by inflammatory cells, are both damaging subtypes [242]. After a stroke, NMDARs induce nNOS‐mediated NO production in a Ca^2+^‐dependent manner, contributing to excitotoxic injury [243, 244]. The excessive NO produced by nNOS and iNOS diffuses freely and reacts with superoxide to form peroxynitrite (ONOO−), leading to lipid peroxidation, mitochondrial dysfunction, and cell apoptosis, all of which exacerbate ischemic–reperfusion injury [245, 246, 247]. iNOS is activated by inflammatory cells, and the Dectin‐1/Syk pathway promotes iNOS‐mediated NO release and enhances TNF‐α expression, further worsening nerve damage [248, 249].

eNOS, expressed in ECs, plays a protective role by maintaining vascular homeostasis, promoting vasodilation, and improving CBF [250]. However, after ischemia, eNOS activity is inhibited, and NO bioavailability is reduced, leading to vascular constriction, platelet activation, and an intensified inflammatory response [251, 252, 253].

Both ROS and RNS activate several cell death pathways, such as apoptosis and inflammation. This interaction forms a vicious cycle, as apoptosis and inflammation further stimulate the production of ROS and RNS, playing a critical role in the destructive process following an IS [254].

#### 4.1.3 The Destructive Effects of Neuroinflammation

After an IS, dying cells release molecules such as ATP, high mobility group protein 1 (HMGB1), uridine 5'‐triphosphate, and heat shock proteins. These danger‐associated molecular patterns (DAMPs) activate pattern recognition receptors (PRRs) on the surface of resident brain cells (e.g., ECs, astrocytes, microglia, neurons), including the toll‐like receptor (TLR) family (TLR1–13) and suppression of tumorigenicity 2. This activation triggers downstream purinergic receptors and intracellular inflammasome families, leading to the release of NF‐κB, ROS, MMPs, EC adhesion molecules, and proinflammatory cytokines (IL‐1β, IL‐6, IL‐17, IL‐18, TNF‐α). This cascade results in aseptic inflammation, oxidative stress, neuronal apoptosis, and BBB disruption [255, 256, 257].

**FIGURE 3:**
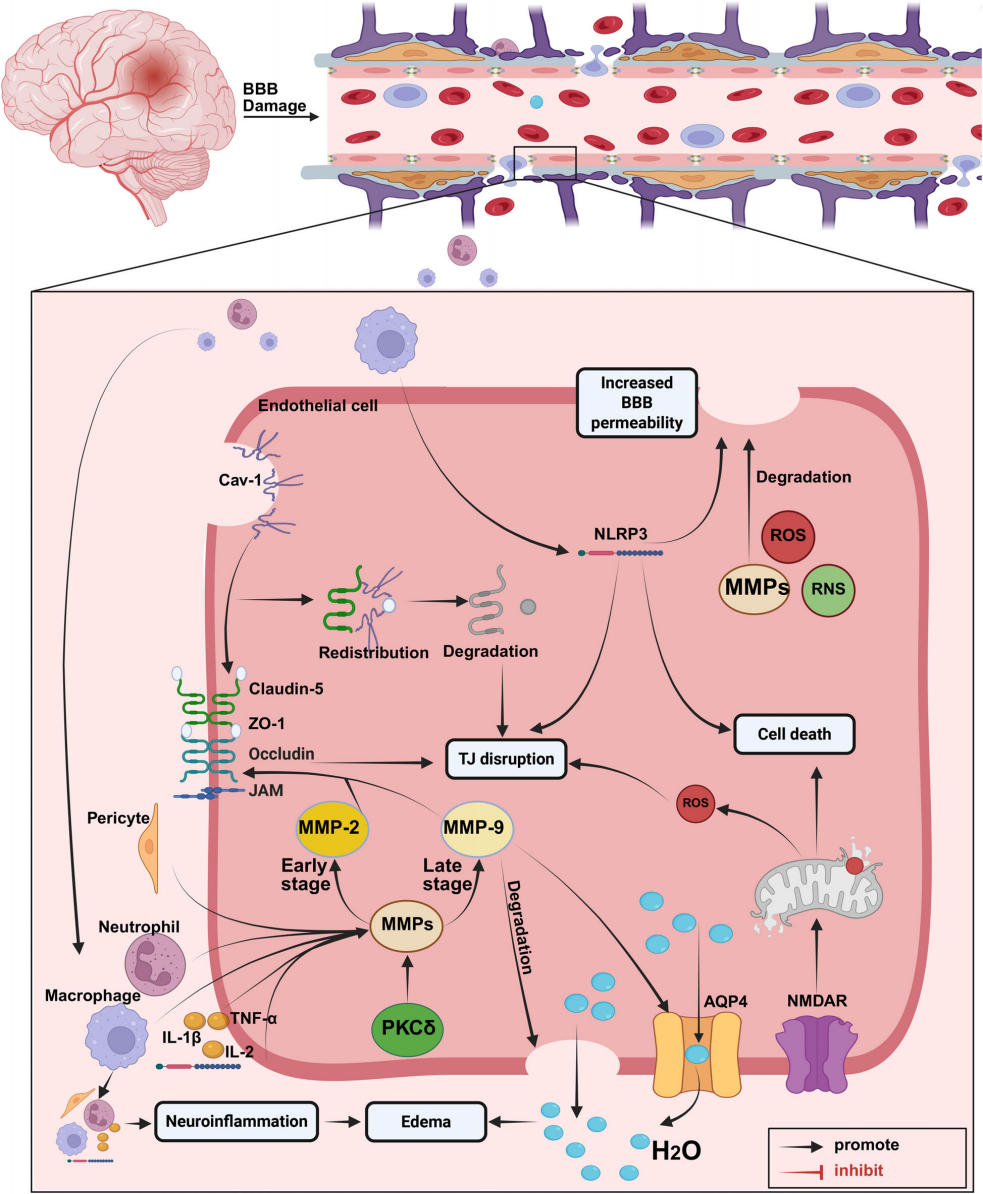
Schematic diagram of blood–brain barrier disruption mechanisms following IS. Following IS, damaged local tissues recruit immune cells, and BBB disruption allows the infiltration of peripheral immune cells, secretion of proinflammatory factors, promotion of TJ destruction, and drive neuroinflammation and cerebral edema. MMPs are mainly produced by peripheral immune cells and pericytes. Their overactivation is the main cause of TJ degradation. In the early stage, it is mainly mediated by MMP‐2, and in the later stage, it is mainly mediated by MMP‐9. In addition, PKCδ, NLRP3 inflammasomes, ROS, RNS, and Cav‐1 also participate in the further destruction and degradation of TJ. These factors also increase BBB permeability and exacerbate cerebral edema by participating in mediating endothelial cell apoptosis, damaging endothelial cell membranes, and activating AQP4. *Abbreviations*: AQP4, aquaporin‐4; BBB, blood–brain barrier; CAV‐1, caveolin‐1; IL, interleukin; JAM, junction adhesion molecule; MMP, matrix metalloproteinases; NLRP3, NOD‐like receptor family pyrin domain containing 3; NMDAR, n‐methyl‐d‐aspartate receptor; PKC, Ca^2+^‐dependent protein kinase C.

Microglia, the resident immune cells of the brain, are the first and most critical immune responders in poststroke neuroinflammation, particularly in the ischemic penumbra [258]. Activated microglia migrate to the injured area, where they release proinflammatory cytokines, NO, ROS, prostaglandins (PGs), MMPs, and chemokines, causing a series of destructive effects [259, 260]. Microglia primarily mediate neuroinflammatory responses through NLRP3 inflammasomes (Figure 3), leading to neuronal death and BBB disruption. ATP activates P2X7 receptors in microglia, promoting NLRP3 inflammasome activation and caspase‐3‐dependent neuronal apoptosis [261]. Once activated, the NLRP3 inflammasome promotes caspase‐1‐dependent cleavage of pro‐IL‐1β into its mature form, leading to the release of proinflammatory cytokines like IL‐1β and IL‐18 [262, 263]. The activation of NLRP3 in microglia synergistically upregulates MMP‐2 and MMP‐9, reduces the expression of tight junction proteins, and increases EC permeability, ultimately leading to the destruction of the BBB's integrity [264].

Neutrophils are the earliest circulating immune cells to enter brain tissue from the periphery after IS [258, 265]. Circulating immune cells rely on (1) tethering, (2) rolling, (3) crawling, (4) arrest, (5) immune cell crossing of ECs, (6) basal layer disruption, crossing of the BBB, and infiltration into brain tissue [266]. The infiltration of neutrophils occurs within a few minutes of IS, significantly increases after 3 h, and reaches its peak between 24 and 72 h, mainly N1 type neutrophils [265, 267].

Neutrophils exacerbate neuroinflammation and ischemic–reperfusion injury by releasing ROS, proteases (e.g., metalloproteinases), proinflammatory cytokines (e.g., IL‐1β, TNF‐α), and neutrophil extracellular traps (NETs) [268]. ROS activate pathways like myosin light chain kinase, protein kinase C (PKC), MAPK, and RhoGTPases, which damage tight junction proteins (e.g., cadherin‐β‐catenin complex, occludin, ZO‐1, and claudin‐5) and reorganize the EC cytoskeleton [269]. Neutrophils also release various proteases during degranulation, including MMPs, proteinase 3, and elastase. MMPs directly contribute to the destruction of the BBB by degrading basement membrane (BM) proteins like type IV collagen [270].

NETs are a network structure composed of a DNA backbone, histones, and granule proteins released by activated neutrophils. They are significantly increased in the plasma of IS patients and are associated with stroke severity and mortality. NETs can reduce pericellular coverage, increase BBB permeability, promote extravascular IgG deposition, and lead to microvascular obstruction [265, 271, 272, 273].

#### 4.1.4 Programmed Cell Death

PCD is a crucial process in regulating cerebral ischemia and reperfusion injury. After a stroke, the main types of PCD include apoptosis, necroptosis, pyroptosis, and ferroptosis [274, 275, 276].

Following an IS, many neurons in the ischemic penumbra or surrounding areas undergo apoptosis hours or even days later [277]. Apoptosis is a highly regulated, energy‐dependent form of cell death that processes excess or damaged cells. It is an organized process that dismantles cells from the inside, minimizing damage to neighboring cells. Its characteristic morphological changes include cell shrinkage, cytoplasmic condensation, nuclear envelope rupture, and the formation of apoptotic bodies [277, 278]. After a stroke, two main apoptotic pathways are triggered: the extrinsic (death receptor) pathway and the intrinsic (mitochondrial) pathway (Figure 2). Immune cells activated during poststroke inflammation can release proapoptotic ligands such as TNF‐α, FasL, and TRAIL. These cytokines bind to their corresponding death receptors (TNF receptor 1, Fas/CD95/APO1, and TRAIL‐R), initiating the extrinsic pathway and ultimately leading to neuronal death [279, 280]. When ligands like FasL bind to cell surface death receptors, they recruit the Fas‐associated death domain protein (FADD). FADD then binds to procaspase‐8 to form a death‐inducing signaling complex, which activates caspase‐8 [281]. Activated caspase‐8 can directly activate caspase‐3, which cleaves DNA repair enzymes like poly (ADP‐ribose) polymerase (PARP), causing nDNA damage and apoptosis [282, 283]. Overactivation of PARP can deplete nicotinamide adenine dinucleotide and ATP, leading to cellular energy depletion and necrotic cell death [284]. Activated caspase‐8 also cleaves Bid into truncated Bid (tBid), which participates in the intrinsic pathway [277]. An IS promotes Ca^2+^ influx by activating acid‐sensing ion channels or through glutamate‐mediated activation of NMDARs or AMPARs, leading to intracellular calcium overload [285, 286]. This increase in calcium activates calpain, which mediates the cleavage of Bid into tBid [287, 288]. tBid then induces conformational changes in proapoptotic members of the Bcl‐2 family, such as Bak, Bax, Bad, and BclXS, which bind tightly to the outer mitochondrial membrane (OMM) [289, 290]. tBid can also form heterodimers with antiapoptotic Bcl‐2 family members (e.g., Bcl‐2 and Bcl‐xL) via its BH3 domain, disrupting their inhibitory effects and promoting apoptosis [289]. After these proapoptotic proteins form heterodimers with tBid, the OMM becomes permeable, the mitochondrial transition pore opens, and Cyt*c* or apoptosis‐inducing factor (AIF) is released [291]. Cyt*c* binds to Apaf‐1 and procaspase‐9 to form an “apoptosome,” which activates caspase‐9 and subsequently caspase‐3. As mentioned, caspase‐3 cleaves critical cellular protein substrates, directly leading to cell death [292]. AIF translocates to the nucleus and mediates large‐scale DNA fragmentation and cell death in a caspase‐independent manner [293].

Unlike apoptosis, necroptosis is a caspase‐independent form of PCD typically activated as a backup mechanism when apoptosis is inhibited. It is characterized by cell swelling and membrane rupture, which releases intracellular contents and triggers an inflammatory response [294, 295]. In IS, receptor‐interacting serine/threonine protein kinase 1 (RIPK1), RIPK3, and mixed lineage kinase domain‐like protein (MLKL) are key components of the necroptosis signaling pathway. RIPK1 activates RIPK3 and MLKL through phosphorylation, leading to the necroptosis of neurons and glial cells [296, 297, 298]. Under ischemic and hypoxic conditions, inflammatory factors like TNF‐α and TLR ligands activate death receptors, triggering the necroptotic signaling cascade [297]. When TNF‐α binds to TNF receptor 1 (TNFR1), it trimerizes and recruits a complex of proteins, including TNFR‐associated death domains, apoptosis inhibitors (cIAP), and TNFR‐associated factor 2/5 (TRAF2/5) to the plasma membrane, forming Complex I [297, 299]. In this complex, RIPK1 is ubiquitinated by cIAP1/2, which recruits transforming growth factor‐activated kinase 1 (TAK1) to activate the IκB kinase complex, thereby activating the prosurvival NF‐κB signaling pathway [299, 300]. When RIPK1 ubiquitination is inhibited or deubiquitinated by CYLD, RIPK1 and TRADD are released from the plasma membrane and bind to FADD and caspase‐8 to form Complex II in the cytoplasm. In this complex, activated caspase‐8 triggers apoptosis, while caspase‐8 inhibits necroptosis by cleaving RIPK1 and RIPK3 [301]. If caspase‐8 activity is inhibited, RIPK1 undergoes autophosphorylation (at the Ser166 site), recruiting and phosphorylating RIPK3 via the RHIM domain. This RIPK1–RIPK3 interaction forms a necrosome [302, 303, 304]. Subsequently, RIPK3 phosphorylates MLKL, which then oligomerizes and translocates to the plasma membrane, causing a series of damaging reactions [305, 306, 307]. MLKL can interact with Ca^2+^ and Na⁺ channels, causing an influx of cations that increases cellular osmotic pressure, leading to membrane rupture [308]. MLKL can also directly disrupt cell membrane integrity by forming transmembrane pores, which releases DAMPs and inflammatory factors (e.g., IL‐1β, TNF‐α). This promotes neuroinflammation and further exacerbates neurovascular unit damage [304, 305, 309, 310]. Necroptosis and ferroptosis can also be linked; iron overload promotes RIPK1 phosphorylation and accelerates necroptosis by opening the mitochondrial MPTP [311].

Pyroptosis is an inflammatory form of PCD activated by caspases and inflammasomes, and it plays a key role in the pathogenesis of IS [312]. It is regulated through two main pathways: the classical pathway mediated by caspase‐1 and the nonclassical pathway mediated by caspase‐4/5/11^313^. After an IS, ischemic and hypoxia cause necrotic cells to secrete DAMPs and pathogen‐associated molecular patterns (PAMPs). These molecules activate and assemble inflammasomes [314, 315]. The activated inflammasome cleaves procaspase‐1 into its mature form [316, 317]. Activated caspase‐1 then cleaves gasdermin D (GSDMD) at Asp275, yielding a 30 kDa N‐terminal fragment (GSDMD N‐terminus) and a 22 kDa C‐terminal fragment (GSDMD C‐terminus) [318]. The N‐terminal fragment of GSDMD translocates to the plasma membrane to form pores, which promote K⁺ efflux and Ca^2+^ influx. This leads to cell swelling, lysis, and the release of large amounts of intracellular contents, thereby promoting inflammation [313, 319]. Activated caspase‐1 also cleaves pro‐IL‐1β and pro‐IL‐18 into their mature forms, which are secreted through GSDMD pores, further exacerbating the inflammatory response [320, 321, 322]. In nonclassical pathways, LPS directly induces caspase‐4/5/11 to cleave GSDMD. Caspase‐11 is mouse‐specific, while caspase‐4/5 are human‐specific [313, 323, 324]. Additionally, GSDMA/B/C/E can be cleaved by other proteases (e.g., SpeB, granzyme A, caspase‐8, caspase‐3), which can also induce pyroptosis [325, 326, 327, 328]. Pyroptosis is a multicell process that plays a critical role in the pathological events following an IS. In the microglia, TLR‐4 on the microglial membrane recognizes PAMPs and DAMPs, activating the NF‐κB pathway via its adaptor molecule MyD88. This increases the synthesis of NLRP3 and pro‐IL‐1β. NLRP3 then rapidly assembles with ASC and procaspase‐1 to form the NLRP3 inflammasome, activating the canonical pyroptosis pathway [313]. In the astrocytes, DAMPs and PAMPs can convert astrocytes into the A1 phenotype, which releases cytotoxins, damages neurons, and induces intracellular inflammasome assembly, leading to pyroptosis [313, 329]. The GSDMD pores open ion channels, causing Ca^2+^ influx and mitochondrial Ca^2+^ overload, which results in mitochondrial dysfunction [330]. The influx of ions causes high osmotic pressure in astrocytes, leading to the opening of aquaporin channels, continuous swelling, and eventual rupture and death [331]. Astrocytes are crucial for maintaining brain homeostasis, and their pyroptosis leads to a loss of homeostasis, disruption of neuronal metabolism, and a worsening of brain damage [332]. Neuronal pyroptosis is primarily induced by inflammatory factors and ROS released by glial cells. These factors can induce NLRP3 inflammasome assembly in neurons, leading to cell death. Activated M1 microglia produce and release large amounts of ROS through the HV1 channel, which induces NLRP3 inflammasome assembly and neuronal pyroptosis [333]. The neuronal pyroptosis pathway is highly dependent on caspase‐1 [334]. AIM2 is highly expressed in poststroke neurons, where it recognizes abnormal double‐stranded DNA and binds to ASC to form an inflammasome, which activates caspase‐1 and participates in the cleavage of IL‐1β, IL‐18, and GSDMD [335, 336]. NLRP1 is also mainly expressed in poststroke neurons, and chemokine receptors CCR5 and CXCR4 are involved in its assembly. The unique pyrin domain of NLRP1 can directly bind to procaspase‐1, promoting the cleavage of GSDMD by caspase‐1 and IL‐1 [337, 338, 339]. BMECs may also undergo pyroptosis. In the early stages of a stroke, IL‐1 and ROS released by microglia induce pyroptosis in BMECs, leading to cytoskeletal rearrangement and loss of tight junction protein function, ultimately causing initial BBB damage [340, 341, 342]. After BMEC pyroptosis, mitochondrial dysfunction leads to the release of large amounts of ROS, which can damage other cells that constitute the BBB, further disrupting its integrity [342]. BMEC pyroptosis also releases a large number of inflammatory factors, especially ICAM‐1, which attracts neutrophils to migrate from capillary walls into the brain to infiltrate the tissue [343].

Ferroptosis is a form of iron‐dependent cell death closely related to pathological processes such as autophagy, inflammation, and oxidative stress. Its main mechanisms of damage include iron metabolism imbalance, lipid peroxidation, and an impaired antioxidant system [344, 345]. Iron homeostasis is fundamental to maintaining normal brain physiology. During an IS and reperfusion, the metabolic balance of iron is disrupted, leading to iron overload, a key factor in triggering ferroptosis and cell death [346]. The imbalance is caused by a disruption between iron uptake and efflux. After a stroke, the BBB is compromised, allowing free iron and ferritin to enter the brain parenchyma [347]. The acidic environment of an IS induces the dissociation of iron from transferrin (TF), increasing extracellular iron levels and its transfer into neurons, leading to increased intracellular iron uptake [348, 349]. Simultaneously, the expression of ferroportin (FPN), the only protein that exports iron from cells, is downregulated after cerebral ischemic, which further exacerbates intracellular iron overload [350, 351]. Ferrous iron (Fe^2+^) participates in intracellular redox reactions, particularly the Fenton reaction. When Fe^2+^ reacts with hydrogen peroxide, it generates highly reactive ⋅OH, which trigger oxidative damage, induce iron toxicity, and lead to cell death [347, 352]. After a stroke, excessive iron and ROS production further promote lipid peroxidation. The main substrate for this process is polyunsaturated fatty acids (PUFAs) [345]. After ischemia, ALOX15 levels increase in neurons and ECs; ALOX15 can directly oxidize lipid membranes containing PUFAs [353]. Calcium‐dependent cPLA2α, an enzyme that regulates the release of AA in PUFAs, is upregulated after a stroke. The Ca^2+^ influx activates cPLA2α to enhance lipid peroxidation, exacerbating ROS levels, infarct volume, and BBB permeability [354, 355, 356]. ACSL4 is also upregulated after an IS, catalyzing AA to form AA‐CoA [357, 358]. This is then incorporated into membrane phospholipids by lysophosphatidylcholine acyltransferase 3 to produce AA‐PE or AdA‐PE [359]. Finally, LOX oxidizes AA‐PE into the lipid peroxide PE‐AA‐OH, with Fe^2+^ being a key component of the LOX catalytic subunit [359]. Membrane lipid peroxidation can also promote the production of 4‐HNE, which covalently modifies membrane transporters such as Na⁺/K⁺‐ATPase, glucose transporters, and glutamate transporters, thereby impairing cellular function [360]. Lipid peroxidation can also directly damage the cell membrane, leading to cellular dysfunction and death [361, 362]. The GSH/GPX4 system is the most well‐known antioxidant pathway. After a stroke, GSH and GPX4 are inhibited by the increased extracellular glutamate concentration and hypochlorous acid (HOCl) produced by MPO [363, 364, 365, 366]. In the ischemic brain, ATP depletion, cell swelling, and glutamate receptor activation lead to increased glutamate release and decreased glutamate uptake [366, 367, 368]. The availability of cysteine regulates GSH synthesis, and a decrease in intracellular cysteine can lead to GSH depletion, GPX4 inhibition, and loss of cellular antioxidant capacity, which ultimately results in lipid peroxidation and iron toxicity [369, 370]. Therefore, the increased extracellular glutamate concentration enhances excitotoxicity and suppresses GPX4 production via the glutamate/cysteine antiporter systemic xc‐system, causing iron toxicity [371]. MPO produces HOCl at the site of injury and inflammation, which rapidly inactivates GPX [372]. Histone deacetylase 9 (HDAC9) is overexpressed in ischemic brain tissue, where it promotes HIF‐1‐induced TfR1 expression by mediating the deacetylation and deubiquitination of HIF‐1. HDAC9 also mediates the deacetylation and ubiquitination of Sp1, downregulating its target gene GPX4 [373]. The increased HIF‐1/TfR1 pathway and decreased Sp1/GPX4 pathway are involved in HDAC9‐mediated ferroptosis after IS, exerting a neuroprotective effect [374]. When the antioxidant defense system (e.g., glutathione, GPX4) is imbalanced, it cannot clear the ROS caused by iron, leading to a sharp increase in intracellular ROS levels. This further exacerbates oxidative stress, neuroinflammation, and neurotoxicity, ultimately leading to cell death [345, 374, 375]. Ferroptosis can also synergize with other PCD pathways, such as necroptosis, to exacerbate ischemic injury [376].

#### 4.1.5 BBB Disruption

The BBB is a crucial biological structure on the walls of cerebral blood vessels. It is composed of brain microvascular ECs, a BM, pericytes (PCs) embedded in the BM, and surrounding astrocytes [377]. The BBB acts as a selective interface, forming a diffusion barrier that regulates which substances from the blood can enter the CNS, protecting it from harmful toxins and pathogens while allowing essential nutrients to pass [378, 379].

Disruption of the BBB is a major adverse event after an IS (Figure 3). This disruption and the resulting increase in BBB permeability occur in two stages [380]. Stage 1 (reversible) occurs within the first hour after ischemic. Oxidative stress‐induced ROS and MMP‐2 degrade tight junction proteins, leading to their dysfunction and increased BBB permeability. This allows immune cells and plasma proteins to enter the brain, exacerbating the inflammatory response [381]. Stage 2 (irreversible) occurs between 24 and 72 h after ischemia. At this stage, the activation of MMPs, particularly MMP‐9, is highly significant. Astrocytes and microglia are major producers of MMP‐9. As neutrophils infiltrate the brain from the periphery, they carry MMP‐9, which degrades the BM and ECM, causing a breakdown of BBB integrity and vascular edema [265, 381].

The BBB's structural damage after a stroke is a complex process involving multiple components: ECs connected by tight junction proteins are the core of the BBB. Hypoxia and oxidative stress damage them, leading to structural and functional disruption [382]. Focal ischemia leads to upregulation of PKCδ, increasing MMP9 activity, which in turn upregulates aquaporin 4 (AQP4) expression, causing excessive water to enter the brain and triggering poststroke edema [383]. ROS and RNS increase BBB permeability by damaging the EC membrane and inducing apoptosis [384, 385].

Tight Junction proteins (e.g., claudin, occludin) are key for maintaining the BBB's integrity. Poststroke oxidative stress and MMPs, especially MMP‐2 and MMP‐9, degrade them, leading to a loss of barrier function and allowing substances and inflammatory cells to enter the brain, which exacerbates stroke consequences and neuronal damage [386, 387, 388, 389]. Cav‐1 is a key scaffold/regulatory protein of Caveolae in lipid rafts, mediating pathophysiological processes including Cav‐1‐dependent endocytosis. Following IS, Cav‐1 expression is upregulated, mediating the redistribution and degradation of claudin‐5, disrupting tight junction proteins, and increasing BBB permeability [390].

BM is located between ECs and astrocytes, providing structural support. Poststroke MMP‐9 activation degrades BM proteins like collagen and laminin, compromising its structure and further increasing BBB permeability [389, 391].

PCs maintain BBB stability by regulating endothelial tight junctions and blood flow. A stroke's hypoxia and oxidative stress cause PC dysfunction or loss. This separates PCs from ECs, deteriorating tight junctions, and leading to neuroinflammation and BBB damage [381, 392, 393, 394].

Astrocytes are a crucial part of the neurovascular unit, their activation in the acute phase of a stroke can lead to the secretion of proinflammatory cytokines, which inhibits axon generation and increases BBB permeability [393, 395].

#### 4.1.6 Angiogenesis

Angiogenesis is the formation of new blood vessels. After an IS, there are three primary forms: vascular remodeling, sprouting angiogenesis, and the formation of new mature arteries from existing arterioles [396]. Studies show that cerebral ischemic can induce transient angiogenesis, which begins in the ischemic penumbra 3–4 days after the stroke [397]. Studies show that cerebral ischemia can induce transient angiogenesis, which begins in the ischemic penumbra 3–4 days after the stroke. This process, primarily through sprouting, restores blood flow and metabolism in the ischemic brain, promoting nerve regeneration and functional recovery [203, 398, 399].

**FIGURE 4:**
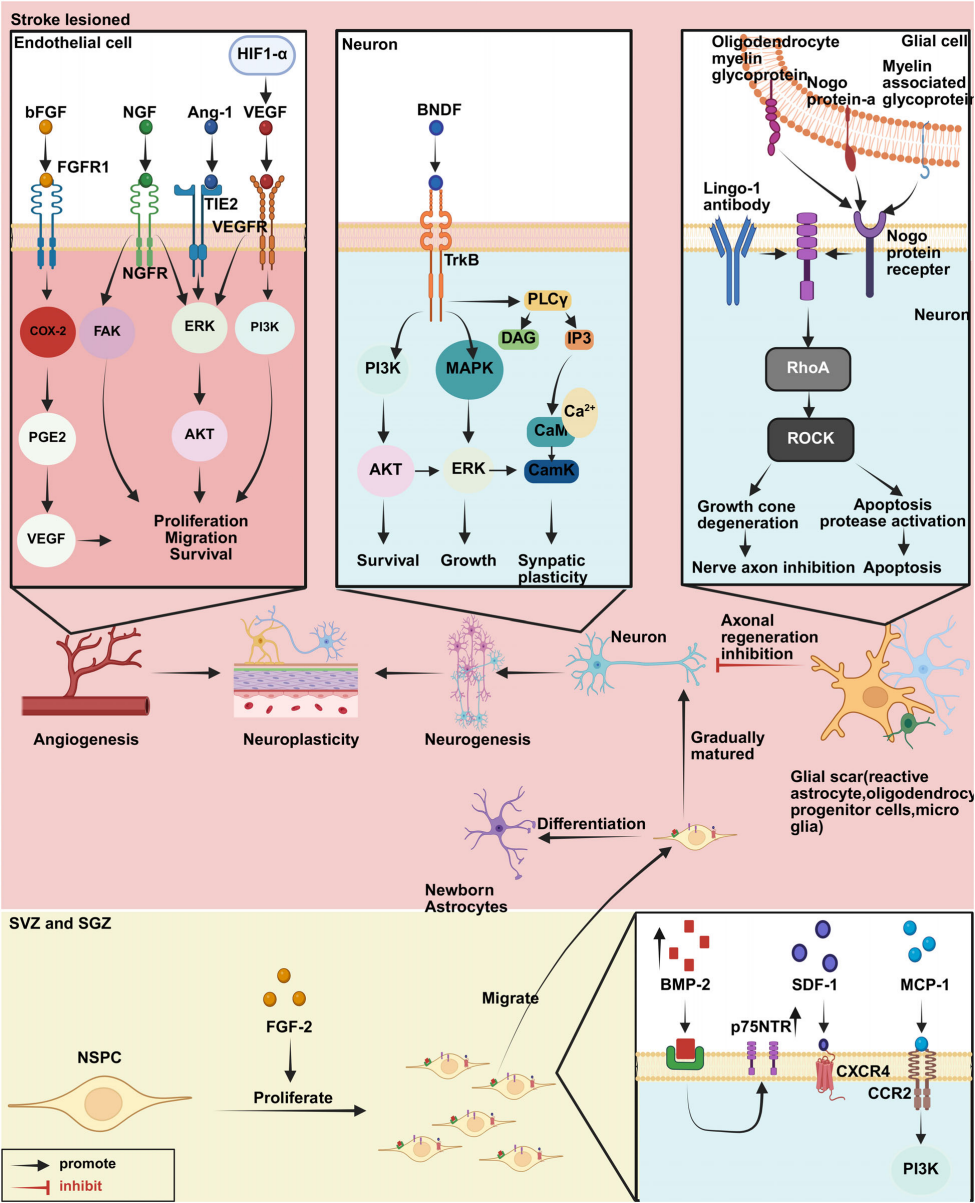
Schematic diagram of endogenous repair mechanisms following IS: neurogenesis, angiogenesis, and axonal regeneration inhibition. Following an IS, the brain initiates multiple endogenous repair processes. Angiogenic factors (bFGF, NGF, VEGF, Ang‐1) bind to their respective receptors, activating downstream signaling pathways (PI3K/AKT, ERK, COX‐2/PGE2, FAK), driving endothelial cell proliferation, migration, and survival, and promoting angiogenesis. NSPCs in SVZ and SGZ proliferate under the influence of FGF‐2, and BMP‐2 induces p75 abundance, enhancing NSPC migration ability. NSPCs migrate toward the lesion site under the influence of chemokines (such as SDF‐1 and MCP‐1), differentiating into neurons and astrocytes. BDNF activates the PI3K/AKT, MAPK/ERK, and PLCγ/IP3/Ca^2+^/CAMK pathways, influencing neuronal survival, growth, and synaptic plasticity. This process is limited by glial scarring. Myelin‐associated glycoproteins (Nogo, OMGp, MAG) bind to their receptors, activating the RhoA/ROCK pathway in neurons, leading to inhibited axon regeneration, which in turn triggers nerve axon inhibition and neuronal apoptosis. *Abbreviations*: AKT, protein kinase b (PKB); ANG‐1, angiopoietin‐1; BFGF, basic fibroblast growth factor; BMP‐2, bone morphogenetic protein‐2; CAM, calmodulin; CAMK, calcium/calmodulin‐dependent protein kinase; CCR2, chemokine (C‐C motif) receptor 2; COX‐2, cyclooxygenase‐2; CXCR4, C‐X‐C chemokine receptor type 4; DAG, diacylglycerol; ERK, extracellular regulated protein kinase; FAK, focal adhesion kinase; FGF‐2, fibroblast growth factor‐2; HIF1‐α, hypoxia‐inducible factor 1‐α; IP3, inositol triphosphate; MAPK, mitogen‐activated protein kinase; MCP‐1, monocyte chemoattractant protein‐1; NGF, nerve growth factor; NSPC, neural stem/progenitor cells; P75NTR, the p75 neurotrophin receptor; PGE2, prostaglandin E2; PI3K, phosphatidylinositol 3‐kinase; PLCγ, phospholipaseγ; RhoA, ras homolog gene family member a; ROCK, Rho‐associated protein kinase; SDF‐1, stromal cell‐derived factor‐1; VEGF, vascular endothelial growth factor.

The process of angiogenesis is mediated by various molecules, including growth factors, cytokines, and angiogenic mediators (Figure 4) [202]. Stroke‐induced ischemic and hypoxia increase the expression of VEGF, a core angiogenic factor, by upregulating the HIF‐1α system [400, 401, 402, 403, 404]. ECs around the infarct begin to proliferate within 12–24 h [405] NO and VEGF cause local vasodilation, loosening endothelial tight junctions and PCs, leading to plasma protein extravasation [396]. Reactive astrocytes reconstruct the ECM, providing a scaffold for EC migration [406]. VEGF then binds to its receptors on ECs, promoting their proliferation and migration to initiate angiogenesis [407]. Activated ECs are regulated by various factors (VEGF, FGF, PLGF, etc.), which release proteases to degrade the ECM, allowing the cells to proliferate and migrate outward, forming vascular sprouts [202, 399]. These cells form a solid string that, under the stimulation of VEGF and other factors, gradually organizes into a tubular structure with a lumen [202, 405]. Once a vascular lumen forms, the new blood vessel matures. VEGF recruits PCs and VSMCs to the vessel wall, providing stability and structural support [408]. The new vessels are then trimmed into a functional network that integrates with surrounding tissues, ensuring stable blood flow [396].

Various molecules and their signaling pathways also regulate angiogenesis. VEGF activates downstream signaling pathways such as ERK and PI3K/AKT, promoting angiogenesis after stroke by enhancing EC proliferation and survival [399]. FGFR1, upregulated in PCs after a stroke, activates the AKT/ERK pathway, inducing the expression of bFGF and PDGFRβ [409, 410]. bFGF promotes neurogenesis and angiogenesis by increasing PGE2 and VEGF expression [411, 412]. NGF promotes angiogenesis by activating the FAK or PI3K/AKT pathways [413, 414]. Angiopoietins (Angs) are important for vascular maturation and stability, with Ang‐1 promoting EC proliferation and survival, while Ang‐2 and VEGF disrupt the BBB to promote angiogenesis [415, 416].

An increased number of microvessels in the infarcted area of stroke patients is associated with longer survival [417]. Therefore, treatment involving the promotion of angiogenesis in the surrounding area of IS patients can effectively reduce infarct volume, promote neuronal cell survival, restore neurovascular network function, and improve patient prognosis, making it a potential target for the treatment of IS [418].

#### 4.1.7 Neural Circuit Reconstruction

The brain has significant plasticity and reorganization ability, and neuroplasticity refers to the ability of the brain to respond to internal or external stimuli, change its morphological structure and functional activity [419]. After an IS, the damaged brain initiates neuroprotective effects and recovery (Figure 4). This spontaneous neuroplasticity includes neurogenesis, angiogenesis, and synaptic development [420]. The regeneration of neurons and the spread of axons facilitate the re‐establishment of pathways, while new synapse formation stabilizes these connections. These processes collectively promote neural circuit reconstruction, ultimately restoring some neural behaviors and functions such as sensory perception, motor behavior, and cognition [420, 421, 422].

Neurogenesis is the process of generating new, functional neurons from neural stem/progenitor cells (NSPCs). It involves the proliferation, migration, and differentiation of endogenous NSPCs into mature neurons, primarily occurring in the subventricular zone (SVZ) and the subgranular zone (SGZ) of the hippocampus [423]. After a stroke, endogenous NSPCs in these areas are activated and migrate to the infarct, where they differentiate into neurons [424]. A stroke shortens the NSPC cell cycle, increasing the percentage of proliferating cells and promoting NSPC proliferation in the SVZ, which expands the NSPC pool [425, 426, 427]. The expression of FGF‐2 and its receptor significantly increases after a stroke, promoting NSPC proliferation [428]. Proliferating neuroblasts migrate from the SVZ through the brain parenchyma to the border of the infarcted area, where they differentiate into mature neurons [429, 430, 431]. This migration is guided by cellular interactions and chemokines. The expression of CXCR4 increases on NSPCs and neuroblasts, and SDF‐1 released from reactive astrocytes and vascular cells acts on CXCR4 to promote their migration [432, 433]. Similarly, MCP‐1 from activated astrocytes and microglia interacts with the CCR2 receptor on NSPCs, increasing their migration via the PI3K pathway [434, 435]. Furthermore, stroke increases BMP‐2 levels, inducing an increase in the abundance of p75 neurotrophic protein receptor in endogenous NSPCs in the SVZ, thereby promoting the migration of SVZ NSPCs to ischemic brain tissue regions [436]. IS also induces upregulation of BDNF and its receptor expression, which can increase neurogenesis in the dentate gyrus and promote neurological function recovery [437, 438, 439].

Despite this regenerative potential, the hostile microenvironment in and around the infarct, including ischemia and hypoxia, causes apoptosis of these NSPCs, limiting neural network reconstruction [424]. Furthermore, the formation of glial scars and the production of axon‐inhibitory proteins like chondroitin sulfate proteoglycans can block neuroblast migration [440, 441, 442]. Thus, the net migration of neuroblasts after a stroke depends on the balance between inhibitory molecules and chemokines, making this balance a potential therapeutic target [443].

After a stroke, the ischemic brain suffers damage to its synaptic structure, including synapse loss, dysregulation of synaptic adhesion, and insufficient synaptic repair, which impairs spatial learning and memory [444, 445]. Synaptogenesis involves three key processes: the formation of neurotransmitter release sites in presynaptic neurons, the establishment of sensory regions in postsynaptic cells, and precise alignment of presynaptic and postsynaptic structures [446, 447, 448]. Glial cells, particularly astrocytes, are crucial for regulating poststroke synaptic development [449, 450, 451]. Astrocytes can be converted into a prorecovery phenotype by the protein lipocalin 2, expressed by neurons after a stroke [452]. Agrin‐a, derived from astrocytes, acts on hippocampal neurons to initiate synaptic development [453]. TSP‐1 and TSP‐2 in astrocytes are elevated after IS, which may also be one of the mechanisms by which astrocytes induce synaptic development after ischemia [454]. The interaction between the processes of astrocytes and the presynaptic and postsynaptic terminals forms a “triple synapse,” promoting synaptic structural plasticity and inducing the formation and stability of new synapses [455, 456]. Astrocytes actively clear glutamate from synaptic gaps via transporters like GLT‐1/EAAT2 and GLAST, preventing toxic accumulation and maintaining the fidelity of synaptic transmission [457, 458]. Astrocytes also release glial neurotransmitters (e.g., ATP, D‐serine, GABA) to regulate synaptic plasticity and long‐term potentiation, processes critical for learning and memory [459, 460, 461]. In some cases, reactive astrocytes can differentiate into functional GABAergic or cholinergic neurons, which form new synapses and integrate into local neural circuits [462, 463].

Axon sprouting is the growth of new branches on existing axons, complementing neurogenesis and synaptic plasticity. This process, which begins about 3 weeks after a stroke and can last for months, helps repair central connections and alleviate functional deficits [464, 465, 466, 467, 468]. GDF10 is a key trigger for poststroke axonal sprouting, and EPO can upregulate its expression via the JAK2/PI3K pathway [469, 470]. Activated astrocytes are the main cells involved in this process, promoting sprouting by secreting GDF10 and regulating axonal reorganization via ephrinA1 and ephrin‐A5 [470, 471, 472].

Neurotrophic factors, including NGF, BDNF, NT‐3, and NT‐4/5, are upregulated and released in the injured area, playing an important role in neurological recovery after IS [473]. Taking BDNF as an example, BDNF acts on TrkB, causing its phosphorylation and mediating the activation of multiple downstream pathways. The PI3K pathway activates protein kinase B (AKT), promoting neuronal survival. The MAPK/ERK pathway promotes neuronal cell growth and differentiation. The PLC pathway activates inositol triphosphate (IP3) receptors to release intracellular calcium reserves, thereby enhancing CaM kinase (CAMK) activity and increasing synaptic plasticity [474]. BDNF, along with other neurotrophic factors, also stimulates axonal growth by mediating the polymerization and accumulation of F‐actin in the growth cone and axonal axis [475].

Overall, the brain spontaneously reconstructs neural circuits after a stroke through neurogenesis, synaptogenesis, and axonogenesis. However, this repair is often incomplete due to the limited regenerative capacity of neurons and the presence of inhibitory factors. Therefore, promoting these neuroplasticity‐mediated structural and functional changes is a significant goal for stroke treatment and prognosis [420, 421, 476].

#### 4.1.8 The Repairing Effect of Neuroinflammation

Neuroinflammation after a stroke has a dual effect. In the acute phase, it is destructive, driven by activated glial cells and peripheral immune cells that cause secondary brain damage. However, during the recovery period, neuroinflammation has a protective and reparative effect, participating in various endogenous brain repair processes to promote functional recovery. The classic microglia: M1 phenotype is destructive, releasing proinflammatory mediators and NO [477, 478]. In contrast, the reparative M2 phenotype secretes anti‐inflammatory factors (e.g., IL‐4, IL‐10, TGF‐β) and nutritional factors (e.g., BDNF, IGF‐1), which inhibit inflammation and promote the proliferation and migration of neural precursor cells [479, 480, 481, 482, 483, 484]. M2 microglia also produce proangiogenic factors (e.g., IL‐6, VEGF, MMPs) to support poststroke angiogenesis [259, 485, 486, 487]. They also help with synaptic pruning and BBB remodeling by enhancing the expression of tight junction proteins during recovery [488, 489].

During recovery, neutrophils can transition to the anti‐inflammatory N2 phenotype, which helps clear other neutrophils, promoting the resolution of neuroinflammation [490, 491]. They also degrade the ECM, releasing growth factors like VEGF and TGF‐β, and clear cellular debris through phagocytosis, creating a better microenvironment for neurovascular unit formation [492, 493, 494].

The phenotype of monocytes changes from predominantly proinflammatory M1 (day 3) to anti‐inflammatory M2 (day 7) after a stroke [495]. M2 macrophages produce anti‐inflammatory cytokines (e.g., CD206, IL‐10) and growth factors (e.g., FGF, TGF‐α, IGF) that weaken inflammation and promote neurovascular unit reconstruction [496, 497, 498]. Their phagocytic activity also clears debris, and they promote axonal sprouting by releasing IL‐10 and oncomodulin [499, 500, 501]. M2 macrophages can also repair the BBB by activating signaling pathways and physically pulling the ruptured ends of vessels together [502, 503, 504, 505]. Infiltrating T cells promote poststroke recovery by secreting growth factors and cytokines [506]. They produce BDNF to support nerve regeneration and interferon‐γ to regulate cognitive behavior [507, 508]. Regulatory T cells are a key group of peripheral immune cells involved in repair. They negatively regulate neuroinflammation by releasing anti‐inflammatory cytokines (e.g., TGF‐β, IL‐10) and can promote NSPC proliferation and reduce hemorrhage [509, 510, 511, 512]. They also protect the BBB by inhibiting MMP‐9 production in neutrophils and downregulating inflammatory molecules in ECs [513, 514, 515]. B cells play a dual role. During recovery, they secrete anti‐inflammatory IL‐10, which reduces infarct severity and improves neurological function [516]. Regulatory B cells have an immunosuppressive function, protecting against neurological deficits [517, 518, 519]. B cells are a main source of BDNF, which is essential for neuronal function and may promote early neuroprotection after a stroke [520, 521].

In summary, neuroinflammation after an IS is a dynamic process with both damaging and reparative effects. Its persistence in the late stage of stroke and its involvement in brain repair make it an important target for improving functional recovery [451, 522].

### 4.2 Pathophysiological Processes and Related Signaling Pathways After HS

HS can lead to brain damage through multiple mechanisms, which can be broadly classified into primary and secondary damage. Primary injury occurs within the first few hours of onset, mainly due to mechanical damage to adjacent tissues caused by the hematoma through dissection and compression. The size of the hematoma is a crucial indicator of the severity of HS and prognosis; a hemorrhage volume >100 mL suggests a worse prognosis and is often accompanied by complications such as increased ICP, decreased CBF, and brain herniation [11]. Secondary damage occurs days to weeks after HS and is a result of the immune and inflammatory cascade triggered by hematoma's toxic components and metabolism in the brain parenchyma. It is the result of the combined action of multiple damage pathways such as neuroinflammation, oxidative stress, ferroptosis, and others, leading to disruption of the BBB. In recent years, with the development of minimally invasive surgical and endoscopic techniques, early hematoma evacuation surgery has achieved good results in controlling hematoma size and complications. However, it has not been effective in functional recovery. This may be related to the fact that hematoma evacuation surgery does not address secondary brain injury following HS [523]. Therefore, we will focus on introducing the mechanisms and related signaling pathways of secondary damage after HS, in order to find new ideas for the treatment of HS.

#### 4.2.1 Neuroinflammation

The main manifestations of neuroinflammation after HS are M1‐type microglial activation, proinflammatory cytokine secretion, and peripheral inflammatory cell infiltration [524, 525].

In the early stage after HS, damaged or dead cells (such as necrotic or lysed red blood cells and nerve cells), components within the hematoma (such as hemoglobin, cell debris, ATP, etc.), and some cytokines (such as HMGB1, extracellular peroxidase protein family, prolactin‐3) are released into the brain tissue, becoming DAMPs [526, 527]. M1‐type microglial cells are activated by DAMPs following hemorrhage. These DAMPs are recognized by PRRs on the microglial cell surface, including TLRs, nucleotide‐binding oligomerization domain (NOD)‐like receptors (NLRs) that form inflammasomes, and triggering receptors expressed on myeloid cells (TREMs) [528, 529].

The TLRs on the surface of microglia are predominantly TLR‐4 [529]. After TLR‐4 binds to its ligand (such as HMGB1), it activates the HMGB1/TLR‐4/NF‐κB signaling pathway via the myeloid differentiation primary response protein (MyD88) dependent pathway, promoting the expression and secretion of proinflammatory factors such as TNF‐α, IL‐6, IL‐1β, and iNOS, thus initiating neuroinflammation [530, 531]. TNF‐α, a major proinflammatory cytokine in neuroinflammation, can bind to the TNFR1 and promote the secretion of IL‐6, IL‐1β, iNOS, and so on through the TNF‐α/TNFR1/NF‐κB pathway, further aggravating neuroinflammation [532, 533]. NOS is a key mediator of immune activation and inflammation, which can oxidize l‐arginine to l‐citrulline and produce NO [534]. NO is widely involved in cell growth, differentiation, and apoptosis, with dual effects of neuroprotection and neurotoxicity. Excessive NO can lead to neuroinflammation, tissue damage, and BBB disruption [535, 536]. IL‐6 has a dual role in the CNS. Low concentrations of IL‐6 promote nerve growth, differentiation, and repair, while high concentrations can cause nerve damage. It can activate neutrophils, chemotactically attract monocytes and macrophages, activate the complement system, and induce microcirculatory disturbances, exacerbating cerebral edema [537]. Additionally, the activation of TLR‐4 can modulate the expression of COX‐2 protein in microglia induced by LPS [538]. COX‐2 is upregulated during inflammation and is a key enzyme that catalyzes the conversion of AA to PGs. In neuroinflammation, PGE2 is the predominant enzyme, and PGE2 can mediate subsequent inflammatory responses, potentially leading to further brain damage [539].

The NOD family proteins and TREM‐1 are extensively expressed on microglia and exert proinflammatory effects in neuroinflammation. Following HS, the NOD1/RIP2 signaling pathway activates the downstream JNK/P38 MAPK and NF‐κB pathways, thereby promoting the secretion of iNOS, IL‐1β, and TNF‐α. Additionally, NOD1/RIP2 engages in a reciprocal feedback loop with IL‐1β and TNF‐α, fostering their mutual activation and exacerbating neuroinflammatory injury [540]. Following HS, HMGB1 activates TREM‐1 and subsequently triggers the downstream PKCδ/caspase recruitment domain protein 9 (CARD9). CARD9 can initiate a cascade of inflammatory cytokines via NF‐κB signaling, thereby recruiting neutrophil infiltration [541].

#### 4.2.2 Oxidative Stress

As previously stated, an excessive production of ROS or an insufficient clearance capacity can lead to the accumulation of ROS within cells, resulting in a state of “oxidative stress.” Oxidative stress also plays a significant role in the process of secondary brain injury following HS. The primary sources of ROS after HS include degradation products of red blood cells, dysfunctional mitochondria, activated microglia, and infiltrating neutrophils [542].

Following an HS, a ruptured blood vessel leads to blood loss and the formation of a hematoma. Within the hematoma, hemoglobin breaks down through the hemoglobin‐heme‐iron metabolic axis, producing iron ions. These iron ions react with hydrogen peroxide in a Fenton reaction, generating highly oxidative ⋅OH, which exacerbate oxidative stress [543]. Oxidative stress can disrupt cell membranes, DNA, and proteins, resulting in cellular damage [544]. ROS, such as O_2_^−^ and OH·, can compromise the integrity of lipid bilayers, enhance membrane permeability, and cause intracellular ion imbalance, thereby further damaging nerve cells and inducing neuronal death [545].

Heme, a degradation product of hemoglobin, can ultimately be broken down into iron. An excess of iron can lead to mitochondrial dysfunction, thereby triggering the generation of ROS [545]. Research has shown that after HS, mitochondrial dysfunction caused by various factors is the direct source of ROS production, and ROS‐specific scavengers can significantly alleviate the increased ROS after HS [546].

As mononuclear phagocytes, microglia exhibit high expression levels of NOX2, an enzyme responsible for generating superoxide. The sole function of NOX family members is to produce ROS. Following HS, TLR‐4 on the microglial surface becomes activated, and this activated TLR‐4 facilitates ROS production via NOX2 [547]. Additionally, infiltrating neutrophils can also trigger NOX activation and subsequent ROS generation [548].

After HS, there exists reciprocal feedback between ROS and neuroinflammation. Inflammatory factors facilitate the release of ROS, while oxidative stress resulting from ROS accumulation can activate the genes that trigger inflammatory signaling cascades, thereby initiating inflammatory responses. Both factors can initiate multiple pathways of brain cell death, promote the breakdown of the BBB, and contribute to the vicious cycle of neurotoxicity.

#### 4.2.3 Programmed Cell Death

Following HS, microglia induce PCD and damage neurons via pathways such as apoptosis, necroptosis, pyroptosis, and ferroptosis.

Apoptosis after HS a time‐dependent increase, with neuronal apoptosis in the perihematoma region peaking at 72 h [549]. As previously stated, apoptosis occurs through both exogenous and endogenous cell death pathways, with caspase‐3 serving as a crucial mediator of neuronal apoptosis that can be activated by both pathways.

Necroptosis following HS is primarily mediated by RIPK1, RIPK3, and MLKL. After cerebral hemorrhage, microglia secrete TNF‐α, which directly induces apoptosis and cell death in neurons, ECs, and oligodendrocytes via the RIPK pathway. TNF‐α secreted by microglia activates RIPK1 [550], after which the activated RIPK1 interacts with RIPK3, triggering its phosphorylation and leading to the formation of a RIPK1/RIPK3 complex. Subsequently, activated RIPK3 recruits and phosphorylates MLKL, which then undergoes isomerization, ultimately resulting in apoptosis through mechanisms such as plasma membrane disruption and cell lysis [550, 551, 552].

Pyroptosis is closely related to inflammation after HS and is an important mechanism of inflammation‐induced neuronal cell death in HS, mainly mediated by the NLRP3 inflammasome [553, 554]. Following cerebral hemorrhage, microglia identify DAMPs and subsequently activate the NLRP3 inflammasome. This activation triggers the autocleavage of procaspase‐1 into active caspase‐1. Caspase‐1 then facilitates the proteolytic processing of pro‐IL‐1β, pro‐IL‐18, and GSDMD, transforming the precursor forms of IL‐1β and IL‐18 into their mature, biologically active states. The N‐terminal domain of GSDMD forms pores in the plasma membrane, thereby enhancing cellular permeability. This increased permeability results in the release of IL‐1β and IL‐18, further aggravating the neuroinflammation caused by HS [555, 556]. Hv1 is selectively expressed in microglia, and NOX‐dependent Hv1 produces ROS in the brain [557]. ROS can stimulate the formation of the NLRP3 inflammasome through oxidative stress, and induce the activation of NLRP3 by binding to receptors on the cell membrane (such as TLR‐4), thereby releasing proinflammatory factors such as IL‐1β and IL‐18 [558]. Furthermore, microglia are capable of inducing pyroptosis in neighboring neurons or other microglia, exacerbating neuronal pyroptosis, forming an inflammatory feedback loop, and thereby intensifying neural injury [559]. Research has demonstrated that suppressing neuronal pyroptosis mediated by NLRP1, NLRP3, and NLRP4 can markedly enhance neurological function in HS mice [560].

Ferroptosis is the main form of PCD after HS. It exists at 1 h after HS and reaches its peak at 24 h after HS [561]. Ferroptosis can lead to apoptosis, necrosis, gray matter damage, and BBB disruption through various mechanisms, and plays an important role in PCD [344, 562, 563]. Following HS, hemoglobin is engulfed by microglia and infiltrating macrophages, and subsequently metabolized into Fe^2+^/Fe^3+^, which in turn induces the formation of ROS and lipid peroxidation. The Fe^2+^ within microglia activates NOX2, resulting in an elevation of superoxide levels and ROS generation, thereby exacerbating neuroinflammation and causing inflammatory injury [564]. High levels of Fe^2+^ in cells can also lead to an imbalance in the antioxidant defense system within the cell, resulting in changes in the redox environment within the mitochondria and triggering the release of ROS [344]. High levels of ROS have a destructive effect on mitochondria, and when ROS diffuses between mitochondria, this destructive effect also spreads, ultimately leading to cell damage [565]. Excess Fe^2+^ within microglia is exported out of these cells via the iron export protein FPN, reaching adjacent neuronal cells where it binds to TF on the neuronal cell surface and subsequently enters the cells, exerting toxic effects [566]. Excessive Fe^2+^ also generates ROS through iron‐catalyzed enzymes, further promoting lipid peroxidation and ultimately leading to ferroptosis in neuronal cells [562]. Meanwhile, NCOA4‐mediated ferritin autophagy can also facilitate iron release [567]. Additionally, exosomes secreted by microglia are capable of mediating neuronal ferroptosis. These exosomes significantly decrease the levels of neuronal ferroptosis‐inhibitory proteins (GPX4, SLC7A11, and FTH1), consequently elevating Fe^2+^ and lipid peroxidation levels in neurons and mitochondria, and ultimately inducing neuronal ferroptosis [568].

#### 4.2.4 BBB Disruption

Following HS, neuroinflammation mediates the disruption of the BBB through multiple pathways. There is communication between tight junction proteins and microglia and astrocytes [569]. Microglia‐released IL‐1β and TNF‐α can downregulate the expression of tight junction proteins in ECs, consequently disrupting the BBB [570]. Additionally, through oxidative stress, the overproduction of ROS in microglia can stimulate ECs, damage tight junction proteins, and enhance the permeability of the BBB [571]. Once activated, microglia migrate to the vicinity of blood vessels, where they invade the end feet of astrocytes and impair the function of the BBB. On one hand, microglia directly interact with and infiltrate the end feet of astrocytes, disrupting their structure and function. On the other hand, microglia degrade the tight junction proteins between astrocytes and ECs, weakening the interaction between the end feet of astrocytes and the ECs of the BBB, thereby causing dysfunction of the BBB [572]. TNF‐α secreted by M1‐type microglia induces necroptosis in ECs, resulting in BBB breakdown [573]. Additionally, microglia trigger the activation of the NLRP3 inflammasome in neurons and ECs through pyroptosis, which mediates the release of inflammatory cytokines like IL‐1β, thereby promoting a cascade of inflammatory responses and further compromising the integrity of the BBB [574].

The interaction between microglia and PCs also holds significant importance in BBB disruption. On the one hand, microglia secrete IL‐1β, which induces PC apoptosis via NF‐κB activation [575]. On the other hand, when PCs are exposed to cytokines like TNF‐α and IL‐1β secreted by microglia, along with excessively generated ROS, it can trigger the production and release of IL‐6 and MMP‐9 [576]. MMP‐9 can degrade the ECM of ECs within the BBB, disrupt the tight junctions between ECs, increase the permeability of the BBB, and lead to the infiltration of immune cells, proteins, and other harmful substances into brain tissue [577].

### 4.3 Pathophysiological Processes and Related Signaling Pathways After TIA

Currently, the ASA defines TIA as a transient episode of neurological dysfunction caused by focal ischemia in the brain, spinal cord, or retina, without the presence of acute infarction, emphasizing a shift from time‐based diagnosis to incorporate tissue‐based criteria [23]. Imaging evidence such as PET and DWI/PWI indicates that TIA patients may still have diffusion–perfusion mismatch after symptom recovery. This imaging manifestation suggests that local brain tissue is still in a low‐perfusion but reversible state, known as the ischemic penumbra [12, 578]. The pathophysiological process after a TIA can be regarded as the dynamic evolution of the ischemic penumbra: local blood flow is inadequate to maintain normal electrophysiological functions, yet energy metabolism has not entirely collapsed, rendering the condition reversible. It may either shrink into benign oligemia or progress to infarction [578].

Neurological impairments observed in TIA are predominantly attributed to localized hypoperfusion. Specifically, when the local CBF drops below approximately 23 mL/100 g/min, it triggers the onset of neurological dysfunction [579]. Within the CBF range of 18–22 to 8–15 mL/100 g/min, an ischemic penumbra emerges, a region that may either recover or progress to an irreversible state [580]. Should the hypoperfusion be transient and followed by a restoration of blood flow, the neurological symptoms are reversible. Conversely, if the CBF declines further to 10 mL/100 g/min and persists at this level for 2–3 h, it culminates in the development of an irreversible infarction [579]. A reduction in blood flow initiates the ischemic cascade, which first results in a marked decrease in ATP synthesis, thereby impairing the function of the Na⁺/K⁺ pump and disrupting ion homeostasis. Following this, in the ischemic penumbra region, impaired glutamate transport leads to an accumulation of glutamate, which excessively activates NMDA receptors and subsequently induces Ca^2+^ influx [581]. Notably, energy depletion in ischemic tissues becomes evident 3 h post‐TIA, glutamate excitotoxicity manifests between 3 and 6 h, and calcium homeostasis disruption occurs within a 24‐h period [12].

Additionally, reperfusion injury plays a pivotal role in the pathophysiological mechanisms following a TIA. It has been demonstrated that within minutes of blood flow restoration, the BM proteins in the vascular wall undergo hydrolysis due to the actions of MMP‐9 and MMP‐2, subsequently resulting in vasogenic brain edema [582, 583]. In the ischemic penumbra, there exists a certain endogenous protective mechanism. Following local ischemia, the expression levels of heat shock proteins rise, with HSP70, HSP27, HSC70, and HSP32 playing pivotal roles [584]. HSP70 serves as a biomarker for cell membrane damage, and its induction is associated with the activation of endogenous protective mechanisms, facilitating the refolding of proteins after reversible ischemia and metabolic stress. These mechanisms may be linked to the restoration of cognitive function in the brain following TIA [12].

Because of the brief ischemic period, TIA remains reversible since ion homeostasis and functional structures have not been entirely disrupted. However, if ischemia persists, it can lead to irreversible damage. Hence, TIA can be seen as an “early warning” sign for IS, indicating that the tissue is in a state close to the danger threshold [12].

## 5 Clinical Management of Stroke

As mentioned earlier, the pathophysiological processes after stroke differ among subtypes, thus requiring different clinical management strategies. This section elaborates on the clinical management strategies for each subtype. The focus of IS management is on rapid reperfusion therapy to the ischemic area, coupled with neuroprotection, long‐term rehabilitation, and functional regeneration therapy. HS management focuses on acute‐phase care, prioritizing blood pressure control, anticoagulation reversal, and surgical management. TIA emphasizes early risk assessment and aggressive secondary prevention to reduce the risk of IS.

### 5.1 Clinical Management of IS

**FIGURE 5:**
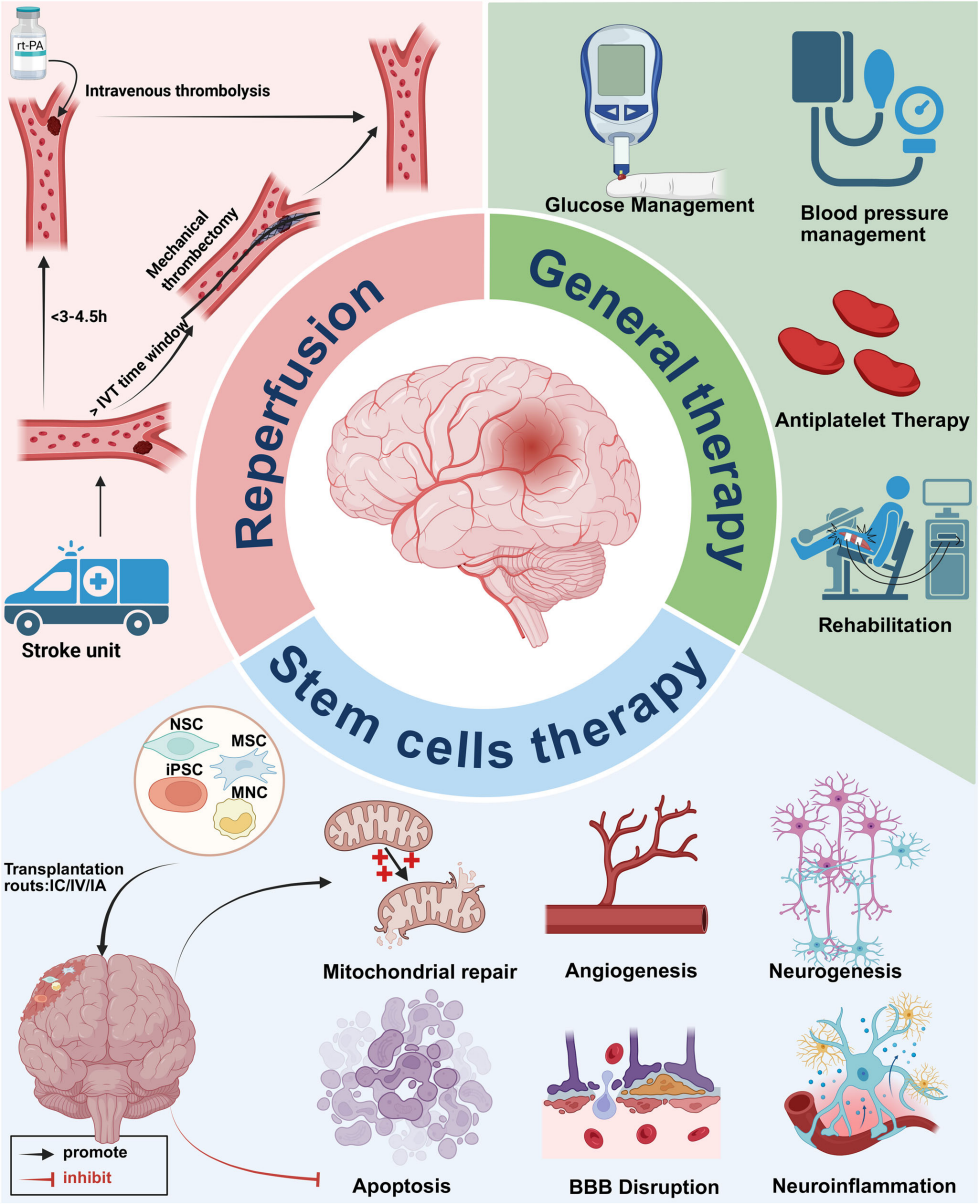
Overview of clinical management strategies for acute and rehabilitative phases of stroke. The clinical management of IS involves reperfusion therapy during the acute phase and general supportive therapy, including blood pressure management, glucose management, antiplatelet therapy, and rehabilitation therapy during the recovery phase. Stem cell therapy can improve the condition of IS patients through multiple mechanisms.

IS is a brain dysfunction caused by the obstruction of blood flow, typically resulting from an arterial blockage. Over the past few decades, research has focused on clinical management strategies aimed at treating its pathophysiology, rapidly restoring CBF, minimizing brain tissue damage, and lowering the risk of secondary complications. The following section discusses the current strategies for treating IS (Figure 5).

#### 5.1.1 Reperfusion

Reperfusion therapy is the most effective clinical treatment for IS, aiming to restore blood flow, rescue neurons, and improve prognosis [585]. The main methods are IVT and mechanical thrombectomy. IVT is a widely used method for patients within 3–4.5 h of symptom onset who have no contraindications. The most common drug is recombinant tPA (r‐tPA), which dissolves clots by converting plasminogen to plasmin [586]. r‐tPA significantly reduces disability and mortality, with early treatment being crucial for a good prognosis [587]. The main risk is intracranial bleeding, which necessitates careful patient screening [588, 589]. The effectiveness of r‐tPA diminishes after 4.5 h, and while some studies have explored extending the window to 6 h, the benefits are poor and the risk of bleeding increases [590, 591, 592, 593]. Therefore, when using IVT therapy for IS patients, individualized assessment of benefits and risks is necessary [594]. Mechanical Thrombectomy involves inserting a catheter through the femoral artery to remove a clot with a specialized device [595]. Techniques include stent thrombectomy (using a mesh to capture the clot), direct aspiration (suction), or a combination of both [596, 597]. Mechanical thrombectomy is suitable for patients with large artery occlusions, particularly in the internal carotid or middle cerebral arteries, who are ineligible for or have failed IVT, or who present outside the IVT time window (up to 24 h in some cases) [598, 599]. It significantly improves recanalization rates and reduces long‐term disability, but carries risks of bleeding, vascular injury, and new clot formation [600, 601, 602].

Mobile stroke units (MSUs) are ambulances equipped with CT scanners and a specialized medical team to provide prehospital care [594]. By bringing IVT to the patient, MSUs significantly shorten the time from stroke onset to treatment, allowing more patients to be treated within the “golden time window” [603, 604]. Studies have shown that MSUs improve patient outcomes and are cost‐effective, although their high cost and logistical challenges limit their widespread adoption [605, 606, 607, 608].

#### 5.1.2 Blood Pressure Management

Elevated blood pressure is common in acute IS patients. The optimal blood pressure target is a complex issue, as both very high and very low blood pressure can worsen outcomes [609, 610]. Current guidelines recommend allowing for some hypertension in the acute phase to maintain cerebral perfusion in the ischemic penumbra [611]. For patients receiving reperfusion therapy, the goal is to keep blood pressure below 185/110 mmHg before treatment and below 180/105 mmHg afterward [602]. However, there is no clear evidence to support aggressive blood pressure lowering, and a cautious, individualized approach is recommended [612, 613, 614, 615].

#### 5.1.3 Glucose Management

Hyperglycemia is a common complication in stroke patients that can aggravate brain damage, increase infarct volume, and worsen prognosis [616, 617, 618]. Most guidelines recommend moderate blood glucose control (140–180 mg/dL) to avoid both hyperglycemia and the risk of hypoglycemia [619]. Intensive glucose control (<110 mg/dL) has not shown clear benefits and may increase the risk of hypoglycemia [620].

#### 5.1.4 Antiplatelet Therapy

Antiplatelet therapy is a core strategy for secondary stroke prevention. Aspirin, clopidogrel, and ticagrelor are the most common drugs [621]. For patients with mild stroke or a TIA, early dual antiplatelet therapy (DAPT) with aspirin plus a P2Y12 inhibitor (e.g., clopidogrel) for a short period (4–12 weeks) is more effective than monotherapy at reducing the risk of early stroke recurrence without a significant increase in major bleeding risk [622, 623]. Long‐term prevention typically involves aspirin or clopidogrel monotherapy [624, 625].

#### 5.1.5 Rehabilitation

Rehabilitation is a gradual, goal‐oriented process that helps patients achieve the highest level of physical, cognitive, and functional independence [626, 627]. It involves physical, occupational, and speech therapy to improve mobility, communication, and daily living skills [628, 629]. Exercise rehabilitation promotes neuroplasticity by increasing neurotrophic factors like BDNF [630]. Techniques such as treadmill training, robot‐assisted gait training, neuromuscular electrical stimulation, music therapy, and functional task training are all used to improve recovery [631, 632, 633, 634]. However, very early, high‐intensity rehabilitation (within 24 h of a stroke) can be harmful, so a cautious approach is recommended [635].

#### 5.1.6 Limitations of Current Clinical Management

Despite significant advancements in reperfusion therapy, several limitations remain. Only a small percentage of patients can receive treatment within the narrow therapeutic time window (less than 10% for IVT and 7–15% for endovascular intervention) [15]. Reperfusion itself can cause new problems, including BBB injury and immune imbalance [16]. There are currently no clinically available drugs to protect brain tissue from ischemic injury or to promote a robust repair response [636]. Trials for neuroprotective drugs have shown limited success [637]. Therefore, the development of new diagnostic and therapeutic technologies is needed to advance recovery from IS [638].

### 5.2 Advances in the Application of Stem Cells in the Treatment of IS

In recent years, the rapid development of stem cell biology has brought new hope for the treatment of stroke. Various types of stem cells have been incorporated into preclinical and clinical studies, demonstrating their therapeutic effects on IS (Figure 5) [639]. Stem cell clinical trials for IS are categorized by the timing of treatment: acute (within 1 week), subacute (1 week to 6 months), and chronic (after 6 months). A detailed summary of these trials can be found in Table 1 which includes information on cell type, injection route, dosage, timing, reperfusion therapy, mechanism, and outcomes.

**TABLE 1:** Clinical research on stem cell therapy for IS.

References	Trial registration	Cell type	Route	Dosage of treatment	Timing of treatment	Intervention group (no reperfusion/tPA/MT/tPA+MT)	Control group (no reperfusion/tPA/MT/tPA+MT)	Pathway and mechanism of action	Assessment	Outcome
[640]	NCT03004976	Allogeneic UCBCs	IV	4.98–20 × 10^8^	3–9 days	10 Unclassified	0	Secretion of BDNF and VEGF to promote neuronal, vascular, and synaptic regeneration	mRS, NIHSS, AE	Well‐tolerated treatment with no adverse reactions and improvement in clinical scores
[641]	IRB105‐71‐A	Allogeneic UCBCs	IV	3–30 × 10^8^	3–10 days	1 (without t‐PA therapy)	0	Downregulation of TNF‐α, IL‐1β, IL‐2; secretion of BDNF and VEGF to promote synaptogenesis and neural plasticity	NIHSS, BI, MRI, Berg balance scale scores	No serious adverse reactions, with improvement in clinical scores, and one patient fully recovered 12 months after treatment
[642]	No ClinicalTrials.gov ID provided	UC‐MSCs	Catheter‐based delivery	2 × 10^7^	11–50 days	4/0/0/0	0	Secretion of VEGF, BDNF, and NGF to promote neural repair with low immunogenicity	mRS	Feasible and safe, with improvement in neurological function in IS patients
[643]	NCT0053519	CD34+ stem cells	IA	1.2–2.79 × 10^6^	7 days	3/0/2/0	0	Enhancement of angiogenesis and neuroregeneration via immunomodulation and paracrine effects	mRS, NIHSS	The clinical function is improved, the lesion volume is reduced, and the treatment process is safe.
[644]	NCT02813512	Autologous adipose‐derived stem cells	IC	0.9–1.1 × 10^8^	1.6–6 years	3/0/0/0	0	Secretion of VEGF, BDNF, NT‐3, and NT‐4 and inhibition of TNF‐α, IL‐6, and IFN‐γ to exert antiapoptotic, anti‐inflammatory, neuroprotective, and proangiogenic effects	NIHSS, BI, Berg balance scale, FMA	Clinical symptoms improved, no significant adverse reactions
[645]	NCT01468064	Autologous EPCs	IV	1.5 × 10^8^	1 months	5/0/0/0	6/0/0/0	EPCs secrete VEGF, BDNF, and SDF‐1α to inhibit inflammation and enhance neuroprotection and angiogenesis.	mRS, NIHSS, BI	Safe, but no significant difference in improvement of neurological function
[646]	NCT00950521	CD34(+)PBSCs	IC	3–8 × 10^6^	6 months–5 years	15/0/0/0	15/0/0/0	PBSCs act via paracrine secretion of neurotrophic and chemotactic factors, facilitating CST repair and neuroplasticity.	mRS, NIHSS, MRI, ESS, EMS	Safe and feasible, effectively improving patient function
[647]	BB IND 13775	Autologous BMMNCs	IV	6 × 10^8^	1–3 days	2/1/7/1	0	Inhibition of inflammation, secretion of protective cytokines, and promotion of angiogenesis and neuroregeneration	mRS, NIHSS, BI	Safe, no adverse reactions occurred, and the patient's clinical functional score improved.
[648]	NCT00859014	Autologous BMMNCs	IV	6 × 10^8^	1–3 days	9/16/0/0	122/63/0/0	Upregulate anti‐inflammatory pathways, enhance angiogenesis, neurogenesis, and synaptogenesis	mRS, NIHSS, BI, SAES	Safe and feasible, with improvement in clinical functional scores
[649]	No ClinicalTrials ID provided	Autologous BMMNCs	IA	5.1–60 × 10^7^	3–10 days	20/0/0/0	0	Regulation of splenic cell phenotypes, inhibition of central and peripheral inflammatory cytokines, and promotion of angiogenesis and tissue repair in ischemic areas	mRS, NIHSS, MRI, CT	Safe, with significant clinical functional improvement in patients
[650]	NCT02178657	Autologous BMMNCs	IA	1.2–3 × 10^8^	<7 days	Low‐dose 2/3/6/7 High‐dose 2/2/7/7	5/3/14/14	Secretion of VEGF, IL‐10, and GM‐CSF to promote repair, regulate microglial activity, and suppress proinflammatory responses	mRS, NIHSS, BI, MRI	Safe, but no significant improvement in patient function
[651]	NCT00761982	Autologous BMMNCs	IA	1.59 × 10^8^	5–9 days	6/4/0/0	8/2/0/0	Secretion of β‐NGF to promote neuroprotection, angiogenesis, axonal regeneration, and synaptic remodeling	mRS, NIHSS, BI	Safe, with a positive correlation between the number of cell infusions and BI improvement
[652]	NCT01028794	Autologous BMMNCs	IV	2.5–3.4 × 10^8^	7–10 days	8/4/0/0	0	Activation of cerebral microvascular systems and secretion of NGF, VEGF, and BDNF to enhance endogenous neurogenesis	mRS, NIHSS, BI, PET	Safe and feasible, cell therapy is beneficial for neurological recovery and improves cerebral blood flow and metabolism.
[653]	NCT01678534	Allogeneic AD‐MSCs	IV	6 × 10^7^	<2 weeks	7/1/0/1	4/1/4/1	Paracrine activity: secretion of VEGF, BDNF, reduction of inflammation, and delivery of repair signals via EVs	mRS, NIHSS	Safe, but the improvement in treatment outcomes is not statistically significant.
[654]	NCT01716481	Autologous MSCs	IV	6 × 10^7^	90 days	28/11/0/0	10/5/0/0	Enhanced neurogenesis, angiogenesis, and functional recovery, likely via NGF, VEGF, and BDNF signaling	mRS	Safe, with no improvement in mRS score, but significant improvement in lower limb motor function
[655]	JapicCTI‐184103	CL2020, an allogenic muse cell‐based product	IV	15 mL CL20208 1.5 × 10^7^	14–28 days	10/7/7/1	5/4/4/1	Recognition of damaged cell‐released S1P and differentiation into neurons and glial cells within ischemic regions	mRS, NIHSS	No major safety issues, with improvement in upper limb and overall function
[656]	NCT00875654	MSCs	IV	Low‐dose group: 1 × 10^6^ High‐dose group: 3 × 10^6^	1–2 months	12/4/0/0	7/8/0/0	MSCs may act during the subacute phase of stroke through inflammation modulation that promotes angiogenesis and neurogenesis, supporting brain repair.	mRS, NIHSS, BI, fMRI	Safe and feasible, motor function recovery
[657]	No ClinicalTrials.gov ID provided	Autologous BMMNCs	IA	5 × 10^8^	8–15 days	9/1/0/0	10/0/0/0	Stem cells produce benefit via reduced apoptosis and inflammation, promotion of angiogenesis and neurogenesis, plasticity, and formation of neural circuitry.	mRS, NIHSS, BI	Safe, with improvement in clinical outcomes
[658]	No ClinicalTrials.gov ID provided	MSCs/MSCs + NSPCs	IV/IV + IC	3 × 10^7^ MSCs/3 × 10^8^ MSCs + 3.6 × 10^8^ NSPCs	1 week–2 years	6/0/0/0	0	Secretion of NGF, BDNF, and VEGF to promote angiogenesis and synergistically repair neural networks	mRS, NIHSS, BI	Improvement in neurological function, disability, and daily living ability; most common side effect was mild fever
[659]	No ClinicalTrials.gov ID provided	MSCs	IV	5 × 10^7^	4 weeks	10/6/0/0	24/12/0/0	MSCs enhance neurogenesis and synaptogenesis.	mRS, NIHSS	Safe, with stroke recovery improving according to specific patient characteristics
[660]	NCT01716481	MSCs	IV	6 × 10^7^	36–74 days	39/0/0/0	15/0/0/0	MSCs significantly increase circulating EVs, especially miRNA‐rich and neurogenesis‐associated vesicles, which correlate with clinical recovery.	MRI, FMA	Improvement in motor function
[661]	NCT01716481	Autologous MSCs	IV	6 × 10^7^	3–45 days	31/0/0/0	13/0/0/0	MSC therapy might reduce degeneration of damaged tracts and facilitate plasticity of interhemispheric and motor‐related neural networks.	fMRI, FMA, DTI	Enhanced neural network remodeling, promoting poststroke motor recovery
[662]	No ClinicalTrials.gov ID provided	Autologous MSCs	IV	5 × 10^7^	15–92 days	4/1/0/0	20/5/0/0	MSCs secrete NGF, BDNF, VEGF, and so on, to enhance endogenous neural plasticity.	mRS, NIHSS, BI, DWI	Improved prognosis, with continued improvement in functional scores; no related adverse reactions
[663]	NCT01501773	Autologous BMMNCs	IV	1.9–18.5 × 10^7^	2–4 weeks	11/0/0/0	0	The major mechanism of MNCs via the release of growth factors, angiogenesis factors, and antiapoptotic factors	mRS, NIHSS, BI, MRI, PET	Safe, with seven patients having good clinical prognosis
[664]	NCT00473057	Autologous BMMNCs	IA	1–5 × 10^8^	2–3 months	6/0/0/0	0	By trophic, anti‐inflammatory and immunomodulatory effects	mRS, NIHSS, BI, SPECT	Safe, with no adverse reactions, no patient scored worst on the BI, mRS, or NIHSS tests
[665]	UMIN000026130	Autologous MSCs	IA	2–5 × 10^7^	47–64 days	3/2/1/1	0	BMSCs migrate from the injection site to the infarct border, releasing VEGF, BDNF, and NG to promote neuroprotection and anti‐inflammation.	mRS, NIHSS, BI, DWI	Safe, with good tolerance and continuous improvement in neurological and functional scores
[666]	CTRI/2008/091/000046	Autologous BMMNCs	IV	54.6 × 10^6^	3–4 months	12/0/0/0	12/0/0/0	Secretion of VEGF and BDNF to activate endogenous neural repair and facilitate relearning via modulation of the host environment	fMRI, DTI	Safe, with an increase in the number of Brodmann area cluster activations after stem cell infusion, indicating neuroplasticity and aiding CIS function recovery
[667]	NCT00473057	Autologous BMMNCs	IA	1–5 × 10^8^	2–3 months	3/2/0/1	0	Release of VEGF, BDNF, NGF, and IL‐10 to promote angiogenesis, neuroprotection, and anti‐inflammatory effects	mRS, NIHSS, BI, SPECT	Safe, with no deterioration in patient neurological function scores
[668]	NCT01461720	Autologous BMMNCs	IV	1.2 × 10^8^	1 week–2 months	9/0/0/0	8/0/0/0	MSCs change the poststroke brain microenvironment through anti‐inflammatory and neurotrophic effects.	mRS, NIHSS, BI, MRI	Safe, with improvement in infarct volume in patients
[669]	No ClinicalTrials.gov ID provided	Autologous BMMNCs	IV	0.8–1.5 × 10^8^	1–4 months	12/0/0/0	0	These cells may act through anti‐inflammatory and trophic mechanisms.	NIHSS, MRI	Safe and feasible, with a 20% reduction in average lesion volume as assessed by MRI
[671]	NCT01501773	Autologous BMSCs	IV	2.8075 × 10^8^	18.5 days	60/0/0/0	60/0/0/0	Not specified	mRS, NIHSS, BI	Safe, but no improvement in neurological function
[672]	No ClinicalTrials.gov ID provided	Autologous BMMNC	IA	6 × 10^7^	2–4 weeks	21/0/0/0	18/0/0/0	Infused stem cells were not only preferentially homing in the infarcted brain but also secrete neurotrophic growth factors and help endogenous neurogenesis after stroke.	mRS,, NIHSS, BI	Safe with minimal harm, but no significant improvement in patient condition from stem cell therapy
[673]	NCT01273337	Autologous BMMNCs	IA	1.8 × 10^8^	2–3 weeks	14/1/5/9	8/2/4/5	Cells exert their effects through angiogenic, neurotrophic and antiapoptotic cytokines.	mRS, BI, MRI	Safe, but the improvement in treatment outcomes is not statistically significant
[674]	NCT01297413	Allogeneic MSCs	IV	0.5–1.5 × 10^6^	>6 months	36/0/0/0	0	MSC may act via multiple mechanisms including release of trophic factors, immune modulation, and systemic trafficking to peri‐infarct regions.	NIHSS, BI	Safe, and can improve patient behavioral function
[675]	No ClinicalTrials.gov ID provided	Autologous BMSCs or BMMNCs	IV	5–6 × 10^7^	3 months–2 years	MNC: 14/0/0/0 MSC: 6/0/0/0	20/0/0/0	Secretion of VEGF, BDNF, NT‐3, and NT‐4 and inhibition of TNF‐α, IL‐6, and IFN‐γ to exert antiapoptotic, anti‐inflammatory, neuroprotective, and proangiogenic effects.	BI, MRI, Berg balance scale, FMA	Safe, with improvement in BI score and increased Brodmann area cluster activation, indicating neuroplasticity of stem cell therapy
[676]	CTRI/2014/09/005028	Autologous BMMNCs	IV	5 × 10^7^	6–15 months	10/0/0/0	10/0/0/0	BM‐MNCs secrete VEGF, BDNF, GDNF, IGF‐1, and NT‐3, exerting neurotrophic and proangiogenic effects.	BI, Berg balance scale, FMA	No serious adverse events, with improvement in BI score
[677]	No ClinicalTrials.gov ID provided	Autologous BMMNCs	Intrathecal	6 × 10^7^	4 months–12 years	5/0/0/0	0	Release of VEGF, BDNF, and SDF‐1α activates PI3K/AKT and MAPK pathways to promote neuroprotection.	FIM, PETCT	Safe, with improvement in patient condition, patients receiving cell therapy within 2 years after stroke showed better changes
[678]	CTRI/2011/08/002677	Autologous BMSCs	IV	5–6 × 10^7^	8–12 months	6/0/0/0	6/0/0/0	MSCs secrete VEGF, BDNF, NT‐3, NT‐4, GDNF, and SDF‐1α to promote neuroprotection and angiogenesis, with no endotoxin or tumor formation.	Berg balance scale, FMA	No cell‐related side effects, with statistically significant improvement in BI
[679]	No ClinicalTrials.gov ID provided	Autologous BMSCs	IC	1.4–5.5 × 10^7^	1 year	5/0/0/0	0	BMSC secreting cytokines and neurotrophic factors	NIHSS, BI	BMSCs can be safely transplanted into the patient's brain, with no complications, some evaluations showed a significant improvement.
[682]	EudraCT:2012‐003482‐18	Human neural stem cell line CTX0E03	IC	2 × 10^7^	2 months–1 year	23/0/0/0	0	CTX0E03 cells modulate the local inflammatory response and promote angiogenesis and neurogenesis in host tissue.	NIHSS, Berg balance scale, FMA	Safe, with improvement in upper limb function in patients
[740]	NCT02961504	Multistem	IV	1.2 × 10^9^	18–36 h	48/32/24/0	50/40/12	Immunomodulation and promotion of tissue regeneration	mRS, NIHSS, BI	Safe but does not improve short‐term outcomes
[680, 681]	NCT01287936	Modified bone marrow‐derived mesenchymal stem cells (SB623)	IC	1.5 × 10^8^	7–36 months	18/0/0/0	0	SB623 cells release VEGF, BDNF, GDNF, and SDF‐1α to initiate anti‐inflammatory effects and enhance synaptic plasticity through multiple mechanisms.	mRS, NIHSS, MRI, Berg balance scale, FMA, ESS	Safe, with improvement in clinical function scores after implantation

Abbreviations: AD‐MSC, adipose tissue‐derived mesenchymal stem cells; AE, adverse events; AKT, protein kinase B; BDNF, brain‐derived neurotrophic factor; BI, Barthel index; BMMNC, bone marrow mononuclear cells; BMSC, bone marrow‐derived mesenchymal stem cells; DTI, diffusion tensor imaging; DWI, diffusion weighted imaging; EMS, ESS motor subscale; EPCS, endothelial progenitor cells; ESS, European stroke scale; EV, extracellular vesicles; FMA, Fugl‐Meyer assessment; IA, intra‐arterial; IC, intracerebral; IV, intravenous; MRI, magnetic resonance imaging; mRS, modified Rankin scale; MSC, mesenchymal stem cells; MT, mechanical thrombectomy; NGF, nerve growth factor; NIHSS, National Institutes of Health Stroke Scale; NSC, neural stem cells; NSPC, neural stem/progenitor cells; NT, neurotrophin; PBSC, peripheral blood stem cells; PET, positron emission tomography; PI3K, phosphoinositide 3‐kinase; S1P, sphingosine‐1‐phosphate; SDF‐1, stromal cell‐derived factor 1; SPECT, single photon emission computed tomography; UCBC, umbilical cord blood stem cells; UC‐MSCS, umbilical cord blood stem cells; VEGF, vascular endothelial growth factor.

#### 5.2.1 Stem Cell Types

Preclinical trials demonstrate that various types, including mononuclear cells (MNCs), mesenchymal stem cells (MSCs), and neural stem cells (NSCs), are effective therapeutic candidates. In clinical trials, autologous bone marrow MNCs (BMMNCs) are the most frequent choice, followed by autologous MSCs; other sources include CD34+ cells, UCBCs, and ADSCs [640‐646]. For immediate treatment, BMMNCs or allogeneic cells are preferred due to the time required for in vitro expansion [640, 641, 647, 648, 649, 650, 651, 652, 653], with MSCs also commonly used in later stages [642, 654, 655, 656, 657, 658, 659, 660, 661, 662, 663, 664, 665, 666, 667, 668, 669]. The characteristics of different stem cell treatments are summarized in Table 2

**TABLE 2:** A Summary of various stem cell.

Cell types	Route	Doses	Timing	Advantages	Disadvantages
MSCs	IV is the most common, followed by IC, IA, and so on.	10^6^–10^8^ cells	Animal experiment: 0–24 h Clinical trial: 3–10 days	Promotes nerve repair, angiogenesis, anti‐inflammation, immune regulation, and inhibits apoptosis, involving signaling pathways such as PI3K/AKT, STAT3, mTOR, MAPK, BDNF/TrkB, and so on	Headache, nausea, seizure
NSCs	IC is predominant, with a small portion being IV, and IA is rare.	10^5^–10^6^ cells	Mainly 0–3 days, some clinical trials being delayed to 7 days or even 60 days	Promoting nerve regeneration, angiogenesis, immune regulation, and so on, mainly involving signaling pathways such as PI3K/AKT, mTOR, STAT3, MAPK, and so on	Fever, infection, headache
iPSCs	IC is the most common, while IV has higher safety but lower colonization rate.	1.2 × 10^5^ cells	0–24 h after stroke	Promote synaptic plasticity, angiogenesis, regulate apoptosis, and neuroinflammation	Fever, headache, bleeding, potential tumorigenic risk
HSCs	IV	10^5^–10^7^ cells	0–24 h after stroke	Secreting neurotrophic factors, anti‐inflammatory, regulating microvascular environment	Fever, hepatic damage, seizure
BMMNC	IV	10⁷–10⁸ cells	3–14 days after stroke	It has immunomodulatory, anti‐inflammatory, and neuro‐ and angiogenesis‐promoting effects, and is highly safe and accessible, making it suitable for clinical settings.	Risk of thrombosis, low effective content (MSCs), insufficient therapeutic effect

*Abbreviations*: AKT, protein kinase B; BDNF, brain‐derived neurotrophic factor; BMMNC, bone marrow mononuclear cells; HSCS, hematopoietic stem cell; IA, intra‐arterial; IC, intracerebral; IPSCS, induced pluripotent stem cells; IV, intravenous; MAPK, mitogen‐activated protein kinase; MSCS, mesenchymal stem cells; MTOR, mammalian target of rapamycin; NSCS, neural stem cells; PI3K, phosphoinositide 3‐kinase; STAT3, signal transducer and activator of transcription 3; TRKB, tropomyosin receptor kinase B.

#### 5.2.2 Timing, Dosage, and Administration Route of Stem Cell Therapy

The timing of stem cell injection is a critical factor for successful outcomes. Early intervention is widely considered the optimal period, as it aims to reduce inflammation and BBB disruption, and clinical evidence generally links earlier therapy to better neuroprotection and crucial support for tissue reconstruction [640, 641, 643, 647, 648, 649, 651, 656, 670]. In subacute phase, while many studies show favorable results [642, 654, 655, 656, 657, 658, 659, 660, 661, 662, 663, 664, 665, 666, 667, 668, 669], some large trials have not found a significant benefit [671, 672, 673]. In the chronic phase where the brain's self‐repair capacity declines, stem cell therapy can still improve function and quality of life by enhancing neuroplasticity [644, 646, 674, 675, 676, 677, 678, 679, 680, 681, 682]. Table 2 shows the optimal dosage for various types of stem cells. Higher stem cell doses generally correlate with improved outcomes, as evidenced by studies showing that high‐dose groups achieved greater motor function recovery and a positive correlation with improvements in the Barthel index [651, 656].

The delivery route of stem cells significantly impacts their efficacy and safety. Table 2 shows the optimal delivery route for stem cells. Intracerebral (IC) injection delivers cells directly to the lesion for high local concentration, ideal for NSC implantation and chronic‐phase treatment [683, 684]. However, its clinical use is limited by risks including invasiveness, hemorrhage, and tumorigenicity [685, 686, 687]. IV injection is the most common and safest clinical route [688], primarily suited for systemic effects like anti‐inflammation and the release of neurotrophic factors due to significant cell filtration by peripheral organs [689, 690, 691]. In contrast, intra‐arterial (IA) injection bypasses filtration to achieve higher local concentrations and potentially better short‐term outcomes but carries the serious risk of microvascular occlusion and increased injury [692, 693]. The optimal stem cell administration route depends on the stroke stage and cell type. Current evidence suggests IV and IA routes are better for acute/subacute phases, whereas IC is likely better for chronic stroke [643, 657, 681, 684].

#### 5.2.3 Mechanism of Stem Cell Therapy for IS

Preclinical studies on stem cells have shown that the mechanism of stem cell therapy for IS involves several key processes, including cell migration, promotion of neurotrophic factor secretion, inhibition of apoptosis and inflammation, and the promotion of angiogenesis and neural circuit reconstruction [694].

Both endogenous and transplanted stem cells migrate to the ischemic area via the interaction between their chemokine receptors (e.g., CXCR4, CCR2) and chemokines (e.g., SDF‐1, CCL2) secreted by activated neurons and glial cells [434, 695, 696]. Specifically, chemokines like SDF‐1 secreted by microglia and astrocytes in the infarct guide the migration of stem cells, such as hBM‐MSCs and iPSC‐derived NSCs, to the injury site [697, 698, 699].

Although spontaneous neuroplasticity promotes functional recovery after IS, this natural process is severely limited by the hostile environment and glial scar formation [424, 441, 442]. Stem cells counteract these limitations by increasing endogenous NSCs, promoting their neuronal differentiation, and reducing glial scars [700]. Transplanted cells promote the re‐establishment of neural circuits and function by maturing into functional neurons and forming synaptic connections with host neurons [701, 702, 703]. Furthermore, stem cells exert a powerful bystander effect by migrating to the injury site and releasing neurotrophic factors (like BDNF, GDNF, VEGF) and growth factors, which enhance neural plasticity, promote axonal regeneration, support cell survival, and induce angiogenesis and neuroprotection [704, 705, 706].

Mitochondrial dysfunction, a hallmark of IS, causes reduced ATP production, increased ROS, and activated apoptosis [707, 708]. MSCs counteract this by transferring mitochondria to dysfunctional cells via tunneling nanotubes, thereby repairing mitochondrial function and reducing cellular damage [709]. This transfer, which is regulated by specific molecular loops (e.g., SIRT‐1/RHOT‐1/PGC‐1α), enhances aerobic respiration in ischemic ECs, promoting their survival, angiogenesis, and cerebral microcirculation [710, 711].

Following IS, both the intrinsic (mitochondrial) and extrinsic (death receptor) apoptotic pathways are activated, which stem cell transplantation effectively inhibits through various mechanisms [712, 713]. MSCs suppress apoptosis by activating antiapoptotic factors like Bcl‐2 [714], inhibiting proapoptotic signals [715], and regulating pathways such as SIRT‐1, AKT, and NF‐κB [716]. Additionally, stem cell therapies reduce apoptosis by improving mitochondrial function; for example, BM‐MSC exosomes target Drp1 and activate the PINK1/Parkin pathway [717, 718].

Angiogenesis is a critical protective mechanism for neuroregeneration after stroke, and stem cell therapies promote it primarily through the bystander effect by secreting growth factors (e.g., VEGF, BDNF, IGF‐1, Ang‐1) [719, 720, 721, 722, 723, 724]. Specifically, NSCs enhance angiogenesis and tight junction proteins by activating the Ang1/Tie2, VEGF/VEGFR2, and JAK2/STAT3 signaling pathways [725, 726]. Furthermore, BM‐MSC exosomes promote angiogenesis by delivering specific miRNAs (miR‐21‐5p, miR‐486) that upregulate key factors (VEGF, VEGFR2, Ang‐1) and downregulate PTEN [727, 728].

Stem cells (e.g., BM‐MSCs, NSCs) significantly improve BBB integrity following IS by downregulating inflammatory factors (like MMP‐9) and adhesion molecules (like ICAM‐1), major contributors to BBB breakdown [729, 730]. Stem cell‐derived EVs protect the BBB by mitigating tight junction protein degradation through antagonizing the endocytic effects of caveolin‐1 [390, 731]. Furthermore, EVs and exosomes deliver bioactive factors (e.g., AKT1, CALM) that activate pathways (e.g., eNOS, Sirt1) to restore BBB function [732].

Clinical findings on stem cell therapy mechanisms largely align with preclinical research, confirming that primary therapeutic effects are mediated by paracrine signaling, not direct cell replacement. Stem cells enhance neural network remodeling and corticospinal tract repair by secreting neurotrophic factors such as BDNF, NGF, and NT‐3 [646, 676, 678], and promote new blood vessel formation and neurovascular unit reconstruction via angiogenic factors like VEGF and SDF‐1α. Furthermore, similar to preclinical findings, stem cell‐derived EVs (carrying signaling miRNAs and proteins) are being investigated clinically for their role in indirectly promoting host cell repair, reducing apoptosis, and enhancing neural plasticity [660].

#### 5.2.4 Current Status and Remaining Challenges of Stem Cell Therapy for IS

Stem cell therapy for IS has been extensively explored in both preclinical and clinical settings. Commonly used cell types include MSCs, NSCs, iPSCs, and BMMNCs. These cells primarily work through paracrine mechanisms, including neurotrophic, immunomodulatory, and antiapoptotic effects. The choice of administration route varies, with MSCs often delivered via IV and NSCs via IC.

Clinical trials suggest that stem cell therapy appears independent of acute reperfusion treatments (such as r‐tPA or mechanical thrombectomy) and can serve as a complementary approach. While most trials excluded patients receiving reperfusion, in those that included them, stem cell transplantation consistently promoted significant functional recovery beyond the benefits achieved by early reperfusion alone.

Despite its promise, stem cell therapy for IS faces significant challenges, including mixed clinical outcomes where some studies report no significant benefits [645, 651, 673, 733]. A major hurdle is the hostile ischemic microenvironment (hypoxia and inflammation), which severely compromises stem cell survival and leads to differentiation imbalance, limiting functional neuron formation (only 3–20% differentiate successfully in situ) [734, 735]. Furthermore, while generally safe, adverse events such as fever, headache, recurrent stroke, and local reactions have been reported. A rare but critical barrier to widespread clinical application is the potential for malignant transformation and tumor formation [684, 736, 737].

Current research on stem cell therapy for stroke faces significant limitations. The specific molecular mechanisms (e.g., paracrine signaling, neural network reconstruction) are not fully understood, requiring further multiomics exploration [687]. There is currently no consensus on the optimal cell type, source, dosage, administration time, and route [738]. Furthermore, the high costs of cell production, quality control, transportation, and storage have severely hampered the widespread use of this therapy [739].

### 5.3 Clinical Management of HS

Currently, the management of HS in the acute phase mainly focuses on blood pressure management and anticoagulant reversal (if necessary) to prevent hematoma expansion (HE). During the acute phase, it is also necessary to prevent and manage HS complications such as increased ICP, hyperglycemia, and fever to prevent disease progression. Surgical treatment may be considered for patients with severe conditions.

#### 5.3.1 Blood Pressure Management

Acute hypertensive response frequently occurs in patients with HS, with most experiencing persistently elevated blood pressure upon presentation and during the initial hours of hospitalization. Persistently high blood pressure is linked to adverse outcomes during hospitalization, including mortality, dependency, and clinical deterioration [741]. Early blood pressure control is vital for preventing adverse events, particularly HE. According to the 2015 AHA/American Stroke Association guidelines for HS management, safely lowering SBP to below 140 mmHg in the acute phase can significantly enhance patient prognosis [742]. Recent research on the correlation between blood pressure and prognosis following cerebral hemorrhage has primarily centered on the extent of blood pressure reduction and blood pressure variability. Studies indicate that attaining early and stable SBP, lowering the mean SBP to 147 mmHg, and regulating SBP variability within 14 mmHg can markedly enhance patient outcomes [743]. Future studies should delve deeper into determining the optimal SBP target and management strategies postcerebral hemorrhage. Clinically, it is imperative to tailor the SBP target according to the initial blood pressure readings and hematoma characteristics, while also ensuring stable blood pressure maintenance during acute phases [11].

#### 5.3.2 Anticoagulant and Antiplatelet Reversal

Over the past few decades, due to the increased use of these anticoagulant drugs, the incidence rate of oral anticoagulant‐associated HS has risen, accounting for nearly a quarter of all HS cases [744]. During the acute phase, prothrombin complex concentrates combined with vitamin K should be administered promptly to reverse the effects of vitamin K antagonists. This approach can more effectively normalize the international normalized ratio, prevent HE, and improve patient prognosis [745].

Currently, for patients with antiplatelet‐related cerebral hemorrhage, platelet transfusion is not recommended without undergoing neurosurgical procedures [746]. Clinical trials have demonstrated that, compared with patients receiving standard medical care, those who underwent platelet transfusions faced an elevated risk of mortality or dependency, along with a higher incidence of severe adverse events [747]. Desmopressin acetate, which stabilizes platelet function, is now recommended for consideration in the treatment of patients with antiplatelet‐related cerebral hemorrhage [746].

#### 5.3.3 Complication Management

The standard clinical management for increased ICP involves elevating the head of the bed to a 30° angle, administering sedation, implementing transient hyperventilation, and utilizing hyperosmolar agents like mannitol or hypertonic saline. A meta‐analysis encompassing five RCTs on drugs for reducing ICP revealed that hypertonic saline exhibited greater efficacy in treating elevated ICP [748]. Consequently, current guidelines advocate for the use of hypertonic saline over mannitol to lower ICP and prevent cerebral edema [749].

Fever represents a frequent complication following cerebral hemorrhage, typically resulting from intraventricular hemorrhage (IVH). Patients experiencing fever often exhibit unfavorable short‐ and long‐term prognoses [750]. According to a meta‐analysis, therapeutic hypothermia can suppress the multicellular death pathways induced by cerebral hemorrhage and mitigate hematoma enlargement [751]. Presently, the ASA guidelines for cerebral hemorrhage management advocate for antipyretics as the primary treatment option for fever in patients with cerebral hemorrhage [742].

Elevated blood glucose is a frequent occurrence following HS, and hyperglycemia is linked to a worse prognosis and a higher incidence of complications post‐HS [752]. Preventing hyperglycemia after HS contributes to a better prognosis and a reduction in complications such as HE, ICP, and seizures [753]. Consequently, the ASA guidelines advise monitoring blood glucose levels after HS to prevent acute hyperglycemia [742].

#### 5.3.4 Surgical Management

The amount of HS is strongly associated with mortality and unfavorable functional outcomes. HS results in blood extravasation into the brain parenchyma, triggering a mass effect. When the hemorrhage spills over into the ventricular system or compresses the cerebrospinal fluid (CSF) drainage pathways, it may cause hydrocephalus, leading to an elevation in ICP and compromised cerebral perfusion [11].

The incidence of IVH among patients with acute HS is roughly 40%, and it is significantly correlated with a poor prognosis [754]. An external ventricular drain (EVD) can mitigate elevated ICP, serve as a shunt for CSF drainage, alleviate obstructive or communicating hydrocephalus induced by IVH, and facilitate ICP monitoring [742]. Studies have shown that EVD can reduce mortality after HS and IVH, although the benefits on functional outcomes remain controversial [755, 756]. The European Stroke Organisation guidelines endorse the use of EVD in combination with intraventricular thrombolysis for the treatment of patients with HS and ventricular dilation [757]. The CLEAR‐III trial has demonstrated the safety and efficacy of EVD combined with r‐tPA in managing obstructive hydrocephalus caused by IVH [758].

Numerous studies have indicated that early minimally invasive evacuation of hematoma may enhance functional prognosis. The ENRICH trial demonstrated that, for patients suffering from spontaneous lobar HS, early minimally invasive hematoma evacuation significantly improved functional outcomes at 180 days (with a good outcome rate of 38.3% in the surgical group vs. 18.0% in the control group). However, it is important to note that 3.3% of patients in the surgical group experienced postoperative rebleeding and neurological deterioration [759]. According to the 2024 guidelines for HS management, compared with drug therapy alone, minimally invasive hematoma evacuation during the acute phase of HS can reduce mortality, but an individualized assessment is necessary [760].

In general, current evidence and guidelines endorse the potential advantages of EVD and minimally invasive hematoma evacuation for specific patients with HS. Further high‐quality randomized trials are required in the future to determine the optimal indications and technical specifics [11].

### 5.4 Clinical Management of TIA

At present, the crux of TIA management involves early risk assessment, antithrombotic and anticoagulant treatment during the acute phase, as well as the implementation of individualized secondary prevention strategies tailored to each patient, with the ultimate aim of reducing the risk of future strokes.

#### 5.4.1 Risk Assessment

TIA serves as a potent predictor for subsequent strokes. To prevent potential ISs that may occur later, clinicians ought to regard TIA as a medical emergency and conduct prompt and efficient diagnostic evaluations [761]. Risk stratification for TIA patients is beneficial in identifying those at high risk and providing guidance for their management [17]. At present, the utilization of validated risk scoring systems as a tool for risk stratification in TIA has garnered broader acceptance, with the ABCD2 score (age, blood pressure, clinical features, duration, and diabetes) being the most frequently employed scoring system. The ABCD2 score divides patients into three distinct risk categories: low (<4), moderate (4–5), and high (6–7) [762]. Research has indicated that the ABCD2 score exhibits high sensitivity but low specificity when differentiating between high‐risk (6–7 points) and low‐risk (0–3 points) patients. Seven days following a TIA, the risk of recurrent stroke stands at 7% in the high‐risk group, in contrast to 2.1% in the low‐risk group [763]. However, a low score does not necessarily mean minimal stroke risk, with about 20% of patients with an ABCD2 score below 4 having a 3‐month stroke risk comparable to those with a score above 4 [764].

Regardless of the ABCD2 score, it is crucial to conduct early noninvasive imaging through methods such as CTA, MRA, or neurovascular ultrasound (namely, extracranial carotid/vertebral color Doppler ultrasound and transcranial Doppler ultrasound) to guide prompt treatment [17]. Currently, the TIA risk scoring system has gradually evolved to incorporate radiological data and a history of recurrent TIA (defined as ≥2 TIA episodes within the past 7 days), aiming to better identify high‐risk patients. The ABCD3‐I (d, c/i) score builds upon the ABCD2 by adding assessments such as recurrent TIA history, MRI findings, and carotid and intracranial stenosis [765].

#### 5.4.2 Antiplatelet and Anticoagulant Therapy

Early antithrombotic treatment can relatively reduce the risk of stroke in TIA patients by about 80% [766]. Consequently, all suspected TIA patients without known contraindications should undergo antithrombotic therapy [623]. TIA patients taking 50–325 mg of aspirin daily can lower their risk of subsequent stroke [767]. For patients with high‐risk TIA (e.g., those with an ABCD score ≥4), DAPT (such as a combination of clopidogrel and aspirin) should be initiated within 48 h of symptom onset to mitigate the risk of IS or other vascular events within 90 days [768, 769].

For TIA patients with concomitant AF, therapeutic anticoagulant therapy can effectively lower the risk of stroke in AF patients, and anticoagulation therapy should be initiated to prevent systemic embolism [17, 770]. Research indicates that, among TIA patients with AF, warfarin can reduce the RR of post‐TIA stroke by approximately 60–70% compared with those who did not receive antithrombotic treatment [771]. Although the benefits of therapeutic anticoagulant therapy generally outweigh the risk of bleeding, it is still essential to evaluate the bleeding risk before commencing anticoagulation [772, 773].

#### 5.4.3 Secondary Prevention and Chronic Disease Management

Statins are effective in lowering LDL levels, thereby promoting plaque stabilization, improving endothelial dysfunction, and mitigating inflammatory responses [774]. Clinical evidence has demonstrated that statin therapy can reduce the recurrence rate of stroke by 16% in patients with IS, and it has been firmly established as a crucial component of secondary prevention following a TIA or stroke [775]. Current guidelines advocate for the use of statins in lipid management for TIA patients to minimize the risk of recurrence [776].

For TIA patients suffering from hypertension, blood pressure management should serve as a crucial element in both primary and secondary stroke prevention [777]. Guidelines recommend the use of antihypertensive medications for secondary prevention in the majority of patients [778]. Maintaining blood pressure below 130/80 mmHg can decrease the risk of stroke recurrence by 22% [17].

Among patients with TIA or IS, diabetes and hyperglycemia are linked to early neurological deterioration and stroke recurrence [779]. For TIA patients suffering from type 2 diabetes, blood glucose levels are mainly managed through lifestyle modifications, along with the use of metformin, sodium‐glucose cotransporter inhibitors, and GLP‐1 receptor agonists [17].

## 6 Conclusion and Prospects

Stroke remains a leading cause of death and disability worldwide. Developing more effective strategies for the prevention, treatment, and improvement of stroke is crucial to addressing the growing global burden of stroke.

Over the past few decades, stroke research has made significant progress in various areas, including pathogenesis, signaling pathways, therapeutic drugs, clinical trials, and poststroke rehabilitation. Nevertheless, significant challenges remain in clinical translation, with many preclinical findings failing to translate into clinical trials. A key reason for this is that animal studies of stroke typically use healthy adult animals as models, which may not accurately reflect the complexities of human stroke patients with risk factors such as diabetes, hypertension, hyperlipidemia, and aging. Future research should employ more appropriate animal models and modeling methods, such as stroke animal models targeting specific risk factors for stroke, to identify potential interactions between comorbidities and stroke treatment, in order to improve the safety and effectiveness of clinical translation outcomes. Brain organoids also provide an important new platform for translating preclinical research into clinical applications. Complementing two‐dimensional and animal models, these 3D models derived from human stem cells can more accurately simulate stroke‐related molecular events, overcoming the limitations of traditional two‐dimensional cultures in simulating complex brain structures. Addressing the challenge of animal models simulating complex patient conditions, brain organoids support patient‐specific modeling, enabling researchers to test treatments on patient‐derived cells and rapidly develop customized therapies for individual patients.

Current clinical management of stroke mainly focuses on acute‐phase reperfusion therapy, complication management, and rehabilitation during the recovery period. Although reperfusion therapy has significantly improved patient mortality and disability rates, its clinical application still has certain limitations. Regenerative therapies, especially stem cell therapy, offer promising treatment opportunities for stroke patients. However, stem cell therapy also faces numerous challenges. Stem cell transplantation results in low survival rates in the brain, difficulty in controlling differentiation, and the potential risks of malignant transformation and tumor formation. The number and scale of clinical trials for stem cell therapy are insufficient to provide definitive evidence of efficacy, and there is no consensus on the optimal cell type, dosage, timing, and route of administration. The high cost of stem cells and the potential ethical and legal issues they may raise are also significant obstacles limiting their clinical application.

Improving the survival rate and efficacy of transplanted cells is a future direction for regenerative medicine in stroke treatment. Specific functions of stem cells can be improved and their adaptation to ischemic and inflammatory environments achieved through gene modification and pretreatment to express beneficial genes. Furthermore, stem cells can be coadministered with drugs, hydrogels, or nanomaterials to enhance delivery and efficacy.

## Author Contributions

Yi Huang and Xiang Gao contributed to the conception and design of the study. He Ren, Yuchun Liu, Mingyue Zhao, Hangyu Shen, Xu Yan, and Sheng Nie organized the database. He Ren wrote the first draft of the manuscript. Yi Huang reviewed and edited. All authors read and approved the final manuscript.

## Funding

This study was supported by the grants from Zhejiang Provincial Natural Science Foundation (MS26H090028), Ningbo Top Medical and Health Research Program (2022020304), Ningbo Natural Science Foundation (2023J019), Chronic Disease Management Research Project of National Health Commission Capacity Building and Continuing Education Center (GWJJMB202510021112), and Ningbo Municipal Youth Science and Technology Innovation Leading Talent Project (2025QL015).

## Ethics Statement

The authors have nothing to report.

## Conflicts of Interest

The authors declare no conflicts of interest.

## Data Availability

The authors have nothing to report.

## References

[1] Collaborators GS . “Global, regional, and national burden of stroke, 1990–2016: A systematic analysis for the Global Burden of Disease Study 2016,” The Lancet Neurology 18, no. 5 (2019): 439–458. 30871944 10.1016/S1474-4422(19)30034-1 PMC6494974

[2] Collaborators GSRF . “Global, regional, and national burden of stroke and its risk factors, 1990–2021: A systematic analysis for the Global Burden of Disease Study 2021,” The Lancet Neurology 23, no. 10 (2024): 973–1003. 39304265 10.1016/S1474-4422(24)00369-7 PMC12254192

[3] A. I. Qureshi , A. D. Mendelow , and D. F. Hanley , “Intracerebral haemorrhage,” Lancet 373, no. 9675 (2009): 1632–1644. 19427958 10.1016/S0140-6736(09)60371-8 PMC3138486

[4] J. van Gijn , R. S. Kerr , and G. J. Rinkel , “Subarachnoid haemorrhage,” Lancet 369, no. 9558 (2007): 306–318. 17258671 10.1016/S0140-6736(07)60153-6

[5] S. Yusuf , S. Rangarajan , K. Teo , et al., “Cardiovascular risk and events in 17 low‐, middle‐, and high‐income countries,” The New England Journal of Medicine 371, no. 9 (2014): 818–827. 25162888 10.1056/NEJMoa1311890

[6] W. J. Tu and L. D. Wang , “China stroke surveillance report 2021,” Military Medical Research 10, no. 1 (2023): 33. 37468952 10.1186/s40779-023-00463-x PMC10355019

[7] D. O. Kleindorfer , A. Towfighi , S. Chaturvedi , et al., “2021 Guideline for the Prevention of Stroke in Patients With Stroke and Transient Ischemic Attack: A Guideline From the American Heart Association/American Stroke Association,” Stroke; A Journal of Cerebral Circulation 52, no. 7 (2021): e364–e467. 10.1161/STR.0000000000000375 34024117

[8] T. M. Woodruff , J. Thundyil , S. C. Tang , C. G. Sobey , and S. M. Taylor , “Arumugam TV. Pathophysiology, treatment, and animal and cellular models of human ischemic stroke,” Molecular Neurodegeneration 6, no. 1 (2011): 11. 21266064 10.1186/1750-1326-6-11 PMC3037909

[9] W. J. Powers , R. L. Grubb , and M. E. Raichle , “Physiological responses to focal cerebral ischemia in humans,” Annals of Neurology 16, no. 5 (1984): 546–552. 6334488 10.1002/ana.410160504

[10] L. R. Wechsler , D. Bates , P. Stroemer , Y. S. Andrews‐Zwilling , and I. Aizman , “Cell Therapy for Chronic Stroke,” Stroke; A Journal of Cerebral Circulation 49, no. 5 (2018): 1066–1074. 10.1161/STROKEAHA.117.018290 29669865

[11] J. Magid‐Bernstein , R. Girard , S. Polster , et al., “Cerebral Hemorrhage: Pathophysiology, Treatment, and Future Directions,” Circulation Research 130, no. 8 (2022): 1204–1229. 35420918 10.1161/CIRCRESAHA.121.319949 PMC10032582

[12] N. N. Belyavskii and S. A. Likhachev , “Pathophysiological aspects of transient ischemic attacks,” Neuroscience and Behavioral Physiology 41, no. 1 (2011): 28–34.

[13] C. Hui , P. Tadi , M. Z. Khan Suheb , and L. Patti , “Ischemic Stroke,” StatPearls (StatPearls Publishing, 2024). 29763173

[14] W. J. Powers , A. A. Rabinstein , T. Ackerson , et al., “2018 Guidelines for the Early Management of Patients With Acute Ischemic Stroke: A Guideline for Healthcare Professionals From the American Heart Association/American Stroke Association,” Stroke; A Journal of Cerebral Circulation 49, no. 3 (2018): e46–e110. 10.1161/STR.0000000000000158 29367334

[15] N. Henninger and M. Fisher , “Extending the Time Window for Endovascular and Pharmacological Reperfusion,” Translational Stroke Research 7, no. 4 (2016): 284–293. 26739964 10.1007/s12975-015-0444-4

[16] N. Yang , H. Lee , and C. Wu , “Intravenous thrombolysis for acute ischemic stroke: From alteplase to tenecteplase,” Brain Circulation 9, no. 2 (2023): 61–63. 37576574 10.4103/bc.bc_70_22 PMC10419734

[17] H. P. Amin , T. E. Madsen , D. M. Bravata , et al., “Diagnosis, Workup, Risk Reduction of Transient Ischemic Attack in the Emergency Department Setting: A Scientific Statement From the American Heart Association,” Stroke; A Journal of Cerebral Circulation 54, no. 3 (2023): e109–e121. 10.1161/STR.0000000000000418 36655570

[18] J.‐F. Zhou , Y. Xiong , X. Kang , et al., “Application of stem cells and exosomes in the treatment of intracerebral hemorrhage: An update,” Stem Cell Research & Therapy 13, no. 1 (2022): 281. 35765072 10.1186/s13287-022-02965-2 PMC9241288

[19] S. Zhou , Y. Yang , N. Qiu , and T. Yang , “The role of long non‐coding RNAs in angiogenesis in ischemic stroke: New perspectives based on advanced techniques in neuroscience,” Advanced Technology in Neuroscience 2, no. 2 (2025): 77–84.

[20] C. Stonesifer , S. Corey , S. Ghanekar , Z. Diamandis , S. A. Acosta , and C. V. Borlongan , “Stem cell therapy for abrogating stroke‐induced neuroinflammation and relevant secondary cell death mechanisms,” Progress in Neurobiology 158 (2017): 94–131. 28743464 10.1016/j.pneurobio.2017.07.004 PMC5671910

[21] M. A. Eckert , Q. Vu , K. Xie , et al., “Evidence for high translational potential of mesenchymal stromal cell therapy to improve recovery From ischemic stroke,” Journal of Cerebral Blood Flow and Metabolism: Official Journal of the International Society of Cerebral Blood Flow and Metabolism 33, no. 9 (2013): 1322–1334. 23756689 10.1038/jcbfm.2013.91 PMC3764389

[22] K.‐A. Kwak , H.‐B. Kwon , J. W. Lee , and Y.‐S. Park , “Current Perspectives Regarding Stem Cell‐based Therapy for Ischemic Stroke,” Current Pharmaceutical Design 24, no. 28 (2018): 3332–3340. 29866000 10.2174/1381612824666180604111806

[23] R. L. Sacco , S. E. Kasner , J. P. Broderick , et al., “An updated definition of stroke for the 21st century: A statement for healthcare professionals From the American Heart Association/American Stroke Association,” Stroke; A Journal of Cerebral Circulation 44, no. 7 (2013): 2064–2089. 10.1161/STR.0b013e318296aeca PMC11078537 23652265

[24] GBD 2016 Neurology Collaborators . Global, regional, and national burden of neurological disorders, 1990–2016: A systematic analysis for the Global Burden of Disease Study 2016. The Lancet Neurology 2019;18(5):459–480. 30879893 10.1016/S1474-4422(18)30499-X PMC6459001

[25] S. S. Martin , A. W. Aday , Z. I. Almarzooq , et al., “2024 Heart Disease and Stroke Statistics: A Report of US and Global Data From the American Heart Association,” Circulation 149, no. 8 (2024): e347–e913. 38264914 10.1161/CIR.0000000000001209 PMC12146881

[26] GBD 2019 Stroke Collaborators . Global, regional, and national burden of stroke and its risk factors, 1990–2019: A systematic analysis for the Global Burden of Disease Study 2019. The Lancet Neurology 2021;20(10):795–820. 34487721 10.1016/S1474-4422(21)00252-0 PMC8443449

[27] GBD 2017 Causes of Death Collaborators . Global, regional, and national age‐sex‐specific mortality for 282 causes of death in 195 countries and territories, 1980–2017: A systematic analysis for the Global Burden of Disease Study 2017. Lancet (London, England) 2018;392(10159):1736–1788. 30496103 10.1016/S0140-6736(18)32203-7 PMC6227606

[28] Y. Fan , Z. Song , and M. Zhang , “Emerging frontiers of artificial intelligence and machine learning in ischemic stroke: A comprehensive investigation of state‐of‐the‐art methodologies, clinical applications, and unraveling challenges,” The EPMA Journal 14, no. 4 (2023): 645–661. 38094579 10.1007/s13167-023-00343-3 PMC10713915

[29] G. A. Mensah , G. A. Roth , and V. Fuster , “The Global Burden of Cardiovascular Diseases and Risk Factors: 2020 and Beyond,” Journal of the American College of Cardiology 74, no. 20 (2019): 2529–2532. 31727292 10.1016/j.jacc.2019.10.009

[30] G. A. Roth , G. A. Mensah , C. O. Johnson , et al., “Global Burden of Cardiovascular Diseases and Risk Factors, 1990–2019: Update From the GBD 2019 Study,” Journal of the American College of Cardiology 76, no. 25 (2020): 2982–3021. 33309175 10.1016/j.jacc.2020.11.010 PMC7755038

[31] I. Kazadi Kabanda , C. Kiangebeni Ngonzo , C.‐K. Emeka Bowamou , et al., “Stroke signs knowledge and factors associated With a delayed hospital arrival of patients With acute stroke in Kinshasa,” Heliyon 10, no. 7 (2024): e28311. 38571603 10.1016/j.heliyon.2024.e28311 PMC10988012

[32] R. Hidayat , M. Fisher , S. P. P. Rima , et al., “The Necessity of Using MRI as an Imaging Modality in Acute Code Stroke in Indonesia,” Vascular Health and Risk Management 21 (2025): 207–215. 40207027 10.2147/VHRM.S503362 PMC11980791

[33] M. L. Prust , R. Forman , and B. Ovbiagele , “Addressing disparities in the global epidemiology of stroke,” Nature Reviews Neurology 20, no. 4 (2024): 207–221. 38228908 10.1038/s41582-023-00921-z

[34] V. L. Feigin and M. O. Owolabi , “Pragmatic solutions to reduce the global burden of stroke: A World Stroke Organization‐Lancet Neurology Commission,” The Lancet Neurology 22, no. 12 (2023): 1160–1206. 37827183 10.1016/S1474-4422(23)00277-6 PMC10715732

[35] M. O. Owolabi , A. G. Thrift , S. Martins , et al., “The state of stroke services Across the globe: Report of World Stroke Organization‐World Health Organization surveys,” International Journal of Stroke: Official Journal of the International Stroke Society 16, no. 8 (2021): 889–901. 33988062 10.1177/17474930211019568 PMC8800855

[36] A. C. de Souza , I. A. Sebastian , W. A. W. Zaidi , et al., “Regional and national differences in stroke thrombolysis use and disparities in pricing, treatment availability, and coverage,” International Journal of Stroke: Official Journal of the International Stroke Society 17, no. 9 (2022): 990–996. 35137645 10.1177/17474930221082446

[37] A. M. Al Hashmi , Y. Imam , O. Y. Mansour , and A. Shuaib , “Organization OtboM‐S. Accreditation of stroke programs at the MENA + region; Between aspiration and reality,” Journal of Stroke and Cerebrovascular Diseases: The Official Journal of National Stroke Association 33, no. 6 (2024): 107639. 38369165 10.1016/j.jstrokecerebrovasdis.2024.107639

[38] R. O. Akinyemi , B. Ovbiagele , O. A. Adeniji , et al., “Stroke in Africa: Profile, progress, prospects and priorities,” Nature Reviews Neurology 17, no. 10 (2021): 634–656. 34526674 10.1038/s41582-021-00542-4 PMC8441961

[39] S. Al‐Rukn , M. Mazya , N. Akhtar , et al., “Stroke in the Middle‐East and North Africa: A 2‐year prospective observational study of intravenous thrombolysis treatment in the region. Results From the SITS‐MENA Registry,” International Journal of Stroke: Official Journal of the International Stroke Society 15, no. 9 (2020): 980–987. 31594533 10.1177/1747493019874729

[40] D. Chang , R. Ibrahim , H. N. Pham , et al., “Rural‐urban stroke mortality gaps in the United States,” Journal of Stroke and Cerebrovascular Diseases: the Official Journal of National Stroke Association 33, no. 8 (2024): 107762. 38723924 10.1016/j.jstrokecerebrovasdis.2024.107762

[41] G. Howard , “Rural‐urban differences in stroke risk,” Preventive Medicine 152, no. Pt 2 (2021): 106661. 34087323 10.1016/j.ypmed.2021.106661 PMC8545748

[42] W.‐J. Tu , Z. Zhao , P. Yin , et al., “Estimated Burden of Stroke in China in 2020,” JAMA Network Open 6, no. 3 (2023): e231455. 36862407 10.1001/jamanetworkopen.2023.1455 PMC9982699

[43] J. Zhu , L. Lin , L. Si , H. Zhao , H. Song , and X. Xu , “Urban and rural disparities in stroke prediction using machine learning Among Chinese older adults,” Scientific Reports 15, no. 1 (2025): 6779. 40000818 10.1038/s41598-025-91157-y PMC11861258

[44] X. Ru , W. Wang , and H. Sun , “GeographicalDifference, Rural‐urban Transition and Trend in Stroke Prevalence in China: Findings From a National Epidemiological Survey of Stroke in China,” Scientific Reports 9, no. 1 (2019): 17330. 31758035 10.1038/s41598-019-53848-1 PMC6874659

[45] S. Khurana , M. Gourie‐Devi , S. Sharma , and S. Kushwaha , “Burden of Stroke in India During 1960 to 2018: A Systematic Review and Meta‐Analysis of Community Based Surveys,” Neurology India 69, no. 3 (2021): 547–559. 34169841 10.4103/0028-3886.317240

[46] V. N. Hedau and T. Patil , “Mounting Stroke Crisis in India: A Systematic Review,” Cureus 16, no. 3 (2024): e57058. 38681344 10.7759/cureus.57058 PMC11052531

[47] E.‐J. Jung , D. Y. Kim , H.‐J. Bae , and K.‐P. Ko , “Assessing regional disparities and vulnerability in stroke care Across Gyeonggi Province: A focus on hospital service areas,” Journal of Stroke and Cerebrovascular Diseases: the Official Journal of National Stroke Association 33, no. 9 (2024): 107817. 38880365 10.1016/j.jstrokecerebrovasdis.2024.107817

[48] Y. Li , M. Zhang , W. Ke , and W. Li , “Impact of Telemedicine Adoption on Hemiplegia in Patients With Stroke in Florida: Longitudinal Observational Study,” Journal of Medical Internet Research [Electronic Resource] 27 (2025): e72315. 40409749 10.2196/72315 PMC12144472

[49] M. Z. Khan , S. Zahid , A. Kichloo , et al., “Gender, Racial, Ethnic, and Socioeconomic Disparities in Palliative Care Encounters in Ischemic Stroke Admissions,” Cardiovascular Revascularization Medicine 35 (2022): 147–154. 33863656 10.1016/j.carrev.2021.04.004

[50] E. K. M. Edzie , P. N. Gorleku , K. Dzefi‐Tettey , et al., “Incidence rate and age of onset of first stroke From CT scan examinations in Cape Coast metropolis,” Heliyon 7, no. 2 (2021): e06214. 33659742 10.1016/j.heliyon.2021.e06214 PMC7892921

[51] L. Liberale , F. Carbone , F. Montecucco , et al., “Ischemic stroke Across sexes: What is the status quo?,” Frontiers in Neuroendocrinology 50 (2018): 3–17. 29753797 10.1016/j.yfrne.2018.05.001

[52] M. El‐Hajj , P. Salameh , S. Rachidi , and H. Hosseini , “The epidemiology of stroke in the Middle East,” European Stroke Journal 1, no. 3 (2016): 180–198. 31008279 10.1177/2396987316654338 PMC6453228

[53] M. S. Ekker , E. M. Boot , A. B. Singhal , et al., “Epidemiology, aetiology, and management of ischaemic stroke in young adults,” The Lancet Neurology 17, no. 9 (2018): 790–801. 30129475 10.1016/S1474-4422(18)30233-3

[54] Y. Béjot , H. Bailly , J. Durier , and M. Giroud , “Epidemiology of stroke in Europe and trends for the 21st century,” La Presse Medicale 45, no. 12 Pt 2 (2016): e391–e398. 27816343 10.1016/j.lpm.2016.10.003

[55] H. Tejada Meza , J. Artal Roy , C. Pérez Lázaro , et al., “Epidemiology and characteristics of ischaemic stroke in young adults in Aragon,” Neurologia 37, no. 6 (2022): 434–440. 10.1016/j.nrleng.2019.05.009 34092536

[56] E. Gurková , L. Štureková , P. Mandysová , and D. Šaňák , “Factors affecting the quality of life After ischemic stroke in young adults: A scoping review,” Health and Quality of Life Outcomes [Electronic Resource] 21, no. 1 (2023): 4. 36653785 10.1186/s12955-023-02090-5 PMC9850784

[57] P. Namaganda , J. Nakibuuka , M. Kaddumukasa , and E. Katabira , “Stroke in young adults, stroke types and risk factors: A case control study,” BMC Neurology 22, no. 1 (2022): 335. 36068544 10.1186/s12883-022-02853-5 PMC9446773

[58] M. H. Leppert , P. M. Ho , J. Burke , et al., “Young Women Had More Strokes Than Young Men in a Large, United States Claims Sample,” Stroke; A Journal of Cerebral Circulation 51, no. 11 (2020): 3352–3355. 10.1161/STROKEAHA.120.030803 PMC7606353 32942966

[59] M. H. Leppert , J. F. Burke , L. D. Lisabeth , et al., “Systematic Review of Sex Differences in Ischemic Strokes Among Young Adults: Are Young Women Disproportionately at Risk?,” Stroke; A Journal of Cerebral Circulation 53, no. 2 (2022): 319–327. 10.1161/STROKEAHA.121.037117 PMC8852306 35073188

[60] S. Koton , Y. Sang , A. L. C. Schneider , W. D. Rosamond , R. F. Gottesman , and J. Coresh , “Trends in Stroke Incidence Rates in Older US Adults: An Update From the Atherosclerosis Risk in Communities (ARIC) Cohort Study,” JAMA Neurology 77, no. 1 (2020): 109–113. 31566685 10.1001/jamaneurol.2019.3258 PMC6777233

[61] J. O. Cerasuolo , L. E. Cipriano , L. A. Sposato , et al., “Population‐based stroke and dementia incidence trends: Age and sex variations,” Alzheimers Dement 13, no. 10 (2017): 1081–1088. 28363085 10.1016/j.jalz.2017.02.010

[62] J. Li , H. Imano , A. Kitamura , et al., “Trends in the incidence of stroke and its subtypes From 1963 to 2018 in Japanese urban and rural communities: The Circulatory Risk in Communities Study (CIRCS),” International Journal of Stroke: Official Journal of the International Stroke Society 18, no. 6 (2023): 657–662. 36268812 10.1177/17474930221135531

[63] L. Li , C. A. Scott , and P. M. Rothwell , “Trends in Stroke Incidence in High‐Income Countries in the 21st Century: Population‐Based Study and Systematic Review,” Stroke; A Journal of Cerebral Circulation 51, no. 5 (2020): 1372–1380. 10.1161/STROKEAHA.119.028484 PMC7185053 32208842

[64] N. Skajaa , K. Adelborg , E. Horváth‐Puhó , et al., “Nationwide Trends in Incidence and Mortality of Stroke Among Younger and Older Adults in Denmark,” Neurology 96, no. 13 (2021): e1711–e1723. 33568547 10.1212/WNL.0000000000011636

[65] T. R. Schneider , T. D. Dittrich , T. Kahles , et al., “First ischemic stroke in young adults: Sex and age‐related differences in stroke rates, risk factors, and etiologies,” European Stroke Journal (2025): 23969873251317347. 10.1177/23969873251317347 PMC11803591 39916317

[66] T. B. H. Potter , J. Tannous , and F. S. Vahidy , “A Contemporary Review of Epidemiology, Risk Factors, Etiology, and Outcomes of Premature Stroke,” Current Atherosclerosis Reports 24, no. 12 (2022): 939–948. 36374365 10.1007/s11883-022-01067-x PMC9660017

[67] T. Akhvlediani , N. Gelenidze , T. Janelidze , et al., “Prevalence of stroke, associated risk factors and stroke related physical, mental, and economic burden in the Republic of Georgia,” European Stroke Journal 7, no. 3 (2022): 305–313. 36082258 10.1177/23969873221101987 PMC9446320

[68] M. A. Danesi , N. U. Okubadejo , F. I. Ojini , and O. O. Ojo , “Incidence and 30‐day case fatality rate of first‐ever stroke in urban Nigeria: The prospective community based Epidemiology of Stroke in Lagos (EPISIL) phase II results,” Journal of the Neurological Sciences 331, no. 1‐2 (2013): 43–47. 23726277 10.1016/j.jns.2013.04.026

[69] D. Adeloye , “An estimate of the incidence and prevalence of stroke in Africa: A systematic review and meta‐analysis,” PLoS ONE 9, no. 6 (2014): e100724. 24967899 10.1371/journal.pone.0100724 PMC4072632

[70] J. K. Russell , C. K. Jones , and P. A. Newhouse , “The Role of Estrogen in Brain and Cognitive Aging,” Neurotherapeutics: the Journal of the American Society for Experimental NeuroTherapeutics 16, no. 3 (2019): 649–665. 31364065 10.1007/s13311-019-00766-9 PMC6694379

[71] C. E. Stewart and F. Sohrabji , “Gonadal hormones and stroke risk: PCOS as a case study,” Frontiers in Neuroendocrinology 58 (2020): 100853. 32640267 10.1016/j.yfrne.2020.100853

[72] M. Giroud , B. Delpont , B. Daubail , et al., “Temporal Trends in Sex Differences With Regard to Stroke Incidence: The Dijon Stroke Registry (1987‐2012),” Stroke; A Journal of Cerebral Circulation 48, no. 4 (2017): 846–849. 10.1161/STROKEAHA.116.015913 28275198

[73] A. Chauhan , H. Moser , and L. D. McCullough , “Sex differences in ischaemic stroke: Potential cellular mechanisms,” Clinical Science (London, England: 1979) 131, no. 7 (2017): 533–552. 28302915 10.1042/CS20160841

[74] T. E. Madsen , J. C. Khoury , M. Leppert , et al., “Temporal Trends in Stroke Incidence Over Time by Sex and Age in the GCNKSS,” Stroke; A Journal of Cerebral Circulation 51, no. 4 (2020): 1070–1076. 10.1161/STROKEAHA.120.028910 PMC7286565 32078459

[75] V. L. Feigin , B. Norrving , and G. A. Mensah , “Global Burden of Stroke,” Circulation Research 120, no. 3 (2017): 439–448. 28154096 10.1161/CIRCRESAHA.116.308413

[76] V. J. Howard , D. O. Kleindorfer , S. E. Judd , et al., “Disparities in stroke incidence contributing to disparities in stroke mortality,” Annals of Neurology 69, no. 4 (2011): 619–627. 21416498 10.1002/ana.22385 PMC3595534

[77] D. O. Kleindorfer , J. Khoury , C. J. Moomaw , et al., “Stroke incidence is decreasing in whites but not in blacks: A population‐based estimate of temporal trends in stroke incidence From the Greater Cincinnati/Northern Kentucky Stroke Study,” Stroke; A Journal of Cerebral Circulation 41, no. 7 (2010): 1326–1331. 10.1161/STROKEAHA.109.575043 PMC2904073 20489177

[78] C. W. Tsao , A. W. Aday , Z. I. Almarzooq , et al., “Heart Disease and Stroke Statistics‐2023 Update: A Report From the American Heart Association,” Circulation 147, no. 8 (2023): e93–e621. 36695182 10.1161/CIR.0000000000001123 PMC12135016

[79] A. N. Wallace , D. P. Gibson , K. S. Asif , et al., “Racial Disparity in Mechanical Thrombectomy Utilization: Multicenter Registry Results From 2016 to 2020,” Journal of the American Heart Association 11, no. 4 (2022): e021865. 35156390 10.1161/JAHA.121.021865 PMC9245822

[80] M. C. Jiménez , J. E. Manson , N. R. Cook , et al., “Racial Variation in Stroke Risk Among Women by Stroke Risk Factors,” Stroke; A Journal of Cerebral Circulation 50, no. 4 (2019): 797–804. 10.1161/STROKEAHA.117.017759 PMC6433502 30869565

[81] J. P. Broderick , T. Brott , T. Tomsick , G. Huster , and R. Miller , “The risk of subarachnoid and intracerebral hemorrhages in blacks as compared With whites,” New England Journal of Medicine 326, no. 11 (1992): 733–736. 1738378 10.1056/NEJM199203123261103

[82] A. I. Qureshi , W. H. Giles , and J. B. Croft , “Racial differences in the incidence of intracerebral hemorrhage: Effects of blood pressure and education,” Neurology 52, no. 8 (1999): 1617–1621. 10331687 10.1212/wnl.52.8.1617

[83] S. Song , L. Liang , G. C. Fonarow , et al., “Comparison of Clinical Care and In‐Hospital Outcomes of Asian American and White Patients With Acute Ischemic Stroke,” JAMA Neurology 76, no. 4 (2019): 430–439. 30667466 10.1001/jamaneurol.2018.4410 PMC6459126

[84] S. J. Mendelson , S. Zhang , R. Matsouaka , et al., “Race‐Ethnic Disparities in Rates of Declination of Thrombolysis for Stroke,” Neurology 98, no. 16 (2022): e1596–e1604. 35228335 10.1212/WNL.0000000000200138 PMC9052571

[85] G. W. Albers , L. R. Caplan , J. D. Easton , et al., “Transient ischemic attack–proposal for a new definition,” New England Journal of Medicine 347, no. 21 (2002): 1713–1716. 12444191 10.1056/NEJMsb020987

[86] N. Najib , P. Magin , D. Lasserson , et al., “Contemporary prognosis of transient ischemic attack patients: A systematic review and meta‐analysis,” International Journal of Stroke 14, no. 5 (2019): 460–467. 30632953 10.1177/1747493018823568

[87] V. A. Lioutas , C. S. Ivan , J. J. Himali , et al., “Incidence of Transient Ischemic Attack and Association With Long‐term Risk of Stroke,” Jama 325, no. 4 (2021): 373–381. 33496774 10.1001/jama.2020.25071 PMC7838926

[88] G. J. Hankey and C. P. Warlow , “Treatment and secondary prevention of stroke: Evidence, costs, and effects on individuals and populations,” Lancet 354, no. 9188 (1999): 1457–1463. 10543686 10.1016/S0140-6736(99)04407-4

[89] S. Khare , “Risk factors of transient ischemic attack: An overview,” Journal of mid‐life Health 7, no. 1 (2016): 2–7. 27134474 10.4103/0976-7800.179166 PMC4832890

[90] S. C. Johnston , P. B. Fayad , P. B. Gorelick , et al., “Prevalence and knowledge of transient ischemic attack Among US adults,” Neurology 60, no. 9 (2003): 1429–1434. 12743226 10.1212/01.wnl.0000063309.41867.0f

[91] T. Komulainen , A. Koivisto , and P. Jäkälä , “Incidence of first‐ever transient ischemic attack in Eastern Finland,” Acta Neurologica Scandinavica 146, no. 5 (2022): 615–622. 36029100 10.1111/ane.13689 PMC9805147

[92] D. J. Robinson , R. Stanton , H. Sucharew , et al., “Racial Disparities in Stroke Recurrence: A Population‐Based Study,” Neurology 99, no. 22 (2022): e2464–e2473. 36041865 10.1212/WNL.0000000000201225 PMC9728039

[93] W. Wang , P. Sun , F. Han , and C. Qu , “Sex Differences in Risk Factors for Transient Ischemic Attack in a Chinese Population,” Frontiers in Neurology 12 (2021): 615399. 34025549 10.3389/fneur.2021.615399 PMC8134545

[94] D. Kleindorfer , P. Panagos , A. Pancioli , et al., “Incidence and short‐term prognosis of transient ischemic attack in a population‐based study,” Stroke; A Journal of Cerebral Circulation 36, no. 4 (2005): 720–723. 10.1161/01.STR.0000158917.59233.b7 15731465

[95] V. L. Feigin , G. A. Roth , M. Naghavi , et al., “Global burden of stroke and risk factors in 188 countries, During 1990–2013: A systematic analysis for the Global Burden of Disease Study 2013,” The Lancet Neurology 15, no. 9 (2016): 913–924. 27291521 10.1016/S1474-4422(16)30073-4

[96] A. Guzik and C. Bushnell , “Stroke Epidemiology and Risk Factor Management,” Continuum (Minneapolis, Minn) 23, no. 1, Cerebrovascular Disease (2017): 15–39. 28157742 10.1212/CON.0000000000000416

[97] B. B. Johansson , “Hypertension mechanisms causing stroke,” Clinical and Experimental Pharmacology & Physiology 26, no. 7 (1999): 563–565. 10405790 10.1046/j.1440-1681.1999.03081.x

[98] F. Hazama , A. Ooshima , T. Tanaka , K. Tomimoto , and K. Okamoto , “Vascular lesions in the various substrains of spontaneously hypertensive rats and the effects of chronic salt ingestion,” Japanese Circulation Journal 39, no. 1 (1975): 7–22. 1127829 10.1253/jcj.39.7

[99] Y. Yamori , R. Horie , M. Sato , S. Sasagawa , and K. Okamoto , “Experimental studies on the pathogenesis and prophylaxis of stroke in stroke‐prone spontaneously hypertensive rats (SHR).(1) Quantitative estimation of cerebrovascular permeability,” Japanese Circulation Journal 39, no. 5 (1975): 611–615. 1152193 10.1253/jcj.39.611

[100] K. Fredriksson , C. Nordborg , H. Kalimo , Y. Olsson , and B. B. Johansson , “Cerebral microangiopathy in stroke‐prone spontaneously hypertensive rats. An immunohistochemical and ultrastructural study,” Acta Neuropathologica 75, no. 3 (1988): 241–252. 3348082 10.1007/BF00690532

[101] B. A. Gross , B. T. Jankowitz , and R. M. Friedlander , “Cerebral Intraparenchymal Hemorrhage: A Review,” Jama 321, no. 13 (2019): 1295–1303. 30938800 10.1001/jama.2019.2413

[102] A. E. Doyle , “Hypertension and vascular disease,” American Journal of Hypertension 4, no. 2 Pt 2 (1991): 103S–106S. 2021454 10.1093/ajh/4.2.103s

[103] R. Clarke , N. Wright , R. Walters , et al., “Genetically Predicted Differences in Systolic Blood Pressure and Risk of Cardiovascular and Noncardiovascular Diseases: A Mendelian Randomization Study in Chinese Adults,” Hypertension 80, no. 3 (2023): 566–576. 36601918 10.1161/HYPERTENSIONAHA.122.20120 PMC7614188

[104] M. O. Owolabi , F. Sarfo , R. Akinyemi , et al., “Dominant modifiable risk factors for stroke in Ghana and Nigeria (SIREN): A case‐control study,” The Lancet Global Health 6, no. 4 (2018): e436–e446. 29496511 10.1016/S2214-109X(18)30002-0 PMC5906101

[105] N. K. Itoga , D. S. Tawfik , M. E. Montez‐Rath , and T. I. Chang , “Contributions of Systolic and Diastolic Blood Pressures to Cardiovascular Outcomes in the ALLHAT Study,” Journal of the American College of Cardiology 78, no. 17 (2021): 1671–1678. 34674811 10.1016/j.jacc.2021.08.035

[106] P. Banyas and A. Jadhav , “Stroke and Transient Ischemic Attack,” Primary Care 51, no. 2 (2024): 283–297. 38692775 10.1016/j.pop.2024.02.004

[107] J. Guo , J. Lv , Y. Guo , et al., “Association Between blood pressure categories and cardiovascular disease mortality in China,” PLoS ONE 16, no. 7 (2021): e0255373. 34329344 10.1371/journal.pone.0255373 PMC8323908

[108] American Diabetes Association . 2. Classification and Diagnosis of Diabetes: Standards of Medical Care in Diabetes‐2020. Diabetes Care 2020;43(Suppl 1):S14–S31. 31862745 10.2337/dc20-S002

[109] B. Klijs , M. Mitratza , P. P. Harteloh , et al., “Estimating the lifetime risk of dementia using nationwide individually linked cause‐of‐death and health register data,” International Journal of Epidemiology 50, no. 3 (2021): 809–816. 33354723 10.1093/ije/dyaa219

[110] F. S. Sarfo , B. Ovbiagele , M. Gebregziabher , et al., “Stroke Among Young West Africans: Evidence From the SIREN (Stroke Investigative Research and Educational Network) Large Multisite Case‐Control Study,” Stroke; A Journal of Cerebral Circulation 49, no. 5 (2018): 1116–1122. 10.1161/STROKEAHA.118.020783 PMC5916042 29618553

[111] A. Mavridis , A. Viktorisson , B. Eliasson , M. von Euler , and K. S. Sunnerhagen , “Risk of Ischemic and Hemorrhagic Stroke in Individuals With Type 1 and Type 2 Diabetes: A Nationwide Cohort Study in Sweden,” Neurology 104, no. 7 (2025): e213480. 40080734 10.1212/WNL.0000000000213480 PMC11907640

[112] C. Banerjee , Y. P. Moon , M. C. Paik , et al., “Duration of diabetes and risk of ischemic stroke: The Northern Manhattan Study,” Stroke; A Journal of Cerebral Circulation 43, no. 5 (2012): 1212–1217. 10.1161/STROKEAHA.111.641381 PMC3336044 22382158

[113] J. I. Barzilay , Y. M. K. Farag , and J. Durthaler , “Albuminuria: An Underappreciated Risk Factor for Cardiovascular Disease,” Journal of the American Heart Association 13, no. 2 (2024): e030131. 38214258 10.1161/JAHA.123.030131 PMC10926810

[114] O. I. Brown , M. Drozd , H. McGowan , et al., “Relationship Among Diabetes, Obesity, and Cardiovascular Disease Phenotypes: A UK Biobank Cohort Study,” Diabetes Care 46, no. 8 (2023): 1531–1540. 37368983 10.2337/dc23-0294 PMC10369123

[115] C. D. Maida , M. Daidone , G. Pacinella , R. L. Norrito , A. Pinto , and A. Tuttolomondo , “Diabetes and Ischemic Stroke: An Old and New Relationship an Overview of the Close Interaction Between These Diseases,” International Journal of Molecular Sciences 23, no. 4 (2022): 2397. 35216512 10.3390/ijms23042397 PMC8877605

[116] F. Cacciapuoti , “Some considerations About the hypercoagulable states and their treatments,” Blood Coagulation & Fibrinolysis 22, no. 3 (2011): 155–159. 21346557 10.1097/MBC.0b013e3283436401

[117] D. Sagris , G. Ntaios , and H. Milionis , “Beyond antithrombotics: Recent advances in pharmacological risk factor management for secondary stroke prevention,” Journal of Neurology, Neurosurgery, and Psychiatry 95, no. 3 (2024): 264–272. 37775267 10.1136/jnnp-2022-329149

[118] J. Li , C. Ji , W. Zhang , L. Lan , and W. Ge , “Effect of new glucose‐lowering drugs on stroke in patients With type 2 diabetes: A systematic review and Meta‐analysis,” Journal of Diabetes and Its Complications 37, no. 1 (2023): 108362. 36462459 10.1016/j.jdiacomp.2022.108362

[119] I. Escudero‐Martínez , L. Morales‐Caba , and T. Segura , “Atrial fibrillation and stroke: A review and new insights,” Trends in Cardiovascular Medicine 33, no. 1 (2023): 23–29. 34890796 10.1016/j.tcm.2021.12.001

[120] F. Rahman , G. F. Kwan , and E. J. Benjamin , “Global epidemiology of atrial fibrillation,” Nature Reviews Cardiology 11, no. 11 (2014): 639–654. 25113750 10.1038/nrcardio.2014.118

[121] L. A. Sposato , L. E. Cipriano , G. Saposnik , E. Ruíz Vargas , P. M. Riccio , and V. Hachinski , “Diagnosis of atrial fibrillation After stroke and transient ischaemic attack: A systematic review and meta‐analysis,” The Lancet Neurology 14, no. 4 (2015): 377–387. 25748102 10.1016/S1474-4422(15)70027-X

[122] B. W. Calenda , V. Fuster , J. L. Halperin , and C. B. Granger , “Stroke risk assessment in atrial fibrillation: Risk factors and markers of atrial myopathy,” Nature Reviews Cardiology 13, no. 9 (2016): 549–559. 27383079 10.1038/nrcardio.2016.106

[123] L. Staerk , J. A. Sherer , D. Ko , E. J. Benjamin , and R. H. Helm , “Atrial Fibrillation: Epidemiology, Pathophysiology, and Clinical Outcomes,” Circulation Research 120, no. 9 (2017): 1501–1517. 28450367 10.1161/CIRCRESAHA.117.309732 PMC5500874

[124] J. Dietzel , K. G. Haeusler , and M. Endres , “Does atrial fibrillation cause cognitive decline and dementia?,” Europace 20, no. 3 (2018): 408–419. 28387847 10.1093/europace/eux031

[125] A. J. Kerr , M. B. Simmonds , and R. A. Stewart , “Influence of heart rate on stroke volume variability in atrial fibrillation in patients With normal and impaired left ventricular function,” American Journal of Cardiology 82, no. 12 (1998): 1496–1500. 9874054 10.1016/s0002-9149(98)00693-6

[126] M. Gardarsdottir , S. Sigurdsson , T. Aspelund , et al., “Atrial fibrillation is associated With decreased total cerebral blood flow and brain perfusion,” Europace 20, no. 8 (2018): 1252–1258. 29016776 10.1093/europace/eux220 PMC6075509

[127] D. S. G. Conway and G. Y. H. Lip , “Inflammation, arrhythmia burden and the thrombotic consequences of atrial fibrillation,” European Heart Journal 25, no. 19 (2004): 1761. 10.1016/j.ehj.2004.08.001 15451156

[128] A. A. Khan and G. Y. H. Lip , “The prothrombotic state in atrial fibrillation: Pathophysiological and management implications,” Cardiovascular Research 115, no. 1 (2019): 31–45. 30388199 10.1093/cvr/cvy272

[129] H. Wersching , T. Duning , H. Lohmann , et al., “Serum C‐reactive protein is linked to cerebral microstructural integrity and cognitive function,” Neurology 74, no. 13 (2010): 1022–1029. 20350977 10.1212/WNL.0b013e3181d7b45b

[130] C. R. Pennington , “Right atrial thrombus: A complication of total parenteral nutrition,” British Medical Journal 295, no. 6595 (1987): 446–447. 10.1136/bmj.295.6595.446-b PMC1247309 3115494

[131] Y. Z. Imam , S. Kamran , N. Akhtar , et al., “Incidence, clinical features and outcomes of atrial fibrillation and stroke in Qatar,” International Journal of Stroke: Official Journal of the International Stroke Society 15, no. 1 (2020): 85–89. 30789323 10.1177/1747493019830577

[132] A. C. Goulart , R. D. Olmos , I. S. Santos , et al., “The impact of atrial fibrillation and long‐term oral anticoagulant use on all‐cause and cardiovascular mortality: A 12‐year evaluation of the prospective Brazilian Study of Stroke Mortality and Morbidity,” International Journal of Stroke 17, no. 1 (2022): 48–58. 33527882 10.1177/1747493021995592

[133] P. Kirchhof , G. Radaideh , Y.‐H. Kim , et al., “Global Prospective Safety Analysis of Rivaroxaban,” Journal of the American College of Cardiology 72, no. 2 (2018): 141–153. 29976287 10.1016/j.jacc.2018.04.058

[134] A. Massaro , R. P. Giugliano , B. Norrving , A. Oto , and R. Veltkamp , “Overcoming global challenges in stroke prophylaxis in atrial fibrillation: The role of non‐vitamin K antagonist oral anticoagulants,” International Journal of Stroke: Official Journal of the International Stroke Society 11, no. 9 (2016): 950–967. 27703066 10.1177/1747493016660106

[135] B. T. King , P. D. Lawrence , T. J. Milling , and S. J. Warach , “Optimal delay time to initiate anticoagulation After ischemic stroke in atrial fibrillation (START): Methodology of a pragmatic, response‐adaptive, prospective randomized clinical trial,” International Journal of Stroke: Official Journal of the International Stroke Society 14, no. 9 (2019): 977–982. 31423922 10.1177/1747493019870651 PMC7401695

[136] D. M. Lloyd‐Jones , N. B. Allen , and C. A. M. Anderson , “Life's Essential 8: Updating and Enhancing the American Heart Association's Construct of Cardiovascular Health: A Presidential Advisory From the American Heart Association,” Circulation 146, no. 5 (2022): e18–e43. 35766027 10.1161/CIR.0000000000001078 PMC10503546

[137] X. Wang , Y. Dong , X. Qi , C. Huang , and L. Hou , “Cholesterol levels and risk of hemorrhagic stroke: A systematic review and meta‐analysis,” Stroke; A Journal of Cerebral Circulation 44, no. 7 (2013): 1833–1839. 10.1161/STROKEAHA.113.001326 23704101

[138] L. Sun , R. Clarke , D. Bennett , et al., “Causal associations of blood lipids With risk of ischemic stroke and intracerebral hemorrhage in Chinese adults,” Nature Medicine 25, no. 4 (2019): 569–574. 10.1038/s41591-019-0366-x PMC6795549 30858617

[139] B. A. Ference , H. N. Ginsberg , I. Graham , et al., “Low‐density lipoproteins cause atherosclerotic cardiovascular disease. 1. Evidence From genetic, epidemiologic, and clinical studies. A consensus statement From the European Atherosclerosis Society Consensus Panel,” European Heart Journal 38, no. 32 (2017): 2459–2472. 28444290 10.1093/eurheartj/ehx144 PMC5837225

[140] K. Tziomalos , V. G. Athyros , A. Karagiannis , and D. P. Mikhailidis , “Dyslipidemia as a risk factor for ischemic stroke,” Current Topics in Medicinal Chemistry 9, no. 14 (2009): 1291–1297. 19849661 10.2174/156802609789869628

[141] M. R. Law , N. J. Wald , and A. R. Rudnicka , “Quantifying effect of statins on low density lipoprotein cholesterol, ischaemic heart disease, and stroke: Systematic review and meta‐analysis,” BMJ (Clinical Research Ed) 326, no. 7404 (2003): 1423. 10.1136/bmj.326.7404.1423 PMC162260 12829554

[142] P. Amarenco , J. S. Kim , J. Labreuche , et al., “A Comparison of Two LDL Cholesterol Targets After Ischemic Stroke,” The New England Journal of Medicine 382, no. 1 (2020): 9. 31738483 10.1056/NEJMoa1910355

[143] E. Lindenstrøm , G. Boysen , and J. Nyboe , “Influence of total cholesterol, high density lipoprotein cholesterol, and triglycerides on risk of cerebrovascular disease: The Copenhagen City Heart Study,” BMJ (Clinical Research Ed) 309, no. 6946 (1994): 11–15. 10.1136/bmj.309.6946.11 PMC2542648 8044059

[144] H. Lee , J.‐B. Park , I.‐C. Hwang , et al., “Association of four lipid components With mortality, myocardial infarction, and stroke in statin‐naïve young adults: A nationwide cohort study,” European Journal of Preventive Cardiology 27, no. 8 (2020): 870–881. 32013600 10.1177/2047487319898571

[145] P. M. Rist , J. E. Buring , P. M. Ridker , C. S. Kase , T. Kurth , and K. M. Rexrode , “Lipid levels and the risk of hemorrhagic stroke Among women,” Neurology 92, no. 19 (2019): e2286–e2294. 30971484 10.1212/WNL.0000000000007454 PMC6537127

[146] A. K. Boehme , C. Esenwa , and M. S. V. Elkind , “Stroke Risk Factors, Genetics, and Prevention,” Circulation Research 120, no. 3 (2017): 472–495. 28154098 10.1161/CIRCRESAHA.116.308398 PMC5321635

[147] Y. Pan , R. Wangqin , H. Li , et al., “LDL‐C levels, lipid‐lowering treatment and recurrent stroke in minor ischaemic stroke or TIA,” Stroke and Vascular Neurology 7, no. 4 (2022): 276–284. 35256525 10.1136/svn-2021-001317 PMC9453834

[148] D. G. Hackam , P. C. Austin , A. Huang , et al., “Statins and intracerebral hemorrhage: A retrospective cohort study,” Archives of Neurology 69, no. 1 (2012): 39–45. 21911657 10.1001/archneurol.2011.228

[149] A. Lauer , S. M. Greenberg , and M. E. Gurol , “Statins in Intracerebral Hemorrhage,” Current Atherosclerosis Reports 17, no. 8 (2015): 46. 26092038 10.1007/s11883-015-0526-5

[150] E. L. Harshfield , M. K. Georgakis , R. Malik , M. Dichgans , and H. S. Markus , “Modifiable Lifestyle Factors and Risk of Stroke: A Mendelian Randomization Analysis,” Stroke; A Journal of Cerebral Circulation 52, no. 3 (2021): 931–936. 10.1161/STROKEAHA.120.031710 PMC7903981 33535786

[151] M. J. Thun , L. F. Apicella , and S. J. Henley , “Smoking vs other risk factors as the cause of smoking‐attributable deaths: Confounding in the courtroom,” Jama 284, no. 6 (2000): 706–712. 10927778 10.1001/jama.284.6.706

[152] D. Hammond , “Smoking behaviour Among young adults: Beyond youth prevention,” Tobacco Control 14, no. 3 (2005): 181–185. 15923468 10.1136/tc.2004.009621 PMC1748046

[153] X. Wang , X. Liu , M. J. O'Donnell , et al., “Tobacco use and risk of acute stroke in 32 countries in the INTERSTROKE study: A case‐control study,” EClinicalMedicine 70 (2024): 102515. 38516107 10.1016/j.eclinm.2024.102515 PMC10955659

[154] A. Hackshaw , J. K. Morris , S. Boniface , J.‐L. Tang , and D. Milenković , “Low cigarette consumption and risk of coronary heart disease and stroke: Meta‐analysis of 141 cohort studies in 55 study reports,” BMJ (Clinical Research Ed) 360 (2018): j5855. 10.1136/bmj.j5855 PMC5781309 29367388

[155] J. V. Lindbohm , J. Kaprio , P. Jousilahti , V. Salomaa , and M. Korja , “Sex, Smoking, and Risk for Subarachnoid Hemorrhage,” Stroke; A Journal of Cerebral Circulation 47, no. 8 (2016): 1975–1981. 10.1161/STROKEAHA.116.012957 27444257

[156] G. Siasos , V. Tsigkou , E. Kokkou , et al., “Smoking and atherosclerosis: Mechanisms of disease and new therapeutic approaches,” Current Medicinal Chemistry 21, no. 34 (2014): 3936–3948. 25174928 10.2174/092986732134141015161539

[157] U. Förstermann and T. Münzel , “Endothelial nitric oxide synthase in vascular disease: From marvel to menace,” Circulation 113, no. 13 (2006): 1708–1714. 16585403 10.1161/CIRCULATIONAHA.105.602532

[158] Z. Gu , V. Fonseca , and C.‐M. Hai , “Nicotinic acetylcholine receptor mediates nicotine‐induced actin cytoskeletal remodeling and extracellular matrix degradation by vascular smooth muscle cells,” Vascular Pharmacology 58, no. 1‐2 (2013): 87–97. 22940282 10.1016/j.vph.2012.08.003 PMC3530635

[159] Q. Zhang , X. Tang , Z.‐F. Zhang , R. Velikina , S. Shi , and A. D. Le , “Nicotine induces hypoxia‐inducible factor‐1alpha expression in human lung cancer cells via nicotinic acetylcholine receptor‐mediated signaling pathways,” Clinical Cancer Research 13, no. 16 (2007): 4686–4694. 17699846 10.1158/1078-0432.CCR-06-2898 PMC4166418

[160] M. K. C. Ng , J. Wu , E. Chang , et al., “A central role for nicotinic cholinergic regulation of growth factor‐induced endothelial cell migration,” Arteriosclerosis, Thrombosis, and Vascular Biology 27, no. 1 (2007): 106–112. 17082486 10.1161/01.ATV.0000251517.98396.4a

[161] A. Tedgui and Z. Mallat , “Anti‐inflammatory mechanisms in the vascular wall,” Circulation Research 88, no. 9 (2001): 877–887. 11348996 10.1161/hh0901.090440

[162] A. Bowie and L. A. O'Neill , “Oxidative stress and nuclear factor‐kappaB activation: A reassessment of the evidence in the light of recent discoveries,” Biochemical Pharmacology 59, no. 1 (2000): 13–23. 10605930 10.1016/s0006-2952(99)00296-8

[163] C. Pelletier , N. Varin‐Blank , J. Rivera , et al., “Fc epsilonRI‐mediated induction of TNF‐alpha gene expression in the RBL‐2H3 mast cell line: Regulation by a novel NF‐kappaB‐Like nuclear binding complex,” Journal of Immunology (Baltimore, Md: 1950) 161, no. 9 (1998): 4768–4776. 9794408

[164] M. A. Collart , P. Baeuerle , and P. Vassalli , “Regulation of tumor necrosis factor alpha transcription in macrophages: Involvement of four kappa B‐Like motifs and of constitutive and inducible forms of NF‐kappa B,” Molecular and Cellular Biology 10, no. 4 (1990): 1498–1506. 2181276 10.1128/mcb.10.4.1498-1506.1990 PMC362253

[165] A. Csiszar , A. Podlutsky , M. S. Wolin , G. Losonczy , P. Pacher , and Z. Ungvari , “Oxidative stress and accelerated vascular aging: Implications for cigarette smoking,” Frontiers in Bioscience‐Landmark Ed 14, no. 8 (2009): 3128–3144. 10.2741/3440 PMC2756477 19273262

[166] E. A. Jaimes , E. G. DeMaster , R.‐X. Tian , and L. Raij , “Stable compounds of cigarette smoke induce endothelial superoxide anion production via NADPH oxidase activation,” Arteriosclerosis, Thrombosis, and Vascular Biology 24, no. 6 (2004): 1031–1036. 15059808 10.1161/01.ATV.0000127083.88549.58

[167] G. Arunachalam , H. Yao , I. K. Sundar , S. Caito , and I. Rahman , “SIRT1 regulates oxidant‐ and cigarette smoke‐induced eNOS acetylation in endothelial cells: Role of resveratrol,” Biochemical and Biophysical Research Communications 393, no. 1 (2010): 66–72. 20102704 10.1016/j.bbrc.2010.01.080 PMC2830376

[168] M. M. Rahman , S. Elmi , T. K. H. Chang , et al., “Increased vascular contractility in isolated vessels From cigarette smoking rats is mediated by basal endothelin release,” Vascular Pharmacology 46, no. 1 (2007): 35–42. 16901763 10.1016/j.vph.2006.06.006

[169] A. D. Flouris , C. I. Vardavas , G. S. Metsios , A. M. Tsatsakis , and Y. Koutedakis , “Biological evidence for the acute health effects of secondhand smoke exposure,” American Journal of Physiology. Lung Cellular and Molecular Physiology 298, no. 1 (2010): L3–L12. 19767410 10.1152/ajplung.00215.2009

[170] C. Armani , L. Landini , and A. Leone , “Molecular and biochemical changes of the cardiovascular system due to smoking exposure,” Current Pharmaceutical Design 15, no. 10 (2009): 1038–1053. 19355946 10.2174/138161209787846973

[171] C. Antoniades , D. Tousoulis , C. Vasiliadou , et al., “Combined effects of smoking and hypercholesterolemia on inflammatory process, thrombosis/fibrinolysis system, and forearm hyperemic response,” American Journal of Cardiology 94, no. 9 (2004): 1181–1184. 15518617 10.1016/j.amjcard.2004.07.090

[172] D. M. Burns , “Epidemiology of smoking‐induced cardiovascular disease,” Progress in Cardiovascular Diseases 46, no. 1 (2003): 11–29. 12920698 10.1016/s0033-0620(03)00079-3

[173] K. Fagerström , “The epidemiology of smoking: Health consequences and benefits of cessation,” Drugs 62, no. Suppl 2 (2002): 1–9. 10.2165/00003495-200262002-00001 12109931

[174] K. A. Epstein , C. M. Viscoli , J. D. Spence , et al., “Smoking cessation and outcome After ischemic stroke or TIA,” Neurology 89, no. 16 (2017): 1723–1729. 28887378 10.1212/WNL.0000000000004524 PMC5644463

[175] A. Aigner , U. Grittner , A. Rolfs , B. Norrving , B. Siegerink , and M. A. Busch , “Contribution of Established Stroke Risk Factors to the Burden of Stroke in Young Adults,” Stroke; A Journal of Cerebral Circulation 48, no. 7 (2017): 1744–1751. 10.1161/STROKEAHA.117.016599 28619986

[176] J. S. Gill , A. V. Zezulka , M. J. Shipley , S. K. Gill , and D. G. Beevers , “Stroke and alcohol consumption,” The New England Journal of Medicine 315, no. 17 (1986): 1041–1046. 2876380 10.1056/NEJM198610233151701

[177] C. Jw , L. Sr , C. Ek , et al., “Cumulative Alcohol Consumption Burden and the Risk of Stroke in Young Adults: A Nationwide Population‐Based Study,” Neurology 100, no. 5 (2023): e505–e515. 36323515 10.1212/WNL.0000000000201473 PMC9931082

[178] W. Qi , J. Ma , T. Guan , et al., “Risk Factors for Incident Stroke and Its Subtypes in China: A Prospective Study,” Journal of the American Heart Association 9, no. 21 (2020): e016352. 33103569 10.1161/JAHA.120.016352 PMC7763402

[179] J. Susts , M. Reinholdsson , K. S. Sunnerhagen , and T. Abzhandadze , “Physical inactivity Before stroke is associated With dependency in basic activities of daily living 3 months After stroke,” Frontiers In Neurology 14 (2023): 1094232. 36824422 10.3389/fneur.2023.1094232 PMC9942155

[180] J. E. Manson , G. A. Colditz , M. J. Stampfer , et al., “A prospective study of maturity‐onset diabetes mellitus and risk of coronary heart disease and stroke in women,” Archives of Internal Medicine 151, no. 6 (1991): 1141–1147. 2043016

[181] J. L. Taylor , “Exercise and the Brain in Cardiovascular Disease: A Narrative Review,” Heart and Mind 7, no. 1 (2023): 5–12.

[182] E. L. O'Keefe , J. H. O'Keefe , and C. J. Lavie , “The Intersection of Exercise, Cognition, and Cardiovascular Disease,” Heart and Mind 7, no. 1 (2023): 3–4.

[183] S. Ghozy , A. H. Zayan , A. E. El‐Qushayri , et al., “Physical activity level and stroke risk in US population: A matched case‐control study of 102,578 individuals,” Annals of Clinical and Translational Neurology 9, no. 3 (2022): 264–275. 35094505 10.1002/acn3.51511 PMC8935290

[184] L. J. Appel , M. W. Brands , S. R. Daniels , N. Karanja , P. J. Elmer , and F. M. Sacks , “Dietary approaches to prevent and treat hypertension: A scientific statement From the American Heart Association,” Hypertension 47, no. 2 (2006): 296–308. 16434724 10.1161/01.HYP.0000202568.01167.B6

[185] O. J. Asowata , I. Bodunde , A. P. Okekunle , et al., “Low vegetable consumption doubles the odds of stroke Among people With hypertension: Findings From the SIREN Study in West Africa,” International Journal of Stroke: Official Journal of the International Stroke Society (2025): 17474930251349474. 10.1177/17474930251349474 40476509

[186] J. Taïlé , M. Bringart , C. Planesse , et al., “Antioxidant Polyphenols of Antirhea borbonica Medicinal Plant and Caffeic Acid Reduce Cerebrovascular, Inflammatory and Metabolic Disorders Aggravated by High‐Fat Diet‐Induced Obesity in a Mouse Model of Stroke,” Antioxidants (Basel, Switzerland) 11, no. 5 (2022): 858. 35624723 10.3390/antiox11050858 PMC9138119

[187] N. Guo , Y. Zhu , D. Tian , et al., “Role of diet in stroke incidence: An umbrella review of meta‐analyses of prospective observational studies,” BMC Medicine [Electronic Resource] 20, no. 1 (2022): 194. 35606791 10.1186/s12916-022-02381-6 PMC9128224

[188] T. Y. N. Tong , P. N. Appleby , T. J. Key , et al., “The associations of major foods and fibre With risks of ischaemic and haemorrhagic stroke: A prospective study of 418 329 participants in the EPIC cohort Across nine European countries,” European Heart Journal 41, no. 28 (2020): 2632–2640. 32090257 10.1093/eurheartj/ehaa007 PMC7377582

[189] A. Ojagbemi , A. P. Okekunle , P. Olowoyo , et al., “Dietary intakes of green leafy vegetables and incidence of cardiovascular diseases,” Cardiovascular Journal of Africa 32, no. 4 (2021): 215–223. 34128951 10.5830/CVJA-2021-017 PMC8756059

[190] J. D. Stanaway , A. Afshin , C. Ashbaugh , et al., “Health effects associated With vegetable consumption: A Burden of Proof study,” Nature Medicine 28, no. 10 (2022): 2066–2074. 10.1038/s41591-022-01970-5 PMC9556321 36216936

[191] A. C. Padin , J. R. Hébert , A. Woody , et al., “A proinflammatory diet is associated With inflammatory gene expression Among healthy, non‐obese adults: Can social ties protect Against the risks?,” Brain, Behavior, and Immunity 82 (2019): 36–44. 31356923 10.1016/j.bbi.2019.07.031 PMC6800628

[192] J.‐M. Schwarz , S. M. Noworolski , A. Erkin‐Cakmak , et al., “Effects of Dietary Fructose Restriction on Liver Fat, De Novo Lipogenesis, and Insulin Kinetics in Children With Obesity,” Gastroenterology 153, no. 3 (2017): 743–752. 28579536 10.1053/j.gastro.2017.05.043 PMC5813289

[193] M. P. Pase , J. J. Himali , A. S. Beiser , et al., “Sugar‐ and Artificially Sweetened Beverages and the Risks of Incident Stroke and Dementia: A Prospective Cohort Study,” Stroke; A Journal of Cerebral Circulation 48, no. 5 (2017): 1139–1146. 10.1161/STROKEAHA.116.016027 PMC5405737 28428346

[194] J. He , L. G. Ogden , S. Vupputuri , L. A. Bazzano , C. Loria , and P. K. Whelton , “Dietary sodium intake and subsequent risk of cardiovascular disease in overweight adults,” Jama 282, no. 21 (1999): 2027–2034. 10591385 10.1001/jama.282.21.2027

[195] S.‐H. Suk , R. L. Sacco , B. Boden‐Albala , et al., “Abdominal obesity and risk of ischemic stroke: The Northern Manhattan Stroke Study,” Stroke; A Journal of Cerebral Circulation 34, no. 7 (2003): 1586–1592. 10.1161/01.STR.0000075294.98582.2F 12775882

[196] S. P. Walker , E. B. Rimm , A. Ascherio , I. Kawachi , M. J. Stampfer , and W. C. Willett , “Body size and fat distribution as predictors of stroke Among US men,” American Journal of Epidemiology 144, no. 12 (1996): 1143–1150. 8956626 10.1093/oxfordjournals.aje.a008892

[197] Y. Lu , K. Hajifathalian , M. Ezzati , M. Woodward , E. B. Rimm , and G. Danaei , “Metabolic mediators of the effects of body‐mass index, overweight, and obesity on coronary heart disease and stroke: A pooled analysis of 97 prospective cohorts With 1·8 million participants,” Lancet (London, England) 383, no. 9921 (2014): 970–983. 24269108 10.1016/S0140-6736(13)61836-X PMC3959199

[198] Y. Lan , J. Shang , Y. Ma , et al., “A new predictor of coronary artery disease in acute ischemic stroke or transient ischemic attack patients: Pericarotid fat density,” European Radiology 34, no. 3 (2024): 1667–1676. 37672057 10.1007/s00330-023-10046-y

[199] M. J. O'Donnell , D. Xavier , L. Liu , et al., “Risk factors for ischaemic and intracerebral haemorrhagic stroke in 22 countries (the INTERSTROKE study): A case‐control study,” Lancet (London, England) 376, no. 9735 (2010): 112–123. 20561675 10.1016/S0140-6736(10)60834-3

[200] J. S. Kim , J. Song , S. Choi , and S. M. Park , “General obesity, abdominal obesity, and the risk of cardiovascular disease including stroke in 5‐year breast cancer survivors,” Breast (Edinburgh, Scotland) 79 (2025): 103857. 39675093 10.1016/j.breast.2024.103857 PMC11699294

[201] S. Kumari , R. Dhapola , P. Sharma , P. Nagar , B. Medhi , and D. HariKrishnaReddy , “The impact of cytokines in neuroinflammation‐mediated stroke,” Cytokine & Growth Factor Reviews 78 (2024): 105–119. 39004599 10.1016/j.cytogfr.2024.06.002

[202] J. Fang , Z. Wang , and C.‐Y. Miao , “Angiogenesis After ischemic stroke,” Acta Pharmacologica Sinica 44, no. 7 (2023): 1305–1321. 36829053 10.1038/s41401-023-01061-2 PMC10310733

[203] M. Hatakeyama , I. Ninomiya , and M. Kanazawa , “Angiogenesis and neuronal remodeling After ischemic stroke,” Neural Regeneration Research 15, no. 1 (2020): 16–19. 31535636 10.4103/1673-5374.264442 PMC6862417

[204] B. Campos , H. Choi , A. T. DeMarco , et al., “Rethinking Remapping: Circuit Mechanisms of Recovery After Stroke,” The Journal of Neuroscience: the Official Journal of the Society for Neuroscience 43, no. 45 (2023): 7489–7500. 37940595 10.1523/JNEUROSCI.1425-23.2023 PMC10634578

[205] R. G. Contreras , A. Torres‐Carrillo , C. Flores‐Maldonado , L. Shoshani , and A. Ponce , “Na(+)/K(+)‐ATPase: More Than an Electrogenic Pump,” International Journal of Molecular Sciences 25, no. 11 (2024): 6122. 38892309 10.3390/ijms25116122 PMC11172918

[206] M. Zhu , H. Sun , L. Cao , Z. Wu , B. Leng , and J. Bian , “Role of Na(+)/K(+)‐ATPase in ischemic stroke: In‐depth perspectives From physiology to pharmacology,” Journal of molecular medicine (Berlin) 100, no. 3 (2022): 395–410. 10.1007/s00109-021-02143-6 34839371

[207] P. Lipton , “Ischemic cell death in brain neurons,” Physiological Reviews 79, no. 4 (1999): 1431–1568. 10508238 10.1152/physrev.1999.79.4.1431

[208] S. A. Maiorov , B. K. Kairat , A. V. Berezhnov , V. P. Zinchenko , S. G. Gaidin , and A. M. Kosenkov , “Peculiarities of ion homeostasis in neurons containing calcium‐permeable AMPA receptors,” Archives of Biochemistry and Biophysics 754 (2024): 109951. 38452968 10.1016/j.abb.2024.109951

[209] S. Takaoka , R. D. Bart , R. Pearlstein , A. Brinkhous , and D. S. Warner , “Neuroprotective effect of NMDA receptor glycine recognition site antagonism persists when brain temperature is controlled,” Journal of Cerebral Blood Flow and Metabolism: Official Journal of the International Society of Cerebral Blood Flow and Metabolism 17, no. 2 (1997): 161–167. 9040495 10.1097/00004647-199702000-00005

[210] H. G. Martin and Y. T. Wang , “Blocking the deadly effects of the NMDA receptor in stroke,” Cell 140, no. 2 (2010): 174–176. 20141829 10.1016/j.cell.2010.01.014

[211] Z. Shen , M. Xiang , C. Chen , et al., “Glutamate excitotoxicity: Potential therapeutic target for ischemic stroke,” Biomedicine & Pharmacotherapy 151 (2022): 113125. 35609367 10.1016/j.biopha.2022.113125

[212] S. Colleoni , A. A. Jensen , E. Landucci , et al., “Neuroprotective effects of the novel glutamate transporter inhibitor (‐)‐3‐hydroxy‐4,5,6,6a‐tetrahydro‐3aH‐pyrrolo[3,4‐d]‐isoxazole‐4‐carboxylic acid, which preferentially inhibits reverse transport (glutamate release) compared With glutamate reuptake,” The Journal of Pharmacology and Experimental Therapeutics 326, no. 2 (2008): 646–656. 18451317 10.1124/jpet.107.135251

[213] D. Mayor and M. Tymianski , “Neurotransmitters in the mediation of cerebral ischemic injury,” Neuropharmacology 134, no. Pt B (2018): 178–188. 29203179 10.1016/j.neuropharm.2017.11.050

[214] M. J. Kim , A. W. Dunah , Y. T. Wang , and M. Sheng , “Differential roles of NR2A‐ and NR2B‐containing NMDA receptors in Ras‐ERK signaling and AMPA receptor trafficking,” Neuron 46, no. 5 (2005): 745–760. 15924861 10.1016/j.neuron.2005.04.031

[215] K. Ning , L. Pei , M. Liao , et al., “Dual neuroprotective signaling mediated by downregulating two distinct phosphatase activities of PTEN,” The Journal of Neuroscience: the Official Journal of the Society for Neuroscience 24, no. 16 (2004): 4052–4060. 15102920 10.1523/JNEUROSCI.5449-03.2004 PMC6729419

[216] A. L. González‐Cota , D. Martínez‐Flores , M. J. Rosendo‐Pineda , and L. Vaca , “NMDA receptor‐mediated Ca(2+) signaling: Impact on cell cycle regulation and the development of neurodegenerative diseases and cancer,” Cell Calcium 119 (2024): 102856. 38408411 10.1016/j.ceca.2024.102856

[217] T. W. Lai , S. Zhang , and Y. T. Wang , “Excitotoxicity and stroke: Identifying novel targets for neuroprotection,” Progress in Neurobiology 115 (2014): 157–188. 24361499 10.1016/j.pneurobio.2013.11.006

[218] G. N. Patrick , L. Zukerberg , M. Nikolic , S. de la Monte , P. Dikkes , and L. H. Tsai , “Conversion of p35 to p25 deregulates Cdk5 activity and promotes neurodegeneration,” Nature 402, no. 6762 (1999): 615–622. 10604467 10.1038/45159

[219] G. A. Rutter and I. R. Sweet , “Glucose Regulation of β‐Cell KATP Channels: Is a New Model Needed?,” Diabetes 73, no. 6 (2024): 849–855. 38768365 10.2337/dbi23-0031 PMC11109788

[220] V. Rahi and R. K. Kaundal , “Exploring the intricacies of calcium dysregulation in ischemic stroke: Insights Into neuronal cell death and therapeutic strategies,” Life Sciences 347 (2024): 122651. 38642844 10.1016/j.lfs.2024.122651

[221] A. Bhardwaj , R. Bhardwaj , A. Saini , D. K. Dhawan , and T. Kaur , “Impact of Calcium Influx on Endoplasmic Reticulum in Excitotoxic Neurons: Role of Chemical Chaperone 4‐PBA,” Cellular and Molecular Neurobiology 43, no. 4 (2023): 1619–1635. 36002608 10.1007/s10571-022-01271-y PMC11412423

[222] S. Khan , N. Bano , S. Ahamad , U. John , N. J. Dar , and S. A. Bhat , “Excitotoxicity, Oxytosis/Ferroptosis, and Neurodegeneration: Emerging Insights Into Mitochondrial Mechanisms,” Aging and Disease 16, no. 5 (2024): 2504–2543. 39122453 10.14336/AD.2024.0125-1 PMC12339096

[223] C. Remya , E. J. Variyar , R. V. Omkumar , C. Sadasivan , and K. V. Dileep , “Unveiling the molecular basis of lobeline's allosteric regulation of NMDAR: Insights From molecular modeling,” Scientific Reports 13, no. 1 (2023): 22418. 38104236 10.1038/s41598-023-49835-2 PMC10725453

[224] S. Guo , A. Wehbe , S. Syed , et al., “Cerebral Glucose Metabolism and Potential Effects on Endoplasmic Reticulum Stress in Stroke,” Aging and Disease 14, no. 2 (2023): 450–467. 37008060 10.14336/AD.2022.0905 PMC10017147

[225] L. Kong , H. Yang , J. Yang , et al., “Role of calcium overload‐mediated disruption of mitochondrial dynamics in offspring neurotoxicity due to methylmercury exposure During pregnancy and lactation,” Ecotoxicology and Environmental Safety 291 (2025): 117835. 39893884 10.1016/j.ecoenv.2025.117835

[226] Z. Li , Q. Ran , C. Qu , et al., “Sigma‐1 receptor activation attenuates DOX‐induced cardiotoxicity by alleviating endoplasmic reticulum stress and mitochondrial calcium overload via PERK and IP3R‐VDAC1‐MCU signaling pathways,” Biology Direct 20, no. 1 (2025): 23. 40001213 10.1186/s13062-025-00617-y PMC11853590

[227] F. Liu , J. Lu , A. Manaenko , J. Tang , and Q. Hu , “Mitochondria in Ischemic Stroke: New Insight and Implications,” Aging and Disease 9, no. 5 (2018): 924–937. 30271667 10.14336/AD.2017.1126 PMC6147588

[228] Y. W. Li , Y. Liu , S. Z. Luo , et al., “The significance of calcium ions in cerebral ischemia‐reperfusion injury: Mechanisms and intervention strategies,” Frontiers in Molecular Biosciences 12 (2025): 1585758. 40421420 10.3389/fmolb.2025.1585758 PMC12104078

[229] A. Jurcau and A. I. Ardelean , “Oxidative Stress in Ischemia/Reperfusion Injuries following Acute Ischemic Stroke,” Biomedicines 10, no. 3 (2022): 574. 35327376 10.3390/biomedicines10030574 PMC8945353

[230] P. J. Crack and J. M. Taylor , “Reactive oxygen species and the modulation of stroke,” Free Radical Biology and Medicine 38, no. 11 (2005): 1433–1444. 15890617 10.1016/j.freeradbiomed.2005.01.019

[231] D. B. Zorov , M. Juhaszova , and S. J. Sollott , “Mitochondrial reactive oxygen species (ROS) and ROS‐induced ROS release,” Physiological Reviews 94, no. 3 (2014): 909–950. 24987008 10.1152/physrev.00026.2013 PMC4101632

[232] C. L. Allen and U. Bayraktutan , “Oxidative stress and its role in the pathogenesis of ischaemic stroke,” International Journal of Stroke 4, no. 6 (2009): 461–470. 19930058 10.1111/j.1747-4949.2009.00387.x

[233] E. D. Hall , “Inhibition of lipid peroxidation in central nervous system trauma and ischemia,” Journal of the Neurological Sciences 134 (1995): 79–83. 8847548 10.1016/0022-510x(95)00211-j

[234] G. Stoll , S. Jander , and M. Schroeter , “Inflammation and glial responses in ischemic brain lesions,” Progress in Neurobiology 56, no. 2 (1998): 149–171. 9760699 10.1016/s0301-0082(98)00034-3

[235] E. Candelario‐Jalil , R. M. Dijkhuizen , and T. Magnus , “Neuroinflammation, Stroke, Blood‐Brain Barrier Dysfunction, and Imaging Modalities,” Stroke; A Journal of Cerebral Circulation 53, no. 5 (2022): 1473–1486. 10.1161/STROKEAHA.122.036946 PMC9038693 35387495

[236] S. W. Suh , B. S. Shin , H. Ma , et al., “Glucose and NADPH oxidase drive neuronal superoxide formation in stroke,” Annals of Neurology 64, no. 6 (2008): 654–663. 19107988 10.1002/ana.21511 PMC4304737

[237] G. W. Kim , T. Sugawara , and P. H. Chan , “Involvement of oxidative stress and caspase‐3 in cortical infarction After photothrombotic ischemia in mice,” Journal of Cerebral Blood Flow and Metabolism: Official Journal of the International Society of Cerebral Blood Flow and Metabolism 20, no. 12 (2000): 1690–1701. 11129785 10.1097/00004647-200012000-00008

[238] S. A. Saeed , K. F. Shad , T. Saleem , F. Javed , and M. U. Khan , “Some new prospects in the understanding of the molecular basis of the pathogenesis of stroke,” Experimental Brain Research 182, no. 1 (2007): 1–10. 17665180 10.1007/s00221-007-1050-9

[239] A. Kim , Y. J. Nam , Y. K. Shin , M. S. Lee , D. S. Sohn , and C. S. Lee , “Rotundarpene inhibits TNF‐α‐induced activation of the Akt, mTOR, and NF‐κB pathways, and the JNK and p38 associated With production of reactive oxygen species,” Molecular and Cellular Biochemistry 434, no. 1‐2 (2017): 113–125. 28432555 10.1007/s11010-017-3041-x

[240] H. Kamada , C. Nito , H. Endo , and P. H. Chan , “Bad as a converging signaling molecule Between survival PI3‐K/Akt and death JNK in neurons After transient focal cerebral ischemia in rats,” Journal of Cerebral Blood Flow and Metabolism: Official Journal of the International Society of Cerebral Blood Flow and Metabolism 27, no. 3 (2007): 521–533. 16820799 10.1038/sj.jcbfm.9600367 PMC1804097

[241] J. Feng , X. Chen , and J. Shen , “Reactive nitrogen species as therapeutic targets for autophagy: Implication for ischemic stroke,” Expert Opinion on Therapeutic Targets 21, no. 3 (2017): 305–317. 28081644 10.1080/14728222.2017.1281250

[242] A. Ledo , J. Frade , R. M. Barbosa , and J. Laranjinha , “Nitric oxide in brain: Diffusion, targets and concentration dynamics in hippocampal subregions,” Molecular Aspects of Medicine 25, no. 1‐2 (2004): 75–89. 15051318 10.1016/j.mam.2004.02.010

[243] J. Garthwaite , S. L. Charles , and R. Chess‐Williams , “Endothelium‐derived relaxing factor release on activation of NMDA receptors suggests role as intercellular messenger in the brain,” Nature 336, no. 6197 (1988): 385–388. 2904125 10.1038/336385a0

[244] A. Bhardwaj , F. J. Northington , R. N. Ichord , D. F. Hanley , R. J. Traystman , and R. C. Koehler , “Characterization of ionotropic glutamate receptor‐mediated nitric oxide production in vivo in rats,” Stroke; A Journal of Cerebral Circulation 28, no. 4 (1997): 850–856. discussion 856–7. 10.1161/01.str.28.4.850 9099207

[245] S. Love , “Oxidative stress in brain ischemia,” Brain Pathology 9, no. 1 (1999): 119–131. 9989455 10.1111/j.1750-3639.1999.tb00214.x PMC8098220

[246] S. Blanco , M. D. M. Muñoz‐Gallardo , R. Hernández , and M. Peinado , “The Interplay Between Melatonin and Nitric Oxide: Mechanisms and Implications in Stroke Pathophysiology,” Antioxidants (Basel) 14, no. 6 (2025): 724. 40563356 10.3390/antiox14060724 PMC12190141

[247] K. M. Nash , I. T. Schiefer , and Z. A. Shah , “Development of a reactive oxygen species‐sensitive nitric oxide synthase inhibitor for the treatment of ischemic stroke,” Free Radical Biology and Medicine 115 (2018): 395–404. 29275014 10.1016/j.freeradbiomed.2017.12.027 PMC11970191

[248] T. Li , T. Xu , J. Zhao , H. Gao , and W. Xie , “Depletion of iNOS‐positive inflammatory cells decelerates neuronal degeneration and alleviates cerebral ischemic damage by suppressing the inflammatory response,” Free Radical Biology and Medicine 181 (2022): 209–220. 35150825 10.1016/j.freeradbiomed.2022.02.008

[249] X. C. Ye , Q. Hao , W. J. Ma , et al., “Dectin‐1/Syk signaling triggers neuroinflammation After ischemic stroke in mice,” J Neuroinflammation 17, no. 1 (2020): 17. 31926564 10.1186/s12974-019-1693-z PMC6954534

[250] R. C. Kukreja and L. Xi , “eNOS phosphorylation: A pivotal molecular switch in vasodilation and cardioprotection?,” Journal of Molecular and Cellular Cardiology 42, no. 2 (2007): 280–282. 17174975 10.1016/j.yjmcc.2006.10.011 PMC3031790

[251] P. de la Riva , J. Marta‐Enguita , J. Rodríguez‐Antigüedad , A. Bergareche , and A. L. de Munain , “Understanding Endothelial Dysfunction and Its Role in Ischemic Stroke After the Outbreak of Recanalization Therapies,” International Journal of Molecular Sciences 25, no. 21 (2024): 11631. 39519182 10.3390/ijms252111631 PMC11546609

[252] M. Bladowski , J. Gawrys , D. Gajecki , E. Szahidewicz‐Krupska , A. Sawicz‐Bladowska , and A. Doroszko , “Role of the Platelets and Nitric Oxide Biotransformation in Ischemic Stroke: A Translative Review From Bench to Bedside,” Oxidative Medicine and Cellular Longevity 2020 (2020): 2979260. 32908630 10.1155/2020/2979260 PMC7474795

[253] M. Khan , T. S. Dhammu , F. Qiao , P. Kumar , A. K. Singh , and I. Singh , “S‐Nitrosoglutathione Mimics the Beneficial Activity of Endothelial Nitric Oxide Synthase‐Derived Nitric Oxide in a Mouse Model of Stroke,” Journal of Stroke and Cerebrovascular Diseases: the Official Journal of National Stroke Association 28, no. 12 (2019): 104470. 31680031 10.1016/j.jstrokecerebrovasdis.2019.104470

[254] Y. Cao , X. Yue , M. Jia , and J. Wang , “Neuroinflammation and anti‐inflammatory therapy for ischemic stroke,” Heliyon 9, no. 7 (2023): e17986. 37519706 10.1016/j.heliyon.2023.e17986 PMC10372247

[255] E. Gülke , M. Gelderblom , and T. Magnus , “Danger signals in stroke and their role on microglia activation After ischemia,” Therapeutic Advances in Neurological Disorders 11 (2018): 1756286418774254. 29854002 10.1177/1756286418774254 PMC5968660

[256] A. Tuttolomondo , D. Di Raimondo , R. di Sciacca , A. Pinto , and G. Licata , “Inflammatory cytokines in acute ischemic stroke,” Current Pharmaceutical Design 14, no. 33 (2008): 3574–3589. 19075734 10.2174/138161208786848739

[257] Q. Wang , X. N. Tang , and M. A. Yenari , “The inflammatory response in stroke,” Journal of Neuroimmunology 184, no. 1‐2 (2007): 53–68. 17188755 10.1016/j.jneuroim.2006.11.014 PMC1868538

[258] M. Gelderblom , F. Leypoldt , K. Steinbach , et al., “Temporal and spatial dynamics of cerebral immune cell accumulation in stroke,” Stroke; A Journal of Cerebral Circulation 40, no. 5 (2009): 1849–1857. 10.1161/STROKEAHA.108.534503 19265055

[259] G. J. del Zoppo , R. Milner , T. Mabuchi , et al., “Microglial activation and matrix protease generation During focal cerebral ischemia,” Stroke; A Journal of Cerebral Circulation 38, no. 2 Suppl (2007): 646–651. 10.1161/01.STR.0000254477.34231.cb 17261708

[260] F. Takata , S. Nakagawa , J. Matsumoto , and S. Dohgu , “Blood‐Brain Barrier Dysfunction Amplifies the Development of Neuroinflammation: Understanding of Cellular Events in Brain Microvascular Endothelial Cells for Prevention and Treatment of BBB Dysfunction,” Frontiers in Cellular Neuroscience 15 (2021): 661838. 34588955 10.3389/fncel.2021.661838 PMC8475767

[261] X. Ye , T. Shen , J. Hu , et al., “Purinergic 2×7 receptor/NLRP3 pathway triggers neuronal apoptosis After ischemic stroke in the mouse,” Experimental Neurology 292 (2017): 46–55. 28274860 10.1016/j.expneurol.2017.03.002

[262] T. Suzuki , K. Kohyama , K. Moriyama , et al., “Extracellular ADP augments microglial inflammasome and NF‐κB activation via the P2Y12 receptor,” European Journal of Immunology 50, no. 2 (2020): 205–219. 31549730 10.1002/eji.201848013

[263] A. Rifat , B. Ossola , R. W. Bürli , et al., “Differential contribution of THIK‐1 K(+) channels and P2×7 receptors to ATP‐mediated neuroinflammation by human microglia,” Journal of Neuroinflammation 21, no. 1 (2024): 58. 38409076 10.1186/s12974-024-03042-6 PMC10895799

[264] R. Kang , M. Gamdzyk , C. Lenahan , J. Tang , S. Tan , and J. H. Zhang , “The Dual Role of Microglia in Blood‐Brain Barrier Dysfunction After Stroke,” Current Neuropharmacology 18, no. 12 (2020): 1237–1249. 32469699 10.2174/1570159X18666200529150907 PMC7770642

[265] M. Xie , Y. Hao , L. Feng , et al., “Neutrophil Heterogeneity and its Roles in the Inflammatory Network After Ischemic Stroke,” Current Neuropharmacology 21, no. 3 (2023): 621–650. 35794770 10.2174/1570159X20666220706115957 PMC10207908

[266] C. V. Carman and R. Martinelli , “T Lymphocyte‐Endothelial Interactions: Emerging Understanding of Trafficking and Antigen‐Specific Immunity,” Frontiers in immunology 6 (2015): 603. 26635815 10.3389/fimmu.2015.00603 PMC4657048

[267] J. Ruhnau , J. Schulze , A. Dressel , and A. Vogelgesang , “Thrombosis, Neuroinflammation, and Poststroke Infection: The Multifaceted Role of Neutrophils in Stroke,” Journal of Immunology Research 2017 (2017): 5140679. 28331857 10.1155/2017/5140679 PMC5346374

[268] A. Mohamud Yusuf , N. Hagemann , P. Ludewig , M. Gunzer , and D. M. Hermann , “Roles of Polymorphonuclear Neutrophils in Ischemic Brain Injury and Post‐Ischemic Brain Remodeling,” Frontiers in immunology 12 (2021): 825572. 35087539 10.3389/fimmu.2021.825572 PMC8787127

[269] I. A. Krizbai , H. Bauer , N. Bresgen , et al., “Effect of oxidative stress on the junctional proteins of cultured cerebral endothelial cells,” Cellular and Molecular Neurobiology 25, no. 1 (2005): 129–139. 15962510 10.1007/s10571-004-1378-7 PMC11529493

[270] F. Arba , C. Rinaldi , D. Caimano , F. Vit , G. Busto , and E. Fainardi , “Blood‐Brain Barrier Disruption and Hemorrhagic Transformation in Acute Ischemic Stroke: Systematic Review and Meta‐Analysis,” Frontiers in Neurology 11 (2020): 594613. 33551955 10.3389/fneur.2020.594613 PMC7859439

[271] L. Kang , H. Yu , X. Yang , et al., “Neutrophil extracellular traps released by neutrophils impair revascularization and vascular remodeling After stroke,” Nature Communications 11, no. 1 (2020): 2488. 10.1038/s41467-020-16191-y PMC7237502 32427863

[272] C. Li , Y. Xing , Y. Zhang , Y. Hua , J. Hu , and Y. Bai , “Neutrophil Extracellular Traps Exacerbate Ischemic Brain Damage,” Molecular Neurobiology 59, no. 1 (2022): 643–656. 34748205 10.1007/s12035-021-02635-z

[273] C. Chen , T. Huang , X. Zhai , et al., “Targeting neutrophils as a novel therapeutic strategy After stroke,” Journal of Cerebral Blood Flow and Metabolism: Official Journal of the International Society of Cerebral Blood Flow and Metabolism 41, no. 9 (2021): 2150–2161. 33691513 10.1177/0271678X211000137 PMC8393299

[274] Q. Z. Tuo , S. T. Zhang , and P. Lei , “Mechanisms of neuronal cell death in ischemic stroke and their therapeutic implications,” Medicinal Research Reviews 42, no. 1 (2022): 259–305. 33957000 10.1002/med.21817

[275] W. T. Yan , Y. D. Yang , X. M. Hu , et al., “Do pyroptosis, apoptosis, and necroptosis (PANoptosis) exist in cerebral ischemia? Evidence From cell and rodent studies,” Neural Regeneration Research 17, no. 8 (2022): 1761–1768. 35017436 10.4103/1673-5374.331539 PMC8820688

[276] Z. Zhu , M. Song , J. Ren , L. Liang , G. Mao , and M. Chen , “Copper homeostasis and cuproptosis in central nervous system diseases,” Cell death & disease 15, no. 11 (2024): 850. 39567497 10.1038/s41419-024-07206-3 PMC11579297

[277] B. R. Broughton , D. C. Reutens , and C. G. Sobey , “Apoptotic mechanisms After cerebral ischemia,” Stroke; A Journal of Cerebral Circulation 40, no. 5 (2009): e331–e339. 10.1161/STROKEAHA.108.531632 19182083

[278] J. F. Kerr , A. H. Wyllie , and A. R. Currie , “Apoptosis: A basic biological phenomenon With wide‐ranging implications in tissue kinetics,” British Journal of Cancer 26, no. 4 (1972): 239–257. 4561027 10.1038/bjc.1972.33 PMC2008650

[279] S. C. Benn and C. J. Woolf , “Adult neuron survival strategies–slamming on the brakes,” Nature Reviews Neuroscience 5, no. 9 (2004): 686–700. 15322527 10.1038/nrn1477

[280] A. Datta , D. Sarmah , L. Mounica , et al., “Cell Death Pathways in Ischemic Stroke and Targeted Pharmacotherapy,” Translational Stroke Research 11, no. 6 (2020): 1185–1202. 32219729 10.1007/s12975-020-00806-z

[281] H. Zou , W. J. Henzel , X. Liu , A. Lutschg , and X. Wang , “Apaf‐1, a human protein homologous to C. elegans CED‐4, participates in cytochrome c‐dependent activation of caspase‐3,” Cell 90, no. 3 (1997): 405–413. 9267021 10.1016/s0092-8674(00)80501-2

[282] S. Namura , J. Zhu , K. Fink , et al., “Activation and cleavage of caspase‐3 in apoptosis induced by experimental cerebral ischemia,” The Journal of Neuroscience: the Official Journal of the Society for Neuroscience 18, no. 10 (1998): 3659–3668. 9570797 10.1523/JNEUROSCI.18-10-03659.1998 PMC6793169

[283] M. Endres , Z. Q. Wang , S. Namura , C. Waeber , and M. A. Moskowitz , “Ischemic brain injury is mediated by the activation of poly(ADP‐ribose)polymerase,” Journal of Cerebral Blood Flow and Metabolism: Official Journal of the International Society of Cerebral Blood Flow and Metabolism 17, no. 11 (1997): 1143–1151. 9390645 10.1097/00004647-199711000-00002

[284] N. Plesnila , S. Zinkel , S. Amin‐Hanjani , J. Qiu , S. J. Korsmeyer , and M. A. Moskowitz , “Function of BID – a molecule of the bcl‐2 family – in ischemic cell death in the brain,” European Surgical Research Europaische Chirurgische Forschung Recherches Chirurgicales Europeennes 34, no. 1‐2 (2002): 37–41. 11867899 10.1159/000048885

[285] U. Dirnagl , C. Iadecola , and M. A. Moskowitz , “Pathobiology of Ischaemic Stroke: an Integrated View,” Trends in Neurosciences 22, no. 9 (1999): 391–397. 10441299 10.1016/s0166-2236(99)01401-0

[286] J. M. Simard , K. V. Tarasov , and V. Gerzanich , “Non‐selective cation channels, transient receptor potential channels and ischemic stroke,” Biochimica Et Biophysica Acta 1772, no. 8 (2007): 947–957. 17446049 10.1016/j.bbadis.2007.03.004 PMC1986778

[287] C. Culmsee , C. Zhu , S. Landshamer , et al., “Apoptosis‐inducing factor triggered by poly(ADP‐ribose) polymerase and Bid mediates neuronal cell death After oxygen‐glucose deprivation and focal cerebral ischemia,” The Journal of Neuroscience: the Official Journal of the Society for Neuroscience 25, no. 44 (2005): 10262–10272. 16267234 10.1523/JNEUROSCI.2818-05.2005 PMC6725791

[288] S. Love , “Apoptosis and brain ischaemia,” Progress in neuro‐psychopharmacology & Biological Psychiatry 27, no. 2 (2003): 267–282. 12657366 10.1016/S0278-5846(03)00022-8

[289] K. A. Webster , R. M. Graham , J. W. Thompson , et al., “Redox stress and the contributions of BH3‐only proteins to infarction,” Antioxidants & Redox Signaling 8, no. 9‐10 (2006): 1667–1676. 16987020 10.1089/ars.2006.8.1667

[290] A. Saito , T. Hayashi , S. Okuno , M. Ferrand‐Drake , and P. H. Chan , “Overexpression of copper/zinc superoxide dismutase in transgenic mice protects Against neuronal cell death After transient focal ischemia by blocking activation of the Bad cell death signaling pathway,” The Journal of Neuroscience: the Official Journal of the Society for Neuroscience 23, no. 5 (2003): 1710–1718. 12629175 10.1523/JNEUROSCI.23-05-01710.2003 PMC6741974

[291] R. A. Kirkland , J. A. Windelborn , J. M. Kasprzak , and J. L. Franklin , “A Bax‐induced pro‐oxidant state is critical for cytochrome c release During programmed neuronal death,” The Journal of Neuroscience: the Official Journal of the Society for Neuroscience 22, no. 15 (2002): 6480–6490. 12151527 10.1523/JNEUROSCI.22-15-06480.2002 PMC6758153

[292] J. L. Franklin , “Redox regulation of the intrinsic pathway in neuronal apoptosis,” Antioxidants & Redox Signaling 14, no. 8 (2011): 1437–1448. 20812874 10.1089/ars.2010.3596 PMC3061193

[293] B. B. Cho and L. H. Toledo‐Pereyra , “Caspase‐independent programmed cell death following ischemic stroke,” Journal of Investigative Surgery: the Official Journal of the Academy of Surgical Research 21, no. 3 (2008): 141–147. 18569435 10.1080/08941930802029945

[294] Q. Wang , F. Yang , K. Duo , et al., “The Role of Necroptosis in Cerebral Ischemic Stroke,” Molecular Neurobiology 61, no. 7 (2024): 3882–3898. 38038880 10.1007/s12035-023-03728-7

[295] A. Kaczmarek , P. Vandenabeele , and D. V. Krysko , “Necroptosis: The release of damage‐associated molecular patterns and its physiological relevance,” Immunity 38, no. 2 (2013): 209–223. 23438821 10.1016/j.immuni.2013.02.003

[296] Y. Ni , W. W. Gu , Z. H. Liu , et al., “RIP1K Contributes to Neuronal and Astrocytic Cell Death in Ischemic Stroke via Activating Autophagic‐lysosomal Pathway,” Neuroscience 371 (2018): 60–74. 29102662 10.1016/j.neuroscience.2017.10.038

[297] X. X. Deng , S. S. Li , and F. Y. Sun , “Necrostatin‐1 Prevents Necroptosis in Brains After Ischemic Stroke via Inhibition of RIPK1‐Mediated RIPK3/MLKL Signaling,” Aging and Disease 10, no. 4 (2019): 807–817. 31440386 10.14336/AD.2018.0728 PMC6675533

[298] M. G. Naito , D. Xu , P. Amin , et al., “Sequential activation of necroptosis and apoptosis cooperates to mediate vascular and neural pathology in stroke,” PNAS 117, no. 9 (2020): 4959–4970. 32071228 10.1073/pnas.1916427117 PMC7060720

[299] B. Gerlach , S. M. Cordier , A. C. Schmukle , et al., “Linear ubiquitination prevents inflammation and regulates immune signalling,” Nature 471, no. 7340 (2011): 591–596. 21455173 10.1038/nature09816

[300] G. Chen and D. V. Goeddel , “TNF‐R1 signaling: A beautiful pathway,” Science (New York, NY) 296, no. 5573 (2002): 1634–1635. 10.1126/science.1071924 12040173

[301] O. Micheau and J. Tschopp , “Induction of TNF receptor I‐mediated apoptosis via two sequential signaling complexes,” Cell 114, no. 2 (2003): 181–190. 12887920 10.1016/s0092-8674(03)00521-x

[302] Y. S. Cho , S. Challa , D. Moquin , et al., “Phosphorylation‐driven assembly of the RIP1‐RIP3 complex regulates programmed necrosis and virus‐induced inflammation,” Cell 137, no. 6 (2009): 1112–1123. 19524513 10.1016/j.cell.2009.05.037 PMC2727676

[303] L. Sun , H. Wang , Z. Wang , et al., “Mixed lineage kinase domain‐Like protein mediates necrosis signaling downstream of RIP3 kinase,” Cell 148, no. 1‐2 (2012): 213–227. 22265413 10.1016/j.cell.2011.11.031

[304] Y. Zhang , M. Li , X. Li , et al., “Catalytically inactive RIP1 and RIP3 deficiency protect Against acute ischemic stroke by inhibiting necroptosis and neuroinflammation,” Cell death & disease 11, no. 7 (2020): 565. 32703968 10.1038/s41419-020-02770-w PMC7378260

[305] V. Hribljan , D. Lisjak , D. J. Petrović , and D. Mitrečić , “Necroptosis is one of the modalities of cell death accompanying ischemic brain stroke: From pathogenesis to therapeutic possibilities,” Croatian Medical Journal 60, no. 2 (2019): 121–126. 31044583 10.3325/cmj.2019.60.121 PMC6509625

[306] Y. Tang , Q. Chu , G. Xie , Y. Tan , Z. Ye , and C. Qin , “MLKL regulates Cx43 ubiquitinational degradation and mediates neuronal necroptosis in ipsilateral thalamus After focal cortical infarction,” Molecular Brain 16, no. 1 (2023): 74. 37904209 10.1186/s13041-023-01064-4 PMC10617209

[307] Z. Cai , S. Jitkaew , J. Zhao , et al., “Plasma membrane translocation of trimerized MLKL protein is required for TNF‐induced necroptosis,” Nature Cell Biology 16, no. 1 (2014): 55–65. 24316671 10.1038/ncb2883 PMC8369836

[308] X. Chen , W. Li , J. Ren , et al., “Translocation of mixed lineage kinase domain‐Like protein to plasma membrane leads to necrotic cell death,” Cell Research 24, no. 1 (2014): 105–121. 24366341 10.1038/cr.2013.171 PMC3879712

[309] Y. Hu , H. Lei , S. Zhang , et al., “Panax notoginseng Saponins Protect Brain Microvascular Endothelial Cells Against Oxygen‐Glucose Deprivation/Resupply‐Induced Necroptosis via Suppression of RIP1‐RIP3‐MLKL Signaling Pathway,” Neurochemical Research 47, no. 11 (2022): 3261–3271. 35904697 10.1007/s11064-022-03675-0

[310] J. M. Murphy , P. E. Czabotar , J. M. Hildebrand , et al., “The pseudokinase MLKL mediates necroptosis via a molecular switch mechanism,” Immunity 39, no. 3 (2013): 443–453. 24012422 10.1016/j.immuni.2013.06.018

[311] Y. Zhou , J. Liao , Z. Mei , X. Liu , and J. Ge , “Insight Into Crosstalk Between Ferroptosis and Necroptosis: Novel Therapeutics in Ischemic Stroke,” Oxidative Medicine and Cellular Longevity 2021 (2021): 9991001. 34257829 10.1155/2021/9991001 PMC8257382

[312] L. Yang , J. Cheng , G. Shi , et al., “Liraglutide Ameliorates Cerebral Ischemia in Mice via Antipyroptotic Pathways,” Neurochemical Research 47, no. 7 (2022): 1904–1916. 35352213 10.1007/s11064-022-03574-4

[313] J. Long , Y. Sun , S. Liu , et al., “Targeting pyroptosis as a preventive and therapeutic approach for stroke,” Cell Death Discovery 9, no. 1 (2023): 155. 37165005 10.1038/s41420-023-01440-y PMC10172388

[314] M. Di Gioia , R. Spreafico , J. R. Springstead , et al., “Endogenous oxidized phospholipids reprogram cellular metabolism and boost hyperinflammation,” Nature Immunology 21, no. 1 (2020): 42–53. 31768073 10.1038/s41590-019-0539-2 PMC6923570

[315] A. Talty , S. Deegan , M. Ljujic , et al., “Inhibition of IRE1α RNase activity reduces NLRP3 inflammasome assembly and processing of pro‐IL1β,” Cell death & disease 10, no. 9 (2019): 622. 31417078 10.1038/s41419-019-1847-z PMC6695440

[316] S. Chen , S. Mei , Y. Luo , H. Wu , J. Zhang , and J. Zhu , “Gasdermin Family: A Promising Therapeutic Target for Stroke,” Translational Stroke Research 9, no. 6 (2018): 555–563. 30280366 10.1007/s12975-018-0666-3

[317] M. Fricker , A. M. Tolkovsky , V. Borutaite , M. Coleman , and G. C. Brown , “Neuronal Cell Death,” Physiological Reviews 98, no. 2 (2018): 813–880. 29488822 10.1152/physrev.00011.2017 PMC5966715

[318] L. Sborgi , S. Rühl , E. Mulvihill , et al., “GSDMD membrane pore formation constitutes the mechanism of pyroptotic cell death,” Embo Journal 35, no. 16 (2016): 1766–1778. 27418190 10.15252/embj.201694696 PMC5010048

[319] A. Linder , S. Bauernfried , Y. Cheng , et al., “CARD8 inflammasome activation triggers pyroptosis in human T cells,” Embo Journal 39, no. 19 (2020): e105071. 32840892 10.15252/embj.2020105071 PMC7527815

[320] J. J. Chae , Y. H. Cho , G. S. Lee , et al., “Gain‐of‐function Pyrin mutations induce NLRP3 protein‐independent interleukin‐1β activation and severe autoinflammation in mice,” Immunity 34, no. 5 (2011): 755–768. 21600797 10.1016/j.immuni.2011.02.020 PMC3129608

[321] L. I. Labzin , M. Bottermann , P. Rodriguez‐Silvestre , et al., “Antibody and DNA sensing pathways converge to activate the inflammasome During primary human macrophage infection,” Embo Journal 38, no. 21 (2019): e101365. 31468569 10.15252/embj.2018101365 PMC6826209

[322] J. Ding , K. Wang , W. Liu , et al., “Pore‐forming activity and structural autoinhibition of the gasdermin family,” Nature 535, no. 7610 (2016): 111–116. 27281216 10.1038/nature18590

[323] N. Kayagaki , S. Warming , M. Lamkanfi , et al., “Non‐canonical inflammasome activation targets caspase‐11,” Nature 479, no. 7371 (2011): 117–121. 22002608 10.1038/nature10558

[324] S. Mariathasan and D. M. Monack , “Inflammasome adaptors and sensors: Intracellular regulators of infection and inflammation,” Nature Reviews Immunology 7, no. 1 (2007): 31–40. 10.1038/nri1997 17186029

[325] Z. Zhou , H. He , K. Wang , et al., “Granzyme A From cytotoxic lymphocytes cleaves GSDMB to trigger pyroptosis in target cells,” Science (New York, NY) 368, no. 6494 (2020): eaaz7548. 10.1126/science.aaz7548 32299851

[326] W. Deng , Y. Bai , F. Deng , et al., “Streptococcal pyrogenic exotoxin B cleaves GSDMA and triggers pyroptosis,” Nature 602, no. 7897 (2022): 496–502. 35110732 10.1038/s41586-021-04384-4 PMC9703647

[327] J. Hou , R. Zhao , W. Xia , et al., “PD‐L1‐mediated gasdermin C expression switches apoptosis to pyroptosis in cancer cells and facilitates tumour necrosis,” Nature Cell Biology 22, no. 10 (2020): 1264–1275. 32929201 10.1038/s41556-020-0575-z PMC7653546

[328] R. Mandal , J. C. Barrón , I. Kostova , S. Becker , and K. Strebhardt , “Caspase‐8: The double‐edged sword,” Biochimica Et Biophysica Acta Reviews on Cancer 1873, no. 2 (2020): 188357. 32147543 10.1016/j.bbcan.2020.188357

[329] L. E. Clarke , S. A. Liddelow , C. Chakraborty , A. E. Münch , M. Heiman , and B. A. Barres , “Normal aging induces A1‐Like astrocyte reactivity,” PNAS 115, no. 8 (2018): E1896–e1905. 29437957 10.1073/pnas.1800165115 PMC5828643

[330] A. Y. Baev , A. Y. Vinokurov , I. N. Novikova , V. V. Dremin , E. V. Potapova , and A. Y. Abramov , “Interaction of Mitochondrial Calcium and ROS in Neurodegeneration,” Cells 11, no. 4 (2022): 706. 35203354 10.3390/cells11040706 PMC8869783

[331] H. Wang , H. Chen , J. Jin , Q. Liu , D. Zhong , and G. Li , “Inhibition of the NLRP3 inflammasome reduces brain edema and regulates the distribution of aquaporin‐4 After cerebral ischaemia‐reperfusion,” Life Sciences 251 (2020): 117638. 32251636 10.1016/j.lfs.2020.117638

[332] P. Mulica , A. Grünewald , and S. L. Pereira , “Astrocyte‐Neuron Metabolic Crosstalk in Neurodegeneration: A Mitochondrial Perspective,” Frontiers in Endocrinology 12 (2021): 668517. 34025580 10.3389/fendo.2021.668517 PMC8138625

[333] X. Li , Z. Yu , W. Zong , et al., “Deficiency of the microglial Hv1 proton channel attenuates neuronal pyroptosis and inhibits inflammatory reaction After spinal cord injury,” Journal of Neuroinflammation 17, no. 1 (2020): 263. 32891159 10.1186/s12974-020-01942-x PMC7487532

[334] P. Xu , C. Tao , Y. Zhu , et al., “TAK1 mediates neuronal pyroptosis in early brain injury After subarachnoid hemorrhage,” Journal of Neuroinflammation 18, no. 1 (2021): 188. 34461942 10.1186/s12974-021-02226-8 PMC8406585

[335] B. Yuan , X. M. Zhou , Z. Q. You , et al., “Inhibition of AIM2 inflammasome activation alleviates GSDMD‐induced pyroptosis in early brain injury After subarachnoid haemorrhage,” Cell death & disease 11, no. 1 (2020): 76. 32001670 10.1038/s41419-020-2248-z PMC6992766

[336] C. R. Lammert , E. L. Frost , C. E. Bellinger , et al., “AIM2 inflammasome surveillance of DNA damage shapes neurodevelopment,” Nature 580, no. 7805 (2020): 647–652. 32350463 10.1038/s41586-020-2174-3 PMC7788527

[337] J. K. Y. Yap , B. S. Pickard , E. W. L. Chan , and S. Y. Gan , “The Role of Neuronal NLRP1 Inflammasome in Alzheimer's Disease: Bringing Neurons Into the Neuroinflammation Game,” Molecular Neurobiology 56, no. 11 (2019): 7741–7753. 31111399 10.1007/s12035-019-1638-7

[338] J. Yan , W. Xu , C. Lenahan , et al., “CCR5 Activation Promotes NLRP1‐Dependent Neuronal Pyroptosis via CCR5/PKA/CREB Pathway After Intracerebral Hemorrhage,” Stroke; A Journal of Cerebral Circulation 52, no. 12 (2021): 4021–4032. 10.1161/STROKEAHA.120.033285 PMC8607924 34719258

[339] R. Gu , L. Wang , H. Zhou , et al., “Rh‐CXCL‐12 Attenuates Neuronal Pyroptosis After Subarachnoid Hemorrhage in Rats via Regulating the CXCR4/NLRP1 Pathway,” Oxidative Medicine and Cellular Longevity 2021 (2021): 6966394. 34795842 10.1155/2021/6966394 PMC8595028

[340] Y. Shi , L. Zhang , H. Pu , et al., “Rapid endothelial cytoskeletal reorganization enables early blood‐brain barrier disruption and long‐term ischaemic reperfusion brain injury,” Nature Communications 7 (2016): 10523. 10.1038/ncomms10523 PMC4737895 26813496

[341] J. L. Chen , W. J. Duan , S. Luo , et al., “Ferulic acid attenuates brain microvascular endothelial cells damage caused by oxygen‐glucose deprivation via punctate‐mitochondria‐dependent mitophagy,” Brain Research 1666 (2017): 17–26. 28438530 10.1016/j.brainres.2017.04.006

[342] S. J. Forrester , D. S. Kikuchi , M. S. Hernandes , Q. Xu , and K. K. Griendling , “Reactive Oxygen Species in Metabolic and Inflammatory Signaling,” Circulation Research 122, no. 6 (2018): 877–902. 29700084 10.1161/CIRCRESAHA.117.311401 PMC5926825

[343] T. M. Bui , H. L. Wiesolek , and R. Sumagin , “ICAM‐1: A master regulator of cellular responses in inflammation, injury resolution, and tumorigenesis,” Journal of Leukocyte Biology 108, no. 3 (2020): 787–799. 32182390 10.1002/JLB.2MR0220-549R PMC7977775

[344] X. Jiang , B. R. Stockwell , and M. Conrad , “Ferroptosis: Mechanisms, biology and role in disease,” Nature Reviews Molecular Cell Biology 22, no. 4 (2021): 266–282. 33495651 10.1038/s41580-020-00324-8 PMC8142022

[345] Y. Xu , K. Li , Y. Zhao , L. Zhou , Y. Liu , and J. Zhao , “Role of Ferroptosis in Stroke,” Cellular and Molecular Neurobiology 43, no. 1 (2023): 205–222. 35102454 10.1007/s10571-022-01196-6 PMC11415219

[346] Y. Kondo , N. Ogawa , M. Asanuma , Z. Ota , and A. Mori , “Regional differences in late‐onset iron deposition, ferritin, transferrin, astrocyte proliferation, and microglial activation After transient forebrain ischemia in rat brain,” Journal of Cerebral Blood Flow and Metabolism: Official Journal of the International Society of Cerebral Blood Flow and Metabolism 15, no. 2 (1995): 216–226. 7860655 10.1038/jcbfm.1995.27

[347] J. Liu , Z. N. Guo , X. L. Yan , et al., “Crosstalk Between Autophagy and Ferroptosis and Its Putative Role in Ischemic Stroke,” Frontiers in Cellular Neuroscience 14 (2020): 577403. 33132849 10.3389/fncel.2020.577403 PMC7566169

[348] D. C. Lipscomb , L. G. Gorman , R. J. Traystman , and P. D. Hurn , “Low molecular weight iron in cerebral ischemic acidosis in vivo,” Stroke; A Journal of Cerebral Circulation 29, no. 2 (1998): 487–492. discussion 493. 10.1161/01.str.29.2.487 9472894

[349] M. H. Selim and R. R. Ratan , “The role of iron neurotoxicity in ischemic stroke,” Ageing Research Reviews 3, no. 3 (2004): 345–353. 15231241 10.1016/j.arr.2004.04.001

[350] T. Ganz , “Cellular iron: Ferroportin is the only way out,” Cell Metabolism 1, no. 3 (2005): 155–157. 16054057 10.1016/j.cmet.2005.02.005

[351] L. Li , Y. W. Li , J. Y. Zhao , Y. Z. Liu , and C. Holscher , “Quantitative analysis of iron concentration and expression of ferroportin 1 in the cortex and hippocampus of rats induced by cerebral ischemia,” Journal of Clinical Neuroscience: Official Journal of the Neurosurgical Society of Australasia 16, no. 11 (2009): 1466–1472. 19766498 10.1016/j.jocn.2009.01.020

[352] D. A. Stoyanovsky , Y. Y. Tyurina , I. Shrivastava , et al., “Iron catalysis of lipid peroxidation in ferroptosis: Regulated enzymatic or random free radical reaction?,” Free Radical Biology and Medicine 133 (2019): 153–161. 30217775 10.1016/j.freeradbiomed.2018.09.008 PMC6555767

[353] K. van Leyen , T. R. Holman , and D. J. Maloney , “The potential of 12/15‐lipoxygenase inhibitors in stroke therapy,” Future Medicinal Chemistry 6, no. 17 (2014): 1853–1855. 25495979 10.4155/fmc.14.129 PMC4280907

[354] P. Lebrero , A. M. Astudillo , J. M. Rubio , et al., “Cellular Plasmalogen Content Does Not Influence Arachidonic Acid Levels or Distribution in Macrophages: A Role for Cytosolic Phospholipase A(2)γ in Phospholipid Remodeling,” Cells 8, no. 8 (2019): 799. 31370188 10.3390/cells8080799 PMC6721556

[355] G. Pérez‐Chacón , A. M. Astudillo , V. Ruipérez , M. A. Balboa , and J. Balsinde , “Signaling role for lysophosphatidylcholine acyltransferase 3 in receptor‐regulated arachidonic acid reacylation reactions in human monocytes,” Journal of Immunology (Baltimore, Md: 1950) 184, no. 2 (2010): 1071–1078. 20018618 10.4049/jimmunol.0902257

[356] K. Kishimoto , R. C. Li , J. Zhang , et al., “Cytosolic phospholipase A2 alpha amplifies early cyclooxygenase‐2 expression, oxidative stress and MAP kinase phosphorylation After cerebral ischemia in mice,” J Neuroinflammation 7 (2010): 42. 20673332 10.1186/1742-2094-7-42 PMC2923122

[357] C. Gubern , S. Camós , I. Ballesteros , et al., “miRNA expression is modulated Over time After focal ischaemia: Up‐regulation of miR‐347 promotes neuronal apoptosis,” The FEBS Journal 280, no. 23 (2013): 6233–6246. 24112606 10.1111/febs.12546

[358] Y. Cui , Y. Zhang , X. Zhao , et al., “ACSL4 exacerbates ischemic stroke by promoting ferroptosis‐induced brain injury and neuroinflammation,” Brain, Behavior, and Immunity 93 (2021): 312–321. 33444733 10.1016/j.bbi.2021.01.003

[359] K. D'Herde and D. V. Krysko , “Ferroptosis: Oxidized PEs trigger death,” Nature Chemical Biology 13, no. 1 (2017): 4–5. 27842067 10.1038/nchembio.2261

[360] M. P. Mattson , “Roles of the lipid peroxidation product 4‐hydroxynonenal in obesity, the metabolic syndrome, and associated vascular and neurodegenerative disorders,” Experimental Gerontology 44, no. 10 (2009): 625–633. 19622391 10.1016/j.exger.2009.07.003 PMC2753676

[361] L. Zhang , X. Y. Bai , K. Y. Sun , et al., “A New Perspective in the Treatment of Ischemic Stroke: Ferroptosis,” Neurochemical Research 49, no. 4 (2024): 815–833. 38170383 10.1007/s11064-023-04096-3

[362] J. Guo , Q.‐Z. Tuo , and P. Lei , “Iron, ferroptosis, and ischemic stroke,” Journal of Neurochemistry 165, no. 4 (2023): 487–520. 36908209 10.1111/jnc.15807

[363] X. Guan , Z. Li , S. Zhu , et al., “Galangin attenuated cerebral ischemia‐reperfusion injury by inhibition of ferroptosis Through activating the SLC7A11/GPX4 axis in gerbils,” Life Sciences 264 (2021): 118660. 33127512 10.1016/j.lfs.2020.118660

[364] Y. Shi , L. Han , X. Zhang , L. Xie , P. Pan , and F. Chen , “Selenium Alleviates Cerebral Ischemia/Reperfusion Injury by Regulating Oxidative Stress, Mitochondrial Fusion and Ferroptosis,” Neurochemical Research 47, no. 10 (2022): 2992–3002. 35725978 10.1007/s11064-022-03643-8 PMC9470641

[365] K. Zhu , X. Zhu , S. Liu , J. Yu , S. Wu , and M. Hei , “Glycyrrhizin Attenuates Hypoxic‐Ischemic Brain Damage by Inhibiting Ferroptosis and Neuroinflammation in Neonatal Rats via the HMGB1/GPX4 Pathway,” Oxidative Medicine and Cellular Longevity 2022 (2022): 8438528. 35432719 10.1155/2022/8438528 PMC9010207

[366] R. E. Speer , S. S. Karuppagounder , M. Basso , et al., “Hypoxia‐inducible factor prolyl hydroxylases as targets for neuroprotection by “antioxidant” metal chelators: From ferroptosis to stroke,” Free Radical Biology and Medicine 62 (2013): 26–36. 23376032 10.1016/j.freeradbiomed.2013.01.026 PMC4327984

[367] Y. Seki , P. J. Feustel , R. W. Keller Jr. , B. I. Tranmer , and H. K. Kimelberg , “Inhibition of ischemia‐induced glutamate release in rat striatum by dihydrokinate and an anion channel blocker,” Stroke; A Journal of Cerebral Circulation 30, no. 2 (1999): 433–440. 10.1161/01.str.30.2.433 9933284

[368] D. Jabaudon , M. Scanziani , B. H. Gähwiler , and U. Gerber , “Acute decrease in net glutamate uptake During energy deprivation,” PNAS 97, no. 10 (2000): 5610–5615. 10805815 10.1073/pnas.97.10.5610 PMC25876

[369] R. Brigelius‐Flohé and M. Maiorino , “Glutathione peroxidases,” Biochimica Et Biophysica Acta 1830, no. 5 (2013): 3289–3303. 23201771 10.1016/j.bbagen.2012.11.020

[370] W. Yang , X. Liu , C. Song , et al., “Structure‐activity relationship studies of phenothiazine derivatives as a new class of ferroptosis inhibitors together With the therapeutic effect in an ischemic stroke model,” European Journal of Medicinal Chemistry 209 (2021): 112842. 33065375 10.1016/j.ejmech.2020.112842

[371] Y. Zhang , X. Lu , B. Tai , W. Li , and T. Li , “Ferroptosis and Its Multifaceted Roles in Cerebral Stroke,” Frontiers in Cellular Neuroscience 15 (2021): 615372. 34149358 10.3389/fncel.2021.615372 PMC8209298

[372] K. Pravalika , D. Sarmah , H. Kaur , et al., “Trigonelline therapy confers neuroprotection by reduced glutathione mediated myeloperoxidase expression in animal model of ischemic stroke,” Life Sciences 216 (2019): 49–58. 30414429 10.1016/j.lfs.2018.11.014

[373] L. Sanguigno , N. Guida , S. Anzilotti , et al., “Stroke by inducing HDAC9‐dependent deacetylation of HIF‐1 and Sp1, promotes TfR1 transcription and GPX4 reduction, thus determining ferroptotic neuronal death,” International Journal of Biological Sciences 19, no. 9 (2023): 2695–2710. 37324938 10.7150/ijbs.80735 PMC10266075

[374] X. Hu , Y. Bao , M. Li , W. Zhang , and C. Chen , “The role of ferroptosis and its mechanism in ischemic stroke,” Experimental Neurology 372 (2024): 114630. 38056585 10.1016/j.expneurol.2023.114630

[375] N. DeGregorio‐Rocasolano , O. Martí‐Sistac , J. Ponce , et al., “Iron‐loaded transferrin (Tf) is detrimental whereas iron‐free Tf confers protection Against brain ischemia by modifying blood Tf saturation and subsequent neuronal damage,” Redox Biology 15 (2018): 143–158. 29248829 10.1016/j.redox.2017.11.026 PMC5975212

[376] B. Du , Z. Deng , K. Chen , et al., “Iron promotes both ferroptosis and necroptosis in the early stage of reperfusion in ischemic stroke,” Genes & Diseases 11, no. 6 (2024): 101262. 39286656 10.1016/j.gendis.2024.101262 PMC11402992

[377] P. Ballabh , A. Braun , and M. Nedergaard , “The blood‐brain barrier: An overview: Structure, regulation, and clinical implications,” Neurobiology of Disease 16, no. 1 (2004): 1–13. 15207256 10.1016/j.nbd.2003.12.016

[378] N. J. Abbott , L. Rönnbäck , and E. Hansson , “Astrocyte‐endothelial interactions at the blood‐brain barrier,” Nature Reviews Neuroscience 7, no. 1 (2006): 41–53. 16371949 10.1038/nrn1824

[379] H. Wolburg , S. Noell , A. Mack , K. Wolburg‐Buchholz , and P. Fallier‐Becker , “Brain endothelial cells and the glio‐vascular complex,” Cell and Tissue Research 335, no. 1 (2009): 75–96. 18633647 10.1007/s00441-008-0658-9

[380] K. Mathias , R. S. Machado , S. Stork , et al., “Blood‐brain barrier permeability in the ischemic stroke: An update,” Microvascular Research 151 (2024): 104621. 37918521 10.1016/j.mvr.2023.104621

[381] P. T. Do , C. C. Wu , Y. H. Chiang , C. J. Hu , and K. Y. Chen , “Mesenchymal Stem/Stromal Cell Therapy in Blood‐Brain Barrier Preservation Following Ischemia: Molecular Mechanisms and Prospects,” International Journal of Molecular Sciences 22, no. 18 (2021): 10045. 34576209 10.3390/ijms221810045 PMC8468469

[382] A. Mayevsky , H. Kutai‐Asis , and M. Tolmasov , “Mitochondrial function and brain Metabolic Score (BMS) in ischemic Stroke: Evaluation of “neuroprotectants” safety and efficacy,” Mitochondrion 50 (2020): 170–194. 31790815 10.1016/j.mito.2019.11.005

[383] A. Datta , D. Sarmah , H. Kaur , et al., “Post‐stroke Impairment of the Blood‐Brain Barrier and Perifocal Vasogenic Edema Is Alleviated by Endovascular Mesenchymal Stem Cell Administration: Modulation of the PKCδ/MMP9/AQP4‐Mediated Pathway,” Molecular Neurobiology 59, no. 5 (2022): 2758–2775. 35187613 10.1007/s12035-022-02761-2

[384] S. Manzanero , T. Santro , and T. V. Arumugam , “Neuronal oxidative stress in acute ischemic stroke: Sources and contribution to cell injury,” Neurochemistry International 62, no. 5 (2013): 712–718. 23201332 10.1016/j.neuint.2012.11.009

[385] P. Wang , Q. Ren , M. Shi , Y. Liu , H. Bai , and Y. Z. Chang , “Overexpression of Mitochondrial Ferritin Enhances Blood‐Brain Barrier Integrity Following Ischemic Stroke in Mice by Maintaining Iron Homeostasis in Endothelial Cells,” Antioxidants (Basel) 11, no. 7 (2022): 1257. 35883748 10.3390/antiox11071257 PMC9312053

[386] C. Yang , Y. Yang , K. M. DeMars , G. A. Rosenberg , and E. Candelario‐Jalil , “Genetic Deletion or Pharmacological Inhibition of Cyclooxygenase‐2 Reduces Blood‐Brain Barrier Damage in Experimental Ischemic Stroke,” Frontiers in Neurology 11 (2020): 887. 32973660 10.3389/fneur.2020.00887 PMC7468510

[387] J. H. Ryu , Y. Kim , M. J. Kim , et al., “Membrane‐Free Stem Cell Extract Enhances Blood‐Brain Barrier Integrity by Suppressing NF‐κB‐Mediated Activation of NLRP3 Inflammasome in Mice With Ischemic Stroke,” Life (Basel, Switzerland) 12, no. 4 (2022): 503. 35454994 10.3390/life12040503 PMC9032759

[388] J. J. Lochhead , G. McCaffrey , C. E. Quigley , et al., “Oxidative stress increases blood‐brain barrier permeability and induces alterations in occludin During hypoxia‐reoxygenation,” Journal of Cerebral Blood Flow and Metabolism: Official Journal of the International Society of Cerebral Blood Flow and Metabolism 30, no. 9 (2010): 1625–1636. 20234382 10.1038/jcbfm.2010.29 PMC2949263

[389] S. Sarvari , F. Moakedi , E. Hone , J. W. Simpkins , and X. Ren , “Mechanisms in blood‐brain barrier opening and metabolism‐challenged cerebrovascular ischemia With emphasis on ischemic stroke,” Metabolic Brain Disease 35, no. 6 (2020): 851–868. 32297170 10.1007/s11011-020-00573-8 PMC7988906

[390] Y. Li , B. Liu , T. Zhao , et al., “Comparative study of extracellular vesicles derived From mesenchymal stem cells and brain endothelial cells attenuating blood‐brain barrier permeability via regulating Caveolin‐1‐dependent ZO‐1 and Claudin‐5 endocytosis in acute ischemic stroke,” Journal of Nanobiotechnology 21, no. 1 (2023): 70. 36855156 10.1186/s12951-023-01828-z PMC9976550

[391] L. Xu , A. Nirwane , and Y. Yao , “Basement membrane and blood‐brain barrier,” Stroke and Vascular Neurology 4, no. 2 (2019): 78–82. 31338215 10.1136/svn-2018-000198 PMC6613871

[392] P. Sun , M. H. Hamblin , and K. J. Yin , “Non‐coding RNAs in the regulation of blood‐brain barrier functions in central nervous system disorders,” Fluids and Barriers of the CNS 19, no. 1 (2022): 27. 35346266 10.1186/s12987-022-00317-z PMC8959280

[393] S. R. Archie , A. Al Shoyaib , and L. Cucullo , “Blood‐Brain Barrier Dysfunction in CNS Disorders and Putative Therapeutic Targets: An Overview,” Pharmaceutics 13, no. 11 (2021): 1779. 34834200 10.3390/pharmaceutics13111779 PMC8622070

[394] A. Armulik , G. Genové , M. Mäe , et al., “Pericytes regulate the blood‐brain barrier,” Nature 468, no. 7323 (2010): 557–561. 20944627 10.1038/nature09522

[395] L. Li , A. Lundkvist , D. Andersson , et al., “Protective role of reactive astrocytes in brain ischemia,” Journal of Cerebral Blood Flow and Metabolism: Official Journal of the International Society of Cerebral Blood Flow and Metabolism 28, no. 3 (2008): 468–481. 17726492 10.1038/sj.jcbfm.9600546

[396] P. Carmeliet , “Mechanisms of angiogenesis and arteriogenesis,” Nature Medicine 6, no. 4 (2000): 389–395. 10.1038/74651 10742145

[397] T. N. Lin , S. W. Sun , W. M. Cheung , F. Li , and C. Chang , “Dynamic changes in cerebral blood flow and angiogenesis After transient focal cerebral ischemia in rats. Evaluation With serial magnetic resonance imaging,” Stroke; A Journal of Cerebral Circulation 33, no. 12 (2002): 2985–2991. 10.1161/01.str.0000037675.97888.9d 12468801

[398] E. Gunsilius , A. L. Petzer , G. Stockhammer , C. M. Kähler , and G. Gastl , “Serial measurement of vascular endothelial growth factor and transforming growth factor‐beta1 in serum of patients With acute ischemic stroke,” Stroke; A Journal of Cerebral Circulation 32, no. 1 (2001): 275–278. 10.1161/01.str.32.1.275-b 11136949

[399] Y. C. Chen , J. S. Wu , S. T. Yang , et al., “Stroke, angiogenesis and phytochemicals,” Frontiers in Bioscience (Scholar Edition) 4, no. 2 (2012): 599–610. 22202079 10.2741/s287

[400] G. Semenza , “Signal transduction to hypoxia‐inducible factor 1,” Biochemical Pharmacology 64, no. 5‐6 (2002): 993–998. 12213597 10.1016/s0006-2952(02)01168-1

[401] L. Chen , Y. M. Zhu , Y. N. Li , et al., “The 15‐LO‐1/15‐HETE system promotes angiogenesis by upregulating VEGF in ischemic brains,” Neurological Research 39, no. 9 (2017): 795–802. 28460604 10.1080/01616412.2017.1321710

[402] H. J. Marti , M. Bernaudin , A. Bellail , et al., “Hypoxia‐induced vascular endothelial growth factor expression precedes neovascularization After cerebral ischemia,” The American Journal of Pathology 156, no. 3 (2000): 965–976. 10702412 10.1016/S0002-9440(10)64964-4 PMC1876841

[403] L. Pérez‐Gutiérrez and N. Ferrara , “Biology and therapeutic targeting of vascular endothelial growth factor A,” Nature Reviews Molecular Cell Biology 24, no. 11 (2023): 816–834. 37491579 10.1038/s41580-023-00631-w

[404] C. S. Abhinand , R. Raju , S. J. Soumya , P. S. Arya , and P. R. Sudhakaran , “VEGF‐A/VEGFR2 signaling network in endothelial cells relevant to angiogenesis,” Journal of Cell Communication and Signaling 10, no. 4 (2016): 347–354. 27619687 10.1007/s12079-016-0352-8 PMC5143324

[405] Y. Yang and M. T. Torbey , “Angiogenesis and Blood‐Brain Barrier Permeability in Vascular Remodeling After Stroke,” Current Neuropharmacology 18, no. 12 (2020): 1250–1265. 32691713 10.2174/1570159X18666200720173316 PMC7770645

[406] G. J. del Zoppo and T. Mabuchi , “Cerebral microvessel responses to focal ischemia,” Journal of Cerebral Blood Flow and Metabolism: Official Journal of the International Society of Cerebral Blood Flow and Metabolism 23, no. 8 (2003): 879–894. 12902832 10.1097/01.WCB.0000078322.96027.78

[407] J. Liu , Y. Wang , Y. Akamatsu , et al., “Vascular remodeling After ischemic stroke: Mechanisms and therapeutic potentials,” Progress in Neurobiology 115 (2014): 138–156. 24291532 10.1016/j.pneurobio.2013.11.004 PMC4295834

[408] A. Zechariah , A. ElAli , T. R. Doeppner , et al., “Vascular endothelial growth factor promotes pericyte coverage of brain capillaries, improves cerebral blood flow During subsequent focal cerebral ischemia, and preserves the metabolic penumbra,” Stroke; A Journal of Cerebral Circulation 44, no. 6 (2013): 1690–1697. 10.1161/STROKEAHA.111.000240 23632977

[409] K. Nakamura , K. Arimura , A. Nishimura , et al., “Possible involvement of basic FGF in the upregulation of PDGFRβ in pericytes After ischemic stroke,” Brain Research 1630 (2016): 98–108. 26569132 10.1016/j.brainres.2015.11.003

[410] Y. Zou , J. Hu , W. Huang , et al., “Non‐Mitogenic Fibroblast Growth Factor 1 Enhanced Angiogenesis Following Ischemic Stroke by Regulating the Sphingosine‐1‐Phosphate 1 Pathway,” Frontiers in pharmacology 11 (2020): 59. 32194396 10.3389/fphar.2020.00059 PMC7063943

[411] Q. Pang , H. Zhang , Z. Chen , et al., “Role of caveolin‐1/vascular endothelial growth factor pathway in basic fibroblast growth factor‐induced angiogenesis and neurogenesis After treadmill training following focal cerebral ischemia in rats,” Brain Research 1663 (2017): 9–19. 28300551 10.1016/j.brainres.2017.03.012

[412] R. Z. Qian , F. Yue , G. P. Zhang , L. K. Hou , X. H. Wang , and H. M. Jin , “Roles of cyclooxygenase‐2 in microvascular endothelial cell proliferation induced by basic fibroblast growth factor,” Chinese Medical Journal 121, no. 24 (2008): 2599–2603. 19187602

[413] H. Zhao , Y. Zhang , Y. Zhang , et al., “NGF/FAK signal pathway is implicated in angiogenesis After acute cerebral ischemia in rats,” Neuroscience Letters 672 (2018): 96–102. 29458087 10.1016/j.neulet.2018.02.023

[414] X. Li , F. Li , L. Ling , C. Li , and Y. Zhong , “Intranasal administration of nerve growth factor promotes angiogenesis via activation of PI3K/Akt signaling following cerebral infarction in rats,” American journal of translational research 10, no. 11 (2018): 3481–3492. 30662601 PMC6291726

[415] Y. Zhu , C. Lee , F. Shen , R. Du , W. L. Young , and G. Y. Yang , “Angiopoietin‐2 facilitates vascular endothelial growth factor‐induced angiogenesis in the mature mouse brain,” Stroke; A Journal of Cerebral Circulation 36, no. 7 (2005): 1533–1537. 10.1161/01.STR.0000170712.46106.2e 15947259

[416] Z. Meng , M. Li , Q. He , et al., “Ectopic expression of human angiopoietin‐1 promotes functional recovery and neurogenesis After focal cerebral ischemia,” Neuroscience 267 (2014): 135–146. 24607344 10.1016/j.neuroscience.2014.02.036

[417] J. Krupinski , J. Kaluza , P. Kumar , S. Kumar , and J. M. Wang , “Role of angiogenesis in patients With cerebral ischemic stroke,” Stroke; A Journal of Cerebral Circulation 25, no. 9 (1994): 1794–1798. 10.1161/01.str.25.9.1794 7521076

[418] P. S. Manoonkitiwongsa , C. Jackson‐Friedman , P. J. McMillan , R. L. Schultz , and P. D. Lyden , “Angiogenesis After stroke is correlated With increased numbers of macrophages: The clean‐up hypothesis,” Journal of Cerebral Blood Flow and Metabolism: Official Journal of the International Society of Cerebral Blood Flow and Metabolism 21, no. 10 (2001): 1223–1231. 11598500 10.1097/00004647-200110000-00011

[419] L. Carey , A. Walsh , A. Adikari , et al., “Finding the Intersection of Neuroplasticity, Stroke Recovery, and Learning: Scope and Contributions to Stroke Rehabilitation,” Neural Plasticity 2019 (2019): 5232374. 31191637 10.1155/2019/5232374 PMC6525913

[420] S. Qin , Z. Zhang , Y. Zhao , et al., “The impact of acupuncture on neuroplasticity After ischemic stroke: A literature review and perspectives,” Frontiers in Cellular Neuroscience 16 (2022): 817732. 36439200 10.3389/fncel.2022.817732 PMC9685811

[421] L. M. Chavez , S. S. Huang , I. MacDonald , J. G. Lin , Y. C. Lee , and Y. H. Chen , “Mechanisms of Acupuncture Therapy in Ischemic Stroke Rehabilitation: A Literature Review of Basic Studies,” International Journal of Molecular Sciences 18, no. 11 (2017): 2270. 29143805 10.3390/ijms18112270 PMC5713240

[422] X. Wang , W. Xuan , and Z.‐Y. Zhu , “The evolving role of neuro‐immune interaction in brain repair After cerebral ischemic stroke,” CNS Neuroscience & Therapeutics 24, no. 12 (2018): 1100–1114. 30350341 10.1111/cns.13077 PMC6489764

[423] B. Wang and K. Jin , “Current perspectives on the link Between neuroinflammation and neurogenesis,” Metabolic Brain Disease 30, no. 2 (2015): 355–365. 24623361 10.1007/s11011-014-9523-6

[424] A. Arvidsson , T. Collin , D. Kirik , Z. Kokaia , and O. Lindvall , “Neuronal replacement From endogenous precursors in the adult brain After stroke,” Nature Medicine 8, no. 9 (2002): 963–970. 10.1038/nm747 12161747

[425] H. Tang , Y. Wang , L. Xie , et al., “Effect of neural precursor proliferation level on neurogenesis in rat brain During aging and After focal ischemia,” Neurobiology of Aging 30, no. 2 (2009): 299–308. 17644223 10.1016/j.neurobiolaging.2007.06.004 PMC2634816

[426] R. L. Zhang , Z. G. Zhang , M. Lu , Y. Wang , J. J. Yang , and M. Chopp , “Reduction of the cell cycle length by decreasing G1 phase and cell cycle reentry expand neuronal progenitor cells in the subventricular zone of adult rat After stroke,” Journal of Cerebral Blood Flow and Metabolism: Official Journal of the International Society of Cerebral Blood Flow and Metabolism 26, no. 6 (2006): 857–863. 16251885 10.1038/sj.jcbfm.9600237

[427] R. Zhang , Z. Zhang , C. Zhang , et al., “Stroke transiently increases subventricular zone cell division From asymmetric to symmetric and increases neuronal differentiation in the adult rat,” The Journal of Neuroscience: the Official Journal of the Society for Neuroscience 24, no. 25 (2004): 5810–5815. 15215303 10.1523/JNEUROSCI.1109-04.2004 PMC6729213

[428] S. Yoshimura , Y. Takagi , J. Harada , et al., “FGF‐2 regulation of neurogenesis in adult hippocampus After brain injury,” PNAS 98, no. 10 (2001): 5874–5879. 11320217 10.1073/pnas.101034998 PMC33306

[429] K. Jin , Y. Sun , L. Xie , et al., “Directed migration of neuronal precursors Into the ischemic cerebral cortex and striatum,” Molecular and Cellular Neurosciences 24, no. 1 (2003): 171–189. 14550778 10.1016/s1044-7431(03)00159-3

[430] P. Thored , A. Arvidsson , E. Cacci , et al., “Persistent production of neurons From adult brain stem cells During recovery After stroke,” Stem Cells 24, no. 3 (2006): 739–747. 16210404 10.1634/stemcells.2005-0281

[431] N. Kaneko , M. Sawada , and K. Sawamoto , “Mechanisms of neuronal migration in the adult brain,” Journal of Neurochemistry 141, no. 6 (2017): 835–847. 28251650 10.1111/jnc.14002

[432] E. Kokovay , S. Goderie , Y. Wang , et al., “Adult SVZ lineage cells home to and leave the vascular niche via differential responses to SDF1/CXCR4 signaling,” Cell Stem Cell 7, no. 2 (2010): 163–173. 20682445 10.1016/j.stem.2010.05.019 PMC2916873

[433] A. M. Robin , Z. G. Zhang , L. Wang , et al., “Stromal cell‐derived factor 1alpha mediates neural progenitor cell motility After focal cerebral ischemia,” Journal of Cerebral Blood Flow and Metabolism: Official Journal of the International Society of Cerebral Blood Flow and Metabolism 26, no. 1 (2006): 125–134. 15959456 10.1038/sj.jcbfm.9600172

[434] Y. P. Yan , K. A. Sailor , B. T. Lang , S. W. Park , R. Vemuganti , and R. J. Dempsey , “Monocyte chemoattractant protein‐1 plays a critical role in neuroblast migration After focal cerebral ischemia,” Journal of Cerebral Blood Flow and Metabolism: Official Journal of the International Society of Cerebral Blood Flow and Metabolism 27, no. 6 (2007): 1213–1224. 17191078 10.1038/sj.jcbfm.9600432

[435] M. Katakowski , Z. G. Zhang , J. Chen , et al., “Phosphoinositide 3‐kinase promotes adult subventricular neuroblast migration After stroke,” Journal of Neuroscience Research 74, no. 4 (2003): 494–501. 14598293 10.1002/jnr.10775

[436] S. S. Deshpande , S. C. Malik , P. Conforti , et al., “P75 neurotrophin receptor controls subventricular zone neural stem cell migration After stroke,” Cell and Tissue Research 387, no. 3 (2022): 415–431. 34698916 10.1007/s00441-021-03539-z PMC8975773

[437] H. Scharfman , J. Goodman , A. Macleod , S. Phani , C. Antonelli , and S. Croll , “Increased neurogenesis and the ectopic granule cells After intrahippocampal BDNF infusion in adult rats,” Experimental Neurology 192, no. 2 (2005): 348–356. 15755552 10.1016/j.expneurol.2004.11.016

[438] Z. Kokaia , G. Andsberg , Q. Yan , and O. Lindvall , “Rapid alterations of BDNF protein levels in the rat brain After focal ischemia: Evidence for increased synthesis and anterograde axonal transport,” Experimental Neurology 154, no. 2 (1998): 289–301. 9878168 10.1006/exnr.1998.6888

[439] G. Andsberg , Z. Kokaia , R. L. Klein , N. Muzyczka , O. Lindvall , and R. J. Mandel , “Neuropathological and behavioral consequences of adeno‐associated viral vector‐mediated continuous intrastriatal neurotrophin delivery in a focal ischemia model in rats,” Neurobiology of Disease 9, no. 2 (2002): 187–204. 11895371 10.1006/nbdi.2001.0456

[440] P. Carmeliet and R. K. Jain , “Molecular mechanisms and clinical applications of angiogenesis,” Nature 473, no. 7347 (2011): 298–307. 21593862 10.1038/nature10144 PMC4049445

[441] L. H. Shen , Y. Li , Q. Gao , S. Savant‐Bhonsale , and M. Chopp , “Down‐regulation of neurocan expression in reactive astrocytes promotes axonal regeneration and facilitates the neurorestorative effects of bone marrow stromal cells in the ischemic rat brain,” Glia 56, no. 16 (2008): 1747–1754. 18618668 10.1002/glia.20722 PMC2575136

[442] R. J. Nudo , “Postinfarct cortical plasticity and behavioral recovery,” Stroke; A Journal of Cerebral Circulation 38, no. 2 Suppl (2007): 840–845. 10.1161/01.STR.0000247943.12887.d2 17261749

[443] L. Ruan , B. Wang , Q. ZhuGe , and K. Jin , “Coupling of neurogenesis and angiogenesis After ischemic stroke,” Brain Research 1623 (2015): 166–173. 25736182 10.1016/j.brainres.2015.02.042 PMC4552615

[444] A. G. Nikonenko , L. Radenovic , P. R. Andjus , and G. G. Skibo , “Structural features of ischemic damage in the hippocampus,” Anatomical Record (Hoboken, NJ: 2007) 292, no. 12 (2009): 1914–1921. 10.1002/ar.20969 19943345

[445] W. J. Costain , I. Rasquinha , J. K. Sandhu , et al., “Cerebral ischemia causes dysregulation of synaptic adhesion in mouse synaptosomes,” Journal of Cerebral Blood Flow and Metabolism: Official Journal of the International Society of Cerebral Blood Flow and Metabolism 28, no. 1 (2008): 99–110. 17519975 10.1038/sj.jcbfm.9600510

[446] K. Hama , “Studies on fine structure and function of synapses,” Progress in Brain Research 21 (1966): 251–267. 5939646 10.1016/s0079-6123(08)62980-5

[447] K. E. Sorra and K. M. Harris , “Overview on the structure, composition, function, development, and plasticity of hippocampal dendritic spines,” Hippocampus 10, no. 5 (2000): 501–511. 11075821 10.1002/1098-1063(2000)10:5<501::AID-HIPO1>3.0.CO;2-T

[448] J. A. Szule , J. H. Jung , and U. J. McMahan , “The structure and function of ‘active zone material’ at synapses,” Philosophical Transactions of the Royal Society of London Series B, Biological Sciences 370, no. 1672 (2015): 20140189. 26009768 10.1098/rstb.2014.0189 PMC4455758

[449] J. Chen , A. Zacharek , X. Cui , et al., “Treatment of stroke With a synthetic liver X receptor agonist, TO901317, promotes synaptic plasticity and axonal regeneration in mice,” Journal of Cerebral Blood Flow and Metabolism: Official Journal of the International Society of Cerebral Blood Flow and Metabolism 30, no. 1 (2010): 102–109. 19724285 10.1038/jcbfm.2009.187 PMC2804900

[450] M. Haber , L. Zhou , and K. K. Murai , “Cooperative astrocyte and dendritic spine dynamics at hippocampal excitatory synapses,” The Journal of Neuroscience: the Official Journal of the Society for Neuroscience 26, no. 35 (2006): 8881–8891. 16943543 10.1523/JNEUROSCI.1302-06.2006 PMC6675342

[451] X. Wang , W. Xuan , and Z. Y. Zhu , “The evolving role of neuro‐immune interaction in brain repair After cerebral ischemic stroke,” CNS neuroscience & therapeutics 24, no. 12 (2018): 1100–1114. 30350341 10.1111/cns.13077 PMC6489764

[452] C. Xing , X. Wang , C. Cheng , et al., “Neuronal production of lipocalin‐2 as a help‐me signal for glial activation,” Stroke; A Journal of Cerebral Circulation 45, no. 7 (2014): 2085–2092. 10.1161/STROKEAHA.114.005733 PMC4122238 24916903

[453] C. E. Tournell , R. A. Bergstrom , and A. Ferreira , “Progesterone‐induced agrin expression in astrocytes modulates glia‐neuron interactions leading to synapse formation,” Neuroscience 141, no. 3 (2006): 1327–1338. 16777347 10.1016/j.neuroscience.2006.05.004

[454] W. C. Risher and C. Eroglu , “Thrombospondins as key regulators of synaptogenesis in the central nervous system,” Matrix Biology: Journal of the International Society for Matrix Biology 31, no. 3 (2012): 170–177. 22285841 10.1016/j.matbio.2012.01.004 PMC3961754

[455] A. Diaz , P. Merino , L. G. Manrique , L. Cheng , and M. Yepes , “Urokinase‐type plasminogen activator (uPA) protects the tripartite synapse in the ischemic brain via ezrin‐mediated formation of peripheral astrocytic processes,” Journal of Cerebral Blood Flow and Metabolism: Official Journal of the International Society of Cerebral Blood Flow and Metabolism 39, no. 11 (2019): 2157–2171. 29890880 10.1177/0271678X18783653 PMC6827113

[456] Y. Bernardinelli , J. Randall , and E. Janett , “Activity‐dependent structural plasticity of perisynaptic astrocytic domains promotes excitatory synapse stability,” Current Biology: CB 24, no. 15 (2014): 1679–1688. 25042585 10.1016/j.cub.2014.06.025

[457] A. R. Rǎdulescu , G. C. Todd , C. L. Williams , et al., “Estimating the glutamate transporter surface density in distinct sub‐cellular compartments of mouse hippocampal astrocytes,” PLoS Computational Biology 18, no. 2 (2022): e1009845. 35120128 10.1371/journal.pcbi.1009845 PMC8849624

[458] A. R. Peterson and D. K. Binder , “Astrocyte Glutamate Uptake and Signaling as Novel Targets for Antiepileptogenic Therapy,” Frontiers in Neurology 11 (2020): 1006. 33013665 10.3389/fneur.2020.01006 PMC7505989

[459] M. Letellier and Y. Goda , “Astrocyte Calcium Signaling Shifts the Polarity of Presynaptic Plasticity,” Neuroscience 525 (2023): 38–46. 37295597 10.1016/j.neuroscience.2023.05.032

[460] Y. Xiong , S. Teng , L. Zheng , et al., “Stretch‐induced Ca(2+) independent ATP release in hippocampal astrocytes,” The Journal of Physiology 596, no. 10 (2018): 1931–1947. 29488635 10.1113/JP275805 PMC5978314

[461] C. Durkee , P. Kofuji , M. Navarrete , and A. Araque , “Astrocyte and neuron cooperation in long‐term depression,” Trends in Neurosciences 44, no. 10 (2021): 837–848. 34334233 10.1016/j.tins.2021.07.004 PMC8484065

[462] G. Perea , R. Gómez , S. Mederos , et al., “Activity‐dependent switch of GABAergic inhibition Into glutamatergic excitation in astrocyte‐neuron networks,” Elife 5 (2016): e20362. 28012274 10.7554/eLife.20362 PMC5231406

[463] C. L. Duan , C. W. Liu , S. W. Shen , et al., “Striatal astrocytes transdifferentiate Into functional mature neurons following ischemic brain injury,” Glia 63, no. 9 (2015): 1660–1670. 26031629 10.1002/glia.22837 PMC5033006

[464] G. G. Skibo and A. G. Nikonenko , “Brain plasticity After ischemic episode,” Vitamins and Hormones 82 (2010): 107–127. 20472135 10.1016/S0083-6729(10)82006-0

[465] D. J. Cook , C. Nguyen , H. N. Chun , et al., “Hydrogel‐delivered brain‐derived neurotrophic factor promotes tissue repair and recovery After stroke,” Journal of Cerebral Blood Flow and Metabolism: Official Journal of the International Society of Cerebral Blood Flow and Metabolism 37, no. 3 (2017): 1030–1045. 27174996 10.1177/0271678X16649964 PMC5363479

[466] S. T. Carmichael , L. Wei , C. M. Rovainen , and T. A. Woolsey , “New patterns of intracortical projections After focal cortical stroke,” Neurobiology of Disease 8, no. 5 (2001): 910–922. 11592858 10.1006/nbdi.2001.0425

[467] C. E. Brown , K. Aminoltejari , H. Erb , I. R. Winship , and T. H. Murphy , “In vivo voltage‐sensitive dye imaging in adult mice reveals that somatosensory maps lost to stroke are replaced Over weeks by new structural and functional circuits With prolonged modes of activation Within both the peri‐infarct zone and distant sites,” The Journal of Neuroscience: the Official Journal of the Society for Neuroscience 29, no. 6 (2009): 1719–1734. 19211879 10.1523/JNEUROSCI.4249-08.2009 PMC6666293

[468] S. T. Carmichael , “Gene expression changes After focal stroke, traumatic brain and spinal cord injuries,” Current Opinion in Neurology 16, no. 6 (2003): 699–704. 14624079 10.1097/01.wco.0000102621.38669.77

[469] D. Chen , X. Wei , J. Zou , et al., “Contra‐Directional Expression of Serum Homocysteine and Uric Acid as Important Biomarkers of Multiple System Atrophy Severity: A Cross‐Sectional Study,” Frontiers in Cellular Neuroscience 9 (2015): 247. 26217177 10.3389/fncel.2015.00247 PMC4492156

[470] S. J. Li , K. F. Cui , J. J. Fu , et al., “EPO promotes axonal sprouting via upregulating GDF10,” Neuroscience Letters 711 (2019): 134412. 31381959 10.1016/j.neulet.2019.134412

[471] A. J. Gleichman and S. T. Carmichael , “Astrocytic therapies for neuronal repair in stroke,” Neuroscience Letters 565 (2014): 47–52. 24184876 10.1016/j.neulet.2013.10.055

[472] D. H. Choi , J. H. Ahn , I. A. Choi , J. H. Kim , B. R. Kim , and J. Lee , “Effect of task‐specific training on Eph/ephrin expression After stroke,” BMB Reports 49, no. 11 (2016): 635–640. 27756445 10.5483/BMBRep.2016.49.11.172 PMC5346325

[473] M. Rickhag , M. Teilum , and T. Wieloch , “Rapid and long‐term induction of effector immediate early genes (BDNF, Neuritin and Arc) in peri‐infarct cortex and dentate gyrus After ischemic injury in rat brain,” Brain Research 1151 (2007): 203–210. 17397810 10.1016/j.brainres.2007.03.005

[474] A. E. Autry and L. M. Monteggia , “Brain‐derived neurotrophic factor and neuropsychiatric disorders,” Pharmacological Reviews 64, no. 2 (2012): 238–258. 22407616 10.1124/pr.111.005108 PMC3310485

[475] M. G. Lykissas , A. K. Batistatou , K. A. Charalabopoulos , and A. E. Beris , “The role of neurotrophins in axonal growth, guidance, and regeneration,” Current Neurovascular Research 4, no. 2 (2007): 143–151. 17504212 10.2174/156720207780637216

[476] J. M. Cassidy and S. C. Cramer , “Spontaneous and Therapeutic‐Induced Mechanisms of Functional Recovery After Stroke,” Translational Stroke Research 8, no. 1 (2017): 33–46. 27109642 10.1007/s12975-016-0467-5 PMC5079852

[477] X. Hu , R. K. Leak , Y. Shi , et al., “Microglial and macrophage polarization—new prospects for brain repair,” Nature reviews Neurology 11, no. 1 (2015): 56–64. 25385337 10.1038/nrneurol.2014.207 PMC4395497

[478] J. Wang , H. Zhao , Z. Fan , et al., “Long Noncoding RNA H19 Promotes Neuroinflammation in Ischemic Stroke by Driving Histone Deacetylase 1‐Dependent M1 Microglial Polarization,” Stroke; A Journal of Cerebral Circulation 48, no. 8 (2017): 2211–2221. 10.1161/STROKEAHA.117.017387 28630232

[479] S. B. R. Harley , E. F. Willis , S. N. Shaikh , et al., “Selective Ablation of BDNF From Microglia Reveals Novel Roles in Self‐Renewal and Hippocampal Neurogenesis,” The Journal of Neuroscience: the Official Journal of the Society for Neuroscience 41, no. 19 (2021): 4172–4186. 33785644 10.1523/JNEUROSCI.2539-20.2021 PMC8143199

[480] H. O. Kalkman and D. Feuerbach , “Microglia M2A Polarization as Potential Link Between Food Allergy and Autism Spectrum Disorders,” Pharmaceuticals (Basel, Switzerland) 10, no. 4 (2017): 95. 29232822 10.3390/ph10040095 PMC5748650

[481] J. Yuan , H. Ge , W. Liu , et al., “M2 microglia promotes neurogenesis and oligodendrogenesis From neural stem/progenitor cells via the PPARγ signaling pathway,” Oncotarget 8, no. 12 (2017): 19855–19865. 28423639 10.18632/oncotarget.15774 PMC5386728

[482] L. Li , Y. Wang , H. Wang , L. Lv , and Z. Y. Zhu , “Metabolic responses of BV‐2 cells to puerarin on its polarization using ultra‐performance liquid chromatography‐mass spectrometry,” Biomedical Chromatography: BMC 34, no. 4 (2020): e4796. 31960437 10.1002/bmc.4796

[483] J. Li , X. Mi , Z. Yang , et al., “Minocycline ameliorates cognitive impairment in rats With trigeminal neuralgia by regulating microglial polarization,” International Immunopharmacology 145 (2025): 113786. 39672028 10.1016/j.intimp.2024.113786

[484] J. Zeng , T. Bao , K. Yang , et al., “The mechanism of microglia‐mediated immune inflammation in ischemic stroke and the role of natural botanical components in regulating microglia: A review,” Frontiers in immunology 13 (2022): 1047550. 36818470 10.3389/fimmu.2022.1047550 PMC9933144

[485] K. Gertz , G. Kronenberg , R. E. Kälin , et al., “Essential role of interleukin‐6 in post‐stroke angiogenesis,” Brain: a Journal of Neurology 135, no. Pt 6 (2012): 1964–1980. 22492561 10.1093/brain/aws075 PMC3359750

[486] J. Galea and D. Brough , “The role of inflammation and interleukin‐1 in acute cerebrovascular disease,” Journal of Inflammation Research 6 (2013): 121–128. 24062616 10.2147/JIR.S35629 PMC3780292

[487] D. A. Greenberg and K. Jin , “Vascular endothelial growth factors (VEGFs) and stroke,” Cellular and Molecular Life Sciences: CMLS 70, no. 10 (2013): 1753–1761. 23475070 10.1007/s00018-013-1282-8 PMC3634892

[488] D. P. Schafer , E. K. Lehrman , A. G. Kautzman , et al., “Microglia sculpt postnatal neural circuits in an activity and complement‐dependent manner,” Neuron 74, no. 4 (2012): 691–705. 22632727 10.1016/j.neuron.2012.03.026 PMC3528177

[489] Y. Yang , V. M. Salayandia , J. F. Thompson , L. Y. Yang , E. Y. Estrada , and Y. Yang , “Attenuation of acute stroke injury in rat brain by minocycline promotes blood‐brain barrier remodeling and alternative microglia/macrophage activation During recovery,” Journal of Neuroinflammation 12 (2015): 26. 25889169 10.1186/s12974-015-0245-4 PMC4340283

[490] Y. Hou , D. Yang , R. Xiang , et al., “N2 neutrophils may participate in spontaneous recovery After transient cerebral ischemia by inhibiting ischemic neuron injury in rats,” International Immunopharmacology 77 (2019): 105970. 31675618 10.1016/j.intimp.2019.105970

[491] W. Cai , S. Liu , M. Hu , et al., “Functional Dynamics of Neutrophils After Ischemic Stroke,” Translational Stroke Research 11, no. 1 (2020): 108–121. 30847778 10.1007/s12975-019-00694-y PMC6993940

[492] K. N. Corps , T. L. Roth , and D. B. McGavern , “Inflammation and neuroprotection in traumatic brain injury,” JAMA neurology 72, no. 3 (2015): 355–362. 25599342 10.1001/jamaneurol.2014.3558 PMC5001842

[493] B. McDonald , K. Pittman , G. B. Menezes , et al., “Intravascular danger signals guide neutrophils to sites of sterile inflammation,” Science (New York, NY) 330, no. 6002 (2010): 362–366. 10.1126/science.1195491 20947763

[494] B. V. Zlokovic , “Remodeling After stroke,” Nature Medicine 12, no. 4 (2006): 390–391. 10.1038/nm0406-390 16598283

[495] S. Wattananit , D. Tornero , N. Graubardt , et al., “Monocyte‐Derived Macrophages Contribute to Spontaneous Long‐Term Functional Recovery After Stroke in Mice,” The Journal of Neuroscience: the Official Journal of the Society for Neuroscience 36, no. 15 (2016): 4182–4195. 27076418 10.1523/JNEUROSCI.4317-15.2016 PMC6601783

[496] N. Wang , H. Liang , and K. Zen , “Molecular mechanisms that influence the macrophage m1‐m2 polarization balance,” Frontiers in immunology 5 (2014): 614. 25506346 10.3389/fimmu.2014.00614 PMC4246889

[497] N. Kolosowska , M. H. Keuters , S. Wojciechowski , et al., “Peripheral Administration of IL‐13 Induces Anti‐inflammatory Microglial/Macrophage Responses and Provides Neuroprotection in Ischemic Stroke,” Neurotherapeutics: the Journal of the American Society for Experimental NeuroTherapeutics 16, no. 4 (2019): 1304–1319. 31372938 10.1007/s13311-019-00761-0 PMC6985054

[498] C. F. Nathan , “Secretory products of macrophages,” The Journal of Clinical Investigation 79, no. 2 (1987): 319–326. 3543052 10.1172/JCI112815 PMC424063

[499] M. J. Kwon , J. Kim , H. Shin , et al., “Contribution of macrophages to enhanced regenerative capacity of dorsal root ganglia sensory neurons by conditioning injury,” The Journal of Neuroscience: the Official Journal of the Society for Neuroscience 33, no. 38 (2013): 15095–15108. 24048840 10.1523/JNEUROSCI.0278-13.2013 PMC6618417

[500] R. Shechter , A. London , C. Varol , et al., “Infiltrating blood‐derived macrophages are vital cells playing an anti‐inflammatory role in recovery From spinal cord injury in mice,” PLoS Medicine 6, no. 7 (2009): e1000113. 19636355 10.1371/journal.pmed.1000113 PMC2707628

[501] X. Y. Xiong , L. Liu , and Q. W. Yang , “Functions and mechanisms of microglia/macrophages in neuroinflammation and neurogenesis After stroke,” Progress in Neurobiology 142 (2016): 23–44. 27166859 10.1016/j.pneurobio.2016.05.001

[502] L. Garcia‐Bonilla , G. Faraco , J. Moore , et al., “Spatio‐temporal profile, phenotypic diversity, and fate of recruited monocytes Into the post‐ischemic brain,” Journal of Neuroinflammation 13, no. 1 (2016): 285. 27814740 10.1186/s12974-016-0750-0 PMC5097435

[503] C. Liu , C. Wu , Q. Yang , et al., “Macrophages Mediate the Repair of Brain Vascular Rupture Through Direct Physical Adhesion and Mechanical Traction,” Immunity 44, no. 5 (2016): 1162–1176. 27156384 10.1016/j.immuni.2016.03.008

[504] P. Carmona‐Mora , B. P. Ander , G. C. Jickling , et al., “Distinct peripheral blood monocyte and neutrophil transcriptional programs following intracerebral hemorrhage and different etiologies of ischemic stroke,” Journal of Cerebral Blood Flow and Metabolism: Official Journal of the International Society of Cerebral Blood Flow and Metabolism 41, no. 6 (2021): 1398–1416. 32960689 10.1177/0271678X20953912 PMC8142129

[505] R. Wang , Y. Liu , Q. Ye , et al., “RNA sequencing reveals novel macrophage transcriptome favoring neurovascular plasticity After ischemic stroke,” Journal of Cerebral Blood Flow and Metabolism: Official Journal of the International Society of Cerebral Blood Flow and Metabolism 40, no. 4 (2020): 720–738. 31722596 10.1177/0271678X19888630 PMC7168800

[506] S. Bauer , B. J. Kerr , and P. H. Patterson , “The neuropoietic cytokine family in development, plasticity, disease and injury,” Nature Reviews Neuroscience 8, no. 3 (2007): 221–232. 17311007 10.1038/nrn2054

[507] A. J. Filiano , Y. Xu , N. J. Tustison , et al., “Unexpected role of interferon‐γ in regulating neuronal connectivity and social behaviour,” Nature 535, no. 7612 (2016): 425–429. 27409813 10.1038/nature18626 PMC4961620

[508] R. Barouch and M. Schwartz , “Autoreactive T cells induce neurotrophin production by immune and neural cells in injured rat optic nerve: Implications for protective autoimmunity,” FASEB Journal: Official Publication of the Federation of American Societies for Experimental Biology 16, no. 10 (2002): 1304–1306. 12154003 10.1096/fj.01-0467fje

[509] A. L. Rodríguez‐Perea , J. Gutierrez‐Vargas , G. P. Cardona‐Gómez , C. J. Guarin , M. Rojas , and P. A. Hernández , “Atorvastatin Modulates Regulatory T Cells and Attenuates Cerebral Damage in a Model of Transient Middle Cerebral Artery Occlusion in Rats,” Journal of Neuroimmune Pharmacology: the Official Journal of the Society on NeuroImmune Pharmacology 12, no. 1 (2017): 152–162. 27614888 10.1007/s11481-016-9706-5

[510] A. Liesz , X. Hu , C. Kleinschnitz , and H. Offner , “Functional role of regulatory lymphocytes in stroke: Facts and controversies,” Stroke; A Journal of Cerebral Circulation 46, no. 5 (2015): 1422–1430. 10.1161/STROKEAHA.114.008608 PMC4414876 25791715

[511] S. Y. Na , E. Mracsko , A. Liesz , T. Hünig , and R. Veltkamp , “Amplification of regulatory T cells using a CD28 superagonist reduces brain damage After ischemic stroke in mice,” Stroke; A Journal of Cerebral Circulation 46, no. 1 (2015): 212–220. 10.1161/STROKEAHA.114.007756 25378432

[512] J. T. Walsh , S. Hendrix , F. Boato , et al., “MHCII‐independent CD4+ T cells protect injured CNS neurons via IL‐4,” The Journal of Clinical Investigation 125, no. 2 (2015): 699–714. 25607842 10.1172/JCI76210 PMC4319416

[513] P. Li , L. Mao , X. Liu , et al., “Essential role of program death 1‐ligand 1 in regulatory T‐cell‐afforded protection Against blood‐brain barrier damage After stroke,” Stroke; A Journal of Cerebral Circulation 45, no. 3 (2014): 857–864. 10.1161/STROKEAHA.113.004100 PMC3939692 24496394

[514] P. Li , Y. Gan , B. L. Sun , et al., “Adoptive regulatory T‐cell therapy protects Against cerebral ischemia,” Annals of Neurology 74, no. 3 (2013): 458–471. 23674483 10.1002/ana.23815 PMC3748165

[515] L. Mao , P. Li , W. Zhu , et al., “Regulatory T cells ameliorate tissue plasminogen activator‐induced brain haemorrhage After stroke,” Brain: a Journal of Neurology 140, no. 7 (2017): 1914–1931. 28535201 10.1093/brain/awx111 PMC6059175

[516] K. P. Doyle , L. N. Quach , M. Solé , et al., “B‐lymphocyte‐mediated delayed cognitive impairment following stroke,” The Journal of Neuroscience: the Official Journal of the Society for Neuroscience 35, no. 5 (2015): 2133–2145. 25653369 10.1523/JNEUROSCI.4098-14.2015 PMC4315838

[517] F. Flores‐Borja , A. Bosma , D. Ng , et al., “CD19+CD24hiCD38hi B cells maintain regulatory T cells while limiting TH1 and TH17 differentiation,” Science Translational Medicine 5, no. 173 (2013): 173ra23. 10.1126/scitranslmed.3005407 23427243

[518] X. Ren , K. Akiyoshi , S. Dziennis , et al., “Regulatory B cells limit CNS inflammation and neurologic deficits in murine experimental stroke,” The Journal of Neuroscience: the Official Journal of the Society for Neuroscience 31, no. 23 (2011): 8556–8563. 21653859 10.1523/JNEUROSCI.1623-11.2011 PMC3111929

[519] A. Mizoguchi , E. Mizoguchi , R. N. Smith , F. I. Preffer , and A. K. Bhan , “Suppressive role of B cells in chronic colitis of T cell receptor alpha mutant mice,” The Journal of Experimental Medicine 186, no. 10 (1997): 1749–1756. 9362534 10.1084/jem.186.10.1749 PMC2199135

[520] J. A. Vega , O. García‐Suárez , J. Hannestad , M. Pérez‐Pérez , and A. Germanà , “Neurotrophins and the immune system,” Journal of Anatomy 203, no. 1 (2003): 1–19. 12892403 10.1046/j.1469-7580.2003.00203.x PMC1571144

[521] M. K. Malone , T. A. Ujas , D. R. S. Britsch , K. M. Cotter , K. Poinsatte , and A. M. Stowe , “The immunopathology of B lymphocytes During stroke‐induced injury and repair,” Seminars in Immunopathology 45, no. 3 (2023): 315–327. 36446955 10.1007/s00281-022-00971-3 PMC9708141

[522] D. Wu , J. Zhou , Y. Zheng , et al., “Pathogenesis‐adaptive polydopamine nanosystem for sequential therapy of ischemic stroke,” Nature Communications 14, no. 1 (2023): 7147. 10.1038/s41467-023-43070-z PMC10628287 37932306

[523] A. D. Mendelow , B. A. Gregson , E. N. Rowan , G. D. Murray , A. Gholkar , and P. M. Mitchell , “Early surgery versus initial conservative treatment in patients With spontaneous supratentorial lobar intracerebral haematomas (STICH II): A randomised trial,” Lancet 382, no. 9890 (2013): 397–408. 23726393 10.1016/S0140-6736(13)60986-1 PMC3906609

[524] B. Lei , H. N. Dawson , B. Roulhac‐Wilson , H. Wang , D. T. Laskowitz , and M. L. James , “Tumor necrosis factor α antagonism improves neurological recovery in murine intracerebral hemorrhage,” J Neuroinflammation 10 (2013): 103. 23962089 10.1186/1742-2094-10-103 PMC3765285

[525] D. L. Alsbrook , M. Di Napoli , K. Bhatia , et al., “Neuroinflammation in Acute Ischemic and Hemorrhagic Stroke,” Current Neurology and Neuroscience Reports 23, no. 8 (2023): 407–431. 37395873 10.1007/s11910-023-01282-2 PMC10544736

[526] X.‐Y. Xiong , L. Liu , and Q.‐W. Yang , “Functions and mechanisms of microglia/macrophages in neuroinflammation and neurogenesis After stroke,” Progress in Neurobiology 142 (2016): 23–44. 27166859 10.1016/j.pneurobio.2016.05.001

[527] K. Saijo and C. K. Glass , “Microglial cell origin and phenotypes in health and disease,” Nature Reviews Immunology 11, no. 11 (2011): 775–787. 10.1038/nri3086 22025055

[528] R. Franco and D. Fernández‐Suárez , “Alternatively activated microglia and macrophages in the central nervous system,” Progress in Neurobiology 131 (2015): 65–86. 26067058 10.1016/j.pneurobio.2015.05.003

[529] J. A. Rodríguez‐Gómez , E. Kavanagh , P. Engskog‐Vlachos , et al., “Microglia: Agents of the CNS Pro‐Inflammatory Response,” Cells 9, no. 7 (2020): 1717. 32709045 10.3390/cells9071717 PMC7407646

[530] Y.‐C. Wang , P.‐F. Wang , H. Fang , J. Chen , X.‐Y. Xiong , and Q.‐W. Yang , “Toll‐Like receptor 4 antagonist attenuates intracerebral hemorrhage‐induced brain injury,” Stroke; A Journal of Cerebral Circulation 44, no. 9 (2013): 2545–2552. 10.1161/STROKEAHA.113.001038 23839500

[531] W. Ling , Y. Cui , J. Gao , et al., “Antcin C ameliorates neuronal inflammation due to cerebral haemorrhage by inhibiting the TLR‐4 pathway,” Folia Neuropathologica 58, no. 4 (2020): 317–323. 33480236 10.5114/fn.2020.102434

[532] J. Holbrook , S. Lara‐Reyna , H. Jarosz‐Griffiths , and M. McDermott , “Tumour necrosis factor signalling in health and disease,” F1000Research 8 (2019). F1000 Faculty Rev‐111. 10.12688/f1000research.17023.1 PMC6352924 30755793

[533] X. Xu , H.‐N. Piao , F. Aosai , et al., “Arctigenin protects Against depression by inhibiting microglial activation and neuroinflammation via HMGB1/TLR4/NF‐κB and TNF‐α/TNFR1/NF‐κB pathways,” British Journal of Pharmacology 177, no. 22 (2020): 5224–5245. 32964428 10.1111/bph.15261 PMC7589024

[534] M. A. Cinelli , H. T. Do , G. P. Miley , and R. B. Silverman , “Inducible nitric oxide synthase: Regulation, structure, and inhibition,” Medicinal Research Reviews 40, no. 1 (2020): 158–189. 31192483 10.1002/med.21599 PMC6908786

[535] K. Kashfi , J. Kannikal , and N. Nath , “Macrophage Reprogramming and Cancer Therapeutics: Role of iNOS‐Derived NO,” Cells 10, no. 11 (2021): 3194. 34831416 10.3390/cells10113194 PMC8624911

[536] D. Z. Liu , B. P. Ander , H. Xu , et al., “Blood‐brain barrier breakdown and repair by Src After thrombin‐induced injury,” Annals of Neurology 67, no. 4 (2010): 526–533. 20437588 10.1002/ana.21924 PMC2919346

[537] A. A. Atta , W. W. Ibrahim , A. F. Mohamed , and N. F. Abdelkader , “Microglia polarization in nociplastic pain: Mechanisms and perspectives,” Inflammopharmacology 31, no. 3 (2023): 1053–1067. 37069462 10.1007/s10787-023-01216-x PMC10229465

[538] H. Y. Nam , J. H. Nam , G. Yoon , et al., “Ibrutinib suppresses LPS‐induced neuroinflammatory responses in BV2 microglial cells and wild‐type mice,” Journal of Neuroinflammation 15, no. 1 (2018): 271. 30231870 10.1186/s12974-018-1308-0 PMC6145206

[539] M. Alhouayek and G. G. Muccioli , “COX‐2‐derived endocannabinoid metabolites as novel inflammatory mediators,” Trends in Pharmacological Sciences 35, no. 6 (2014): 284–292. 24684963 10.1016/j.tips.2014.03.001

[540] M. Wang , X. Ye , J. Hu , et al., “NOD1/RIP2 signalling enhances the microglia‐driven inflammatory response and undergoes crosstalk With inflammatory cytokines to exacerbate brain damage following intracerebral haemorrhage in mice,” Journal of Neuroinflammation 17, no. 1 (2020): 364. 33261639 10.1186/s12974-020-02015-9 PMC7708246

[541] Q. Lu , R. Liu , P. Sherchan , et al., “TREM (Triggering Receptor Expressed on Myeloid Cells)‐1 Inhibition Attenuates Neuroinflammation via PKC (Protein Kinase C) δ/CARD9 (Caspase Recruitment Domain Family Member 9) Signaling Pathway After Intracerebral Hemorrhage in Mice,” Stroke; A Journal of Cerebral Circulation 52, no. 6 (2021): 2162–2173. 10.1161/STROKEAHA.120.032736 PMC8321132 33947214

[542] Y. Zhang , S. Khan , Y. Liu , G. Wu , V. W. Yong , and M. Xue , “Oxidative Stress Following Intracerebral Hemorrhage: From Molecular Mechanisms to Therapeutic Targets,” Frontiers in immunology 13 (2022): 847246. 35355999 10.3389/fimmu.2022.847246 PMC8959663

[543] C. Duan , H. Wang , D. Jiao , et al., “Curcumin Restrains Oxidative Stress of After Intracerebral Hemorrhage in Rat by Activating the Nrf2/HO‐1 Pathway,” Frontiers in pharmacology 13 (2022): 889226. 35571134 10.3389/fphar.2022.889226 PMC9092178

[544] S. M. Sadrzadeh and J. W. Eaton , “Hemoglobin‐mediated oxidant damage to the central nervous system requires endogenous ascorbate,” The Journal of Clinical Investigation 82, no. 5 (1988): 1510–1515. 2846656 10.1172/JCI113759 PMC442716

[545] J. Wu , Y. Hua , R. F. Keep , T. Schallert , J. T. Hoff , and G. Xi , “Oxidative brain injury From extravasated erythrocytes After intracerebral hemorrhage,” Brain Research 953, no. 1‐2 (2002): 45–52. 12384237 10.1016/s0006-8993(02)03268-7

[546] Q. Ma , S. Chen , Q. Hu , H. Feng , J. H. Zhang , and J. Tang , “NLRP3 inflammasome contributes to inflammation After intracerebral hemorrhage,” Annals of Neurology 75, no. 2 (2014): 209–219. 24273204 10.1002/ana.24070 PMC4386653

[547] J. Haslund‐Vinding , G. McBean , V. Jaquet , and F. Vilhardt , “NADPH oxidases in oxidant production by microglia: Activating receptors, pharmacology and association With disease,” British Journal of Pharmacology 174, no. 12 (2017): 1733–1749. 26750203 10.1111/bph.13425 PMC5446574

[548] S. L. Joice , F. Mydeen , P. O. Couraud , et al., “Modulation of blood‐brain barrier permeability by neutrophils: In vitro and in vivo studies,” Brain Research 1298 (2009): 13–23. 19728990 10.1016/j.brainres.2009.08.076

[549] S. Deng , P. Jin , P. Sherchan , et al., “Recombinant CCL17‐dependent CCR4 activation alleviates neuroinflammation and neuronal apoptosis Through the PI3K/AKT/Foxo1 signaling pathway After ICH in mice,” J Neuroinflammation 18, no. 1 (2021): 62. 33648537 10.1186/s12974-021-02112-3 PMC7923481

[550] Y. Tao , Y. Murakami , D. G. Vavvas , and K. H. Sonoda , “Necroptosis and Neuroinflammation in Retinal Degeneration,” Frontiers in neuroscience 16 (2022): 911430. 35844208 10.3389/fnins.2022.911430 PMC9277228

[551] M. K. Khoury , K. Gupta , S. R. Franco , and B. Liu , “Necroptosis in the Pathophysiology of Disease,” The American Journal of Pathology 190, no. 2 (2020): 272–285. 31783008 10.1016/j.ajpath.2019.10.012 PMC6983729

[552] R. Weinlich , A. Oberst , H. M. Beere , and D. R. Green , “Necroptosis in development, inflammation and disease,” Nature Reviews Molecular Cell Biology 18, no. 2 (2017): 127–136. 27999438 10.1038/nrm.2016.149

[553] P. Xu , X. Zhang , Q. Liu , et al., “Microglial TREM‐1 receptor mediates neuroinflammatory injury via interaction With SYK in experimental ischemic stroke,” Cell death & disease 10, no. 8 (2019): 555. 31324751 10.1038/s41419-019-1777-9 PMC6642102

[554] R. Ding , H. Li , Y. Liu , et al., “Activating cGAS‐STING axis contributes to neuroinflammation in CVST mouse model and induces inflammasome activation and microglia pyroptosis,” Journal of Neuroinflammation 19, no. 1 (2022): 137. 35689216 10.1186/s12974-022-02511-0 PMC9188164

[555] C. Zhao and W. Zhao , “NLRP3 Inflammasome‐A Key Player in Antiviral Responses,” Frontiers in Immunology 11 (2020): 211. 32133002 10.3389/fimmu.2020.00211 PMC7040071

[556] L. Gu , M. Sun , R. Li , et al., “Didymin Suppresses Microglia Pyroptosis and Neuroinflammation Through the Asc/Caspase‐1/GSDMD Pathway Following Experimental Intracerebral Hemorrhage,” Frontiers in Immunology 13 (2022): 810582. 35154128 10.3389/fimmu.2022.810582 PMC8828494

[557] D. S. Tian , C. Y. Li , C. Qin , M. Murugan , L. J. Wu , and J. L. Liu , “Deficiency in the voltage‐gated proton channel Hv1 increases M2 polarization of microglia and attenuates brain damage From photothrombotic ischemic stroke,” Journal of Neurochemistry 139, no. 1 (2016): 96–105. 27470181 10.1111/jnc.13751 PMC5037018

[558] J. Wang and S. Doré , “Inflammation After intracerebral hemorrhage,” Journal of Cerebral Blood Flow and Metabolism: Official Journal of the International Society of Cerebral Blood Flow and Metabolism 27, no. 5 (2007): 894–908. 17033693 10.1038/sj.jcbfm.9600403

[559] Z. Dong , Q. Peng , K. Pan , W. Lin , and Y. Wang , “Microglial and Neuronal Cell Pyroptosis Induced by Oxygen‐Glucose Deprivation/Reoxygenation Aggravates Cell Injury via Activation of the Caspase‐1/GSDMD Signaling Pathway,” Neurochemical Research 48, no. 9 (2023): 2660–2673. 37067736 10.1007/s11064-023-03931-x

[560] X. Lin , H. Ye , F. Siaw‐Debrah , et al., “AC‐YVAD‐CMK Inhibits Pyroptosis and Improves Functional Outcome After Intracerebral Hemorrhage,” BioMed Research International 2018 (2018): 3706047. 30410928 10.1155/2018/3706047 PMC6206581

[561] L. Gu , H. Chen , R. Geng , et al., “Single‐cell and Spatial Transcriptomics Reveals Ferroptosis as The Most Enriched Programmed Cell Death Process in Hemorrhage Stroke‐induced Oligodendrocyte‐mediated White Matter Injury,” International Journal of Biological Sciences 20, no. 10 (2024): 3842–3862. 39113700 10.7150/ijbs.96262 PMC11302879

[562] J. Wan , H. Ren , and J. Wang , “Iron toxicity, lipid peroxidation and ferroptosis After intracerebral haemorrhage,” Stroke and Vascular Neurology 4, no. 2 (2019): 93–95. 31338218 10.1136/svn-2018-000205 PMC6613877

[563] Y. Wei , X. Song , Y. Gao , Y. Gao , Y. Li , and L. Gu , “Iron toxicity in intracerebral hemorrhage: Physiopathological and therapeutic implications,” Brain Research Bulletin 178 (2022): 144–154. 34838852 10.1016/j.brainresbull.2021.11.014

[564] W. Zhang , Z. F. Yan , J. H. Gao , et al., “Role and mechanism of microglial activation in iron‐induced selective and progressive dopaminergic neurodegeneration,” Molecular Neurobiology 49, no. 3 (2014): 1153–1165. 24277523 10.1007/s12035-013-8586-4 PMC4878835

[565] D. B. Zorov , M. Juhaszova , and S. J. Sollott , “Mitochondrial ROS‐induced ROS release: An update and review,” Biochimica Et Biophysica Acta 1757, no. 5‐6 (2006): 509–517. 16829228 10.1016/j.bbabio.2006.04.029

[566] R. Xu , D. Zhu , J. Guo , and C. Wang , “IL‐18 Promotes Erythrophagocytosis and Erythrocyte Degradation by M1 Macrophages in a Calcific Microenvironment,” The Canadian Journal of Cardiology 37, no. 9 (2021): 1460–1471. 33984428 10.1016/j.cjca.2021.04.007

[567] Y. Fang , X. Chen , Q. Tan , H. Zhou , J. Xu , and Q. Gu , “Inhibiting Ferroptosis Through Disrupting the NCOA4‐FTH1 Interaction: A New Mechanism of Action,” ACS Central Science 7, no. 6 (2021): 980–989. 34235259 10.1021/acscentsci.0c01592 PMC8227600

[568] S. Gao , S. Jia , L. Bai , D. Li , and C. Meng , “Transcriptome Analysis Unveils That Exosomes Derived From M1‐Polarized Microglia Induce Ferroptosis of Neuronal Cells,” Cells 11, no. 24 (2022): 3956. 36552720 10.3390/cells11243956 PMC9776787

[569] N. Schurhoff and M. Toborek , “Circadian rhythms in the blood‐brain barrier: Impact on neurological disorders and stress responses,” Molecular Brain 16, no. 1 (2023): 5. 36635730 10.1186/s13041-023-00997-0 PMC9835375

[570] Y. Huang , S. Chen , Y. Luo , and Z. Han , “Crosstalk Between Inflammation and the BBB in Stroke,” Current Neuropharmacology 18, no. 12 (2020): 1227–1236. 32562523 10.2174/1570159X18666200620230321 PMC7770647

[571] P. B. Pun , J. Lu , and S. Moochhala , “Involvement of ROS in BBB dysfunction,” Free Radical Research 43, no. 4 (2009): 348–364. 19241241 10.1080/10715760902751902

[572] X. Li , Y. Cai , Z. Zhang , and J. Zhou , “Glial and Vascular Cell Regulation of the Blood‐Brain Barrier in Diabetes,” Diabetes & Metabolism Journal 46, no. 2 (2022): 222–238. 35299293 10.4093/dmj.2021.0146 PMC8987684

[573] A.‐Q. Chen , Z. Fang , X.‐L. Chen , et al., “Microglia‐derived TNF‐α mediates endothelial necroptosis aggravating blood brain‐barrier disruption After ischemic stroke,” Cell Death & Disease 10, no. 7 (2019): 487. 31221990 10.1038/s41419-019-1716-9 PMC6586814

[574] J. Pan , J. Peng , X. Li , H. Wang , X. Rong , and Y. Peng , “Transmission of NLRP3‐IL‐1β Signals in Cerebral Ischemia and Reperfusion Injury: From Microglia to Adjacent Neuron and Endothelial Cells via IL‐1β/IL‐1R1/TRAF6,” Molecular Neurobiology 60, no. 5 (2023): 2749–2766. 36717480 10.1007/s12035-023-03232-y

[575] J. H. Yun , “Interleukin‐1β induces pericyte apoptosis via the NF‐κB pathway in diabetic retinopathy,” Biochemical and Biophysical Research Communications 546 (2021): 46–53. 33571904 10.1016/j.bbrc.2021.01.108

[576] P. T. Ronaldson and T. P. Davis , “Regulation of blood‐brain barrier integrity by microglia in health and disease: A therapeutic opportunity,” Journal of Cerebral Blood Flow and Metabolism: Official Journal of the International Society of Cerebral Blood Flow and Metabolism 40, no. 1 (2020): S6–s24. 32928017 10.1177/0271678X20951995 PMC7687032

[577] M. Chaturvedi and L. Kaczmarek , “Mmp‐9 inhibition: A therapeutic strategy in ischemic stroke,” Molecular Neurobiology 49, no. 1 (2014): 563–573. 24026771 10.1007/s12035-013-8538-z PMC3918117

[578] D. I. Hadjiev and P. P. Mineva , “A reappraisal of the definition and pathophysiology of the transient ischemic attack,” Medical Science Monitor: International Medical Journal of Experimental and Clinical Research 13, no. 3 (2007): Ra50–Ra53. 17325647

[579] T. H. Jones , R. B. Morawetz , R. M. Crowell , et al., “Thresholds of focal cerebral ischemia in awake monkeys,” Journal of Neurosurgery 54, no. 6 (1981): 773–782. 7241187 10.3171/jns.1981.54.6.0773

[580] A. M. Kaufmann , A. D. Firlik , M. B. Fukui , L. R. Wechsler , C. A. Jungries , and H. Yonas , “Ischemic core and penumbra in human stroke,” Stroke; A Journal of Cerebral Circulation 30, no. 1 (1999): 93–99. 10.1161/01.str.30.1.93 9880395

[581] E. Gusev and V. Skvortsova , Cerebral Ischemia [in Russian] (Meditsina Moscow 2001).

[582] C. J. Chen , S. L. Liao , W. Y. Chen , J. S. Hong , and J. S. Kuo , “Cerebral ischemia/reperfusion injury in rat brain: Effects of naloxone,” Neuroreport 12, no. 6 (2001): 1245–1249. 11338200 10.1097/00001756-200105080-00038

[583] A. M. Planas , S. Solé , and C. Justicia , “Expression and activation of matrix metalloproteinase‐2 and ‐9 in rat brain After transient focal cerebral ischemia,” Neurobiology of Disease 8, no. 5 (2001): 834–846. 11592852 10.1006/nbdi.2001.0435

[584] F. R. Sharp , A. Lu , Y. Tang , and D. E. Millhorn , “Multiple molecular penumbras After focal cerebral ischemia,” Journal of Cerebral Blood Flow and Metabolism: Official Journal of the International Society of Cerebral Blood Flow and Metabolism 20, no. 7 (2000): 1011–1032. 10908035 10.1097/00004647-200007000-00001

[585] S. M. Uniken Venema , J. W. Dankbaar , A. van der Lugt , D. W. J. Dippel , and H. B. van der Worp , “Cerebral Collateral Circulation in the Era of Reperfusion Therapies for Acute Ischemic Stroke,” Stroke; A Journal of Cerebral Circulation 53, no. 10 (2022): 3222–3234. 10.1161/STROKEAHA.121.037869 35938420

[586] Tissue plasminogen activator for acute ischemic stroke. New England Journal of Medicine 1995;333(24):1581–1587. 7477192 10.1056/NEJM199512143332401

[587] S. Külkens and W. Hacke , “Thrombolysis With alteplase for acute ischemic stroke: Review of SITS‐MOST and other Phase IV studies,” Expert Review of Neurotherapeutics 7, no. 7 (2007): 783–788. 17610385 10.1586/14737175.7.7.783

[588] M. Yang , L. Tang , S. Bing , and X. Tang , “Association Between fibrinogen‐to‐albumin ratio and hemorrhagic transformation After intravenous thrombolysis in ischemic stroke patients,” Neurological Sciences: Official Journal of the Italian Neurological Society and of the Italian Society of Clinical Neurophysiology 44, no. 4 (2023): 1281–1288. 36529794 10.1007/s10072-022-06544-4

[589] R. Wen , M. Wang , W. Bian , et al., “Machine learning‐based prediction of symptomatic intracerebral hemorrhage After intravenous thrombolysis for stroke: A large multicenter study,” Frontiers in Neurology 14 (2023): 1247492. 37928151 10.3389/fneur.2023.1247492 PMC10624225

[590] M. Romoli , L. Vandelli , G. Bigliardi , et al., “Fibrinogen Depletion Coagulopathy Predicts Major Bleeding After Thrombolysis for Ischemic Stroke: A Multicenter Study,” Stroke; A Journal of Cerebral Circulation 53, no. 12 (2022): 3671–3678. 10.1161/STROKEAHA.122.039652 36039754

[591] T. Chen , Y. Cui , and H. S. Chen , “The association of gender With functional outcome in thrombolysed stroke: A secondary analysis of INTRECIS study,” Heliyon 10, no. 11 (2024): e32630. 38961923 10.1016/j.heliyon.2024.e32630 PMC11219987

[592] C. Duan , Y. Xiong , H. Q. Gu , et al., “Outcomes in minor stroke patients treated With intravenous thrombolysis,” CNS neuroscience & therapeutics 29, no. 8 (2023): 2308–2317. 36942504 10.1111/cns.14164 PMC10352890

[593] G. Xie , G. Jiang , L. Huang , et al., “Asparagine Endopeptidase Inhibition Attenuates Tissue Plasminogen Activator‐Induced Brain Hemorrhagic Transformation After Ischemic Stroke,” CNS neuroscience & therapeutics 31, no. 3 (2025): e70345. 40116141 10.1111/cns.70345 PMC11926568

[594] R. Sharma and K. Lee , “Advances in treatments for acute ischemic stroke,” BMJ (Clinical Research Ed) 389 (2025): e076161. 10.1136/bmj-2023-076161 40335091

[595] A. P. Jadhav , S. M. Desai , and T. G. Jovin , “Indications for Mechanical Thrombectomy for Acute Ischemic Stroke: Current Guidelines and Beyond,” Neurology 97, no. 20 Suppl 2 (2021): S126–s136. 34785611 10.1212/WNL.0000000000012801

[596] X. Guo , Y. Xiong , X. Huang , et al., “Aspiration versus stent retriever for posterior circulation stroke: A meta‐analysis,” CNS neuroscience & therapeutics 29, no. 2 (2023): 525–537. 36513959 10.1111/cns.14045 PMC9873527

[597] H. Fan , Z. Li , Y. Li , et al., “Comparison of a direct aspiration first pass technique vs. stent retriever thrombectomy for the treatment of acute large vessel occlusion stroke in the anterior circulation With atrial fibrillation,” Frontiers in Neurology 14 (2023): 1138993. 36908589 10.3389/fneur.2023.1138993 PMC9998705

[598] M. S. Alqahtani , N. F. Alharbi , B. G. Alghamdi , et al., “Reversible CT Scan Hypodensity in Acute Ischemic Stroke Patient With Low Initial Alberta Stroke Program Early CT Score (ASPECTS) Following Endovascular Thrombectomy: A Case Report,” Cureus 15, no. 3 (2023): e36194. 37065395 10.7759/cureus.36194 PMC10104593

[599] D. Penders , M. Vanloon , B. Verbraeken , et al., “Correspondence: Microsurgical thrombectomy: Where the ancient art meets the new era,” Neurosurgical Review 47, no. 1 (2024): 800. 39407047 10.1007/s10143-024-03031-6

[600] R. G. Nogueira , A. P. Jadhav , D. C. Haussen , et al., “Thrombectomy 6 to 24 H After Stroke With a Mismatch Between Deficit and Infarct,” New England Journal of Medicine 378, no. 1 (2018): 11–21. 29129157 10.1056/NEJMoa1706442

[601] Q. Li , M. Abdalkader , J. E. Siegler , et al., “Mechanical Thrombectomy for Large Ischemic Stroke: A Systematic Review and Meta‐analysis,” Neurology 101, no. 9 (2023): e922–e932. 37277200 10.1212/WNL.0000000000207536 PMC10501098

[602] W. J. Powers , A. A. Rabinstein , T. Ackerson , et al., “Guidelines for the Early Management of Patients With Acute Ischemic Stroke: 2019 Update to the 2018 Guidelines for the Early Management of Acute Ischemic Stroke: A Guideline for Healthcare Professionals From the American Heart Association/American Stroke Association,” Stroke; A Journal of Cerebral Circulation 50, no. 12 (2019): e344–e418. 10.1161/STR.0000000000000211 31662037

[603] K. Fassbender , S. Walter , Y. Liu , et al., ““Mobile stroke unit” for hyperacute stroke treatment,” Stroke; A Journal of Cerebral Circulation 34, no. 6 (2003): e44. 10.1161/01.STR.0000075573.22885.3B 12750527

[604] S. S. Rajan , J. M. Yamal , M. Wang , et al., “A Prospective Multicenter Analysis of Mobile Stroke Unit Cost‐Effectiveness,” Annals of Neurology 97, no. 2 (2025): 209–221. 39625067 10.1002/ana.27105

[605] J. C. Grotta , J. M. Yamal , S. A. Parker , et al., “Prospective, Multicenter, Controlled Trial of Mobile Stroke Units,” New England Journal of Medicine 385, no. 11 (2021): 971–981. 34496173 10.1056/NEJMoa2103879

[606] M. Ebinger , B. Siegerink , A. Kunz , et al., “Association Between Dispatch of Mobile Stroke Units and Functional Outcomes Among Patients With Acute Ischemic Stroke in Berlin,” Jama 325, no. 5 (2021): 454–466. 33528537 10.1001/jama.2020.26345 PMC7856548

[607] J. S. Rink , M. F. Froelich , M. Nour , et al., “Lifetime economic potential of mobile stroke units in acute stroke care: A model‐based analysis of the drivers of cost‐effectiveness,” Journal of Telemedicine and Telecare 30, no. 8 (2024): 1335–1344. 36484406 10.1177/1357633X221140951

[608] B. B. Navi , H. J. Audebert , A. W. Alexandrov , D. A. Cadilhac , and J. C. Grotta , “Mobile Stroke Units: Evidence, Gaps, and Next Steps,” Stroke; A Journal of Cerebral Circulation 53, no. 6 (2022): 2103–2113. 10.1161/STROKEAHA.121.037376 35331008

[609] Q. H. Guo , C. H. Liu , and J. G. Wang , “Blood Pressure Goals in Acute Stroke,” American Journal of Hypertension 35, no. 6 (2022): 483–499. 35323883 10.1093/ajh/hpac039 PMC9203067

[610] M. Kamieniarz‐Mędrygał and R. Kaźmierski , “Ambiguities in blood pressure management in acute ischaemic stroke,” Neurologia i Neurochirurgia Polska 56, no. 2 (2022): 131–140. 34622935 10.5603/PJNNS.a2021.0064

[611] E. A. Mistry , A. M. Mistry , M. O. Nakawah , et al., “Systolic Blood Pressure Within 24 H After Thrombectomy for Acute Ischemic Stroke Correlates With Outcome,” Journal of the American Heart Association 6, no. 5 (2017): e006167. 28522673 10.1161/JAHA.117.006167 PMC5524120

[612] H. Wang , Y. Guo , J. Xu , et al., “Blood pressure variability and outcome in atherosclerosis versus cardioembolism cerebral large vessel occlusion After successful thrombectomy,” Hypertension Research: Official Journal of the Japanese Society of Hypertension 47, no. 4 (2024): 898–909. 37978233 10.1038/s41440-023-01500-x

[613] M. Smith , U. Reddy , C. Robba , D. Sharma , and G. Citerio , “Acute ischaemic stroke: Challenges for the intensivist,” Intensive Care Medicine 45, no. 9 (2019): 1177–1189. 31346678 10.1007/s00134-019-05705-y

[614] C. S. Anderson , Y. Huang , R. I. Lindley , et al., “Intensive blood pressure reduction With intravenous thrombolysis therapy for acute ischaemic stroke (ENCHANTED): An international, randomised, open‐label, blinded‐endpoint, phase 3 trial,” Lancet 393, no. 10174 (2019): 877–888. 30739745 10.1016/S0140-6736(19)30038-8

[615] P. Yang , L. Song , Y. Zhang , et al., “Intensive blood pressure control After endovascular thrombectomy for acute ischaemic stroke (ENCHANTED2/MT): A multicentre, open‐label, blinded‐endpoint, randomised controlled trial,” Lancet 400, no. 10363 (2022): 1585–1596. 36341753 10.1016/S0140-6736(22)01882-7

[616] P. J. Lindsberg and R. O. Roine , “Hyperglycemia in acute stroke,” Stroke; A Journal of Cerebral Circulation 35, no. 2 (2004): 363–364. 10.1161/01.STR.0000115297.92132.84 14757880

[617] H. Dhungana , M. T. Huuskonen , M. Jaronen , et al., “Sulfosuccinimidyl oleate sodium is neuroprotective and alleviates stroke‐induced neuroinflammation,” Journal of Neuroinflammation 14, no. 1 (2017): 237. 29202856 10.1186/s12974-017-1010-7 PMC5716243

[618] C. Kersten , A. A. M. Zandbergen , O. A. Berkhemer , et al., “Association of hyperglycemia and computed tomographic perfusion deficits in patients who underwent endovascular treatment for acute ischemic stroke caused by a proximal intracranial occlusion: A subgroup analysis of a randomized phase 3 trial (MR CLEAN),” Journal of the Neurological Sciences 440 (2022): 120333. 35834861 10.1016/j.jns.2022.120333

[619] S. Nukui , H. Akiyama , K. Soga , et al., “Risk of Hyperglycemia and Hypoglycemia in Patients With Acute Ischemic Stroke Based on Continuous Glucose Monitoring,” Journal of Stroke and Cerebrovascular Diseases: the Official Journal of National Stroke Association 28, no. 12 (2019): 104346. 31548085 10.1016/j.jstrokecerebrovasdis.2019.104346

[620] N. K. Bains , W. Huang , B. R. French , F. Siddiq , C. R. Gomez , and A. I. Qureshi , “Hyperglycemic control in acute ischemic stroke patients undergoing endovascular treatment: Post hoc analysis of the Stroke Hyperglycemia Insulin Network Effort trial,” Journal of Neurointerventional Surgery 15, no. 4 (2023): 370–374. 35414602 10.1136/neurintsurg-2021-018485

[621] D. G. Hackam and J. D. Spence , “Antiplatelet Therapy in Ischemic Stroke and Transient Ischemic Attack,” Stroke; A Journal of Cerebral Circulation 50, no. 3 (2019): 773–778. 10.1161/STROKEAHA.118.023954 30626286

[622] K. S. Wong , Y. Wang , X. Leng , et al., “Early dual versus mono antiplatelet therapy for acute non‐cardioembolic ischemic stroke or transient ischemic attack: An updated systematic review and meta‐analysis,” Circulation 128, no. 15 (2013): 1656–1666. 24030500 10.1161/CIRCULATIONAHA.113.003187

[623] A. Stringberg , R. Camden , K. Qualls , and S. H. Naqvi , “Update on Dual Antiplatelet Therapy for Secondary Stroke Prevention,” Missouri Medicine 116, no. 4 (2019): 303–307. 31527979 PMC6699814

[624] J. A. Joglar , M. K. Chung , A. L. Armbruster , et al., “2023 ACC/AHA/ACCP/HRS Guideline for the Diagnosis and Management of Atrial Fibrillation: A Report of the American College of Cardiology/American Heart Association Joint Committee on Clinical Practice Guidelines,” Circulation 149, no. 1 (2024): e1–e156. 38033089 10.1161/CIR.0000000000001193 PMC11095842

[625] R. Oza , K. Rundell , and M. Garcellano , “Recurrent Ischemic Stroke: Strategies for Prevention,” American Family Physician 96, no. 7 (2017): 436–440. 29094912

[626] A. T. Patel , P. W. Duncan , S. M. Lai , and S. Studenski , “The relation Between impairments and functional outcomes poststroke,” Archives of Physical Medicine and Rehabilitation 81, no. 10 (2000): 1357–1363. 11030501 10.1053/apmr.2000.9397

[627] D. Hebert , M. P. Lindsay , A. McIntyre , et al., “Canadian stroke best practice recommendations: Stroke rehabilitation practice guidelines, update 2015,” International Journal of Stroke 11, no. 4 (2016): 459–484. 27079654 10.1177/1747493016643553

[628] C. J. Winstein , J. Stein , R. Arena , et al., “Guidelines for Adult Stroke Rehabilitation and Recovery: A Guideline for Healthcare Professionals From the American Heart Association/American Stroke Association,” Stroke; A Journal of Cerebral Circulation 47, no. 6 (2016): e98–e169. 10.1161/STR.0000000000000098 27145936

[629] B. H. Dobkin , “Strategies for stroke rehabilitation,” Lancet Neurology 3, no. 9 (2004): 528–536. 15324721 10.1016/S1474-4422(04)00851-8 PMC4164204

[630] Y. Xing and Y. Bai , “A Review of Exercise‐Induced Neuroplasticity in Ischemic Stroke: Pathology and Mechanisms,” Molecular Neurobiology 57, no. 10 (2020): 4218–4231. 32691303 10.1007/s12035-020-02021-1

[631] A. M. Moseley , A. Stark , I. D. Cameron , and A. Pollock , “Treadmill training and body weight support for walking After stroke,” The Cochrane Database of Systematic Reviews , no. 4 (2005): Cd002840. 10.1002/14651858.CD002840.pub2 16235304

[632] D. H. Saunders , M. Sanderson , S. Hayes , et al., “Physical fitness training for stroke patients,” The Cochrane Database of Systematic Reviews 3, no. 3 (2016): Cd003316. 27010219 10.1002/14651858.CD003316.pub6 PMC6464717

[633] C. Stein , C. G. Fritsch , C. Robinson , G. Sbruzzi , and R. D. Plentz , “Effects of Electrical Stimulation in Spastic Muscles After Stroke: Systematic Review and Meta‐Analysis of Randomized Controlled Trials,” Stroke; A Journal of Cerebral Circulation 46, no. 8 (2015): 2197–2205. 10.1161/STROKEAHA.115.009633 26173724

[634] J. Bradt , W. L. Magee , C. Dileo , B. L. Wheeler , and E. McGilloway , “Music therapy for acquired brain injury,” The Cochrane Database of Systematic Reviews , no. 7 (2010): Cd006787. 10.1002/14651858.CD006787.pub2 20614449

[635] C. Gómez Pajuelo , J. Pérez Naranjo , and I. Ruiz Martínez , “Treatment of mild to moderate hypertension With verapamil slow‐release in outpatients. Collaborative Group of the Spanish League for the Fight Against Hypertension,” Journal of Cardiovascular Pharmacology 13, no. Suppl 4 (1989): S50–S52. 10.1097/00005344-198900134-00014 2475687

[636] M. D. Hurd , I. Goel , Y. Sakai , and Y. Teramura , “Current status of ischemic stroke treatment: From thrombolysis to potential regenerative medicine,” Regenerative Therapy 18 (2021): 408–417. 34722837 10.1016/j.reth.2021.09.009 PMC8517544

[637] S. C. Cramer , B. Abila , N. E. Scott , M. Simeoni , and L. A. Enney , “Safety, pharmacokinetics, and pharmacodynamics of escalating repeat doses of GSK249320 in patients With stroke,” Stroke; A Journal of Cerebral Circulation 44, no. 5 (2013): 1337–1342. 10.1161/STROKEAHA.111.674366 23471268

[638] D. Wu , M. Zhang , Y. Tao , et al., “Characteristic ‘Pattern of Autofluorescence’ of the Fingernails and Certain Skin's Positions is a Novel Diagnostic Biomarker for Acute Ischemic Stroke: A Preliminary Study,” Phenomics (2025). 10.1007/s43657-024-00209-2 PMC12886686 41675276

[639] A. Bersano and L. Gatti , “Pathophysiology and Treatment of Stroke: Present Status and Future Perspectives,” International Journal of Molecular Sciences 24, no. 19 (2023): 14848. 37834297 10.3390/ijms241914848 PMC10573361

[640] D. T. Laskowitz , E. R. Bennett , R. J. Durham , et al., “Allogeneic Umbilical Cord Blood Infusion for Adults With Ischemic Stroke: Clinical Outcomes From a Phase I Safety Study,” Stem Cells Translational Medicine 7, no. 7 (2018): 521–529. 29752869 10.1002/sctm.18-0008 PMC6052613

[641] T.‐K. Lee , C.‐Y. Lu , S.‐T. Tsai , et al., “Complete Restoration of Motor Function in Acute Cerebral Stroke Treated With Allogeneic Human Umbilical Cord Blood Monocytes: Preliminary Results of a phase I Clinical Trial,” Cell Transplantation 30 (2021): 9636897211067447. 34939863 10.1177/09636897211067447 PMC8728774

[642] Y. Jiang , W. Zhu , J. Zhu , L. Wu , G. Xu , and X. Liu , “Feasibility of delivering mesenchymal stem cells via catheter to the proximal end of the lesion artery in patients With stroke in the territory of the middle cerebral artery,” Cell Transplantation 22, no. 12 (2013): 2291–2298. 23127560 10.3727/096368912X658818

[643] S. Banerjee , P. Bentley , M. Hamady , et al., “Intra‐Arterial Immunoselected CD34+ Stem Cells for Acute Ischemic Stroke,” Stem Cells Translational Medicine 3, no. 11 (2014): 1322–1330. 25107583 10.5966/sctm.2013-0178 PMC4214837

[644] T.‐L. Chiu , R. Baskaran , S.‐T. Tsai , et al., “Intracerebral transplantation of autologous adipose‐derived stem cells for chronic ischemic stroke: A phase I study,” Journal of Tissue Engineering and Regenerative Medicine 16, no. 1 (2022): 3–13. 34644444 10.1002/term.3256

[645] J. Fang , Y. Guo , S. Tan , et al., “Autologous Endothelial Progenitor Cells Transplantation for Acute Ischemic Stroke: A 4‐Year Follow‐Up Study,” Stem Cells Translational Medicine 8, no. 1 (2019): 14–21. 30156755 10.1002/sctm.18-0012 PMC6312444

[646] D.‐C. Chen , S.‐Z. Lin , J.‐R. Fan , et al., “Intracerebral implantation of autologous peripheral blood stem cells in stroke patients: A randomized phase II study,” Cell Transplantation 23, no. 12 (2014): 1599–1612. 24480430 10.3727/096368914X678562

[647] S. I. Savitz , V. Misra , M. Kasam , et al., “Intravenous autologous bone marrow mononuclear cells for ischemic stroke,” Annals of Neurology 70, no. 1 (2011): 59–69. 21786299 10.1002/ana.22458

[648] F. S. Vahidy , M. E. Haque , M. H. Rahbar , et al., “Intravenous Bone Marrow Mononuclear Cells for Acute Ischemic Stroke: Safety, Feasibility, and Effect Size From a Phase I Clinical Trial,” Stem Cells (Dayton, Ohio) 37, no. 11 (2019): 1481–1491. 31529663 10.1002/stem.3080

[649] M. A. G. Friedrich , M. P. Martins , M. D. Araújo , et al., “Intra‐arterial infusion of autologous bone marrow mononuclear cells in patients With moderate to severe middle cerebral artery acute ischemic stroke,” Cell Transplantation 21, no. Suppl 1 (2012): S13–S21. 22507676 10.3727/096368912x612512

[650] F. Moniche , J. A. Cabezas‐Rodriguez , R. Valverde , et al., “Safety and efficacy of intra‐arterial bone marrow mononuclear cell transplantation in patients With acute ischaemic stroke in Spain (IBIS trial): A phase 2, randomised, open‐label, standard‐of‐care controlled, multicentre trial,” The Lancet Neurology 22, no. 2 (2023): 137–146. 36681446 10.1016/S1474-4422(22)00526-9

[651] F. Moniche , A. Gonzalez , J.‐R. Gonzalez‐Marcos , et al., “Intra‐arterial bone marrow mononuclear cells in ischemic stroke: A pilot clinical trial,” Stroke; A Journal of Cerebral Circulation 43, no. 8 (2012): 2242–2244. 10.1161/STROKEAHA.112.659409 22764211

[652] A. Taguchi , C. Sakai , T. Soma , et al., “Intravenous Autologous Bone Marrow Mononuclear Cell Transplantation for Stroke: Phase1/2a Clinical Trial in a Homogeneous Group of Stroke Patients,” Stem Cells and Development 24, no. 19 (2015): 2207–2218. 26176265 10.1089/scd.2015.0160 PMC4582686

[653] E. de Celis‐Ruiz , B. Fuentes , M. Alonso de Leciñana , et al., “Final Results of Allogeneic Adipose Tissue‐Derived Mesenchymal Stem Cells in Acute Ischemic Stroke (AMASCIS): A Phase II, Randomized, Double‐Blind, Placebo‐Controlled, Single‐Center, Pilot Clinical Trial,” Cell Transplantation 31 (2022): 9636897221083863. 35301883 10.1177/09636897221083863 PMC8943307

[654] J.‐W. Chung , W. H. Chang , O. Y. Bang , et al., “Efficacy and Safety of Intravenous Mesenchymal Stem Cells for Ischemic Stroke,” Neurology 96, no. 7 (2021): e1012–e1023. 33472925 10.1212/WNL.0000000000011440

[655] K. Niizuma , S.‐I. Osawa , H. Endo , et al., “Randomized placebo‐controlled trial of CL2020, an allogenic muse cell‐based product, in subacute ischemic stroke,” Journal of Cerebral Blood Flow and Metabolism: Official Journal of the International Society of Cerebral Blood Flow and Metabolism 43, no. 12 (2023): 2029–2039. 37756573 10.1177/0271678X231202594 PMC10925866

[656] A. Jaillard , M. Hommel , A. Moisan , et al., “Autologous Mesenchymal Stem Cells Improve Motor Recovery in Subacute Ischemic Stroke: A Randomized Clinical Trial,” Translational Stroke Research 11, no. 5 (2020): 910–923. 32462427 10.1007/s12975-020-00787-z

[657] V. Bhatia , V. Gupta , D. Khurana , R. R. Sharma , and N. Khandelwal , “Randomized Assessment of the Safety and Efficacy of Intra‐Arterial Infusion of Autologous Stem Cells in Subacute Ischemic Stroke,” AJNR American Journal of Neuroradiology 39, no. 5 (2018): 899–904. 29545253 10.3174/ajnr.A5586 PMC7410650

[658] L.‐Y. Qiao , F.‐J. Huang , M. Zhao , et al., “A two‐year follow‐up study of cotransplantation With neural stem/progenitor cells and mesenchymal stromal cells in ischemic stroke patients,” Cell Transplantation 23, no. Suppl 1 (2014): S65–S72. 25333752 10.3727/096368914X684961

[659] L. Js , H. Jm , M. Gj , L. Ph , A. Yh , and B. Oy , “A long‐term follow‐up study of intravenous autologous mesenchymal stem cell transplantation in patients With ischemic stroke,” Stem Cells (Dayton, Ohio) 28, no. 6 (2010): 1099–1106. 20506226 10.1002/stem.430

[660] O. Y. Bang , E. H. Kim , Y. H. Cho , et al., “Circulating Extracellular Vesicles in Stroke Patients Treated With Mesenchymal Stem Cells: A Biomarker Analysis of a Randomized Trial,” Stroke; A Journal of Cerebral Circulation 53, no. 7 (2022): 2276–2286. 10.1161/STROKEAHA.121.036545 35341320

[661] J. Lee , W. H. Chang , J.‐W. Chung , et al., “Efficacy of Intravenous Mesenchymal Stem Cells for Motor Recovery After Ischemic Stroke: A Neuroimaging Study,” Stroke; A Journal of Cerebral Circulation 53, no. 1 (2022): 20–28. 10.1161/STROKEAHA.121.034505 34583525

[662] O. Y. Bang , J. S. Lee , P. H. Lee , and G. Lee , “Autologous mesenchymal stem cell transplantation in stroke patients,” Annals of Neurology 57, no. 6 (2005): 874–882. 15929052 10.1002/ana.20501

[663] K. Prasad , S. Mohanty , R. Bhatia , et al., “Autologous intravenous bone marrow mononuclear cell therapy for patients With subacute ischaemic stroke: A pilot study,” The Indian Journal of Medical Research 136, no. 2 (2012): 221–228. 22960888 PMC3461733

[664] L. M. Barbosa da Fonseca , B. Gutfilen , P. H. Rosado de Castro , et al., “Migration and homing of bone‐marrow mononuclear cells in chronic ischemic stroke After intra‐arterial injection,” Experimental Neurology 221, no. 1 (2010): 122–128. 19853605 10.1016/j.expneurol.2009.10.010

[665] M. Kawabori , S. Kuroda , H. Shichinohe , et al., “Intracerebral transplantation of MRI‐trackable autologous bone marrow stromal cells for patients With subacute ischemic stroke,” Med (New York, NY) 5, no. 5 (2024): 432–444. e4. 10.1016/j.medj.2024.02.009 38547868

[666] A. Bhasin , M. Srivastava , R. Bhatia , S. Mohanty , S. Kumaran , and S. Bose , “Autologous intravenous mononuclear stem cell therapy in chronic ischemic stroke,” Journal of Stem Cells & Regenerative Medicine 8, no. 3 (2012): 181–189. 24693196 10.46582/jsrm.0803011 PMC3908296

[667] V. Battistella , G. R. De Freitas , L. M. B. Da Fonseca , et al., “Safety of Autologous Bone Marrow Mononuclear Cell Transplantation in Patients With Nonacute Ischemic Stroke,” Regenerative Medicine 6, no. 1 (2011): 45–52. 21175286 10.2217/rme.10.97

[668] Z. K. Law , H. J. Tan , S. P. Chin , et al., “The effects of intravenous infusion of autologous mesenchymal stromal cells in patients With subacute middle cerebral artery infarct: A phase 2 randomized controlled trial on safety, tolerability and efficacy,” Cytotherapy 23, no. 9 (2021): 833–840. 33992536 10.1016/j.jcyt.2021.03.005

[669] O. Honmou , K. Houkin , T. Matsunaga , et al., “Intravenous administration of auto serum‐expanded autologous mesenchymal stem cells in stroke,” Brain: A Journal of Neurology 134, no. Pt 6 (2011): 1790–1807. 21493695 10.1093/brain/awr063 PMC3102237

[670] A. Taguchi , C. Sakai , T. Soma , et al., “Intravenous Autologous Bone Marrow Mononuclear Cell Transplantation for Stroke: Phase1/2a Clinical Trial in a Homogeneous Group of Stroke Patients,” Stem Cells and Development 24, no. 19 (2015): 2207–2218. 26176265 10.1089/scd.2015.0160 PMC4582686

[671] K. Prasad , A. Sharma , A. Garg , et al., “Intravenous autologous bone marrow mononuclear stem cell therapy for ischemic stroke: A multicentric, randomized trial,” Stroke; A Journal of Cerebral Circulation 45, no. 12 (2014): 3618–3624. 10.1161/STROKEAHA.114.007028 25378424

[672] A. A. Ghali , M. K. Yousef , O. A. Ragab , and E. A. ElZamarany , “Intra‐arterial Infusion of Autologous Bone Marrow Mononuclear Stem Cells in Subacute Ischemic Stroke Patients,” Frontiers in Neurology 7 (2016): 228. 28018286 10.3389/fneur.2016.00228 PMC5159483

[673] S. I. Savitz , D. Yavagal , G. Rappard , et al., “A Phase 2 Randomized, Sham‐Controlled Trial of Internal Carotid Artery Infusion of Autologous Bone Marrow‐Derived ALD‐401 Cells in Patients With Recent Stable Ischemic Stroke (RECOVER‐Stroke),” Circulation 139, no. 2 (2019): 192–205. 30586746 10.1161/CIRCULATIONAHA.117.030659

[674] M. L. Levy , J. R. Crawford , N. Dib , L. Verkh , N. Tankovich , and S. C. Cramer , “Phase I/II Study of Safety and Preliminary Efficacy of Intravenous Allogeneic Mesenchymal Stem Cells in Chronic Stroke,” Stroke; A Journal of Cerebral Circulation 50, no. 10 (2019): 2835–2841. 10.1161/STROKEAHA.119.026318 31495331

[675] A. Bhasin , M. V. P. Srivastava , S. Mohanty , R. Bhatia , S. S. Kumaran , and S. Bose , “Stem cell therapy: A clinical trial of stroke,” Clinical Neurology and Neurosurgery 115, no. 7 (2013): 1003–1008. 23183251 10.1016/j.clineuro.2012.10.015

[676] A. Bhasin , M. V. P. Srivastava , S. Mohanty , et al., “Paracrine Mechanisms of Intravenous Bone Marrow‐Derived Mononuclear Stem Cells in Chronic Ischemic Stroke,” Cerebrovascular Diseases Extra 6, no. 3 (2016): 107–119. 27846623 10.1159/000446404 PMC5123023

[677] A. Sharma , H. Sane , N. Gokulchandran , et al., “Autologous bone marrow mononuclear cells intrathecal transplantation in chronic stroke,” Stroke Research and Treatment 2014 (2014): 234095. 25126443 10.1155/2014/234095 PMC4121152

[678] A. Bhasin , S. S. Kumaran , R. Bhatia , S. Mohanty , and M. V. P. Srivastava , “Safety and Feasibility of Autologous Mesenchymal Stem Cell Transplantation in Chronic Stroke in Indian patients. A four‐year follow up,” Journal of Stem Cells & Regenerative Medicine 13, no. 1 (2017): 14–19. 28684893 10.46582/jsrm.1301003 PMC5494434

[679] C. Suárez‐Monteagudo , P. Hernández‐Ramírez , L. Alvarez‐González , et al., “Autologous bone marrow stem cell neurotransplantation in stroke patients. An open study,” Restorative Neurology and Neuroscience 27, no. 3 (2009): 151–161. 19531871 10.3233/RNN-2009-0483

[680] G. K. Steinberg , D. Kondziolka , L. R. Wechsler , et al., “Clinical Outcomes of Transplanted Modified Bone Marrow‐Derived Mesenchymal Stem Cells in Stroke: A Phase 1/2a Study,” Stroke; A Journal of Cerebral Circulation 47, no. 7 (2016): 1817–1824. 10.1161/STROKEAHA.116.012995 PMC5828512 27256670

[681] G. K. Steinberg , D. Kondziolka , L. R. Wechsler , et al., “Two‐year safety and clinical outcomes in chronic ischemic stroke patients after implantation of modified bone marrow‐derived mesenchymal stem cells (SB623): A phase 1/2a study,” Journal of Neurosurgery 131, no. 5 (2018): 1462–1472. 30497166 10.3171/2018.5.JNS173147

[682] K. W. Muir , D. Bulters , M. Willmot , et al., “Intracerebral implantation of human neural stem cells and motor recovery After stroke: Multicentre prospective single‐arm study (PISCES‐2),” Journal of Neurology, Neurosurgery, and Psychiatry 91, no. 4 (2020): 396–401. 32041820 10.1136/jnnp-2019-322515 PMC7147186

[683] E. S. Sussman and G. K. Steinberg , “A Focused Review of Clinical and Preclinical Studies of Cell‐Based Therapies in Stroke,” Neurosurgery 64, no. CN_suppl_1 (2017): 92–96. 28899062 10.1093/neuros/nyx329 PMC5901313

[684] J. Ya , J. Pellumbaj , A. Hashmat , and U. Bayraktutan , “The Role of Stem Cells as Therapeutics for Ischaemic Stroke,” Cells 13, no. 2 (2024): 112. 38247804 10.3390/cells13020112 PMC10814781

[685] B. Achón Buil , C. Tackenberg , and R. Rust , “Editing a gateway for cell therapy Across the blood‐brain barrier,” Brain: a Journal of Neurology 146, no. 3 (2023): 823–841. 36397727 10.1093/brain/awac393 PMC9976985

[686] S. I. Savitz , J. Dinsmore , J. Wu , G. V. Henderson , P. Stieg , and L. R. Caplan , “Neurotransplantation of fetal porcine cells in patients With basal ganglia infarcts: A preliminary safety and feasibility study,” Cerebrovascular Diseases (Basel, Switzerland) 20, no. 2 (2005): 101–107. 10.1159/000086518 15976503

[687] Y. Wang , C. Chang , R. Wang , X. Li , and X. Bao , “The advantages of multi‐level omics research on stem cell‐based therapies for ischemic stroke,” Neural Regeneration Research 19, no. 9 (2024): 1998–2003. 38227528 10.4103/1673-5374.390959 PMC11040296

[688] S. Yang , W. Lu , D. S. Zhou , and Y. Tang , “Enriched environment and white matter in aging brain,” Anatomical Record (Hoboken, NJ: 2007) 295, no. 9 (2012): 1406–1414. 10.1002/ar.22526 22777883

[689] Y. Sun , X. Jiang , and J. Gao , “Stem cell‐based ischemic stroke therapy: Novel modifications and clinical challenges,” Asian Journal of Pharmaceutical Sciences 19, no. 1 (2024): 100867. 38357525 10.1016/j.ajps.2023.100867 PMC10864855

[690] S. Zhang , B. B. Lachance , B. Moiz , and X. Jia , “Optimizing Stem Cell Therapy After Ischemic Brain Injury,” Journal of Stroke 22, no. 3 (2020): 286–305. 33053945 10.5853/jos.2019.03048 PMC7568970

[691] Y. Wang , X. Ji , R. K. Leak , F. Chen , and G. Cao , “Stem cell therapies in age‐related neurodegenerative diseases and stroke,” Ageing Research Reviews 34 (2017): 39–50. 27876573 10.1016/j.arr.2016.11.002 PMC5250574

[692] L. Li , Q. Jiang , G. Ding , et al., “Effects of administration route on migration and distribution of neural progenitor cells transplanted Into rats With focal cerebral ischemia, an MRI study,” Journal of Cerebral Blood Flow and Metabolism: Official Journal of the International Society of Cerebral Blood Flow and Metabolism 30, no. 3 (2010): 653–662. 19888287 10.1038/jcbfm.2009.238 PMC2844252

[693] K. N. Yarygin , D. D. Namestnikova , K. K. Sukhinich , I. L. Gubskiy , A. G. Majouga , and I. V. Kholodenko , “Cell Therapy of Stroke: Do the Intra‐Arterially Transplanted Mesenchymal Stem Cells Cross the Blood‐Brain Barrier?,” Cells 10, no. 11 (2021): 2997. 34831220 10.3390/cells10112997 PMC8616541

[694] J. Li , Q. Zhang , W. Wang , F. Lin , S. Wang , and J. Zhao , “Mesenchymal stem cell therapy for ischemic stroke: A look Into treatment mechanism and therapeutic potential,” Journal of Neurology 268, no. 11 (2021): 4095–4107. 32761505 10.1007/s00415-020-10138-5

[695] K. Rosenkranz , S. Kumbruch , K. Lebermann , et al., “The chemokine SDF‐1/CXCL12 contributes to the ‘homing’ of umbilical cord blood cells to a hypoxic‐ischemic lesion in the rat brain,” Journal of Neuroscience Research 88, no. 6 (2010): 1223–1233. 19937807 10.1002/jnr.22292

[696] C. H. Ryu , S. A. Park , S. M. Kim , et al., “Migration of human umbilical cord blood mesenchymal stem cells mediated by stromal cell‐derived factor‐1/CXCR4 axis via Akt, ERK, and p38 signal transduction pathways,” Biochemical and Biophysical Research Communications 398, no. 1 (2010): 105–110. 20558135 10.1016/j.bbrc.2010.06.043

[697] D.‐J. Chang , N. Lee , I.‐H. Park , et al., “Therapeutic potential of human induced pluripotent stem cells in experimental stroke,” Cell Transplantation 22, no. 8 (2013): 1427–1440. 23044029 10.3727/096368912X657314

[698] D. J. Chang , S. H. Oh , N. Lee , et al., “Contralaterally transplanted human embryonic stem cell‐derived neural precursor cells (ENStem‐A) migrate and improve brain functions in stroke‐damaged rats,” Experimental & Molecular Medicine 45, no. 11 (2013): e53. 24232252 10.1038/emm.2013.93 PMC3849578

[699] Y. Wang , Y. Deng , and G. Q. Zhou , “SDF‐1alpha/CXCR4‐mediated migration of systemically transplanted bone marrow stromal cells towards ischemic brain lesion in a rat model,” Brain Research 1195 (2008): 104–112. 18206136 10.1016/j.brainres.2007.11.068

[700] L. H. Shen , Y. Li , J. Chen , et al., “One‐year follow‐up After bone marrow stromal cell treatment in middle‐aged female rats With stroke,” Stroke; A Journal of Cerebral Circulation 38, no. 7 (2007): 2150–2156. 10.1161/STROKEAHA.106.481218 17525391

[701] X. He , J. Chen , Y. Zhong , et al., “Forebrain neural progenitors effectively integrate Into host brain circuits and improve neural function After ischemic stroke,” Nature Communications 16, no. 1 (2025): 5132. 10.1038/s41467-025-60187-5 PMC12134216 40461535

[702] X. Ji , Y. Zhou , Q. Gao , et al., “Functional reconstruction of the basal ganglia neural circuit by human striatal neurons in hypoxic‐ischaemic injured brain,” Brain: a Journal of Neurology 146, no. 2 (2023): 612–628. 36516880 10.1093/brain/awac358 PMC9924911

[703] S. H. Cameron , A. J. Alwakeel , L. Goddard , et al., “Delayed post‐treatment With bone marrow‐derived mesenchymal stem cells is neurorestorative of striatal medium‐spiny projection neurons and improves motor function After neonatal rat hypoxia‐ischemia,” Molecular and Cellular Neurosciences 68 (2015): 56–72. 25828540 10.1016/j.mcn.2015.03.019

[704] C. Hicks , L. Stevanato , R. P. Stroemer , E. Tang , S. Richardson , and J. D. Sinden , “In vivo and in vitro characterization of the angiogenic effect of CTX0E03 human neural stem cells,” Cell Transplantation 22, no. 9 (2013): 1541–1552. 23067568 10.3727/096368912X657936

[705] J. Shen , T. Zhang , H. Guan , X. Li , S. Zhang , and G. Xu , “PDGFR‐beta signaling mediates endogenous neurogenesis After postischemic neural stem/progenitor cell transplantation in mice,” Brain Injury 37, no. 12‐14 (2023): 1345–1354. 37975626 10.1080/02699052.2023.2280894

[706] O. Sadan , N. Shemesh , Y. Cohen , E. Melamed , and D. Offen , “Adult neurotrophic factor‐secreting stem cells: A potential novel therapy for neurodegenerative diseases,” The Israel Medical Association Journal: IMAJ 11, no. 4 (2009): 201–204. 19603590

[707] J. L. Yang , S. Mukda , and S. D. Chen , “Diverse roles of mitochondria in ischemic stroke,” Redox Biology 16 (2018): 263–275. 29549824 10.1016/j.redox.2018.03.002 PMC5854930

[708] P. Bakthavachalam and P. S. T. Shanmugam , “Mitochondrial dysfunction—Silent killer in cerebral ischemia,” Journal of the Neurological Sciences 375 (2017): 417–423. 28320180 10.1016/j.jns.2017.02.043

[709] K. Liu , K. Ji , L. Guo , et al., “Mesenchymal stem cells rescue injured endothelial cells in an in vitro ischemia‐reperfusion model via tunneling nanotube Like structure‐mediated mitochondrial transfer,” Microvascular Research 92 (2014): 10–18. 24486322 10.1016/j.mvr.2014.01.008

[710] D. Sarmah , A. Datta , N. Rana , et al., “SIRT‐1/RHOT‐1/PGC‐1α loop modulates mitochondrial biogenesis and transfer to offer resilience following endovascular stem cell therapy in ischemic stroke,” Free Radical Biology & Medicine 225 (2024): 255–274. 39306015 10.1016/j.freeradbiomed.2024.09.022

[711] K. Liu , L. Guo , Z. Zhou , M. Pan , and C. Yan , “Mesenchymal stem cells transfer mitochondria Into cerebral microvasculature and promote recovery From ischemic stroke,” Microvascular Research 123 (2019): 74–80. 30611747 10.1016/j.mvr.2019.01.001

[712] H. He , Q. Zeng , G. Huang , et al., “Bone marrow mesenchymal stem cell transplantation exerts neuroprotective effects following cerebral ischemia/reperfusion injury by inhibiting autophagy via the PI3K/Akt pathway,” Brain Research 1707 (2019): 124–132. 30448444 10.1016/j.brainres.2018.11.018

[713] I. Papazian , V. Kyrargyri , M. Evangelidou , A. Voulgari‐Kokota , and L. Probert , “Mesenchymal Stem Cell Protection of Neurons Against Glutamate Excitotoxicity Involves Reduction of NMDA‐Triggered Calcium Responses and Surface GluR1, and Is Partly Mediated by TNF,” International Journal of Molecular Sciences 19, no. 3 (2018): 651. 29495345 10.3390/ijms19030651 PMC5877512

[714] P. Zhang , J. Li , Y. Liu , et al., “Human neural stem cell transplantation attenuates apoptosis and improves neurological functions After cerebral ischemia in rats,” Acta Anaesthesiologica Scandinavica 53, no. 9 (2009): 1184–1191. 19650809 10.1111/j.1399-6576.2009.02024.x

[715] D. Kong , J. Zhu , Q. Liu , et al., “Mesenchymal stem cells protect neurons Against hypoxic‐ischemic injury via inhibiting parthanatos, necroptosis, and apoptosis, but not autophagy,” Cellular and Molecular Neurobiology 37, no. 2 (2017): 303–313. 27044018 10.1007/s10571-016-0370-3 PMC11482119

[716] D. Sarmah , A. Datta , H. Kaur , et al., “Sirtuin‐1 ‐ Mediated NF‐κB Pathway Modulation to Mitigate Inflammasome Signaling and Cellular Apoptosis is One of the Neuroprotective Effects of Intra‐arterial Mesenchymal Stem Cell Therapy Following Ischemic Stroke,” Stem Cell Reviews and Reports 18, no. 2 (2022): 821–838. 35112234 10.1007/s12015-021-10315-7

[717] M. Zhao , J. Wang , S. Zhu , et al., “Human neural stem cell‐derived exosomes activate PINK1/Parkin pathway to protect Against oxidative stress‐induced neuronal injury in ischemic stroke,” Journal of Translational Medicine 23, no. 1 (2025): 402. 40188077 10.1186/s12967-025-06283-y PMC11971779

[718] Q.‐S. Wang , R.‐J. Xiao , J. Peng , Z.‐T. Yu , J.‐Q. Fu , and Y. Xia , “Bone Marrow Mesenchymal Stem Cell‐Derived Exosomal KLF4 Alleviated Ischemic Stroke Through Inhibiting N6‐Methyladenosine Modification Level of Drp1 by Targeting lncRNA‐ZFAS1,” Molecular Neurobiology 60, no. 7 (2023): 3945–3962. 37002530 10.1007/s12035-023-03301-2

[719] Y. Li , J. Chen , L. Wang , M. Lu , and M. Chopp , “Treatment of stroke in rat With intracarotid administration of marrow stromal cells,” Neurology 56, no. 12 (2001): 1666–1672. 11425931 10.1212/wnl.56.12.1666

[720] K. Wakabayashi , A. Nagai , A. M. Sheikh , et al., “Transplantation of human mesenchymal stem cells promotes functional improvement and increased expression of neurotrophic factors in a rat focal cerebral ischemia model,” Journal of Neuroscience Research 88, no. 5 (2010): 1017–1025. 19885863 10.1002/jnr.22279

[721] A. Zacharek , A. Shehadah , J. Chen , et al., “Comparison of bone marrow stromal cells derived From stroke and normal rats for stroke treatment,” Stroke; A Journal of Cerebral Circulation 41, no. 3 (2010): 524–530. 10.1161/STROKEAHA.109.568881 PMC2847444 20056925

[722] H. Ghazavi , S. J. Hoseini , A. Ebrahimzadeh‐Bideskan , et al., “Fibroblast Growth Factor Type 1 (FGF1)‐Overexpressed Adipose‐Derived Mesenchaymal Stem Cells (AD‐MSC(FGF1)) Induce Neuroprotection and Functional Recovery in a Rat Stroke Model,” Stem Cell Reviews and Reports 13, no. 5 (2017): 670–685. 28795363 10.1007/s12015-017-9755-z

[723] X. Zong , S. Wu , F. Li , et al., “Transplantation of VEGF‐mediated bone marrow mesenchymal stem cells promotes functional improvement in a rat acute cerebral infarction model,” Brain Research 1676 (2017): 9–18. 28823954 10.1016/j.brainres.2017.08.006

[724] H. Duan , S. Li , P. Hao , et al., “Activation of endogenous neurogenesis and angiogenesis by basic fibroblast growth factor‐chitosan gel in an adult rat model of ischemic stroke,” Neural Regeneration Research 19, no. 2 (2024): 409–415. 37488905 10.4103/1673-5374.375344 PMC10503635

[725] Y. Moriyama , N. Takagi , K. Hashimura , C. Itokawa , and K. Tanonaka , “Intravenous injection of neural progenitor cells facilitates angiogenesis After cerebral ischemia,” Brain and Behavior 3, no. 2 (2013): 43–53. 23532762 10.1002/brb3.113 PMC3607146

[726] G.‐H. Ha , E. J. Kim , J. S. Park , et al., “JAK2/STAT3 pathway mediates neuroprotective and pro‐angiogenic treatment effects of adult human neural stem cells in middle cerebral artery occlusion stroke animal models,” Aging 14, no. 22 (2022): 8944–8969. 36446389 10.18632/aging.204410 PMC9740376

[727] H. Bao , S. Mao , X. Hu , et al., “Exosomal miR‐486 derived From bone marrow mesenchymal stem cells promotes angiogenesis following cerebral ischemic injury by regulating the PTEN/Akt pathway,” Scientific Reports 14, no. 1 (2024): 18086. 39103424 10.1038/s41598-024-69172-2 PMC11300871

[728] H. Hu , X. Hu , L. Li , et al., “Exosomes Derived From Bone Marrow Mesenchymal Stem Cells Promote Angiogenesis in Ischemic Stroke Mice via Upregulation of MiR‐21‐5p,” Biomolecules 12, no. 7 (2022): 883. 35883438 10.3390/biom12070883 PMC9313463

[729] Z. Cheng , L. Wang , M. Qu , et al., “Mesenchymal stem cells attenuate blood‐brain barrier leakage After cerebral ischemia in mice,” Journal of Neuroinflammation 15, no. 1 (2018): 135. 29724240 10.1186/s12974-018-1153-1 PMC5932816

[730] L. Huang , S. Wong , E. Y. Snyder , M. H. Hamblin , and J.‐P. Lee , “Human neural stem cells rapidly ameliorate symptomatic inflammation in early‐stage ischemic‐reperfusion cerebral injury,” Stem Cell Research & Therapy 5, no. 6 (2014): 129. 25418536 10.1186/scrt519 PMC4445985

[731] Y. Li , X. Quan , J. Hu , et al., “BMSCs‐derived small extracellular vesicles antagonize cerebral endothelial Caveolin‐1 driven autophagic degradation of tight‐junction proteins to protect blood‐brain barrier post‐stroke,” International Journal of Biological Sciences 21, no. 2 (2025): 842–859. 39781452 10.7150/ijbs.101937 PMC11705626

[732] Q. Li , X. Niu , Y. Yi , et al., “Inducible Pluripotent Stem Cell‐Derived Small Extracellular Vesicles Rejuvenate Senescent Blood‐Brain Barrier to Protect Against Ischemic Stroke in Aged Mice,” ACS Nano 17, no. 1 (2023): 775–789. 36562422 10.1021/acsnano.2c10824

[733] L. Calvo , N. Sobrino , R. Fernández de Soria , et al., “Distal hemoperfusion During coronary angioplasty With prolonged balloon inflation],” Revista Espanola De Cardiologia 42, no. 9 (1989): 587–592. Hemoperfusión distal durante la angioplastia coronaria con inflado prolongado de balón. 2533375

[734] T. Bliss , R. Guzman , M. Daadi , and G. K. Steinberg , “Cell transplantation therapy for stroke,” Stroke; A Journal of Cerebral Circulation 38, no. 2 Suppl (2007): 817–826. 10.1161/01.STR.0000247888.25985.62 17261746

[735] G. J. Delcroix , P. C. Schiller , J. P. Benoit , and C. N. Montero‐Menei , “Adult cell therapy for brain neuronal damages and the role of tissue engineering,” Biomaterials 31, no. 8 (2010): 2105–2120. 20005569 10.1016/j.biomaterials.2009.11.084

[736] Z. Li , X. Dong , M. Tian , et al., “Stem cell‐based therapies for ischemic stroke: A systematic review and meta‐analysis of clinical trials,” Stem Cell Research & Therapy 11, no. 1 (2020): 252. 32586371 10.1186/s13287-020-01762-z PMC7318436

[737] O. Y. Bang , E. H. Kim , J. M. Cha , and G. J. Moon , “Adult Stem Cell Therapy for Stroke: Challenges and Progress,” Journal of Stroke 18, no. 3 (2016): 256–266. 27733032 10.5853/jos.2016.01263 PMC5066440

[738] T. Osanai , S. Takamiya , Y. Morii , K. Ogasawara , K. Houkin , and M. Fujimura , “Efficacy and safety of stem cell therapy for acute and subacute ischemic stroke: A systematic review and meta‐analysis,” Scientific Reports 15, no. 1 (2025): 21214. 40595869 10.1038/s41598-025-04405-6 PMC12217828

[739] M. Kawabori , H. Shichinohe , S. Kuroda , and K. Houkin , “Clinical Trials of Stem Cell Therapy for Cerebral Ischemic Stroke,” International Journal of Molecular Sciences 21, no. 19 (2020): 7380. 33036265 10.3390/ijms21197380 PMC7582939

[740] K. Houkin , T. Osanai , S. Uchiyama , et al., “Allogeneic Stem Cell Therapy for Acute Ischemic Stroke: The Phase 2/3 TREASURE Randomized Clinical Trial,” JAMA Neurology 81, no. 2 (2024): 154–162. 38227308 10.1001/jamaneurol.2023.5200 PMC10792497

[741] M. A. Hawkes and A. A. Rabinstein , “Acute Hypertensive Response in Patients With Acute Intracerebral Hemorrhage: A Narrative Review,” Neurology 97, no. 7 (2021): 316–329. 34031208 10.1212/WNL.0000000000012276

[742] J. C. Hemphill 3rd , S. M. Greenberg , C. S. Anderson , et al., “Guidelines for the Management of Spontaneous Intracerebral Hemorrhage: A Guideline for Healthcare Professionals From the American Heart Association/American Stroke Association,” Stroke; A Journal of Cerebral Circulation 46, no. 7 (2015): 2032–2060. 10.1161/STR.0000000000000069 26022637

[743] T. J. Moullaali , X. Wang , R. H. Martin , et al., “Blood pressure control and clinical outcomes in acute intracerebral haemorrhage: A preplanned pooled analysis of individual participant data,” Lancet Neurology 18, no. 9 (2019): 857–864. 31397290 10.1016/S1474-4422(19)30196-6

[744] B. Cucchiara , S. Messe , L. Sansing , S. Kasner , and P. Lyden , “Hematoma growth in oral anticoagulant related intracerebral hemorrhage,” Stroke; A Journal of Cerebral Circulation 39, no. 11 (2008): 2993–2996. 10.1161/STROKEAHA.108.520668 18703803

[745] H. B. Huttner , P. D. Schellinger , M. Hartmann , et al., “Hematoma growth and outcome in treated neurocritical care patients With intracerebral hemorrhage related to oral anticoagulant therapy: Comparison of acute treatment strategies using vitamin K, fresh frozen plasma, and prothrombin complex concentrates,” Stroke; A Journal of Cerebral Circulation 37, no. 6 (2006): 1465–1470. 10.1161/01.STR.0000221786.81354.d6 16675739

[746] J. A. Frontera , J. J. Lewin 3rd , A. A. Rabinstein , et al., “Guideline for Reversal of Antithrombotics in Intracranial Hemorrhage: A Statement for Healthcare Professionals From the Neurocritical Care Society and Society of Critical Care Medicine,” Neurocritical Care 24, no. 1 (2016): 6–46. 26714677 10.1007/s12028-015-0222-x

[747] M. I. Baharoglu , C. Cordonnier , R. Al‐Shahi Salman , et al., “Platelet transfusion versus standard care After acute stroke due to spontaneous cerebral haemorrhage associated With antiplatelet therapy (PATCH): A randomised, open‐label, phase 3 trial,” Lancet 387, no. 10038 (2016): 2605–2613. 27178479 10.1016/S0140-6736(16)30392-0

[748] H. Kamel , B. B. Navi , K. Nakagawa , J. C. Hemphill 3rd , and N. U. Ko , “Hypertonic saline versus mannitol for the treatment of elevated intracranial pressure: A meta‐analysis of randomized clinical trials,” Critical Care Medicine 39, no. 3 (2011): 554–559. 21242790 10.1097/CCM.0b013e318206b9be

[749] A. M. Cook , G. Morgan Jones , G. W. J. Hawryluk , et al., “Guidelines for the Acute Treatment of Cerebral Edema in Neurocritical Care Patients,” Neurocritical Care 32, no. 3 (2020): 647–666. 32227294 10.1007/s12028-020-00959-7 PMC7272487

[750] S. Schwarz , K. Häfner , A. Aschoff , and S. Schwab , “Incidence and prognostic significance of fever following intracerebral hemorrhage,” Neurology 54, no. 2 (2000): 354–361. 10668696 10.1212/wnl.54.2.354

[751] T. S. Baker , J. Durbin , Z. Troiani , et al., “Therapeutic hypothermia for intracerebral hemorrhage: Systematic review and meta‐analysis of the experimental and clinical literature,” International Journal of Stroke 17, no. 5 (2022): 506–516. 34427479 10.1177/17474930211044870

[752] D. A. Godoy , G. R. Piñero , S. Svampa , F. Papa , and M. Di Napoli , “Hyperglycemia and short‐term outcome in patients With spontaneous intracerebral hemorrhage,” Neurocritical Care 9, no. 2 (2008): 217–229. 18300001 10.1007/s12028-008-9063-1

[753] A. I. Qureshi , Y. Y. Palesch , R. Martin , et al., “Association of serum glucose concentrations During acute hospitalization With hematoma expansion, perihematomal edema, and three month outcome Among patients With intracerebral hemorrhage,” Neurocritical Care 15, no. 3 (2011): 428–435. 21573860 10.1007/s12028-011-9541-8

[754] U. Pensato , C. Kaveeta , K. Tanaka , et al., “Initial intraventricular involvement and early intracerebral hematoma retraction: The 'ventricular washout,” International Journal of Stroke 10, no. 3 (2025): 748–756. 10.1177/23969873251330186 PMC11966627 40172123

[755] D. B. Herrick , N. Ullman , S. Nekoovaght‐Tak , et al., “Determinants of external ventricular drain placement and associated outcomes in patients With spontaneous intraventricular hemorrhage,” Neurocritical Care 21, no. 3 (2014): 426–434. 24522761 10.1007/s12028-014-9959-x

[756] S. T. Menacho , R. Grandhi , A. Delic , et al., “Impact of Intracranial Pressure Monitor‐Guided Therapy on Neurologic Outcome After Spontaneous Nontraumatic Intracranial Hemorrhage,” Journal of Stroke and Cerebrovascular Diseases: the Official Journal of National Stroke Association 30, no. 3 (2021): 105540. 33360250 10.1016/j.jstrokecerebrovasdis.2020.105540 PMC8080544

[757] T. Steiner , J. C. Purrucker , D. Aguiar de Sousa , et al., “European Stroke Organisation (ESO) and European Association of Neurosurgical Societies (EANS) guideline on stroke due to spontaneous intracerebral haemorrhage,” European Stroke Journal (2025): 23969873251340815. 10.1177/23969873251340815 PMC12098356 40401775

[758] D. F. Hanley , K. Lane , N. McBee , et al., “Thrombolytic removal of intraventricular haemorrhage in treatment of severe stroke: Results of the randomised, multicentre, multiregion, placebo‐controlled CLEAR III trial,” Lancet 389, no. 10069 (2017): 603–611. 28081952 10.1016/S0140-6736(16)32410-2 PMC6108339

[759] G. Pradilla , J. J. Ratcliff , A. J. Hall , et al., “Trial of Early Minimally Invasive Removal of Intracerebral Hemorrhage,” New England Journal of Medicine 390, no. 14 (2024): 1277–1289. 38598795 10.1056/NEJMoa2308440

[760] I. M. Ruff , A. de Havenon , D. L. Bergman , et al., “2024 AHA/ASA Performance and Quality Measures for Spontaneous Intracerebral Hemorrhage: A Report From the American Heart Association/American Stroke Association,” Stroke; A Journal of Cerebral Circulation 55, no. 7 (2024): e199–e230. 10.1161/STR.0000000000000464 38695183

[761] J. Ortiz‐Garcia , C. R. Gomez , M. J. Schneck , and J. Biller , “Recent advances in the management of transient ischemic attacks,” Faculty Reviews 11 (2022): 19. 35949262 10.12703/r/11-19 PMC9340656

[762] M. F. Giles and P. M. Rothwell , “Systematic review and pooled analysis of published and unpublished validations of the ABCD and ABCD2 transient ischemic attack risk scores,” Stroke; A Journal of Cerebral Circulation 41, no. 4 (2010): 667–673. 10.1161/STROKEAHA.109.571174 20185786

[763] L. M. Sanders , V. K. Srikanth , D. J. Blacker , D. J. Jolley , K. A. Cooper , and T. G. Phan , “Performance of the ABCD2 score for stroke risk post TIA: Meta‐analysis and probability modeling,” Neurology 79, no. 10 (2012): 971–980. 22700810 10.1212/WNL.0b013e31825f9d02

[764] G. W. Hosier , S. J. Phillips , S. P. Doucette , K. D. Magee , and G. J. Gubitz , “Transient ischemic attack: Management in the emergency department and impact of an outpatient neurovascular clinic,” Cjem 18, no. 5 (2016): 331–339. 26879765 10.1017/cem.2016.3

[765] T. Kiyohara , M. Kamouchi , Y. Kumai , et al., “ABCD3 and ABCD3‐I scores are superior to ABCD2 score in the prediction of short‐ and long‐term risks of stroke After transient ischemic attack,” Stroke; A Journal of Cerebral Circulation 45, no. 2 (2014): 418–425. 10.1161/STROKEAHA.113.003077 24335223

[766] D. O. Kleindorfer and A. Towfighi , “2021 Guideline for the Prevention of Stroke in Patients With Stroke and Transient Ischemic Attack: A Guideline From the American Heart Association/American Stroke Association,” Stroke; A Journal of Cerebral Circulation 52, no. 7 (2021): e364–e467. 10.1161/STR.0000000000000375 34024117

[767] H. Rahman , S. U. Khan , F. Nasir , T. Hammad , M. A. Meyer , and E. Kaluski , “Optimal Duration of Aspirin Plus Clopidogrel After Ischemic Stroke or Transient Ischemic Attack,” Stroke; A Journal of Cerebral Circulation 50, no. 4 (2019): 947–953. 10.1161/STROKEAHA.118.023978 PMC7457746 30852971

[768] I. Daghlas , S. C. Johnston , J. D. Easton , and A. S. Kim , “Baseline Stroke Risk and Efficacy of Dual‐Antiplatelet Therapy: A Post Hoc Analysis of the POINT Trial,” Stroke; A Journal of Cerebral Circulation 55, no. 2 (2024): 385–391. 10.1161/STROKEAHA.123.044927 PMC10857750 38174567

[769] Y. Wang , Y. Wang , X. Zhao , et al., “Clopidogrel With aspirin in acute minor stroke or transient ischemic attack,” New England Journal of Medicine 369, no. 1 (2013): 11–19. 23803136 10.1056/NEJMoa1215340

[770] B. Coll‐Vinent , A. Martín , J. Sánchez , et al., “Benefits of Emergency Departments' Contribution to Stroke Prophylaxis in Atrial Fibrillation: The EMERG‐AF Study (Emergency Department Stroke Prophylaxis and Guidelines Implementation in Atrial Fibrillation),” Stroke; A Journal of Cerebral Circulation 48, no. 5 (2017): 1344–1352. 10.1161/STROKEAHA.116.014855 PMC5404399 28389612

[771] R. G. Hart , L. A. Pearce , and M. I. Aguilar , “Meta‐analysis: Antithrombotic therapy to prevent stroke in patients who have nonvalvular atrial fibrillation,” Annals of Internal Medicine 146, no. 12 (2007): 857–867. 17577005 10.7326/0003-4819-146-12-200706190-00007

[772] M. Man‐Son‐Hing , G. Nichol , A. Lau , and A. Laupacis , “Choosing antithrombotic therapy for elderly patients With atrial fibrillation who are at risk for falls,” Archives of Internal Medicine 159, no. 7 (1999): 677–685. 10218746 10.1001/archinte.159.7.677

[773] L. A. Sposato , S. Chaturvedi , C. Y. Hsieh , C. A. Morillo , and H. Kamel , “Atrial Fibrillation Detected After Stroke and Transient Ischemic Attack: A Novel Clinical Concept Challenging Current Views,” Stroke; A Journal of Cerebral Circulation 53, no. 3 (2022): e94–e103. 10.1161/STROKEAHA.121.034777 34986652

[774] J. Davignon , “Beneficial cardiovascular pleiotropic effects of statins,” Circulation 109, no. 23 Suppl 1 (2004): Iii39–lii43. 15198965 10.1161/01.CIR.0000131517.20177.5a

[775] L. B. Goldstein , P. Amarenco , J. Zivin , et al., “Statin treatment and stroke outcome in the Stroke Prevention by Aggressive Reduction in Cholesterol Levels (SPARCL) trial,” Stroke; A Journal of Cerebral Circulation 40, no. 11 (2009): 3526–3531. 10.1161/STROKEAHA.109.557330 19745172

[776] D. Sagris , G. Ntaios , G. Georgiopoulos , et al., “Recommendations for lipid modification in patients With ischemic stroke or transient ischemic attack: A clinical guide by the Hellenic Stroke Organization and the Hellenic Atherosclerosis Society,” International Journal of Stroke 16, no. 6 (2021): 738–750. 33202196 10.1177/1747493020971970

[777] G. Arling , A. Perkins , L. J. Myers , J. J. Sico , and D. M. Bravata , “Blood Pressure Trajectories and Outcomes for Veterans Presenting at VA Medical Centers With a Stroke or Transient Ischemic Attack,” American Journal of Medicine 135, no. 7 (2022): 889–896. e1. 35292287 10.1016/j.amjmed.2022.02.012

[778] G. B. Boncoraglio , C. Del Giovane , and I. Tramacere , “Antihypertensive Drugs for Secondary Prevention After Ischemic Stroke or Transient Ischemic Attack: A Systematic Review and Meta‐Analysis,” Stroke; A Journal of Cerebral Circulation 52, no. 6 (2021): 1974–1982. 10.1161/STROKEAHA.120.031945 33902303

[779] W. Chen , Y. Pan , J. Jing , et al., “Recurrent Stroke in Minor Ischemic Stroke or Transient Ischemic Attack With Metabolic Syndrome and/or Diabetes Mellitus,” Journal of the American Heart Association 6, no. 6 (2017): e005446. 28572281 10.1161/JAHA.116.005446 PMC5669168

